# Supplementary material for: Trends of Mansonia (Diptera, Culicidae, Mansoniini) in Porto Velho: Seasonal patterns and meteorological influences
Source: PLoS One. 2024 May 8;19(5):e0303405. doi: 10.1371/journal.pone.0303405 (PMC11078429; doi:10.1371/journal.pone.0303405)
Supplement: S1 Table — The forecast analyses were carried out only with traps that remained in the field for a longer period: P01, P02, P03, P04 and P08. (PDF) [file pone.0303405.s001.pdf]

year	month	date	week	collection number	locality	trap code	family	subfamily	genus	species	amount
2019	Junho	03/06/2019	23	1	Teotônio	P03	Culicidae	Anophelinae	*Anopheles*	*Anopheles sp.*	1
2019	Junho	03/06/2019	23	1	Teotônio	P03	Culicidae	Culicinae	*Culex*	*Culex sp.*	32
2019	Junho	03/06/2019	23	1	Teotônio	P03	Culicidae	Culicinae	*Mansonia*	*Mansonia sp.*	6
2019	Junho	03/06/2019	23	1	Jaci Paraná	P06	Culicidae	Culicinae	*Culex*	*Culex sp.*	8
2019	Junho	03/06/2019	23	1	Jaci Paraná	P06	Culicidae	Culicinae	*Mansonia*	*Mansonia sp.*	4
2019	Junho	03/06/2019	23	1	São Domingos	P02	Culicidae	Culicinae	*Aedeomyia*	*Adeomyia squamipennis*	18
2019	Junho	03/06/2019	23	1	São Domingos	P02	Culicidae	Culicinae	*Coquillettidia*	*Coquillettidia sp.*	1
2019	Junho	03/06/2019	23	1	São Domingos	P02	Culicidae	Culicinae	*Culex*	*Culex sp.*	338
2019	Junho	03/06/2019	23	1	São Domingos	P02	Culicidae	Culicinae	*Mansonia*	*Mansonia sp.*	131
2019	Junho	03/06/2019	23	1	São Domingos	P02	Culicidae	Culicinae	*Uranotaenia*	*Uranotaenia sp.*	1
2019	Junho	10/06/2019	24	2	Teotônio	P03	Culicidae	Culicinae	*Aedeomyia*	*Adeomyia squamipennis*	5
2019	Junho	10/06/2019	24	2	Teotônio	P03	Culicidae	Anophelinae	*Anopheles*	*Anopheles sp.*	5
2019	Junho	10/06/2019	24	2	Teotônio	P03	Culicidae	Culicinae	*Culex*	*Culex sp.*	24
2019	Junho	10/06/2019	24	2	Teotônio	P03	Culicidae	Culicinae	*Mansonia*	*Mansonia humeralis*	1
2019	Junho	10/06/2019	24	2	Teotônio	P03	Culicidae	Culicinae	*Uranotaenia*	*Uranotaenia sp.*	1
2019	Junho	10/06/2019	24	2	Jaci Paraná	P06	Culicidae	Culicinae	*Aedeomyia*	*Adeomyia squamipennis*	3
2019	Junho	10/06/2019	24	2	Jaci Paraná	P06	Culicidae	Culicinae	*Aedes*	*Aedes aegypti*	13
2019	Junho	10/06/2019	24	2	Jaci Paraná	P06	Culicidae	Culicinae	*Culex*	*Culex sp.*	104
2019	Junho	10/06/2019	24	2	Jaci Paraná	P06	Culicidae	Culicinae	*Mansonia*	*Mansonia sp.*	8
2019	Junho	10/06/2019	24	2	São Domingos	P02	Culicidae	Culicinae	*Aedeomyia*	*Adeomyia squamipennis*	10
2019	Junho	10/06/2019	24	2	São Domingos	P02	Culicidae	Anophelinae	*Anopheles*	*Anopheles sp.*	1
2019	Junho	10/06/2019	24	2	São Domingos	P02	Culicidae	Culicinae	*Coquillettidia*	*Coquillettidia sp.*	1
2019	Junho	10/06/2019	24	2	São Domingos	P02	Culicidae	Culicinae	*Culex*	*Culex sp.*	151
2019	Junho	10/06/2019	24	2	São Domingos	P02	Culicidae	Culicinae	*Mansonia*	*Mansonia humeralis*	100
2019	Junho	17/06/2019	25	3	Teotônio	P03	Culicidae	Culicinae	*Culex*	*Culex sp.*	62
2019	Junho	17/06/2019	25	3	Teotônio	P03	Culicidae	Culicinae	*Mansonia*	*Mansonia sp.*	5
2019	Junho	17/06/2019	25	3	Jaci Paraná	P06	Culicidae	Culicinae	*Aedes*	*Aedes aegypti*	3
2019	Junho	17/06/2019	25	3	Jaci Paraná	P06	Culicidae	Culicinae	*Culex*	*Culex sp.*	99
2019	Junho	17/06/2019	25	3	Jaci Paraná	P06	Culicidae	Culicinae	*Mansonia*	*Mansonia sp.*	3
2019	Junho	17/06/2019	25	3	São Domingos	P02	Culicidae	Culicinae	*Aedeomyia*	*Adeomyia squamipennis*	6
2019	Junho	17/06/2019	25	3	São Domingos	P02	Culicidae	Culicinae	*Coquillettidia*	*Coquillettidia sp.*	11
2019	Junho	17/06/2019	25	3	São Domingos	P02	Culicidae	Culicinae	*Culex*	*Culex sp.*	371
2019	Junho	17/06/2019	25	3	São Domingos	P02	Culicidae	Culicinae	*Mansonia*	*Mansonia sp.*	153
2019	Junho	24/06/2019	26	4	Teotônio	P03	Culicidae	Culicinae	*Mansonia*	*Mansonia sp.*	3
2019	Junho	24/06/2019	26	4	Jaci Paraná	P06	Culicidae	Culicinae	*Aedeomyia*	*Adeomyia squamipennis*	1
2019	Junho	24/06/2019	26	4	Jaci Paraná	P06	Culicidae	Culicinae	*Culex*	*Culex sp.*	84
2019	Junho	24/06/2019	26	4	Jaci Paraná	P06	Culicidae	Culicinae	*Uranotaenia*	*Uranotaenia sp.*	2
2019	Junho	24/06/2019	26	4	São Domingos	P02	Culicidae	Culicinae	*Aedeomyia*	*Adeomyia squamipennis*	12
2019	Junho	24/06/2019	26	4	São Domingos	P02	Culicidae	Anophelinae	*Anopheles*	*Anopheles sp.*	2
2019	Junho	24/06/2019	26	4	São Domingos	P02	Culicidae	Culicinae	*Coquillettidia*	*Coquillettidia sp.*	15
2019	Junho	24/06/2019	26	4	São Domingos	P02	Culicidae	Culicinae	*Culex*	*Culex sp.*	416
2019	Junho	24/06/2019	26	4	São Domingos	P02	Culicidae	Culicinae	*Mansonia*	*Mansonia sp.*	117
2019	Julho	01/07/2019	27	5	Jaci Paraná	P06	Culicidae	Culicinae	*Aedes*	*Aedes aegypti*	7
2019	Julho	01/07/2019	27	5	Jaci Paraná	P06	Culicidae	Culicinae	*Culex*	*Culex sp.*	146
2019	Julho	01/07/2019	27	5	Jaci Paraná	P06	Culicidae	Culicinae	*Mansonia*	*Mansonia sp.*	17
2019	Julho	01/07/2019	27	5	Teotônio	P03	Culicidae	Culicinae	*Coquillettidia*	*Coquillettidia sp.*	1
2019	Julho	01/07/2019	27	5	Teotônio	P03	Culicidae	Culicinae	*Culex*	*Culex sp.*	4
2019	Julho	01/07/2019	27	5	Teotônio	P03	Culicidae	Culicinae	*Mansonia*	*Mansonia sp.*	4
2019	Julho	01/07/2019	27	5	São Domingos	P02	Culicidae	Culicinae	*Aedeomyia*	*Adeomyia squamipennis*	34
2019	Julho	01/07/2019	27	5	São Domingos	P02	Culicidae	Anophelinae	*Anopheles*	*Anopheles sp.*	2
2019	Julho	01/07/2019	27	5	São Domingos	P02	Culicidae	Culicinae	*Coquillettidia*	*Coquillettidia sp.*	8
2019	Julho	01/07/2019	27	5	São Domingos	P02	Culicidae	Culicinae	*Mansonia*	*Mansonia sp.*	197
2019	Julho	08/07/2019	28	6	Samauma	P08	Culicidae	Culicinae	*Aedeomyia*	*Adeomyia squamipennis*	7
2019	Julho	08/07/2019	28	6	Samauma	P08	Culicidae	Anophelinae	*Anopheles*	*Anopheles sp.*	2
2019	Julho	08/07/2019	28	6	Samauma	P08	Culicidae	Culicinae	*Coquillettidia*	*Coquillettidia sp.*	2
2019	Julho	08/07/2019	28	6	Samauma	P08	Culicidae	Culicinae	*Culex*	*Culex sp.*	1342
2019	Julho	08/07/2019	28	6	Samauma	P08	Culicidae	Culicinae	*Mansonia*	*Mansonia sp.*	905
2019	Julho	08/07/2019	28	6	Samauma	P08	Culicidae	Culicinae	*Uranotaenia*	*Uranotaenia sp.*	20
2019	Julho	08/07/2019	28	6	Jaci Paraná	P06	Culicidae	Culicinae	*Aedeomyia*	*Adeomyia squamipennis*	1
2019	Julho	08/07/2019	28	6	Jaci Paraná	P06	Culicidae	Culicinae	*Aedes*	*Aedes aegypti*	23
2019	Julho	08/07/2019	28	6	Jaci Paraná	P06	Culicidae	Culicinae	*Culex*	*Culex sp.*	192
2019	Julho	08/07/2019	28	6	Jaci Paraná	P06	Culicidae	Culicinae	*Mansonia*	*Mansonia sp.*	24
2019	Julho	08/07/2019	28	6	Santa Rita	P05	Culicidae	Culicinae	*Mansonia*	*Mansonia sp.*	25
2019	Julho	08/07/2019	28	6	Morrinhos	P04	Culicidae	Culicinae	*Aedes*	*Aedes sp.*	1
2019	Julho	08/07/2019	28	6	Morrinhos	P04	Culicidae	Anophelinae	*Anopheles*	*Anopheles sp.*	1
2019	Julho	08/07/2019	28	6	Morrinhos	P04	Culicidae	Culicinae	*Culex*	*Culex sp.*	7
2019	Julho	08/07/2019	28	6	Morrinhos	P04	Culicidae	Culicinae	*Mansonia*	*Mansonia sp.*	64
2019	Julho	08/07/2019	28	6	Teotônio	P03	Culicidae	Culicinae	*Aedeomyia*	*Adeomyia squamipennis*	11
2019	Julho	08/07/2019	28	6	Teotônio	P03	Culicidae	Anophelinae	*Anopheles*	*Anopheles sp.*	2
2019	Julho	08/07/2019	28	6	Teotônio	P03	Culicidae	Culicinae	*Coquillettidia*	*Coquillettidia sp.*	28
2019	Julho	08/07/2019	28	6	Teotônio	P03	Culicidae	Culicinae	*Culex*	*Culex sp.*	176
2019	Julho	08/07/2019	28	6	Teotônio	P03	Culicidae	Culicinae	*Mansonia*	*Mansonia sp.*	27
2019	Julho	08/07/2019	28	6	São Domingos	P02	Culicidae	Culicinae	*Aedeomyia*	*Adeomyia squamipennis*	14
2019	Julho	08/07/2019	28	6	São Domingos	P02	Culicidae	Anophelinae	*Anopheles*	*Anopheles sp.*	6
2019	Julho	08/07/2019	28	6	São Domingos	P02	Culicidae	Culicinae	*Coquillettidia*	*Coquillettidia sp.*	42
2019	Julho	08/07/2019	28	6	São Domingos	P02	Culicidae	Culicinae	*Culex*	*Culex sp.*	1323
2019	Julho	08/07/2019	28	6	São Domingos	P02	Culicidae	Culicinae	*Mansonia*	*Mansonia sp.*	315
2019	Julho	08/07/2019	28	6	Cujubim Grande	P01	Culicidae	Culicinae	*Culex*	*Culex sp.*	125
2019	Julho	08/07/2019	28	6	Cujubim Grande	P01	Culicidae	Culicinae	*Mansonia*	*Mansonia sp.*	9
2019	Julho	08/07/2019	28	6	Cujubim Grande	P01	Culicidae	Culicinae	*Uranotaenia*	*Uranotaenia sp.*	1
2019	Julho	15/07/2019	29	7	Rio Contra	P07	Culicidae	Anophelinae	*Anopheles*	*Anopheles sp.*	3
2019	Julho	15/07/2019	29	7	Rio Contra	P07	Culicidae	Culicinae	*Culex*	*Culex sp.*	14
2019	Julho	15/07/2019	29	7	Rio Contra	P07	Culicidae	Culicinae	*Mansonia*	*Mansonia sp.*	21
2019	Julho	15/07/2019	29	7	Rio Contra	P07	Culicidae	Culicinae	*Uranotaenia*	*Uranotaenia lowii*	1
2019	Julho	15/07/2019	29	7	Samauma	P08	Culicidae	Culicinae	*Aedeomyia*	*Adeomyia squamipennis*	5
2019	Julho	15/07/2019	29	7	Samauma	P08	Culicidae	Anophelinae	*Anopheles*	*Anopheles sp.*	1
2019	Julho	15/07/2019	29	7	Samauma	P08	Culicidae	Culicinae	*Culex*	*Culex sp.*	1928
2019	Julho	15/07/2019	29	7	Samauma	P08	Culicidae	Culicinae	*Mansonia*	*Mansonia sp.*	660
2019	Julho	15/07/2019	29	7	Samauma	P08	Culicidae	Culicinae	*Uranotaenia*	*Uranotaenia sp.*	6
2019	Julho	15/07/2019	29	7	Jaci Paraná	P06	Culicidae	Culicinae	*Aedes*	*Aedes sp.*	9
2019	Julho	15/07/2019	29	7	Jaci Paraná	P06	Culicidae	Culicinae	*Culex*	*Culex sp.*	132
2019	Julho	15/07/2019	29	7	Jaci Paraná	P06	Culicidae	Culicinae	*Mansonia*	*Mansonia sp.*	11
2019	Julho	15/07/2019	29	7	Santa Rita	P05	Culicidae	Culicinae	*Culex*	*Culex sp.*	8
2019	Julho	15/07/2019	29	7	Santa Rita	P05	Culicidae	Culicinae	*Mansonia*	*Mansonia sp.*	18
2019	Julho	15/07/2019	29	7	Morrinhos	P04	Culicidae	Culicinae	*Culex*	*Culex sp.*	4
2019	Julho	15/07/2019	29	7	Morrinhos	P04	Culicidae	Culicinae	*Mansonia*	*Mansonia sp.*	54
2019	Julho	15/07/2019	29	7	Teotônio	P03	Culicidae	Culicinae	*Aedes*	*Aedes sp.*	11
2019	Julho	15/07/2019	29	7	Teotônio	P03	Culicidae	Culicinae	*Coquillettidia*	*Coquillettidia sp.*	47
2019	Julho	15/07/2019	29	7	Teotônio	P03	Culicidae	Culicinae	*Culex*	*Culex sp.*	87
2019	Julho	15/07/2019	29	7	Teotônio	P03	Culicidae	Culicinae	*Mansonia*	*Mansonia sp.*	9
2019	Julho	15/07/2019	29	7	Teotônio	P03	Culicidae	Culicinae	*Psorophora*	*Psorophora sp.*	1
2019	Julho	15/07/2019	29	7	São Domingos	P02	Culicidae	Culicinae	*Aedeomyia*	*Adeomyia squamipennis*	17
2019	Julho	15/07/2019	29	7	São Domingos	P02	Culicidae	Culicinae	*Coquillettidia*	*Coquillettidia sp.*	18
2019	Julho	15/07/2019	29	7	São Domingos	P02	Culicidae	Culicinae	*Culex*	*Culex sp.*	502
2019	Julho	15/07/2019	29	7	São Domingos	P02	Culicidae	Culicinae	*Mansonia*	*Mansonia sp.*	112
2019	Julho	22/07/2019	30	8	Rio Contra	P07	Culicidae	Anophelinae	*Anopheles*	*Anopheles sp.*	2
2019	Julho	22/07/2019	30	8	Rio Contra	P07	Culicidae	Culicinae	*Culex*	*Culex sp.*	2
2019	Julho	22/07/2019	30	8	Rio Contra	P07	Culicidae	Culicinae	*Mansonia*	*Mansonia sp.*	17
2019	Julho	22/07/2019	30	8	Samauma	P08	Culicidae	Culicinae	*Aedeomyia*	*Adeomyia squamipennis*	7
2019	Julho	22/07/2019	30	8	Samauma	P08	Culicidae	Anophelinae	*Anopheles*	*Anopheles sp.*	4
2019	Julho	22/07/2019	30	8	Samauma	P08	Culicidae	Culicinae	*Coquillettidia*	*Coquillettidia sp.*	25
2019	Julho	22/07/2019	30	8	Samauma	P08	Culicidae	Culicinae	*Culex*	*Culex sp.*	1575
2019	Julho	22/07/2019	30	8	Samauma	P08	Culicidae	Culicinae	*Mansonia*	*Mansonia sp.*	692
2019	Julho	22/07/2019	30	8	Samauma	P08	Culicidae	Culicinae	*Uranotaenia*	*Uranotaenia sp.*	3
2019	Julho	22/07/2019	30	8	Jaci Paraná	P06	Culicidae	Culicinae	*Aedes*	*Aedes aegypti*	9
2019	Julho	22/07/2019	30	8	Jaci Paraná	P06	Culicidae	Culicinae	*Culex*	*Culex sp.*	79
2019	Julho	22/07/2019	30	8	Jaci Paraná	P06	Culicidae	Culicinae	*Mansonia*	*Mansonia sp.*	13

2019	Julho	22/07/2019	30	8	Santa Rita	P05	Culicidae	Culicinae	*Culex*	*Culex sp.*	3
2019	Julho	22/07/2019	30	8	Santa Rita	P05	Culicidae	Culicinae	*Mansonia*	*Mansonia sp.*	5
2019	Julho	22/07/2019	30	8	Morrinhos	P04	Culicidae	Culicinae	*Culex*	*Culex sp.*	7
2019	Julho	22/07/2019	30	8	Morrinhos	P04	Culicidae	Culicinae	*Mansonia*	*Mansonia sp.*	40
2019	Julho	22/07/2019	30	8	Teotônio	P03	Culicidae	Culicinae	*Aedeomyia*	*Adeomyia squamipennis*	4
2019	Julho	22/07/2019	30	8	Teotônio	P03	Culicidae	Culicinae	*Culex*	*Culex sp.*	80
2019	Julho	22/07/2019	30	8	São Domingos	P02	Culicidae	Culicinae	*Aedeomyia*	*Adeomyia squamipennis*	6
2019	Julho	22/07/2019	30	8	São Domingos	P02	Culicidae	Anophelinae	*Anopheles*	*Anopheles sp.*	1
2019	Julho	22/07/2019	30	8	São Domingos	P02	Culicidae	Culicinae	*Coquillettidia*	*Coquillettidia sp.*	21
2019	Julho	22/07/2019	30	8	São Domingos	P02	Culicidae	Culicinae	*Culex*	*Culex sp.*	131
2019	Julho	22/07/2019	30	8	São Domingos	P02	Culicidae	Culicinae	*Mansonia*	*Mansonia sp.*	106
2019	Julho	29/07/2019	31	9	Rio Contra	P07	Culicidae	Anophelinae	*Anopheles*	*Anopheles sp.*	7
2019	Julho	29/07/2019	31	9	Rio Contra	P07	Culicidae	Culicinae	*Mansonia*	*Mansonia sp.*	262
2019	Julho	29/07/2019	31	9	Samauma	P08	Culicidae	Culicinae	*Culex*	*Culex sp.*	477
2019	Julho	29/07/2019	31	9	Samauma	P08	Culicidae	Culicinae	*Mansonia*	*Mansonia sp.*	262
2019	Julho	29/07/2019	31	9	Jaci Paraná	P06	Culicidae	Culicinae	*Aedes*	*Aedes sp.*	3
2019	Julho	29/07/2019	31	9	Jaci Paraná	P06	Culicidae	Culicinae	*Culex*	*Culex sp.*	58
2019	Julho	29/07/2019	31	9	Jaci Paraná	P06	Culicidae	Culicinae	*Mansonia*	*Mansonia sp.*	5
2019	Julho	29/07/2019	31	9	Santa Rita	P05	Culicidae	Culicinae	*Mansonia*	*Mansonia sp.*	1
2019	Julho	29/07/2019	31	9	Morrinhos	P04	Culicidae	Culicinae	*Mansonia*	*Mansonia sp.*	5
2019	Julho	29/07/2019	31	9	Teotônio	P03	Culicidae	Culicinae	*Culex*	*Culex sp.*	25
2019	Julho	29/07/2019	31	9	Teotônio	P03	Culicidae	Culicinae	*Mansonia*	*Mansonia sp.*	1
2019	Julho	29/07/2019	31	9	São Domingos	P02	Culicidae	Culicinae	*Aedeomyia*	*Adeomyia squamipennis*	9
2019	Julho	29/07/2019	31	9	São Domingos	P02	Culicidae	Anophelinae	*Anopheles*	*Anopheles sp.*	2
2019	Julho	29/07/2019	31	9	São Domingos	P02	Culicidae	Culicinae	*Coquillettidia*	*Coquillettidia venezuelensis*	5
2019	Julho	29/07/2019	31	9	São Domingos	P02	Culicidae	Culicinae	*Culex*	*Culex sp.*	668
2019	Julho	29/07/2019	31	9	São Domingos	P02	Culicidae	Culicinae	*Mansonia*	*Mansonia sp.*	129
2019	Julho	29/07/2019	31	9	Cujubim Grande	P01	Culicidae	Anophelinae	*Anopheles*	*Anopheles sp.*	1
2019	Julho	29/07/2019	31	9	Cujubim Grande	P01	Culicidae	Culicinae	*Culex*	*Culex sp.*	352
2019	Julho	29/07/2019	31	9	Cujubim Grande	P01	Culicidae	Culicinae	*Mansonia*	*Mansonia sp.*	15
2019	Agosto	05/08/2019	32	10	Rio Contra	P07	Culicidae	Culicinae	*Aedeomyia*	*Adeomyia squamipennis*	2
2019	Agosto	05/08/2019	32	10	Rio Contra	P07	Culicidae	Culicinae	*Culex*	*Culex sp.*	4
2019	Agosto	05/08/2019	32	10	Rio Contra	P07	Culicidae	Culicinae	*Mansonia*	*Mansonia indubitans*	2
2019	Agosto	05/08/2019	32	10	Samauma	P08	Culicidae	Culicinae	*Culex*	*Culex sp.*	1400
2019	Agosto	05/08/2019	32	10	Samauma	P08	Culicidae	Culicinae	*Mansonia*	*Mansonia sp.*	104
2019	Agosto	05/08/2019	32	10	Jaci Paraná	P06	Culicidae	Culicinae	*Aedes*	*Aedes sp.*	3
2019	Agosto	05/08/2019	32	10	Jaci Paraná	P06	Culicidae	Culicinae	*Culex*	*Culex sp.*	50
2019	Agosto	05/08/2019	32	10	Jaci Paraná	P06	Culicidae	Culicinae	*Mansonia*	*Mansonia sp.*	2
2019	Agosto	05/08/2019	32	10	Santa Rita	P05	Culicidae	Culicinae	*Culex*	*Culex sp.*	75
2019	Agosto	05/08/2019	32	10	Santa Rita	P05	Culicidae	Culicinae	*Mansonia*	*Mansonia sp.*	4
2019	Agosto	05/08/2019	32	10	Morrinhos	P04	Culicidae	Culicinae	*Mansonia*	*Mansonia sp.*	7
2019	Agosto	05/08/2019	32	10	Teotônio	P03	Culicidae	Culicinae	*Aedeomyia*	*Adeomyia squamipennis*	2
2019	Agosto	05/08/2019	32	10	Teotônio	P03	Culicidae	Culicinae	*Coquillettidia*	*Coquillettidia sp.*	8
2019	Agosto	05/08/2019	32	10	Teotônio	P03	Culicidae	Culicinae	*Culex*	*Culex sp.*	58
2019	Agosto	05/08/2019	32	10	Teotônio	P03	Culicidae	Culicinae	*Mansonia*	*Mansonia sp.*	8
2019	Agosto	05/08/2019	32	10	São Domingos	P02	Culicidae	Culicinae	*Aedeomyia*	*Adeomyia squamipennis*	8
2019	Agosto	05/08/2019	32	10	São Domingos	P02	Culicidae	Culicinae	*Coquillettidia*	*Coquillettidia sp.*	10
2019	Agosto	05/08/2019	32	10	São Domingos	P02	Culicidae	Culicinae	*Culex*	*Culex sp.*	841
2019	Agosto	05/08/2019	32	10	São Domingos	P02	Culicidae	Culicinae	*Mansonia*	*Mansonia sp.*	123
2019	Agosto	05/08/2019	32	10	Cujubim Grande	P01	Culicidae	Anophelinae	*Anopheles*	*Anopheles sp.*	1
2019	Agosto	05/08/2019	32	10	Cujubim Grande	P01	Culicidae	Culicinae	*Culex*	*Culex sp.*	268
2019	Agosto	05/08/2019	32	10	Cujubim Grande	P01	Culicidae	Culicinae	*Mansonia*	*Mansonia sp.*	8
2019	Agosto	12/08/2019	33	11	Rio Contra	P07	Culicidae	Culicinae	*Culex*	*Culex sp.*	1
2019	Agosto	12/08/2019	33	11	Samauma	P08	Culicidae	Culicinae	*Coquillettidia*	*Coquillettidia sp.*	1
2019	Agosto	12/08/2019	33	11	Samauma	P08	Culicidae	Culicinae	*Culex*	*Culex sp.*	800
2019	Agosto	12/08/2019	33	11	Samauma	P08	Culicidae	Culicinae	*Mansonia*	*Mansonia sp.*	173
2019	Agosto	12/08/2019	33	11	Jaci Paraná	P06	Culicidae	Culicinae	*Aedes*	*Aedes sp.*	3
2019	Agosto	12/08/2019	33	11	Jaci Paraná	P06	Culicidae	Culicinae	*Culex*	*Culex sp.*	93
2019	Agosto	12/08/2019	33	11	Santa Rita	P05	Culicidae	Culicinae	*Culex*	*Culex sp.*	97
2019	Agosto	12/08/2019	33	11	Santa Rita	P05	Culicidae	Culicinae	*Mansonia*	*Mansonia humeralis*	6
2019	Agosto	12/08/2019	33	11	Morrinhos	P04	Culicidae	Culicinae	*Culex*	*Culex sp.*	3
2019	Agosto	12/08/2019	33	11	Morrinhos	P04	Culicidae	Culicinae	*Mansonia*	*Mansonia sp.*	15
2019	Agosto	12/08/2019	33	11	Teotônio	P03	Culicidae	Culicinae	*Aedeomyia*	*Adeomyia squamipennis*	9
2019	Agosto	12/08/2019	33	11	Teotônio	P03	Culicidae	Culicinae	*Coquillettidia*	*Coquillettidia sp.*	44
2019	Agosto	12/08/2019	33	11	Teotônio	P03	Culicidae	Culicinae	*Culex*	*Culex sp.*	133
2019	Agosto	12/08/2019	33	11	Teotônio	P03	Culicidae	Culicinae	*Mansonia*	*Mansonia sp.*	16
2019	Agosto	12/08/2019	33	11	São Domingos	P02	Culicidae	Culicinae	*Aedeomyia*	*Adeomyia squamipennis*	9
2019	Agosto	12/08/2019	33	11	São Domingos	P02	Culicidae	Culicinae	*Coquillettidia*	*Coquillettidia sp.*	53
2019	Agosto	12/08/2019	33	11	São Domingos	P02	Culicidae	Culicinae	*Culex*	*Culex sp.*	1197
2019	Agosto	12/08/2019	33	11	São Domingos	P02	Culicidae	Culicinae	*Mansonia*	*Mansonia sp.*	154
2019	Agosto	12/08/2019	33	11	Cujubim Grande	P01	Culicidae	Culicinae	*Aedeomyia*	*Adeomyia squamipennis*	1
2019	Agosto	12/08/2019	33	11	Cujubim Grande	P01	Culicidae	Culicinae	*Culex*	*Culex sp.*	61
2019	Agosto	12/08/2019	33	11	Cujubim Grande	P01	Culicidae	Culicinae	*Mansonia*	*Mansonia sp.*	8
2019	Agosto	19/08/2019	34	12	Rio Contra	P07	Culicidae	Culicinae	*Mansonia*	*Mansonia indubitans*	1
2019	Agosto	19/08/2019	34	12	Samauma	P08	Culicidae	Culicinae	*Aedeomyia*	*Adeomyia squamipennis*	2
2019	Agosto	19/08/2019	34	12	Samauma	P08	Culicidae	Culicinae	*Coquillettidia*	*Coquillettidia sp.*	1
2019	Agosto	19/08/2019	34	12	Samauma	P08	Culicidae	Culicinae	*Culex*	*Culex sp.*	240
2019	Agosto	19/08/2019	34	12	Samauma	P08	Culicidae	Culicinae	*Mansonia*	*Mansonia sp.*	99
2019	Agosto	19/08/2019	34	12	Samauma	P08	Culicidae	Culicinae	*Uranotaenia*	*Uranotaenia sp.*	1
2019	Agosto	19/08/2019	34	12	Jaci Paraná	P06	Culicidae	Culicinae	*Culex*	*Culex sp.*	107
2019	Agosto	19/08/2019	34	12	Jaci Paraná	P06	Culicidae	Culicinae	*Mansonia*	*Mansonia sp.*	4
2019	Agosto	19/08/2019	34	12	Santa Rita	P05	Culicidae	Culicinae	*Culex*	*Culex sp.*	200
2019	Agosto	19/08/2019	34	12	Santa Rita	P05	Culicidae	Culicinae	*Mansonia*	*Mansonia sp.*	14
2019	Agosto	19/08/2019	34	12	Teotônio	P03	Culicidae	Culicinae	*Aedeomyia*	*Adeomyia squamipennis*	3
2019	Agosto	19/08/2019	34	12	Teotônio	P03	Culicidae	Culicinae	*Coquillettidia*	*Coquillettidia sp.*	16
2019	Agosto	19/08/2019	34	12	Teotônio	P03	Culicidae	Culicinae	*Culex*	*Culex sp.*	70
2019	Agosto	19/08/2019	34	12	Teotônio	P03	Culicidae	Culicinae	*Mansonia*	*Mansonia sp.*	14
2019	Agosto	19/08/2019	34	12	São Domingos	P02	Culicidae	Culicinae	*Aedeomyia*	*Adeomyia squamipennis*	17
2019	Agosto	19/08/2019	34	12	São Domingos	P02	Culicidae	Culicinae	*Coquillettidia*	*Coquillettidia sp.*	19
2019	Agosto	19/08/2019	34	12	São Domingos	P02	Culicidae	Culicinae	*Culex*	*Culex sp.*	1052
2019	Agosto	19/08/2019	34	12	São Domingos	P02	Culicidae	Culicinae	*Mansonia*	*Mansonia humeralis*	87
2019	Agosto	19/08/2019	34	12	Cujubim Grande	P01	Culicidae	Culicinae	*Culex*	*Culex sp.*	288
2019	Agosto	19/08/2019	34	12	Cujubim Grande	P01	Culicidae	Culicinae	*Mansonia*	*Mansonia sp.*	4
2019	Agosto	26/08/2019	35	13	Rio Contra	P07	Culicidae	Culicinae	*Culex*	*Culex sp.*	1
2019	Agosto	26/08/2019	35	13	Samauma	P08	Culicidae	Culicinae	*Aedeomyia*	*Adeomyia squamipennis*	1
2019	Agosto	26/08/2019	35	13	Samauma	P08	Culicidae	Anophelinae	*Anopheles*	*Anopheles sp.*	1
2019	Agosto	26/08/2019	35	13	Samauma	P08	Culicidae	Culicinae	*Coquillettidia*	*Coquillettidia sp.*	6
2019	Agosto	26/08/2019	35	13	Samauma	P08	Culicidae	Culicinae	*Culex*	*Culex sp.*	980
2019	Agosto	26/08/2019	35	13	Samauma	P08	Culicidae	Culicinae	*Mansonia*	*Mansonia sp.*	285
2019	Agosto	26/08/2019	35	13	Jaci Paraná	P06	Culicidae	Culicinae	*Aedeomyia*	*Adeomyia squamipennis*	1
2019	Agosto	26/08/2019	35	13	Jaci Paraná	P06	Culicidae	Culicinae	*Aedes*	*Aedes sp.*	6
2019	Agosto	26/08/2019	35	13	Jaci Paraná	P06	Culicidae	Culicinae	*Coquillettidia*	*Coquillettidia sp.*	2
2019	Agosto	26/08/2019	35	13	Jaci Paraná	P06	Culicidae	Culicinae	*Culex*	*Culex sp.*	88
2019	Agosto	26/08/2019	35	13	Jaci Paraná	P06	Culicidae	Culicinae	*Mansonia*	*Mansonia titillans*	3
2019	Agosto	26/08/2019	35	13	Santa Rita	P05	Culicidae	Culicinae	*Culex*	*Culex sp.*	91
2019	Agosto	26/08/2019	35	13	Santa Rita	P05	Culicidae	Culicinae	*Mansonia*	*Mansonia sp.*	6
2019	Agosto	26/08/2019	35	13	Morrinhos	P04	Culicidae	Culicinae	*Culex*	*Culex sp.*	2
2019	Agosto	26/08/2019	35	13	Morrinhos	P04	Culicidae	Culicinae	*Mansonia*	*Mansonia sp.*	10
2019	Agosto	26/08/2019	35	13	Teotônio	P03	Culicidae	Culicinae	*Aedeomyia*	*Adeomyia squamipennis*	2
2019	Agosto	26/08/2019	35	13	Teotônio	P03	Culicidae	Culicinae	*Coquillettidia*	*Coquillettidia sp.*	3
2019	Agosto	26/08/2019	35	13	Teotônio	P03	Culicidae	Culicinae	*Culex*	*Culex sp.*	14
2019	Agosto	26/08/2019	35	13	Teotônio	P03	Culicidae	Culicinae	*Mansonia*	*Mansonia humeralis*	1
2019	Agosto	26/08/2019	35	13	São Domingos	P02	Culicidae	Culicinae	*Aedeomyia*	*Adeomyia squamipennis*	28
2019	Agosto	26/08/2019	35	13	São Domingos	P02	Culicidae	Culicinae	*Coquillettidia*	*Coquillettidia sp.*	19
2019	Agosto	26/08/2019	35	13	São Domingos	P02	Culicidae	Culicinae	*Culex*	*Culex sp.*	552
2019	Agosto	26/08/2019	35	13	São Domingos	P02	Culicidae	Culicinae	*Mansonia*	*Mansonia sp.*	134
2019	Agosto	26/08/2019	35	13	Cujubim Grande	P01	Culicidae	Culicinae	*Culex*	*Culex sp.*	37
2019	Setembro	02/09/2019	36	14	Rio Contra	P07	Culicidae	Culicinae	*Culex*	*Culex sp.*	1

2019	Setembro	02/09/2019	36	14	Samauma	P08	Culicidae	Culicinae	*Coquillettidia*	*Coquillettidia sp.*	3
2019	Setembro	02/09/2019	36	14	Samauma	P08	Culicidae	Culicinae	*Culex*	*Culex sp.*	830
2019	Setembro	02/09/2019	36	14	Samauma	P08	Culicidae	Culicinae	*Mansonia*	*Mansonia sp.*	600
2019	Setembro	02/09/2019	36	14	Jaci Paraná	P06	Culicidae	Culicinae	*Aedes*	*Aedes aegypti*	1
2019	Setembro	02/09/2019	36	14	Jaci Paraná	P06	Culicidae	Culicinae	*Culex*	*Culex sp.*	144
2019	Setembro	02/09/2019	36	14	Jaci Paraná	P06	Culicidae	Culicinae	*Mansonia*	*Mansonia sp.*	10
2019	Setembro	02/09/2019	36	14	Santa Rita	P05	Culicidae	Culicinae	*Culex*	*Culex sp.*	108
2019	Setembro	02/09/2019	36	14	Santa Rita	P05	Culicidae	Culicinae	*Mansonia*	*Mansonia sp.*	6
2019	Setembro	02/09/2019	36	14	Morrinhos	P04	Culicidae	Culicinae	*Aedeomyia*	*Adeomyia squamipennis*	4
2019	Setembro	02/09/2019	36	14	Morrinhos	P04	Culicidae	Culicinae	*Mansonia*	*Mansonia sp.*	57
2019	Setembro	02/09/2019	36	14	Teotônio	P03	Culicidae	Culicinae	*Aedeomyia*	*Adeomyia squamipennis*	2
2019	Setembro	02/09/2019	36	14	Teotônio	P03	Culicidae	Culicinae	*Culex*	*Culex sp.*	10
2019	Setembro	02/09/2019	36	14	Teotônio	P03	Culicidae	Culicinae	*Mansonia*	*Mansonia sp.*	5
2019	Setembro	02/09/2019	36	14	São Domingos	P02	Culicidae	Culicinae	*Aedeomyia*	*Adeomyia squamipennis*	52
2019	Setembro	02/09/2019	36	14	São Domingos	P02	Culicidae	Culicinae	*Coquillettidia*	*Coquillettidia sp.*	23
2019	Setembro	02/09/2019	36	14	São Domingos	P02	Culicidae	Culicinae	*Culex*	*Culex sp.*	520
2019	Setembro	02/09/2019	36	14	São Domingos	P02	Culicidae	Culicinae	*Mansonia*	*Mansonia sp.*	300
2019	Setembro	09/09/2019	37	15	Rio Contra	P07	Culicidae	Culicinae	*Mansonia*	*Mansonia sp.*	2
2019	Setembro	09/09/2019	37	15	Samauma	P08	Culicidae	Culicinae	*Aedeomyia*	*Adeomyia squamipennis*	6
2019	Setembro	09/09/2019	37	15	Samauma	P08	Culicidae	Culicinae	*Coquillettidia*	*Coquillettidia sp.*	3
2019	Setembro	09/09/2019	37	15	Samauma	P08	Culicidae	Culicinae	*Culex*	*Culex sp.*	1400
2019	Setembro	09/09/2019	37	15	Samauma	P08	Culicidae	Culicinae	*Mansonia*	*Mansonia sp.*	724
2019	Setembro	09/09/2019	37	15	Jaci Paraná	P06	Culicidae	Culicinae	*Aedes*	*Aedes sp.*	29
2019	Setembro	09/09/2019	37	15	Jaci Paraná	P06	Culicidae	Culicinae	*Culex*	*Culex sp.*	130
2019	Setembro	09/09/2019	37	15	Jaci Paraná	P06	Culicidae	Culicinae	*Mansonia*	*Mansonia sp.*	1
2019	Setembro	09/09/2019	37	15	Santa Rita	P05	Culicidae	Culicinae	*Culex*	*Culex sp.*	58
2019	Setembro	09/09/2019	37	15	Santa Rita	P05	Culicidae	Culicinae	*Mansonia*	*Mansonia sp.*	4
2019	Setembro	09/09/2019	37	15	Morrinhos	P04	Culicidae	Culicinae	*Culex*	*Culex sp.*	2
2019	Setembro	09/09/2019	37	15	Morrinhos	P04	Culicidae	Culicinae	*Mansonia*	*Mansonia sp.*	55
2019	Setembro	09/09/2019	37	15	Teotônio	P03	Culicidae	Culicinae	*Culex*	*Culex sp.*	4
2019	Setembro	09/09/2019	37	15	Teotônio	P03	Culicidae	Culicinae	*Mansonia*	*Mansonia sp.*	2
2019	Setembro	09/09/2019	37	15	São Domingos	P02	Culicidae	Culicinae	*Aedeomyia*	*Adeomyia squamipennis*	11
2019	Setembro	09/09/2019	37	15	São Domingos	P02	Culicidae	Culicinae	*Coquillettidia*	*Coquillettidia sp.*	78
2019	Setembro	09/09/2019	37	15	São Domingos	P02	Culicidae	Culicinae	*Culex*	*Culex sp.*	1040
2019	Setembro	09/09/2019	37	15	São Domingos	P02	Culicidae	Culicinae	*Mansonia*	*Mansonia sp.*	235
2019	Setembro	09/09/2019	37	15	Joana D'arc Line 15	P10	Culicidae	Culicinae	*Culex*	*Culex sp.*	13
2019	Setembro	09/09/2019	37	15	Joana D'arc Line 15	P10	Culicidae	Culicinae	*Mansonia*	*Mansonia sp.*	13
2019	Setembro	09/09/2019	37	15	Joana D'arc Line 09	P09	Culicidae	Culicinae	*Culex*	*Culex sp.*	6
2019	Setembro	09/09/2019	37	15	Joana D'arc Line 09	P09	Culicidae	Culicinae	*Mansonia*	*Mansonia sp.*	7
2019	Setembro	16/09/2019	38	16	Rio Contra	P07	Culicidae	Culicinae	*Mansonia*	*Mansonia sp.*	2
2019	Setembro	16/09/2019	38	16	Samauma	P08	Culicidae	Culicinae	*Coquillettidia*	*Coquillettidia sp.*	1
2019	Setembro	16/09/2019	38	16	Samauma	P08	Culicidae	Culicinae	*Culex*	*Culex sp.*	360
2019	Setembro	16/09/2019	38	16	Samauma	P08	Culicidae	Culicinae	*Mansonia*	*Mansonia sp.*	517
2019	Setembro	16/09/2019	38	16	Jaci Paraná	P06	Culicidae	Culicinae	*Aedes*	*Aedes sp.*	43
2019	Setembro	16/09/2019	38	16	Jaci Paraná	P06	Culicidae	Culicinae	*Coquillettidia*	*Coquillettidia sp.*	2
2019	Setembro	16/09/2019	38	16	Jaci Paraná	P06	Culicidae	Culicinae	*Culex*	*Culex sp.*	150
2019	Setembro	16/09/2019	38	16	Jaci Paraná	P06	Culicidae	Culicinae	*Mansonia*	*Mansonia sp.*	3
2019	Setembro	16/09/2019	38	16	Santa Rita	P05	Culicidae	Culicinae	*Culex*	*Culex sp.*	25
2019	Setembro	16/09/2019	38	16	Santa Rita	P05	Culicidae	Culicinae	*Mansonia*	*Mansonia sp.*	15
2019	Setembro	16/09/2019	38	16	Morrinhos	P04	Culicidae	Culicinae	*Mansonia*	*Mansonia sp.*	13
2019	Setembro	16/09/2019	38	16	Teotônio	P03	Culicidae	Culicinae	*Aedeomyia*	*Adeomyia squamipennis*	2
2019	Setembro	16/09/2019	38	16	Teotônio	P03	Culicidae	Culicinae	*Coquillettidia*	*Coquillettidia sp.*	9
2019	Setembro	16/09/2019	38	16	Teotônio	P03	Culicidae	Culicinae	*Mansonia*	*Mansonia sp.*	31
2019	Setembro	16/09/2019	38	16	São Domingos	P02	Culicidae	Culicinae	*Aedeomyia*	*Adeomyia squamipennis*	2
2019	Setembro	16/09/2019	38	16	São Domingos	P02	Culicidae	Culicinae	*Coquillettidia*	*Coquillettidia sp.*	4
2019	Setembro	16/09/2019	38	16	São Domingos	P02	Culicidae	Culicinae	*Culex*	*Culex sp.*	230
2019	Setembro	16/09/2019	38	16	São Domingos	P02	Culicidae	Culicinae	*Mansonia*	*Mansonia sp.*	72
2019	Setembro	16/09/2019	38	16	Cujubim Grande	P01	Culicidae	Culicinae	*Aedes*	*Aedes sp.*	1
2019	Setembro	16/09/2019	38	16	Cujubim Grande	P01	Culicidae	Culicinae	*Culex*	*Culex sp.*	266
2019	Setembro	16/09/2019	38	16	Cujubim Grande	P01	Culicidae	Culicinae	*Mansonia*	*Mansonia humeralis*	1
2019	Setembro	16/09/2019	38	16	Joana D'arc Line 15	P10	Culicidae	Culicinae	*Mansonia*	*Mansonia indubitans*	9
2019	Setembro	16/09/2019	38	16	Joana D'arc Line 09	P09	Culicidae	Culicinae	*Culex*	*Culex coronator*	1
2019	Setembro	16/09/2019	38	16	Joana D'arc Line 09	P09	Culicidae	Culicinae	*Mansonia*	*Mansonia sp.*	4
2019	Setembro	23/09/2019	39	17	Rio Contra	P07	Culicidae	Culicinae	*Mansonia*	*Mansonia sp.*	13
2019	Setembro	23/09/2019	39	17	Samauma	P08	Culicidae	Culicinae	*Culex*	*Culex sp.*	150
2019	Setembro	23/09/2019	39	17	Samauma	P08	Culicidae	Culicinae	*Mansonia*	*Mansonia sp.*	482
2019	Setembro	23/09/2019	39	17	Jaci Paraná	P06	Culicidae	Culicinae	*Culex*	*Culex sp.*	122
2019	Setembro	23/09/2019	39	17	Jaci Paraná	P06	Culicidae	Culicinae	*Mansonia*	*Mansonia sp.*	6
2019	Setembro	23/09/2019	39	17	Santa Rita	P05	Culicidae	Culicinae	*Culex*	*Culex sp.*	8
2019	Setembro	23/09/2019	39	17	Santa Rita	P05	Culicidae	Culicinae	*Mansonia*	*Mansonia sp.*	12
2019	Setembro	23/09/2019	39	17	Morrinhos	P04	Culicidae	Culicinae	*Mansonia*	*Mansonia sp.*	56
2019	Setembro	23/09/2019	39	17	Teotônio	P03	Culicidae	Culicinae	*Culex*	*Culex sp.*	4
2019	Setembro	23/09/2019	39	17	Teotônio	P03	Culicidae	Culicinae	*Mansonia*	*Mansonia sp.*	1
2019	Setembro	23/09/2019	39	17	São Domingos	P02	Culicidae	Culicinae	*Aedeomyia*	*Adeomyia squamipennis*	6
2019	Setembro	23/09/2019	39	17	São Domingos	P02	Culicidae	Culicinae	*Coquillettidia*	*Coquillettidia sp.*	7
2019	Setembro	23/09/2019	39	17	São Domingos	P02	Culicidae	Culicinae	*Culex*	*Culex sp.*	500
2019	Setembro	23/09/2019	39	17	São Domingos	P02	Culicidae	Culicinae	*Mansonia*	*Mansonia sp.*	168
2019	Setembro	23/09/2019	39	17	Cujubim Grande	P01	Culicidae	Culicinae	*Culex*	*Culex sp.*	156
2019	Setembro	23/09/2019	39	17	Cujubim Grande	P01	Culicidae	Culicinae	*Mansonia*	*Mansonia sp.*	5
2019	Setembro	23/09/2019	39	17	Joana D'arc Line 15	P10	Culicidae	Culicinae	*Culex*	*Culex sp.*	8
2019	Setembro	23/09/2019	39	17	Joana D'arc Line 15	P10	Culicidae	Culicinae	*Mansonia*	*Mansonia sp.*	15
2019	Setembro	23/09/2019	39	17	Joana D'arc Line 09	P09	Culicidae	Anophelinae	*Anopheles*	*Anopheles sp.*	1
2019	Setembro	23/09/2019	39	17	Joana D'arc Line 09	P09	Culicidae	Culicinae	*Culex*	*Culex sp.*	1
2019	Setembro	23/09/2019	39	17	Joana D'arc Line 09	P09	Culicidae	Culicinae	*Mansonia*	*Mansonia sp.*	6
2019	Setembro	30/09/2019	40	18	Rio Contra	P07	Culicidae	Culicinae	*Culex*	*Culex sp.*	1
2019	Setembro	30/09/2019	40	18	Rio Contra	P07	Culicidae	Culicinae	*Mansonia*	*Mansonia sp.*	2
2019	Setembro	30/09/2019	40	18	Rio Contra	P07	Culicidae	Culicinae	*Uranotaenia*	*Uranotaenia lowii*	1
2019	Setembro	30/09/2019	40	18	Samauma	P08	Culicidae	Culicinae	*Aedeomyia*	*Adeomyia squamipennis*	2
2019	Setembro	30/09/2019	40	18	Samauma	P08	Culicidae	Anophelinae	*Anopheles*	*Anopheles sp.*	2
2019	Setembro	30/09/2019	40	18	Samauma	P08	Culicidae	Culicinae	*Coquillettidia*	*Coquillettidia sp.*	7
2019	Setembro	30/09/2019	40	18	Samauma	P08	Culicidae	Culicinae	*Culex*	*Culex sp.*	1363
2019	Setembro	30/09/2019	40	18	Samauma	P08	Culicidae	Culicinae	*Mansonia*	*Mansonia sp.*	950
2019	Setembro	30/09/2019	40	18	Jaci Paraná	P06	Culicidae	Culicinae	*Aedeomyia*	*Adeomyia squamipennis*	1
2019	Setembro	30/09/2019	40	18	Jaci Paraná	P06	Culicidae	Culicinae	*Aedes*	*Aedes sp.*	25
2019	Setembro	30/09/2019	40	18	Jaci Paraná	P06	Culicidae	Culicinae	*Culex*	*Culex sp.*	178
2019	Setembro	30/09/2019	40	18	Jaci Paraná	P06	Culicidae	Culicinae	*Mansonia*	*Mansonia sp.*	4
2019	Setembro	30/09/2019	40	18	Santa Rita	P05	Culicidae	Culicinae	*Culex*	*Culex sp.*	1
2019	Setembro	30/09/2019	40	18	Santa Rita	P05	Culicidae	Culicinae	*Mansonia*	*Mansonia sp.*	5
2019	Setembro	30/09/2019	40	18	Morrinhos	P04	Culicidae	Culicinae	*Aedeomyia*	*Adeomyia squamipennis*	1
2019	Setembro	30/09/2019	40	18	Morrinhos	P04	Culicidae	Culicinae	*Culex*	*Culex sp.*	9
2019	Setembro	30/09/2019	40	18	Morrinhos	P04	Culicidae	Culicinae	*Mansonia*	*Mansonia sp.*	101
2019	Setembro	30/09/2019	40	18	Teotônio	P03	Culicidae	Culicinae	*Coquillettidia*	*Coquillettidia sp.*	4
2019	Setembro	30/09/2019	40	18	Teotônio	P03	Culicidae	Culicinae	*Culex*	*Culex sp.*	18
2019	Setembro	30/09/2019	40	18	Teotônio	P03	Culicidae	Culicinae	*Mansonia*	*Mansonia sp.*	11
2019	Setembro	30/09/2019	40	18	São Domingos	P02	Culicidae	Culicinae	*Aedeomyia*	*Adeomyia squamipennis*	5
2019	Setembro	30/09/2019	40	18	São Domingos	P02	Culicidae	Anophelinae	*Anopheles*	*Anopheles sp.*	7
2019	Setembro	30/09/2019	40	18	São Domingos	P02	Culicidae	Culicinae	*Coquillettidia*	*Coquillettidia sp.*	6
2019	Setembro	30/09/2019	40	18	São Domingos	P02	Culicidae	Culicinae	*Culex*	*Culex sp.*	556
2019	Setembro	30/09/2019	40	18	São Domingos	P02	Culicidae	Culicinae	*Mansonia*	*Mansonia sp.*	97
2019	Setembro	30/09/2019	40	18	Cujubim Grande	P01	Culicidae	Culicinae	*Culex*	*Culex sp.*	405
2019	Setembro	30/09/2019	40	18	Cujubim Grande	P01	Culicidae	Culicinae	*Mansonia*	*Mansonia sp.*	2
2019	Setembro	30/09/2019	40	18	Joana D'arc Line 15	P10	Culicidae	Culicinae	*Culex*	*Culex sp.*	23
2019	Setembro	30/09/2019	40	18	Joana D'arc Line 15	P10	Culicidae	Culicinae	*Mansonia*	*Mansonia sp.*	55
2019	Setembro	30/09/2019	40	18	Joana D'arc Line 09	P09	Culicidae	Culicinae	*Culex*	*Culex sp.*	5
2019	Setembro	30/09/2019	40	18	Joana D'arc Line 09	P09	Culicidae	Culicinae	*Mansonia*	*Mansonia sp.*	9
2019	Outubro	07/10/2019	41	19	Samauma	P08	Culicidae	Culicinae	*Coquillettidia*	*Coquillettidia sp.*	2
2019	Outubro	07/10/2019	41	19	Samauma	P08	Culicidae	Culicinae	*Culex*	*Culex sp.*	620
2019	Outubro	07/10/2019	41	19	Samauma	P08	Culicidae	Culicinae	*Mansonia*	*Mansonia sp.*	827

2019	Outubro	07/10/2019	41	19	Jaci Paraná	P06	Culicidae	Culicinae	*Aedes*	*Aedes sp.*	19
2019	Outubro	07/10/2019	41	19	Jaci Paraná	P06	Culicidae	Culicinae	*Culex*	*Culex sp.*	182
2019	Outubro	07/10/2019	41	19	Jaci Paraná	P06	Culicidae	Culicinae	*Mansonia*	*Mansonia sp.*	1
2019	Outubro	07/10/2019	41	19	Santa Rita	P05	Culicidae	Culicinae	*Culex*	*Culex sp.*	32
2019	Outubro	07/10/2019	41	19	Santa Rita	P05	Culicidae	Culicinae	*Mansonia*	*Mansonia sp.*	14
2019	Outubro	07/10/2019	41	19	Morrinhos	P04	Culicidae	Culicinae	*Culex*	*Culex sp.*	4
2019	Outubro	07/10/2019	41	19	Morrinhos	P04	Culicidae	Culicinae	*Mansonia*	*Mansonia sp.*	45
2019	Outubro	07/10/2019	41	19	Teotônio	P03	Culicidae	Culicinae	*Culex*	*Culex sp.*	4
2019	Outubro	07/10/2019	41	19	Teotônio	P03	Culicidae	Culicinae	*Mansonia*	*Mansonia sp.*	1
2019	Outubro	07/10/2019	41	19	São Domingos	P02	Culicidae	Culicinae	*Aedeomyia*	*Adeomyia squamipennis*	12
2019	Outubro	07/10/2019	41	19	São Domingos	P02	Culicidae	Culicinae	*Coquillettidia*	*Coquillettidia sp.*	21
2019	Outubro	07/10/2019	41	19	São Domingos	P02	Culicidae	Culicinae	*Culex*	*Culex sp.*	600
2019	Outubro	07/10/2019	41	19	São Domingos	P02	Culicidae	Culicinae	*Mansonia*	*Mansonia sp.*	132
2019	Outubro	07/10/2019	41	19	Cujubim Grande	P01	Culicidae	Culicinae	*Culex*	*Culex sp.*	390
2019	Outubro	07/10/2019	41	19	Cujubim Grande	P01	Culicidae	Culicinae	*Mansonia*	*Mansonia sp.*	7
2019	Outubro	07/10/2019	41	19	Joana D'arc Line 15	P10	Culicidae	Culicinae	*Culex*	*Culex sp.*	77
2019	Outubro	07/10/2019	41	19	Joana D'arc Line 15	P10	Culicidae	Culicinae	*Mansonia*	*Mansonia sp.*	70
2019	Outubro	07/10/2019	41	19	Joana D'arc Line 09	P09	Culicidae	Culicinae	*Culex*	*Culex sp.*	28
2019	Outubro	07/10/2019	41	19	Joana D'arc Line 09	P09	Culicidae	Culicinae	*Mansonia*	*Mansonia sp.*	15
2019	Outubro	14/10/2019	42	20	Rio Contra	P07	Culicidae	Culicinae	*Culex*	*Culex sp.*	1
2019	Outubro	14/10/2019	42	20	Samauma	P08	Culicidae	Culicinae	*Culex*	*Culex sp.*	200
2019	Outubro	14/10/2019	42	20	Samauma	P08	Culicidae	Culicinae	*Mansonia*	*Mansonia sp.*	633
2019	Outubro	14/10/2019	42	20	Jaci Paraná	P06	Culicidae	Culicinae	*Aedes*	*Aedes sp.*	6
2019	Outubro	14/10/2019	42	20	Jaci Paraná	P06	Culicidae	Culicinae	*Coquillettidia*	*Coquillettidia sp.*	1
2019	Outubro	14/10/2019	42	20	Jaci Paraná	P06	Culicidae	Culicinae	*Culex*	*Culex sp.*	105
2019	Outubro	14/10/2019	42	20	Jaci Paraná	P06	Culicidae	Culicinae	*Mansonia*	*Mansonia sp.*	2
2019	Outubro	14/10/2019	42	20	Santa Rita	P05	Culicidae	Culicinae	*Culex*	*Culex sp.*	60
2019	Outubro	14/10/2019	42	20	Morrinhos	P04	Culicidae	Culicinae	*Culex*	*Culex sp.*	1
2019	Outubro	14/10/2019	42	20	Morrinhos	P04	Culicidae	Culicinae	*Mansonia*	*Mansonia sp.*	2
2019	Outubro	14/10/2019	42	20	Teotônio	P03	Culicidae	Culicinae	*Culex*	*Culex sp.*	2
2019	Outubro	14/10/2019	42	20	São Domingos	P02	Culicidae	Culicinae	*Aedeomyia*	*Adeomyia squamipennis*	4
2019	Outubro	14/10/2019	42	20	São Domingos	P02	Culicidae	Culicinae	*Culex*	*Culex sp.*	620
2019	Outubro	14/10/2019	42	20	São Domingos	P02	Culicidae	Culicinae	*Mansonia*	*Mansonia sp.*	10
2019	Outubro	14/10/2019	42	20	Cujubim Grande	P01	Culicidae	Culicinae	*Aedes*	*Aedes sp.*	2
2019	Outubro	14/10/2019	42	20	Cujubim Grande	P01	Culicidae	Culicinae	*Culex*	*Culex sp.*	100
2019	Outubro	14/10/2019	42	20	Cujubim Grande	P01	Culicidae	Culicinae	*Mansonia*	*Mansonia titillans*	1
2019	Outubro	14/10/2019	42	20	Joana D'arc Line 15	P10	Culicidae	Culicinae	*Culex*	*Culex sp.*	21
2019	Outubro	14/10/2019	42	20	Joana D'arc Line 15	P10	Culicidae	Culicinae	*Mansonia*	*Mansonia sp.*	28
2019	Outubro	14/10/2019	42	20	Joana D'arc Line 09	P09	Culicidae	Culicinae	*Culex*	*Culex sp.*	6
2019	Outubro	14/10/2019	42	20	Joana D'arc Line 09	P09	Culicidae	Culicinae	*Mansonia*	*Mansonia sp.*	18
2019	Outubro	21/10/2019	43	21	Rio Contra	P07	Culicidae	Culicinae	*Mansonia*	*Mansonia sp.*	5
2019	Outubro	21/10/2019	43	21	Samauma	P08	Culicidae	Culicinae	*Coquillettidia*	*Coquillettidia sp.*	2
2019	Outubro	21/10/2019	43	21	Samauma	P08	Culicidae	Culicinae	*Culex*	*Culex sp.*	250
2019	Outubro	21/10/2019	43	21	Samauma	P08	Culicidae	Culicinae	*Mansonia*	*Mansonia sp.*	1443
2019	Outubro	21/10/2019	43	21	Jaci Paraná	P06	Culicidae	Culicinae	*Aedes*	*Aedes sp.*	15
2019	Outubro	21/10/2019	43	21	Jaci Paraná	P06	Culicidae	Culicinae	*Culex*	*Culex sp.*	167
2019	Outubro	21/10/2019	43	21	Jaci Paraná	P06	Culicidae	Culicinae	*Mansonia*	*Mansonia sp.*	13
2019	Outubro	21/10/2019	43	21	Santa Rita	P05	Culicidae	Culicinae	*Culex*	*Culex sp.*	519
2019	Outubro	21/10/2019	43	21	Santa Rita	P05	Culicidae	Culicinae	*Mansonia*	*Mansonia sp.*	17
2019	Outubro	21/10/2019	43	21	Morrinhos	P04	Culicidae	Culicinae	*Culex*	*Culex sp.*	6
2019	Outubro	21/10/2019	43	21	Morrinhos	P04	Culicidae	Culicinae	*Mansonia*	*Mansonia sp.*	35
2019	Outubro	21/10/2019	43	21	Teotônio	P03	Culicidae	Culicinae	*Culex*	*Culex sp.*	3
2019	Outubro	21/10/2019	43	21	Teotônio	P03	Culicidae	Culicinae	*Mansonia*	*Mansonia sp.*	1
2019	Outubro	21/10/2019	43	21	São Domingos	P02	Culicidae	Culicinae	*Coquillettidia*	*Coquillettidia sp.*	12
2019	Outubro	21/10/2019	43	21	São Domingos	P02	Culicidae	Culicinae	*Culex*	*Culex sp.*	300
2019	Outubro	21/10/2019	43	21	São Domingos	P02	Culicidae	Culicinae	*Mansonia*	*Mansonia sp.*	20
2019	Outubro	21/10/2019	43	21	Cujubim Grande	P01	Culicidae	Culicinae	*Culex*	*Culex sp.*	100
2019	Outubro	21/10/2019	43	21	Cujubim Grande	P01	Culicidae	Culicinae	*Mansonia*	*Mansonia sp.*	6
2019	Outubro	21/10/2019	43	21	Joana D'arc Line 15	P10	Culicidae	Culicinae	*Culex*	*Culex sp.*	9
2019	Outubro	21/10/2019	43	21	Joana D'arc Line 15	P10	Culicidae	Culicinae	*Mansonia*	*Mansonia sp.*	22
2019	Outubro	21/10/2019	43	21	Joana D'arc Line 09	P09	Culicidae	Culicinae	*Culex*	*Culex sp.*	2
2019	Outubro	21/10/2019	43	21	Joana D'arc Line 09	P09	Culicidae	Culicinae	*Mansonia*	*Mansonia sp.*	9
2019	Outubro	28/10/2019	44	22	Rio Contra	P07	Culicidae	Culicinae	*Culex*	*Culex sp.*	15
2019	Outubro	28/10/2019	44	22	Rio Contra	P07	Culicidae	Culicinae	*Mansonia*	*Mansonia sp.*	30
2019	Outubro	28/10/2019	44	22	Samauma	P08	Culicidae	Culicinae	*Culex*	*Culex sp.*	56
2019	Outubro	28/10/2019	44	22	Samauma	P08	Culicidae	Culicinae	*Mansonia*	*Mansonia sp.*	133
2019	Outubro	28/10/2019	44	22	Jaci Paraná	P06	Culicidae	Culicinae	*Aedes*	*Aedes sp.*	10
2019	Outubro	28/10/2019	44	22	Jaci Paraná	P06	Culicidae	Culicinae	*Culex*	*Culex sp.*	113
2019	Outubro	28/10/2019	44	22	Jaci Paraná	P06	Culicidae	Culicinae	*Mansonia*	*Mansonia sp.*	3
2019	Outubro	28/10/2019	44	22	Santa Rita	P05	Culicidae	Culicinae	*Culex*	*Culex sp.*	45
2019	Outubro	28/10/2019	44	22	Santa Rita	P05	Culicidae	Culicinae	*Mansonia*	*Mansonia sp.*	38
2019	Outubro	28/10/2019	44	22	Morrinhos	P04	Culicidae	Culicinae	*Mansonia*	*Mansonia sp.*	79
2019	Outubro	28/10/2019	44	22	Teotônio	P03	Culicidae	Culicinae	*Coquillettidia*	*Coquillettidia sp.*	1
2019	Outubro	28/10/2019	44	22	Teotônio	P03	Culicidae	Culicinae	*Culex*	*Culex sp.*	2
2019	Outubro	28/10/2019	44	22	São Domingos	P02	Culicidae	Anophelinae	*Anopheles*	*Anopheles darlingi*	1
2019	Outubro	28/10/2019	44	22	São Domingos	P02	Culicidae	Culicinae	*Coquillettidia*	*Coquillettidia sp.*	3
2019	Outubro	28/10/2019	44	22	São Domingos	P02	Culicidae	Culicinae	*Culex*	*Culex sp.*	170
2019	Outubro	28/10/2019	44	22	São Domingos	P02	Culicidae	Culicinae	*Mansonia*	*Mansonia sp.*	48
2019	Outubro	28/10/2019	44	22	Cujubim Grande	P01	Culicidae	Culicinae	*Culex*	*Culex sp.*	70
2019	Outubro	28/10/2019	44	22	Cujubim Grande	P01	Culicidae	Culicinae	*Mansonia*	*Mansonia sp.*	2
2019	Outubro	28/10/2019	44	22	Joana D'arc Line 15	P10	Culicidae	Culicinae	*Culex*	*Culex sp.*	17
2019	Outubro	28/10/2019	44	22	Joana D'arc Line 15	P10	Culicidae	Culicinae	*Mansonia*	*Mansonia sp.*	41
2019	Outubro	28/10/2019	44	22	Joana D'arc Line 09	P09	Culicidae	Culicinae	*Culex*	*Culex sp.*	9
2019	Outubro	28/10/2019	44	22	Joana D'arc Line 09	P09	Culicidae	Culicinae	*Mansonia*	*Mansonia sp.*	12
2019	Novembro	04/11/2019	45	23	Rio Contra	P07	Culicidae	Culicinae	*Mansonia*	*Mansonia sp.*	7
2019	Novembro	04/11/2019	45	23	Samauma	P08	Culicidae	Culicinae	*Aedes*	*Aedes sp.*	3
2019	Novembro	04/11/2019	45	23	Samauma	P08	Culicidae	Culicinae	*Culex*	*Culex sp.*	117
2019	Novembro	04/11/2019	45	23	Samauma	P08	Culicidae	Culicinae	*Mansonia*	*Mansonia sp.*	868
2019	Novembro	04/11/2019	45	23	Jaci Paraná	P06	Culicidae	Culicinae	*Culex*	*Culex sp.*	185
2019	Novembro	04/11/2019	45	23	Jaci Paraná	P06	Culicidae	Culicinae	*Mansonia*	*Mansonia sp.*	11
2019	Novembro	04/11/2019	45	23	Santa Rita	P05	Culicidae	Anophelinae	*Anopheles*	*Anopheles darlingi*	1
2019	Novembro	04/11/2019	45	23	Santa Rita	P05	Culicidae	Culicinae	*Culex*	*Culex sp.*	536
2019	Novembro	04/11/2019	45	23	Santa Rita	P05	Culicidae	Culicinae	*Mansonia*	*Mansonia sp.*	9
2019	Novembro	04/11/2019	45	23	Morrinhos	P04	Culicidae	Culicinae	*Culex*	*Culex sp.*	10
2019	Novembro	04/11/2019	45	23	Morrinhos	P04	Culicidae	Culicinae	*Mansonia*	*Mansonia sp.*	34
2019	Novembro	04/11/2019	45	23	Teotônio	P03	Culicidae	Culicinae	*Coquillettidia*	*Coquillettidia sp.*	6
2019	Novembro	04/11/2019	45	23	Teotônio	P03	Culicidae	Culicinae	*Culex*	*Culex sp.*	120
2019	Novembro	04/11/2019	45	23	Teotônio	P03	Culicidae	Culicinae	*Mansonia*	*Mansonia sp.*	10
2019	Novembro	04/11/2019	45	23	São Domingos	P02	Culicidae	Culicinae	*Coquillettidia*	*Coquillettidia sp.*	14
2019	Novembro	04/11/2019	45	23	São Domingos	P02	Culicidae	Culicinae	*Culex*	*Culex sp.*	450
2019	Novembro	04/11/2019	45	23	São Domingos	P02	Culicidae	Culicinae	*Mansonia*	*Mansonia sp.*	80
2019	Novembro	04/11/2019	45	23	Cujubim Grande	P01	Culicidae	Culicinae	*Culex*	*Culex sp.*	34
2019	Novembro	04/11/2019	45	23	Cujubim Grande	P01	Culicidae	Culicinae	*Mansonia*	*Mansonia sp.*	4
2019	Novembro	04/11/2019	45	23	Joana D'arc Line 15	P10	Culicidae	Culicinae	*Mansonia*	*Mansonia sp.*	28
2019	Novembro	04/11/2019	45	23	Joana D'arc Line 09	P09	Culicidae	Culicinae	*Mansonia*	*Mansonia sp.*	11
2019	Novembro	11/11/2019	46	24	Rio Contra	P07	Culicidae	Culicinae	*Aedes*	*Aedes sp.*	2
2019	Novembro	11/11/2019	46	24	Rio Contra	P07	Culicidae	Anophelinae	*Anopheles*	*Anopheles darlingi*	1
2019	Novembro	11/11/2019	46	24	Rio Contra	P07	Culicidae	Culicinae	*Culex*	*Culex sp.*	3
2019	Novembro	11/11/2019	46	24	Rio Contra	P07	Culicidae	Culicinae	*Mansonia*	*Mansonia sp.*	46
2019	Novembro	11/11/2019	46	24	Samauma	P08	Culicidae	Culicinae	*Aedeomyia*	*Adeomyia squamipennis*	7
2019	Novembro	11/11/2019	46	24	Samauma	P08	Culicidae	Culicinae	*Culex*	*Culex sp.*	269
2019	Novembro	11/11/2019	46	24	Samauma	P08	Culicidae	Culicinae	*Mansonia*	*Mansonia sp.*	2083
2019	Novembro	11/11/2019	46	24	Jaci Paraná	P06	Culicidae	Culicinae	*Aedes*	*Aedes sp.*	33
2019	Novembro	11/11/2019	46	24	Jaci Paraná	P06	Culicidae	Culicinae	*Coquillettidia*	*Coquillettidia sp.*	1
2019	Novembro	11/11/2019	46	24	Jaci Paraná	P06	Culicidae	Culicinae	*Culex*	*Culex sp.*	73
2019	Novembro	11/11/2019	46	24	Jaci Paraná	P06	Culicidae	Culicinae	*Mansonia*	*Mansonia sp.*	10
2019	Novembro	11/11/2019	46	24	Santa Rita	P05	Culicidae	Culicinae	*Coquillettidia*	*Coquillettidia sp.*	2
2019	Novembro	11/11/2019	46	24	Santa Rita	P05	Culicidae	Culicinae	*Culex*	*Culex sp.*	330

2019	Novembro	11/11/2019	46	24	Santa Rita	P05	Culicidae	Culicinae	*Mansonia*	*Mansonia sp.*	6
2019	Novembro	11/11/2019	46	24	Morrinhos	P04	Culicidae	Culicinae	*Mansonia*	*Mansonia sp.*	24
2019	Novembro	11/11/2019	46	24	Teotônio	P03	Culicidae	Culicinae	*Coquillettidia*	*Coquillettidia sp.*	1
2019	Novembro	11/11/2019	46	24	Teotônio	P03	Culicidae	Culicinae	*Culex*	*Culex sp.*	13
2019	Novembro	11/11/2019	46	24	São Domingos	P02	Culicidae	Culicinae	*Coquillettidia*	*Coquillettidia sp.*	12
2019	Novembro	11/11/2019	46	24	São Domingos	P02	Culicidae	Culicinae	*Culex*	*Culex sp.*	9
2019	Novembro	11/11/2019	46	24	São Domingos	P02	Culicidae	Culicinae	*Mansonia*	*Mansonia sp.*	23
2019	Novembro	11/11/2019	46	24	Cujubim Grande	P01	Culicidae	Culicinae	*Culex*	*Culex sp.*	50
2019	Novembro	11/11/2019	46	24	Cujubim Grande	P01	Culicidae	Culicinae	*Mansonia*	*Mansonia sp.*	5
2019	Novembro	11/11/2019	46	24	Joana D'arc Line 15	P10	Culicidae	Culicinae	*Mansonia*	*Mansonia sp.*	9
2019	Novembro	11/11/2019	46	24	Joana D'arc Line 09	P09	Culicidae	Culicinae	*Mansonia*	*Mansonia sp.*	6
2019	Novembro	18/11/2019	47	25	Rio Contra	P07	Culicidae	Culicinae	*Aedeomyia*	*Adeomyia squamipennis*	1
2019	Novembro	18/11/2019	47	25	Rio Contra	P07	Culicidae	Culicinae	*Culex*	*Culex sp.*	7
2019	Novembro	18/11/2019	47	25	Rio Contra	P07	Culicidae	Culicinae	*Mansonia*	*Mansonia sp.*	8
2019	Novembro	18/11/2019	47	25	Samauma	P08	Culicidae	Culicinae	*Coquillettidia*	*Coquillettidia sp.*	1
2019	Novembro	18/11/2019	47	25	Samauma	P08	Culicidae	Culicinae	*Culex*	*Culex sp.*	206
2019	Novembro	18/11/2019	47	25	Samauma	P08	Culicidae	Culicinae	*Mansonia*	*Mansonia sp.*	316
2019	Novembro	18/11/2019	47	25	Jaci Paraná	P06	Culicidae	Culicinae	*Aedes*	*Aedes sp.*	40
2019	Novembro	18/11/2019	47	25	Jaci Paraná	P06	Culicidae	Culicinae	*Coquillettidia*	*Coquillettidia venezuelensis*	1
2019	Novembro	18/11/2019	47	25	Jaci Paraná	P06	Culicidae	Culicinae	*Culex*	*Culex sp.*	68
2019	Novembro	18/11/2019	47	25	Jaci Paraná	P06	Culicidae	Culicinae	*Mansonia*	*Mansonia sp.*	18
2019	Novembro	18/11/2019	47	25	Santa Rita	P05	Culicidae	Culicinae	*Aedeomyia*	*Adeomyia squamipennis*	1
2019	Novembro	18/11/2019	47	25	Santa Rita	P05	Culicidae	Culicinae	*Aedes*	*Aedes sp.*	2
2019	Novembro	18/11/2019	47	25	Santa Rita	P05	Culicidae	Anophelinae	*Anopheles*	*Anopheles sp.*	1
2019	Novembro	18/11/2019	47	25	Santa Rita	P05	Culicidae	Culicinae	*Coquillettidia*	*Coquillettidia sp.*	5
2019	Novembro	18/11/2019	47	25	Santa Rita	P05	Culicidae	Culicinae	*Culex*	*Culex sp.*	692
2019	Novembro	18/11/2019	47	25	Santa Rita	P05	Culicidae	Culicinae	*Mansonia*	*Mansonia sp.*	15
2019	Novembro	18/11/2019	47	25	Morrinhos	P04	Culicidae	Culicinae	*Culex*	*Culex sp.*	11
2019	Novembro	18/11/2019	47	25	Morrinhos	P04	Culicidae	Culicinae	*Mansonia*	*Mansonia sp.*	20
2019	Novembro	18/11/2019	47	25	Teotônio	P03	Culicidae	Culicinae	*Coquillettidia*	*Coquillettidia sp.*	1
2019	Novembro	18/11/2019	47	25	Teotônio	P03	Culicidae	Culicinae	*Culex*	*Culex sp.*	7
2019	Novembro	18/11/2019	47	25	Teotônio	P03	Culicidae	Culicinae	*Mansonia*	*Mansonia sp.*	4
2019	Novembro	18/11/2019	47	25	São Domingos	P02	Culicidae	Culicinae	*Aedes*	*Aedes sp.*	1
2019	Novembro	18/11/2019	47	25	São Domingos	P02	Culicidae	Culicinae	*Coquillettidia*	*Coquillettidia sp.*	1
2019	Novembro	18/11/2019	47	25	São Domingos	P02	Culicidae	Culicinae	*Mansonia*	*Mansonia sp.*	3
2019	Novembro	18/11/2019	47	25	Cujubim Grande	P01	Culicidae	Culicinae	*Aedes*	*Aedes sp.*	1
2019	Novembro	18/11/2019	47	25	Cujubim Grande	P01	Culicidae	Culicinae	*Culex*	*Culex sp.*	14
2019	Novembro	18/11/2019	47	25	Cujubim Grande	P01	Culicidae	Culicinae	*Mansonia*	*Mansonia sp.*	2
2019	Novembro	18/11/2019	47	25	Joana D'arc Line 15	P10	Culicidae	Culicinae	*Culex*	*Culex sp.*	5
2019	Novembro	18/11/2019	47	25	Joana D'arc Line 15	P10	Culicidae	Culicinae	*Mansonia*	*Mansonia sp.*	6
2019	Novembro	18/11/2019	47	25	Joana D'arc Line 09	P09	Culicidae	Culicinae	*Culex*	*Culex sp.*	11
2019	Novembro	18/11/2019	47	25	Joana D'arc Line 09	P09	Culicidae	Culicinae	*Mansonia*	*Mansonia sp.*	7
2019	Novembro	25/11/2019	48	26	Rio Contra	P07	Culicidae	Culicinae	*Culex*	*Culex sp.*	1
2019	Novembro	25/11/2019	48	26	Rio Contra	P07	Culicidae	Culicinae	*Mansonia*	*Mansonia sp.*	6
2019	Novembro	25/11/2019	48	26	Samauma	P08	Culicidae	Culicinae	*Aedeomyia*	*Adeomyia squamipennis*	1
2019	Novembro	25/11/2019	48	26	Samauma	P08	Culicidae	Culicinae	*Aedes*	*Aedes sp.*	2
2019	Novembro	25/11/2019	48	26	Samauma	P08	Culicidae	Anophelinae	*Anopheles*	*Anopheles sp.*	1
2019	Novembro	25/11/2019	48	26	Samauma	P08	Culicidae	Culicinae	*Coquillettidia*	*Coquillettidia sp.*	2
2019	Novembro	25/11/2019	48	26	Samauma	P08	Culicidae	Culicinae	*Culex*	*Culex sp.*	310
2019	Novembro	25/11/2019	48	26	Samauma	P08	Culicidae	Culicinae	*Mansonia*	*Mansonia sp.*	368
2019	Novembro	25/11/2019	48	26	Samauma	P08	Culicidae	Culicinae	*Uranotaenia*	*Uranotaenia sp.*	3
2019	Novembro	25/11/2019	48	26	Jaci Paraná	P06	Culicidae	Culicinae	*Aedes*	*Aedes aegypti*	9
2019	Novembro	25/11/2019	48	26	Jaci Paraná	P06	Culicidae	Culicinae	*Aedes*	*Aedes sp.*	2
2019	Novembro	25/11/2019	48	26	Jaci Paraná	P06	Culicidae	Culicinae	*Culex*	*Culex sp.*	84
2019	Novembro	25/11/2019	48	26	Jaci Paraná	P06	Culicidae	Culicinae	*Mansonia*	*Mansonia sp.*	11
2019	Novembro	25/11/2019	48	26	Santa Rita	P05	Culicidae	Culicinae	*Aedes*	*Aedes sp.*	1
2019	Novembro	25/11/2019	48	26	Santa Rita	P05	Culicidae	Culicinae	*Coquillettidia*	*Coquillettidia sp.*	1
2019	Novembro	25/11/2019	48	26	Santa Rita	P05	Culicidae	Culicinae	*Culex*	*Culex sp.*	101
2019	Novembro	25/11/2019	48	26	Santa Rita	P05	Culicidae	Culicinae	*Mansonia*	*Mansonia sp.*	25
2019	Novembro	25/11/2019	48	26	Morrinhos	P04	Culicidae	Culicinae	*Aedes*	*Aedes sp.*	8
2019	Novembro	25/11/2019	48	26	Morrinhos	P04	Culicidae	Culicinae	*Culex*	*Culex sp.*	13
2019	Novembro	25/11/2019	48	26	Morrinhos	P04	Culicidae	Culicinae	*Mansonia*	*Mansonia sp.*	36
2019	Novembro	25/11/2019	48	26	São Domingos	P02	Culicidae	Culicinae	*Coquillettidia*	*Coquillettidia sp.*	3
2019	Novembro	25/11/2019	48	26	São Domingos	P02	Culicidae	Culicinae	*Culex*	*Culex sp.*	47
2019	Novembro	25/11/2019	48	26	São Domingos	P02	Culicidae	Culicinae	*Mansonia*	*Mansonia sp.*	36
2019	Novembro	25/11/2019	48	26	Cujubim Grande	P01	Culicidae	Culicinae	*Culex*	*Culex sp.*	3
2019	Novembro	25/11/2019	48	26	Cujubim Grande	P01	Culicidae	Culicinae	*Mansonia*	*Mansonia sp.*	1
2019	Novembro	25/11/2019	48	26	Cujubim Grande	P01	Culicidae	Culicinae	*Uranotaenia*	*Uranotaenia sp.*	1
2019	Novembro	25/11/2019	48	26	Joana D'arc Line 15	P10	Culicidae	Culicinae	*Culex*	*Culex sp.*	17
2019	Novembro	25/11/2019	48	26	Joana D'arc Line 15	P10	Culicidae	Culicinae	*Mansonia*	*Mansonia sp.*	22
2019	Novembro	25/11/2019	48	26	Joana D'arc Line 09	P09	Culicidae	Culicinae	*Culex*	*Culex sp.*	13
2019	Novembro	25/11/2019	48	26	Joana D'arc Line 09	P09	Culicidae	Culicinae	*Mansonia*	*Mansonia sp.*	18
2019	Dezembro	02/12/2019	49	27	Rio Contra	P07	Culicidae	Anophelinae	*Anopheles*	*Anopheles sp.*	1
2019	Dezembro	02/12/2019	49	27	Rio Contra	P07	Culicidae	Culicinae	*Culex*	*Culex sp.*	6
2019	Dezembro	02/12/2019	49	27	Rio Contra	P07	Culicidae	Culicinae	*Mansonia*	*Mansonia sp.*	4
2019	Dezembro	02/12/2019	49	27	Samauma	P08	Culicidae	Culicinae	*Aedeomyia*	*Adeomyia squamipennis*	10
2019	Dezembro	02/12/2019	49	27	Samauma	P08	Culicidae	Culicinae	*Aedes*	*Aedes sp.*	3
2019	Dezembro	02/12/2019	49	27	Samauma	P08	Culicidae	Culicinae	*Coquillettidia*	*Coquillettidia sp.*	4
2019	Dezembro	02/12/2019	49	27	Samauma	P08	Culicidae	Culicinae	*Culex*	*Culex sp.*	178
2019	Dezembro	02/12/2019	49	27	Samauma	P08	Culicidae	Culicinae	*Mansonia*	*Mansonia sp.*	853
2019	Dezembro	02/12/2019	49	27	Samauma	P08	Culicidae	Culicinae	*Psorophora*	*Psorophora sp.*	3
2019	Dezembro	02/12/2019	49	27	Jaci Paraná	P06	Culicidae	Culicinae	*Aedes*	*Aedes aegypti*	42
2019	Dezembro	02/12/2019	49	27	Jaci Paraná	P06	Culicidae	Culicinae	*Culex*	*Culex sp.*	38
2019	Dezembro	02/12/2019	49	27	Jaci Paraná	P06	Culicidae	Culicinae	*Mansonia*	*Mansonia sp.*	19
2019	Dezembro	02/12/2019	49	27	Santa Rita	P05	Culicidae	Culicinae	*Coquillettidia*	*Coquillettidia sp.*	2
2019	Dezembro	02/12/2019	49	27	Santa Rita	P05	Culicidae	Culicinae	*Culex*	*Culex sp.*	273
2019	Dezembro	02/12/2019	49	27	Santa Rita	P05	Culicidae	Culicinae	*Mansonia*	*Mansonia sp.*	22
2019	Dezembro	02/12/2019	49	27	Morrinhos	P04	Culicidae	Culicinae	*Culex*	*Culex sp.*	17
2019	Dezembro	02/12/2019	49	27	Morrinhos	P04	Culicidae	Culicinae	*Mansonia*	*Mansonia sp.*	69
2019	Dezembro	02/12/2019	49	27	Teotônio	P03	Culicidae	Culicinae	*Culex*	*Culex sp.*	16
2019	Dezembro	02/12/2019	49	27	São Domingos	P02	Culicidae	Culicinae	*Coquillettidia*	*Coquillettidia sp.*	10
2019	Dezembro	02/12/2019	49	27	São Domingos	P02	Culicidae	Culicinae	*Culex*	*Culex sp.*	137
2019	Dezembro	02/12/2019	49	27	São Domingos	P02	Culicidae	Culicinae	*Mansonia*	*Mansonia sp.*	122
2019	Dezembro	02/12/2019	49	27	Cujubim Grande	P01	Culicidae	Anophelinae	*Anopheles*	*Anopheles sp.*	1
2019	Dezembro	02/12/2019	49	27	Cujubim Grande	P01	Culicidae	Culicinae	*Culex*	*Culex sp.*	82
2019	Dezembro	02/12/2019	49	27	Cujubim Grande	P01	Culicidae	Culicinae	*Mansonia*	*Mansonia sp.*	2
2019	Dezembro	02/12/2019	49	27	Joana D'arc Line 15	P10	Culicidae	Culicinae	*Culex*	*Culex sp.*	17
2019	Dezembro	02/12/2019	49	27	Joana D'arc Line 15	P10	Culicidae	Culicinae	*Mansonia*	*Mansonia sp.*	39
2019	Dezembro	02/12/2019	49	27	Joana D'arc Line 09	P09	Culicidae	Culicinae	*Mansonia*	*Mansonia titillans*	3
2019	Dezembro	09/12/2019	50	28	Rio Contra	P07	Culicidae	Culicinae	*Culex*	*Culex sp.*	5
2019	Dezembro	09/12/2019	50	28	Rio Contra	P07	Culicidae	Culicinae	*Mansonia*	*Mansonia sp.*	3
2019	Dezembro	09/12/2019	50	28	Samauma	P08	Culicidae	Culicinae	*Culex*	*Culex sp.*	270
2019	Dezembro	09/12/2019	50	28	Samauma	P08	Culicidae	Culicinae	*Mansonia*	*Mansonia sp.*	381
2019	Dezembro	09/12/2019	50	28	Jaci Paraná	P06	Culicidae	Culicinae	*Aedes*	*Aedes sp.*	18
2019	Dezembro	09/12/2019	50	28	Jaci Paraná	P06	Culicidae	Culicinae	*Culex*	*Culex sp.*	53
2019	Dezembro	09/12/2019	50	28	Jaci Paraná	P06	Culicidae	Culicinae	*Mansonia*	*Mansonia sp.*	10
2019	Dezembro	09/12/2019	50	28	Santa Rita	P05	Culicidae	Culicinae	*Culex*	*Culex sp.*	100
2019	Dezembro	09/12/2019	50	28	Santa Rita	P05	Culicidae	Culicinae	*Mansonia*	*Mansonia sp.*	19
2019	Dezembro	09/12/2019	50	28	Morrinhos	P04	Culicidae	Culicinae	*Culex*	*Culex sp.*	3
2019	Dezembro	09/12/2019	50	28	Morrinhos	P04	Culicidae	Culicinae	*Mansonia*	*Mansonia sp.*	15
2019	Dezembro	09/12/2019	50	28	Teotônio	P03	Culicidae	Culicinae	*Coquillettidia*	*Coquillettidia sp.*	28
2019	Dezembro	09/12/2019	50	28	Teotônio	P03	Culicidae	Culicinae	*Culex*	*Culex sp.*	57
2019	Dezembro	09/12/2019	50	28	Teotônio	P03	Culicidae	Culicinae	*Mansonia*	*Mansonia sp.*	13
2019	Dezembro	09/12/2019	50	28	São Domingos	P02	Culicidae	Culicinae	*Culex*	*Culex sp.*	7
2019	Dezembro	09/12/2019	50	28	São Domingos	P02	Culicidae	Culicinae	*Mansonia*	*Mansonia sp.*	2
2019	Dezembro	09/12/2019	50	28	Cujubim Grande	P01	Culicidae	Culicinae	*Culex*	*Culex sp.*	45
2019	Dezembro	09/12/2019	50	28	Cujubim Grande	P01	Culicidae	Culicinae	*Mansonia*	*Mansonia sp.*	1
2019	Dezembro	09/12/2019	50	28	Joana D'arc Line 15	P10	Culicidae	Culicinae	*Culex*	*Culex sp.*	7

2019	Dezembro	09/12/2019	50	28	Joana D'arc Line 15	P10	Culicidae	Culicinae	*Mansonia*	*Mansonia sp.*	31
2019	Dezembro	09/12/2019	50	28	Joana D'arc Line 09	P09	Culicidae	Culicinae	*Culex*	*Culex sp.*	5
2019	Dezembro	09/12/2019	50	28	Joana D'arc Line 09	P09	Culicidae	Culicinae	*Mansonia*	*Mansonia sp.*	5
2019	Dezembro	16/12/2019	51	29	Rio Contra	P07	Culicidae	Culicinae	*Mansonia*	*Mansonia sp.*	2
2019	Dezembro	16/12/2019	51	29	Samauma	P08	Culicidae	Culicinae	*Aedeomyia*	*Adeomyia squamipennis*	3
2019	Dezembro	16/12/2019	51	29	Samauma	P08	Culicidae	Culicinae	*Culex*	*Culex sp.*	200
2019	Dezembro	16/12/2019	51	29	Samauma	P08	Culicidae	Culicinae	*Mansonia*	*Mansonia sp.*	365
2019	Dezembro	16/12/2019	51	29	Jaci Paraná	P06	Culicidae	Culicinae	*Aedeomyia*	*Adeomyia squamipennis*	2
2019	Dezembro	16/12/2019	51	29	Jaci Paraná	P06	Culicidae	Culicinae	*Aedes*	*Aedes sp.*	14
2019	Dezembro	16/12/2019	51	29	Jaci Paraná	P06	Culicidae	Culicinae	*Culex*	*Culex sp.*	40
2019	Dezembro	16/12/2019	51	29	Jaci Paraná	P06	Culicidae	Culicinae	*Mansonia*	*Mansonia sp.*	20
2019	Dezembro	16/12/2019	51	29	Santa Rita	P05	Culicidae	Culicinae	*Culex*	*Culex sp.*	40
2019	Dezembro	16/12/2019	51	29	Santa Rita	P05	Culicidae	Culicinae	*Mansonia*	*Mansonia humeralis*	2
2019	Dezembro	16/12/2019	51	29	Morrinhos	P04	Culicidae	Culicinae	*Coquillettidia*	*Coquillettidia sp.*	1
2019	Dezembro	16/12/2019	51	29	Morrinhos	P04	Culicidae	Culicinae	*Mansonia*	*Mansonia sp.*	10
2019	Dezembro	16/12/2019	51	29	Teotônio	P03	Culicidae	Culicinae	*Culex*	*Culex sp.*	3
2019	Dezembro	16/12/2019	51	29	Teotônio	P03	Culicidae	Culicinae	*Culex*	*Culex sp.*	9
2019	Dezembro	16/12/2019	51	29	Teotônio	P03	Culicidae	Culicinae	*Mansonia*	*Mansonia sp.*	1
2019	Dezembro	16/12/2019	51	29	São Domingos	P02	Culicidae	Culicinae	*Mansonia*	*Mansonia amazonensis*	7
2019	Dezembro	16/12/2019	51	29	Cujubim Grande	P01	Culicidae	Culicinae	*Culex*	*Culex sp.*	70
2019	Dezembro	16/12/2019	51	29	Cujubim Grande	P01	Culicidae	Culicinae	*Mansonia*	*Mansonia sp.*	18
2019	Dezembro	16/12/2019	51	29	Joana D'arc Line 15	P10	Culicidae	Culicinae	*Culex*	*Culex sp.*	23
2019	Dezembro	16/12/2019	51	29	Joana D'arc Line 15	P10	Culicidae	Culicinae	*Mansonia*	*Mansonia sp.*	33
2019	Dezembro	16/12/2019	51	29	Joana D'arc Line 09	P09	Culicidae	Culicinae	*Mansonia*	*Mansonia sp.*	21
2019	Dezembro	23/12/2019	52	30	Rio Contra	P07	Culicidae	Culicinae	*Aedes*	*Aedes serratus*	1
2019	Dezembro	23/12/2019	52	30	Rio Contra	P07	Culicidae	Culicinae	*Culex*	*Culex sp.*	8
2019	Dezembro	23/12/2019	52	30	Rio Contra	P07	Culicidae	Culicinae	*Mansonia*	*Mansonia sp.*	2
2019	Dezembro	23/12/2019	52	30	Samauma	P08	Culicidae	Culicinae	*Culex*	*Culex sp.*	200
2019	Dezembro	23/12/2019	52	30	Samauma	P08	Culicidae	Culicinae	*Mansonia*	*Mansonia sp.*	500
2019	Dezembro	23/12/2019	52	30	Jaci Paraná	P06	Culicidae	Culicinae	*Aedes*	*Aedes sp.*	21
2019	Dezembro	23/12/2019	52	30	Jaci Paraná	P06	Culicidae	Culicinae	*Mansonia*	*Mansonia sp.*	25
2019	Dezembro	23/12/2019	52	30	Santa Rita	P05	Culicidae	Culicinae	*Culex*	*Culex sp.*	61
2019	Dezembro	23/12/2019	52	30	Santa Rita	P05	Culicidae	Culicinae	*Mansonia*	*Mansonia sp.*	4
2019	Dezembro	23/12/2019	52	30	Morrinhos	P04	Culicidae	Culicinae	*Aedes*	*Aedes sp.*	49
2019	Dezembro	23/12/2019	52	30	Morrinhos	P04	Culicidae	Culicinae	*Culex*	*Culex sp.*	34
2019	Dezembro	23/12/2019	52	30	Morrinhos	P04	Culicidae	Culicinae	*Mansonia*	*Mansonia sp.*	200
2019	Dezembro	23/12/2019	52	30	Teotônio	P03	Culicidae	Culicinae	*Aedes*	*Aedes sp.*	1
2019	Dezembro	23/12/2019	52	30	Teotônio	P03	Culicidae	Culicinae	*Culex*	*Culex sp.*	9
2019	Dezembro	23/12/2019	52	30	Teotônio	P03	Culicidae	Culicinae	*Mansonia*	*Mansonia amazonensis*	2
2019	Dezembro	23/12/2019	52	30	São Domingos	P02	Culicidae	Culicinae	*Aedes*	*Aedes sp.*	1
2019	Dezembro	23/12/2019	52	30	São Domingos	P02	Culicidae	Culicinae	*Coquillettidia*	*Coquillettidia sp.*	4
2019	Dezembro	23/12/2019	52	30	São Domingos	P02	Culicidae	Culicinae	*Culex*	*Culex sp.*	65
2019	Dezembro	23/12/2019	52	30	São Domingos	P02	Culicidae	Culicinae	*Mansonia*	*Mansonia sp.*	21
2019	Dezembro	23/12/2019	52	30	Cujubim Grande	P01	Culicidae	Culicinae	*Culex*	*Culex sp.*	70
2019	Dezembro	23/12/2019	52	30	Cujubim Grande	P01	Culicidae	Culicinae	*Mansonia*	*Mansonia sp.*	4
2019	Dezembro	23/12/2019	52	30	Joana D'arc Line 15	P10	Culicidae	Culicinae	*Culex*	*Culex sp.*	12
2019	Dezembro	23/12/2019	52	30	Joana D'arc Line 15	P10	Culicidae	Culicinae	*Mansonia*	*Mansonia sp.*	15
2019	Dezembro	23/12/2019	52	30	Joana D'arc Line 09	P09	Culicidae	Culicinae	*Mansonia*	*Mansonia sp.*	8
2020	Janeiro	30/12/2019	1	31	Rio Contra	P07	Culicidae	Anophelinae	*Anopheles*	*Anopheles darlingi*	1
2020	Janeiro	30/12/2019	1	31	Rio Contra	P07	Culicidae	Culicinae	*Culex*	*Culex sp.*	5
2020	Janeiro	30/12/2019	1	31	Rio Contra	P07	Culicidae	Culicinae	*Mansonia*	*Mansonia sp.*	1
2020	Janeiro	30/12/2019	1	31	Samauma	P08	Culicidae	Culicinae	*Aedeomyia*	*Adeomyia squamipennis*	4
2020	Janeiro	30/12/2019	1	31	Samauma	P08	Culicidae	Culicinae	*Culex*	*Culex sp.*	50
2020	Janeiro	30/12/2019	1	31	Samauma	P08	Culicidae	Culicinae	*Limatus*	*Limatus durhamii*	10
2020	Janeiro	30/12/2019	1	31	Samauma	P08	Culicidae	Culicinae	*Mansonia*	*Mansonia sp.*	380
2020	Janeiro	30/12/2019	1	31	Jaci Paraná	P06	Culicidae	Culicinae	*Aedeomyia*	*Adeomyia squamipennis*	2
2020	Janeiro	30/12/2019	1	31	Jaci Paraná	P06	Culicidae	Culicinae	*Aedes*	*Aedes scapularis*	15
2020	Janeiro	30/12/2019	1	31	Jaci Paraná	P06	Culicidae	Culicinae	*Coquillettidia*	*Coquillettidia sp.*	1
2020	Janeiro	30/12/2019	1	31	Jaci Paraná	P06	Culicidae	Culicinae	*Culex*	*Culex sp.*	100
2020	Janeiro	30/12/2019	1	31	Jaci Paraná	P06	Culicidae	Culicinae	*Mansonia*	*Mansonia sp.*	22
2020	Janeiro	30/12/2019	1	31	Santa Rita	P05	Culicidae	Culicinae	*Culex*	*Culex sp.*	91
2020	Janeiro	30/12/2019	1	31	Santa Rita	P05	Culicidae	Culicinae	*Mansonia*	*Mansonia sp.*	10
2020	Janeiro	30/12/2019	1	31	Morrinhos	P04	Culicidae	Culicinae	*Culex*	*Culex sp.*	56
2020	Janeiro	30/12/2019	1	31	Morrinhos	P04	Culicidae	Culicinae	*Mansonia*	*Mansonia sp.*	164
2020	Janeiro	30/12/2019	1	31	Teotônio	P03	Culicidae	Culicinae	*Coquillettidia*	*Coquillettidia sp.*	3
2020	Janeiro	30/12/2019	1	31	Teotônio	P03	Culicidae	Culicinae	*Limatus*	*Limatus durhamii*	2
2020	Janeiro	30/12/2019	1	31	Teotônio	P03	Culicidae	Culicinae	*Mansonia*	*Mansonia amazonensis*	2
2020	Janeiro	30/12/2019	1	31	São Domingos	P02	Culicidae	Culicinae	*Culex*	*Culex sp.*	8
2020	Janeiro	30/12/2019	1	31	São Domingos	P02	Culicidae	Culicinae	*Mansonia*	*Mansonia sp.*	2
2020	Janeiro	30/12/2019	1	31	Cujubim Grande	P01	Culicidae	Culicinae	*Culex*	*Culex sp.*	40
2020	Janeiro	30/12/2019	1	31	Joana D'arc Line 15	P10	Culicidae	Culicinae	*Culex*	*Culex sp.*	16
2020	Janeiro	30/12/2019	1	31	Joana D'arc Line 15	P10	Culicidae	Culicinae	*Mansonia*	*Mansonia sp.*	25
2020	Janeiro	30/12/2019	1	31	Joana D'arc Line 09	P09	Culicidae	Anophelinae	*Anopheles*	*Anopheles sp.*	2
2020	Janeiro	30/12/2019	1	31	Joana D'arc Line 09	P09	Culicidae	Culicinae	*Culex*	*Culex sp.*	9
2020	Janeiro	30/12/2019	1	31	Joana D'arc Line 09	P09	Culicidae	Culicinae	*Mansonia*	*Mansonia flaveola*	8
2020	Janeiro	06/01/2020	2	32	Rio Contra	P07	Culicidae	Culicinae	*Culex*	*Culex sp.*	2
2020	Janeiro	06/01/2020	2	32	Rio Contra	P07	Culicidae	Culicinae	*Mansonia*	*Mansonia sp.*	5
2020	Janeiro	06/01/2020	2	32	Samauma	P08	Culicidae	Culicinae	*Culex*	*Culex sp.*	81
2020	Janeiro	06/01/2020	2	32	Samauma	P08	Culicidae	Culicinae	*Limatus*	*Limatus durhamii*	2
2020	Janeiro	06/01/2020	2	32	Samauma	P08	Culicidae	Culicinae	*Mansonia*	*Mansonia sp.*	88
2020	Janeiro	06/01/2020	2	32	Jaci Paraná	P06	Culicidae	Culicinae	*Aedes*	*Aedes scapularis*	14
2020	Janeiro	06/01/2020	2	32	Jaci Paraná	P06	Culicidae	Culicinae	*Aedes*	*Aedes sp.*	15
2020	Janeiro	06/01/2020	2	32	Jaci Paraná	P06	Culicidae	Anophelinae	*Anopheles*	*Anopheles darlingi*	1
2020	Janeiro	06/01/2020	2	32	Jaci Paraná	P06	Culicidae	Culicinae	*Culex*	*Culex sp.*	100
2020	Janeiro	06/01/2020	2	32	Jaci Paraná	P06	Culicidae	Culicinae	*Mansonia*	*Mansonia sp.*	44
2020	Janeiro	06/01/2020	2	32	Jaci Paraná	P06	Culicidae	Culicinae	*Psorophora*	*Psorophora ferox*	6
2020	Janeiro	06/01/2020	2	32	Santa Rita	P05	Culicidae	Culicinae	*Culex*	*Culex sp.*	60
2020	Janeiro	06/01/2020	2	32	Santa Rita	P05	Culicidae	Culicinae	*Mansonia*	*Mansonia sp.*	12
2020	Janeiro	06/01/2020	2	32	Morrinhos	P04	Culicidae	Culicinae	*Aedes*	*Aedes scapularis*	1
2020	Janeiro	06/01/2020	2	32	Morrinhos	P04	Culicidae	Culicinae	*Culex*	*Culex sp.*	10
2020	Janeiro	06/01/2020	2	32	Morrinhos	P04	Culicidae	Culicinae	*Mansonia*	*Mansonia sp.*	145
2020	Janeiro	06/01/2020	2	32	Teotônio	P03	Culicidae	Culicinae	*Coquillettidia*	*Coquillettidia sp.*	3
2020	Janeiro	06/01/2020	2	32	Teotônio	P03	Culicidae	Culicinae	*Culex*	*Culex sp.*	10
2020	Janeiro	06/01/2020	2	32	Teotônio	P03	Culicidae	Culicinae	*Mansonia*	*Mansonia sp.*	1
2020	Janeiro	06/01/2020	2	32	São Domingos	P02	Culicidae	Culicinae	*Coquillettidia*	*Coquillettidia venezuelensis*	2
2020	Janeiro	06/01/2020	2	32	São Domingos	P02	Culicidae	Culicinae	*Culex*	*Culex sp.*	9
2020	Janeiro	06/01/2020	2	32	São Domingos	P02	Culicidae	Culicinae	*Mansonia*	*Mansonia sp.*	4
2020	Janeiro	06/01/2020	2	32	Cujubim Grande	P01	Culicidae	Anophelinae	*Anopheles*	*Anopheles darlingi*	1
2020	Janeiro	06/01/2020	2	32	Cujubim Grande	P01	Culicidae	Culicinae	*Culex*	*Culex sp.*	8
2020	Janeiro	06/01/2020	2	32	Joana D'arc Line 15	P10	Culicidae	Culicinae	*Mansonia*	*Mansonia sp.*	6
2020	Janeiro	06/01/2020	2	32	Joana D'arc Line 09	P09	Culicidae	Culicinae	*Culex*	*Culex sp.*	3
2020	Janeiro	06/01/2020	2	32	Joana D'arc Line 09	P09	Culicidae	Culicinae	*Mansonia*	*Mansonia sp.*	8
2020	Janeiro	13/01/2020	3	33	Rio Contra	P07	Culicidae	Culicinae	*Culex*	*Culex sp.*	2
2020	Janeiro	13/01/2020	3	33	Rio Contra	P07	Culicidae	Culicinae	*Limatus*	*Limatus durhamii*	1
2020	Janeiro	13/01/2020	3	33	Samauma	P08	Culicidae	Culicinae	*Aedeomyia*	*Adeomyia squamipennis*	1
2020	Janeiro	13/01/2020	3	33	Samauma	P08	Culicidae	Culicinae	*Coquillettidia*	*Coquillettidia sp.*	2
2020	Janeiro	13/01/2020	3	33	Samauma	P08	Culicidae	Culicinae	*Culex*	*Culex sp.*	93
2020	Janeiro	13/01/2020	3	33	Samauma	P08	Culicidae	Culicinae	*Limatus*	*Limatus durhamii*	5
2020	Janeiro	13/01/2020	3	33	Samauma	P08	Culicidae	Culicinae	*Mansonia*	*Mansonia sp.*	109
2020	Janeiro	13/01/2020	3	33	Jaci Paraná	P06	Culicidae	Culicinae	*Aedeomyia*	*Adeomyia squamipennis*	2
2020	Janeiro	13/01/2020	3	33	Jaci Paraná	P06	Culicidae	Culicinae	*Aedes*	*Aedes scapularis*	33
2020	Janeiro	13/01/2020	3	33	Jaci Paraná	P06	Culicidae	Culicinae	*Aedes*	*Aedes sp.*	33
2020	Janeiro	13/01/2020	3	33	Jaci Paraná	P06	Culicidae	Anophelinae	*Anopheles*	*Anopheles darlingi*	1
2020	Janeiro	13/01/2020	3	33	Jaci Paraná	P06	Culicidae	Culicinae	*Culex*	*Culex sp.*	113
2020	Janeiro	13/01/2020	3	33	Jaci Paraná	P06	Culicidae	Culicinae	*Limatus*	*Limatus durhamii*	1
2020	Janeiro	13/01/2020	3	33	Jaci Paraná	P06	Culicidae	Culicinae	*Mansonia*	*Mansonia sp.*	60
2020	Janeiro	13/01/2020	3	33	Jaci Paraná	P06	Culicidae	Culicinae	*Psorophora*	*Psorophora sp.*	5
2020	Janeiro	13/01/2020	3	33	Santa Rita	P05	Culicidae	Culicinae	*Aedeomyia*	*Adeomyia squamipennis*	2

2020	Janeiro	13/01/2020	3	33	Santa Rita	P05	Culicidae	Culicinae	*Coquillettidia*	*Coquillettidia venezuelensis*	2
2020	Janeiro	13/01/2020	3	33	Santa Rita	P05	Culicidae	Culicinae	*Culex*	*Culex sp.*	17
2020	Janeiro	13/01/2020	3	33	Santa Rita	P05	Culicidae	Culicinae	*Mansonia*	*Mansonia sp.*	30
2020	Janeiro	13/01/2020	3	33	Morrinhos	P04	Culicidae	Culicinae	*Aedes*	*Aedes scapularis*	1
2020	Janeiro	13/01/2020	3	33	Morrinhos	P04	Culicidae	Culicinae	*Coquillettidia*	*Coquillettidia sp.*	1
2020	Janeiro	13/01/2020	3	33	Morrinhos	P04	Culicidae	Culicinae	*Culex*	*Culex sp.*	12
2020	Janeiro	13/01/2020	3	33	Morrinhos	P04	Culicidae	Culicinae	*Mansonia*	*Mansonia sp.*	208
2020	Janeiro	13/01/2020	3	33	São Domingos	P02	Culicidae	Culicinae	*Mansonia*	*Mansonia sp.*	2
2020	Janeiro	13/01/2020	3	33	Cujubim Grande	P01	Culicidae	Culicinae	*Culex*	*Culex sp.*	42
2020	Janeiro	13/01/2020	3	33	Cujubim Grande	P01	Culicidae	Culicinae	*Limatus*	*Limatus durhamii*	4
2020	Janeiro	13/01/2020	3	33	Cujubim Grande	P01	Culicidae	Culicinae	*Mansonia*	*Mansonia sp.*	3
2020	Janeiro	13/01/2020	3	33	Joana D'arc Line 15	P10	Culicidae	Culicinae	*Culex*	*Culex sp.*	1
2020	Janeiro	13/01/2020	3	33	Joana D'arc Line 15	P10	Culicidae	Culicinae	*Mansonia*	*Mansonia sp.*	6
2020	Janeiro	13/01/2020	3	33	Joana D'arc Line 15	P10	Culicidae	Culicinae	*Psorophora*	*Psorophora sp.*	1
2020	Janeiro	13/01/2020	3	33	Joana D'arc Line 09	P09	Culicidae	Anophelinae	*Anopheles*	*Anopheles sp.*	1
2020	Janeiro	13/01/2020	3	33	Joana D'arc Line 09	P09	Culicidae	Culicinae	*Culex*	*Culex sp.*	5
2020	Janeiro	13/01/2020	3	33	Joana D'arc Line 09	P09	Culicidae	Culicinae	*Mansonia*	*Mansonia sp.*	4
2020	Janeiro	13/01/2020	3	33	Joana D'arc Line 09	P09	Culicidae	Culicinae	*Wyeomyia*	*Wyeomyia sp.*	1
2020	Janeiro	20/01/2020	4	34	Rio Contra	P07	Culicidae	Culicinae	*Mansonia*	*Mansonia sp.*	9
2020	Janeiro	20/01/2020	4	34	Samauma	P08	Culicidae	Culicinae	*Aedeomyia*	*Adeomyia squamipennis*	9
2020	Janeiro	20/01/2020	4	34	Samauma	P08	Culicidae	Culicinae	*Aedes*	*Aedes scapularis*	1
2020	Janeiro	20/01/2020	4	34	Samauma	P08	Culicidae	Culicinae	*Coquillettidia*	*Coquillettidia sp.*	1
2020	Janeiro	20/01/2020	4	34	Samauma	P08	Culicidae	Culicinae	*Culex*	*Culex sp.*	164
2020	Janeiro	20/01/2020	4	34	Samauma	P08	Culicidae	Culicinae	*Limatus*	*Limatus durhamii*	3
2020	Janeiro	20/01/2020	4	34	Samauma	P08	Culicidae	Culicinae	*Mansonia*	*Mansonia sp.*	636
2020	Janeiro	20/01/2020	4	34	Jaci Paraná	P06	Culicidae	Culicinae	*Aedeomyia*	*Adeomyia squamipennis*	5
2020	Janeiro	20/01/2020	4	34	Jaci Paraná	P06	Culicidae	Culicinae	*Aedes*	*Aedes scapularis*	7
2020	Janeiro	20/01/2020	4	34	Jaci Paraná	P06	Culicidae	Culicinae	*Aedes*	*Aedes sp.*	13
2020	Janeiro	20/01/2020	4	34	Jaci Paraná	P06	Culicidae	Culicinae	*Culex*	*Culex sp.*	85
2020	Janeiro	20/01/2020	4	34	Jaci Paraná	P06	Culicidae	Culicinae	*Limatus*	*Limatus durhamii*	2
2020	Janeiro	20/01/2020	4	34	Jaci Paraná	P06	Culicidae	Culicinae	*Mansonia*	*Mansonia sp.*	37
2020	Janeiro	20/01/2020	4	34	Jaci Paraná	P06	Culicidae	Culicinae	*Psorophora*	*Psorophora sp.*	3
2020	Janeiro	20/01/2020	4	34	Santa Rita	P05	Culicidae	Culicinae	*Culex*	*Culex sp.*	21
2020	Janeiro	20/01/2020	4	34	Santa Rita	P05	Culicidae	Culicinae	*Mansonia*	*Mansonia sp.*	63
2020	Janeiro	20/01/2020	4	34	Morrinhos	P04	Culicidae	Culicinae	*Culex*	*Culex sp.*	13
2020	Janeiro	20/01/2020	4	34	Morrinhos	P04	Culicidae	Culicinae	*Mansonia*	*Mansonia sp.*	260
2020	Janeiro	20/01/2020	4	34	Teotônio	P03	Culicidae	Culicinae	*Coquillettidia*	*Coquillettidia sp.*	1
2020	Janeiro	20/01/2020	4	34	Teotônio	P03	Culicidae	Culicinae	*Culex*	*Culex sp.*	2
2020	Janeiro	20/01/2020	4	34	Teotônio	P03	Culicidae	Culicinae	*Limatus*	*Limatus durhamii*	1
2020	Janeiro	20/01/2020	4	34	São Domingos	P02	Culicidae	Culicinae	*Culex*	*Culex sp.*	2
2020	Janeiro	20/01/2020	4	34	São Domingos	P02	Culicidae	Culicinae	*Mansonia*	*Mansonia sp.*	2
2020	Janeiro	20/01/2020	4	34	Cujubim Grande	P01	Culicidae	Culicinae	*Aedes*	*Aedes sp.*	1
2020	Janeiro	20/01/2020	4	34	Cujubim Grande	P01	Culicidae	Culicinae	*Culex*	*Culex sp.*	51
2020	Janeiro	20/01/2020	4	34	Cujubim Grande	P01	Culicidae	Culicinae	*Limatus*	*Limatus durhamii*	2
2020	Janeiro	20/01/2020	4	34	Cujubim Grande	P01	Culicidae	Culicinae	*Mansonia*	*Mansonia sp.*	3
2020	Janeiro	20/01/2020	4	34	Joana D'arc Line 15	P10	Culicidae	Culicinae	*Culex*	*Culex sp.*	4
2020	Janeiro	20/01/2020	4	34	Joana D'arc Line 15	P10	Culicidae	Culicinae	*Mansonia*	*Mansonia indubitans*	1
2020	Janeiro	20/01/2020	4	34	Joana D'arc Line 09	P09	Culicidae	Anophelinae	*Anopheles*	*Anopheles sp.*	1
2020	Janeiro	20/01/2020	4	34	Joana D'arc Line 09	P09	Culicidae	Culicinae	*Culex*	*Culex sp.*	2
2020	Janeiro	20/01/2020	4	34	Joana D'arc Line 09	P09	Culicidae	Culicinae	*Wyeomyia*	*Wyeomyia sp.*	1
2020	Janeiro	27/01/2020	5	35	Rio Contra	P07	Culicidae	Culicinae	*Culex*	*Culex sp.*	1
2020	Janeiro	27/01/2020	5	35	Rio Contra	P07	Culicidae	Culicinae	*Mansonia*	*Mansonia sp.*	1
2020	Janeiro	27/01/2020	5	35	Samauma	P08	Culicidae	Culicinae	*Aedeomyia*	*Adeomyia squamipennis*	6
2020	Janeiro	27/01/2020	5	35	Samauma	P08	Culicidae	Anophelinae	*Anopheles*	*Anopheles darlingi*	1
2020	Janeiro	27/01/2020	5	35	Samauma	P08	Culicidae	Culicinae	*Coquillettidia*	*Coquillettidia sp.*	1
2020	Janeiro	27/01/2020	5	35	Samauma	P08	Culicidae	Culicinae	*Culex*	*Culex sp.*	188
2020	Janeiro	27/01/2020	5	35	Samauma	P08	Culicidae	Culicinae	*Limatus*	*Limatus durhamii*	3
2020	Janeiro	27/01/2020	5	35	Samauma	P08	Culicidae	Culicinae	*Mansonia*	*Mansonia sp.*	575
2020	Janeiro	27/01/2020	5	35	Jaci Paraná	P06	Culicidae	Culicinae	*Aedeomyia*	*Adeomyia squamipennis*	1
2020	Janeiro	27/01/2020	5	35	Jaci Paraná	P06	Culicidae	Culicinae	*Aedes*	*Aedes aegypti*	11
2020	Janeiro	27/01/2020	5	35	Jaci Paraná	P06	Culicidae	Culicinae	*Culex*	*Culex sp.*	28
2020	Janeiro	27/01/2020	5	35	Jaci Paraná	P06	Culicidae	Culicinae	*Mansonia*	*Mansonia sp.*	65
2020	Janeiro	27/01/2020	5	35	Santa Rita	P05	Culicidae	Culicinae	*Culex*	*Culex sp.*	6
2020	Janeiro	27/01/2020	5	35	Santa Rita	P05	Culicidae	Culicinae	*Limatus*	*Limatus durhamii*	1
2020	Janeiro	27/01/2020	5	35	Santa Rita	P05	Culicidae	Culicinae	*Mansonia*	*Mansonia sp.*	49
2020	Janeiro	27/01/2020	5	35	Morrinhos	P04	Culicidae	Culicinae	*Culex*	*Culex sp.*	10
2020	Janeiro	27/01/2020	5	35	Morrinhos	P04	Culicidae	Culicinae	*Limatus*	*Limatus durhamii*	1
2020	Janeiro	27/01/2020	5	35	Morrinhos	P04	Culicidae	Culicinae	*Mansonia*	*Mansonia sp.*	187
2020	Janeiro	27/01/2020	5	35	Teotônio	P03	Culicidae	Culicinae	*Culex*	*Culex sp.*	2
2020	Janeiro	27/01/2020	5	35	São Domingos	P02	Culicidae	Anophelinae	*Anopheles*	*Anopheles darlingi*	1
2020	Janeiro	27/01/2020	5	35	São Domingos	P02	Culicidae	Culicinae	*Culex*	*Culex sp.*	30
2020	Janeiro	27/01/2020	5	35	São Domingos	P02	Culicidae	Culicinae	*Mansonia*	*Mansonia sp.*	1
2020	Janeiro	27/01/2020	5	35	Cujubim Grande	P01	Culicidae	Culicinae	*Coquillettidia*	*Coquillettidia venezuelensis*	1
2020	Janeiro	27/01/2020	5	35	Cujubim Grande	P01	Culicidae	Culicinae	*Culex*	*Culex sp.*	7
2020	Janeiro	27/01/2020	5	35	Cujubim Grande	P01	Culicidae	Culicinae	*Limatus*	*Limatus durhamii*	1
2020	Janeiro	27/01/2020	5	35	Cujubim Grande	P01	Culicidae	Culicinae	*Mansonia*	*Mansonia sp.*	2
2020	Janeiro	27/01/2020	5	35	Joana D'arc Line 15	P10	Culicidae	Culicinae	*Culex*	*Culex sp.*	4
2020	Janeiro	27/01/2020	5	35	Joana D'arc Line 15	P10	Culicidae	Culicinae	*Mansonia*	*Mansonia indubitans*	1
2020	Janeiro	27/01/2020	5	35	Joana D'arc Line 15	P10	Culicidae	Culicinae	*Wyeomyia*	*Wyeomyia sp.*	1
2020	Janeiro	27/01/2020	5	35	Joana D'arc Line 09	P09	Culicidae	Culicinae	*Culex*	*Culex sp.*	10
2020	Fevereiro	03/02/2020	6	36	Rio Contra	P07	Culicidae	Culicinae	*Mansonia*	*Mansonia sp.*	1
2020	Fevereiro	03/02/2020	6	36	Jaci Paraná	P06	Culicidae	Culicinae	*Aedeomyia*	*Adeomyia squamipennis*	4
2020	Fevereiro	03/02/2020	6	36	Jaci Paraná	P06	Culicidae	Culicinae	*Aedes*	*Aedes scapularis*	1
2020	Fevereiro	03/02/2020	6	36	Jaci Paraná	P06	Culicidae	Culicinae	*Aedes*	*Aedes sp.*	8
2020	Fevereiro	03/02/2020	6	36	Jaci Paraná	P06	Culicidae	Anophelinae	*Anopheles*	*Anopheles darlingi*	2
2020	Fevereiro	03/02/2020	6	36	Jaci Paraná	P06	Culicidae	Culicinae	*Culex*	*Culex sp.*	25
2020	Fevereiro	03/02/2020	6	36	Jaci Paraná	P06	Culicidae	Culicinae	*Mansonia*	*Mansonia sp.*	116
2020	Fevereiro	03/02/2020	6	36	Santa Rita	P05	Culicidae	Anophelinae	*Anopheles*	*Anopheles darlingi*	2
2020	Fevereiro	03/02/2020	6	36	Santa Rita	P05	Culicidae	Culicinae	*Coquillettidia*	*Coquillettidia sp.*	1
2020	Fevereiro	03/02/2020	6	36	Santa Rita	P05	Culicidae	Culicinae	*Culex*	*Culex sp.*	3
2020	Fevereiro	03/02/2020	6	36	Santa Rita	P05	Culicidae	Culicinae	*Mansonia*	*Mansonia sp.*	89
2020	Fevereiro	03/02/2020	6	36	Morrinhos	P04	Culicidae	Culicinae	*Culex*	*Culex sp.*	10
2020	Fevereiro	03/02/2020	6	36	Morrinhos	P04	Culicidae	Culicinae	*Mansonia*	*Mansonia sp.*	250
2020	Fevereiro	03/02/2020	6	36	Teotônio	P03	Culicidae	Culicinae	*Coquillettidia*	*Coquillettidia sp.*	1
2020	Fevereiro	03/02/2020	6	36	Teotônio	P03	Culicidae	Culicinae	*Limatus*	*Limatus durhamii*	1
2020	Fevereiro	03/02/2020	6	36	Teotônio	P03	Culicidae	Culicinae	*Mansonia*	*Mansonia sp.*	1
2020	Fevereiro	03/02/2020	6	36	São Domingos	P02	Culicidae	Culicinae	*Coquillettidia*	*Coquillettidia sp.*	1
2020	Fevereiro	03/02/2020	6	36	São Domingos	P02	Culicidae	Culicinae	*Culex*	*Culex sp.*	16
2020	Fevereiro	03/02/2020	6	36	São Domingos	P02	Culicidae	Culicinae	*Mansonia*	*Mansonia sp.*	1
2020	Fevereiro	03/02/2020	6	36	Cujubim Grande	P01	Culicidae	Culicinae	*Culex*	*Culex sp.*	6
2020	Fevereiro	03/02/2020	6	36	Cujubim Grande	P01	Culicidae	Culicinae	*Mansonia*	*Mansonia sp.*	1
2020	Fevereiro	03/02/2020	6	36	Joana D'arc Line 15	P10	Culicidae	Culicinae	*Wyeomyia*	*Wyeomyia sp.*	1
2020	Fevereiro	03/02/2020	6	36	Joana D'arc Line 09	P09	Culicidae	Culicinae	*Culex*	*Culex sp.*	4
2020	Fevereiro	03/02/2020	6	36	Joana D'arc Line 09	P09	Culicidae	Culicinae	*Mansonia*	*Mansonia titillans*	1
2020	Fevereiro	10/02/2020	7	37	Rio Contra	P07	Culicidae	Culicinae	*Mansonia*	*Mansonia sp.*	1
2020	Fevereiro	10/02/2020	7	37	Samauma	P08	Culicidae	Culicinae	*Aedeomyia*	*Adeomyia squamipennis*	9
2020	Fevereiro	10/02/2020	7	37	Samauma	P08	Culicidae	Culicinae	*Culex*	*Culex sp.*	181
2020	Fevereiro	10/02/2020	7	37	Samauma	P08	Culicidae	Culicinae	*Limatus*	*Limatus durhamii*	6
2020	Fevereiro	10/02/2020	7	37	Samauma	P08	Culicidae	Culicinae	*Mansonia*	*Mansonia sp.*	816
2020	Fevereiro	10/02/2020	7	37	Jaci Paraná	P06	Culicidae	Culicinae	*Aedes*	*Aedes scapularis*	2
2020	Fevereiro	10/02/2020	7	37	Jaci Paraná	P06	Culicidae	Culicinae	*Aedes*	*Aedes sp.*	15
2020	Fevereiro	10/02/2020	7	37	Jaci Paraná	P06	Culicidae	Culicinae	*Culex*	*Culex sp.*	15
2020	Fevereiro	10/02/2020	7	37	Jaci Paraná	P06	Culicidae	Culicinae	*Limatus*	*Limatus durhamii*	1
2020	Fevereiro	10/02/2020	7	37	Jaci Paraná	P06	Culicidae	Culicinae	*Mansonia*	*Mansonia sp.*	29
2020	Fevereiro	10/02/2020	7	37	Santa Rita	P05	Culicidae	Culicinae	*Culex*	*Culex sp.*	10
2020	Fevereiro	10/02/2020	7	37	Santa Rita	P05	Culicidae	Culicinae	*Mansonia*	*Mansonia sp.*	81
2020	Fevereiro	10/02/2020	7	37	Morrinhos	P04	Culicidae	Culicinae	*Coquillettidia*	*Coquillettidia sp.*	1
2020	Fevereiro	10/02/2020	7	37	Morrinhos	P04	Culicidae	Culicinae	*Culex*	*Culex sp.*	11

2020	Fevereiro	10/02/2020	7	37	Morrinhos	P04	Culicidae	Culicinae	*Mansonia*	*Mansonia sp.*	243
2020	Fevereiro	10/02/2020	7	37	Teotônio	P03	Culicidae	Culicinae	*Culex*	*Culex sp.*	3
2020	Fevereiro	10/02/2020	7	37	Teotônio	P03	Culicidae	Culicinae	*Limatus*	*Limatus durhamii*	1
2020	Fevereiro	10/02/2020	7	37	São Domingos	P02	Culicidae	Anophelinae	*Anopheles*	*Anopheles darlingi*	1
2020	Fevereiro	10/02/2020	7	37	São Domingos	P02	Culicidae	Culicinae	*Coquillettidia*	*Coquillettidia sp.*	2
2020	Fevereiro	10/02/2020	7	37	São Domingos	P02	Culicidae	Culicinae	*Culex*	*Culex sp.*	15
2020	Fevereiro	10/02/2020	7	37	São Domingos	P02	Culicidae	Culicinae	*Mansonia*	*Mansonia sp.*	6
2020	Fevereiro	10/02/2020	7	37	Cujubim Grande	P01	Culicidae	Culicinae	*Culex*	*Culex sp.*	15
2020	Fevereiro	10/02/2020	7	37	Cujubim Grande	P01	Culicidae	Culicinae	*Limatus*	*Limatus durhamii*	1
2020	Fevereiro	10/02/2020	7	37	Cujubim Grande	P01	Culicidae	Culicinae	*Mansonia*	*Mansonia sp.*	4
2020	Fevereiro	10/02/2020	7	37	Joana D'arc Line 15	P10	Culicidae	Culicinae	*Culex*	*Culex coronator*	1
2020	Fevereiro	10/02/2020	7	37	Joana D'arc Line 15	P10	Culicidae	Culicinae	*Limatus*	*Limatus durhamii*	6
2020	Fevereiro	10/02/2020	7	37	Joana D'arc Line 15	P10	Culicidae	Culicinae	*Mansonia*	*Mansonia sp.*	8
2020	Fevereiro	10/02/2020	7	37	Joana D'arc Line 15	P10	Culicidae	Culicinae	*Wyeomyia*	*Wyeomyia sp.*	1
2020	Fevereiro	10/02/2020	7	37	Joana D'arc Line 09	P09	Culicidae	Anophelinae	*Anopheles*	*Anopheles darlingi*	1
2020	Fevereiro	10/02/2020	7	37	Joana D'arc Line 09	P09	Culicidae	Culicinae	*Mansonia*	*Mansonia sp.*	16
2020	Fevereiro	17/02/2020	8	38	Rio Contra	P07	Culicidae	Anophelinae	*Anopheles*	*Anopheles sp.*	6
2020	Fevereiro	17/02/2020	8	38	Rio Contra	P07	Culicidae	Culicinae	*Culex*	*Culex sp.*	5
2020	Fevereiro	17/02/2020	8	38	Rio Contra	P07	Culicidae	Culicinae	*Mansonia*	*Mansonia sp.*	17
2020	Fevereiro	17/02/2020	8	38	Samauma	P08	Culicidae	Culicinae	*Aedeomyia*	*Adeomyia squamipennis*	10
2020	Fevereiro	17/02/2020	8	38	Samauma	P08	Culicidae	Culicinae	*Aedes*	*Aedes scapularis*	22
2020	Fevereiro	17/02/2020	8	38	Samauma	P08	Culicidae	Culicinae	*Culex*	*Culex sp.*	108
2020	Fevereiro	17/02/2020	8	38	Samauma	P08	Culicidae	Culicinae	*Mansonia*	*Mansonia sp.*	1237
2020	Fevereiro	17/02/2020	8	38	Samauma	P08	Culicidae	Culicinae	*Uranotaenia*	*Uranotaenia sp.*	3
2020	Fevereiro	17/02/2020	8	38	Jaci Paraná	P06	Culicidae	Culicinae	*Culex*	*Culex sp.*	12
2020	Fevereiro	17/02/2020	8	38	Jaci Paraná	P06	Culicidae	Culicinae	*Mansonia*	*Mansonia sp.*	43
2020	Fevereiro	17/02/2020	8	38	Santa Rita	P05	Culicidae	Culicinae	*Aedes*	*Aedes scapularis*	8
2020	Fevereiro	17/02/2020	8	38	Santa Rita	P05	Culicidae	Culicinae	*Culex*	*Culex sp.*	11
2020	Fevereiro	17/02/2020	8	38	Santa Rita	P05	Culicidae	Culicinae	*Mansonia*	*Mansonia sp.*	39
2020	Fevereiro	17/02/2020	8	38	Morrinhos	P04	Culicidae	Culicinae	*Aedes*	*Aedes scapularis*	5
2020	Fevereiro	17/02/2020	8	38	Morrinhos	P04	Culicidae	Culicinae	*Mansonia*	*Mansonia sp.*	72
2020	Fevereiro	17/02/2020	8	38	Teotônio	P03	Culicidae	Culicinae	*Culex*	*Culex sp.*	6
2020	Fevereiro	17/02/2020	8	38	Teotônio	P03	Culicidae	Culicinae	*Mansonia*	*Mansonia sp.*	12
2020	Fevereiro	17/02/2020	8	38	São Domingos	P02	Culicidae	Culicinae	*Aedes*	*Aedes aegypti*	2
2020	Fevereiro	17/02/2020	8	38	São Domingos	P02	Culicidae	Culicinae	*Culex*	*Culex sp.*	4
2020	Fevereiro	17/02/2020	8	38	São Domingos	P02	Culicidae	Culicinae	*Mansonia*	*Mansonia sp.*	2
2020	Fevereiro	17/02/2020	8	38	Cujubim Grande	P01	Culicidae	Culicinae	*Culex*	*Culex sp.*	72
2020	Fevereiro	17/02/2020	8	38	Cujubim Grande	P01	Culicidae	Culicinae	*Mansonia*	*Mansonia sp.*	3
2020	Fevereiro	17/02/2020	8	38	Joana D'arc Line 15	P10	Culicidae	Culicinae	*Culex*	*Culex sp.*	1
2020	Fevereiro	17/02/2020	8	38	Joana D'arc Line 15	P10	Culicidae	Culicinae	*Mansonia*	*Mansonia sp.*	2
2020	Fevereiro	17/02/2020	8	38	Joana D'arc Line 15	P10	Culicidae	Culicinae	*Wyeomyia*	*Wyeomyia sp.*	1
2020	Fevereiro	17/02/2020	8	38	Joana D'arc Line 09	P09	Culicidae	Culicinae	*Culex*	*Culex sp.*	2
2020	Fevereiro	17/02/2020	8	38	Joana D'arc Line 09	P09	Culicidae	Culicinae	*Mansonia*	*Mansonia sp.*	15
2020	Fevereiro	24/02/2020	9	39	Rio Contra	P07	Culicidae	Culicinae	*Culex*	*Culex sp.*	2
2020	Fevereiro	24/02/2020	9	39	Rio Contra	P07	Culicidae	Culicinae	*Mansonia*	*Mansonia sp.*	5
2020	Fevereiro	24/02/2020	9	39	Samauma	P08	Culicidae	Culicinae	*Culex*	*Culex sp.*	430
2020	Fevereiro	24/02/2020	9	39	Samauma	P08	Culicidae	Culicinae	*Limatus*	*Limatus durhamii*	10
2020	Fevereiro	24/02/2020	9	39	Samauma	P08	Culicidae	Culicinae	*Mansonia*	*Mansonia sp.*	500
2020	Fevereiro	24/02/2020	9	39	Jaci Paraná	P06	Culicidae	Culicinae	*Aedes*	*Aedes scapularis*	4
2020	Fevereiro	24/02/2020	9	39	Jaci Paraná	P06	Culicidae	Culicinae	*Aedes*	*Aedes sp.*	5
2020	Fevereiro	24/02/2020	9	39	Jaci Paraná	P06	Culicidae	Culicinae	*Culex*	*Culex sp.*	5
2020	Fevereiro	24/02/2020	9	39	Jaci Paraná	P06	Culicidae	Culicinae	*Mansonia*	*Mansonia sp.*	43
2020	Fevereiro	24/02/2020	9	39	Santa Rita	P05	Culicidae	Culicinae	*Culex*	*Culex sp.*	5
2020	Fevereiro	24/02/2020	9	39	Santa Rita	P05	Culicidae	Culicinae	*Mansonia*	*Mansonia sp.*	86
2020	Fevereiro	24/02/2020	9	39	Morrinhos	P04	Culicidae	Culicinae	*Aedeomyia*	*Adeomyia squamipennis*	1
2020	Fevereiro	24/02/2020	9	39	Morrinhos	P04	Culicidae	Culicinae	*Coquillettidia*	*Coquillettidia sp.*	1
2020	Fevereiro	24/02/2020	9	39	Morrinhos	P04	Culicidae	Culicinae	*Culex*	*Culex sp.*	5
2020	Fevereiro	24/02/2020	9	39	Morrinhos	P04	Culicidae	Culicinae	*Limatus*	*Limatus durhamii*	1
2020	Fevereiro	24/02/2020	9	39	Morrinhos	P04	Culicidae	Culicinae	*Mansonia*	*Mansonia sp.*	253
2020	Fevereiro	24/02/2020	9	39	Teotônio	P03	Culicidae	Culicinae	*Culex*	*Culex sp.*	4
2020	Fevereiro	24/02/2020	9	39	Teotônio	P03	Culicidae	Culicinae	*Mansonia*	*Mansonia sp.*	2
2020	Fevereiro	24/02/2020	9	39	São Domingos	P02	Culicidae	Culicinae	*Culex*	*Culex sp.*	1
2020	Fevereiro	24/02/2020	9	39	São Domingos	P02	Culicidae	Culicinae	*Mansonia*	*Mansonia sp.*	3
2020	Fevereiro	24/02/2020	9	39	Cujubim Grande	P01	Culicidae	Culicinae	*Culex*	*Culex sp.*	18
2020	Fevereiro	24/02/2020	9	39	Cujubim Grande	P01	Culicidae	Culicinae	*Limatus*	*Limatus durhamii*	1
2020	Fevereiro	24/02/2020	9	39	Cujubim Grande	P01	Culicidae	Culicinae	*Mansonia*	*Mansonia sp.*	1
2020	Fevereiro	24/02/2020	9	39	Joana D'arc Line 15	P10	Culicidae	Culicinae	*Culex*	*Culex sp.*	1
2020	Fevereiro	24/02/2020	9	39	Joana D'arc Line 15	P10	Culicidae	Culicinae	*Mansonia*	*Mansonia indubitans*	8
2020	Fevereiro	24/02/2020	9	39	Joana D'arc Line 09	P09	Culicidae	Culicinae	*Mansonia*	*Mansonia sp.*	10
2020	Março	02/03/2020	10	40	Rio Contra	P07	Culicidae	Culicinae	*Culex*	*Culex sp.*	12
2020	Março	02/03/2020	10	40	Rio Contra	P07	Culicidae	Culicinae	*Mansonia*	*Mansonia sp.*	23
2020	Março	02/03/2020	10	40	Rio Contra	P07	Culicidae	Culicinae	*Psorophora*	*Psorophora confinnis*	3
2020	Março	02/03/2020	10	40	Samauma	P08	Culicidae	Culicinae	*Aedeomyia*	*Adeomyia squamipennis*	6
2020	Março	02/03/2020	10	40	Samauma	P08	Culicidae	Culicinae	*Culex*	*Culex sp.*	100
2020	Março	02/03/2020	10	40	Samauma	P08	Culicidae	Culicinae	*Limatus*	*Limatus durhamii*	13
2020	Março	02/03/2020	10	40	Samauma	P08	Culicidae	Culicinae	*Mansonia*	*Mansonia sp.*	355
2020	Março	02/03/2020	10	40	Jaci Paraná	P06	Culicidae	Culicinae	*Aedes*	*Aedes aegypti*	3
2020	Março	02/03/2020	10	40	Jaci Paraná	P06	Culicidae	Culicinae	*Aedes*	*Aedes scapularis*	2
2020	Março	02/03/2020	10	40	Jaci Paraná	P06	Culicidae	Culicinae	*Culex*	*Culex sp.*	5
2020	Março	02/03/2020	10	40	Jaci Paraná	P06	Culicidae	Culicinae	*Mansonia*	*Mansonia titillans*	12
2020	Março	02/03/2020	10	40	Santa Rita	P05	Culicidae	Culicinae	*Culex*	*Culex sp.*	13
2020	Março	02/03/2020	10	40	Santa Rita	P05	Culicidae	Culicinae	*Mansonia*	*Mansonia sp.*	87
2020	Março	02/03/2020	10	40	Morrinhos	P04	Culicidae	Culicinae	*Aedes*	*Aedes aegypti*	1
2020	Março	02/03/2020	10	40	Morrinhos	P04	Culicidae	Culicinae	*Culex*	*Culex sp.*	8
2020	Março	02/03/2020	10	40	Morrinhos	P04	Culicidae	Culicinae	*Mansonia*	*Mansonia sp.*	153
2020	Março	02/03/2020	10	40	Teotônio	P03	Culicidae	Culicinae	*Mansonia*	*Mansonia sp.*	2
2020	Março	02/03/2020	10	40	São Domingos	P02	Culicidae	Culicinae	*Culex*	*Culex sp.*	3
2020	Março	02/03/2020	10	40	São Domingos	P02	Culicidae	Culicinae	*Mansonia*	*Mansonia sp.*	5
2020	Março	02/03/2020	10	40	Cujubim Grande	P01	Culicidae	Culicinae	*Culex*	*Culex sp.*	7
2020	Março	02/03/2020	10	40	Joana D'arc Line 15	P10	Culicidae	Culicinae	*Culex*	*Culex sp.*	1
2020	Março	02/03/2020	10	40	Joana D'arc Line 15	P10	Culicidae	Culicinae	*Mansonia*	*Mansonia sp.*	3
2020	Março	02/03/2020	10	40	Joana D'arc Line 09	P09	Culicidae	Culicinae	*Culex*	*Culex sp.*	1
2020	Março	02/03/2020	10	40	Joana D'arc Line 09	P09	Culicidae	Culicinae	*Mansonia*	*Mansonia sp.*	12
2020	Março	09/03/2020	11	41	Samauma	P08	Culicidae	Culicinae	*Aedeomyia*	*Adeomyia squamipennis*	3
2020	Março	09/03/2020	11	41	Samauma	P08	Culicidae	Culicinae	*Coquillettidia*	*Coquillettidia sp.*	2
2020	Março	09/03/2020	11	41	Samauma	P08	Culicidae	Culicinae	*Culex*	*Culex sp.*	20
2020	Março	09/03/2020	11	41	Samauma	P08	Culicidae	Culicinae	*Limatus*	*Limatus durhamii*	4
2020	Março	09/03/2020	11	41	Samauma	P08	Culicidae	Culicinae	*Mansonia*	*Mansonia sp.*	506
2020	Março	09/03/2020	11	41	Jaci Paraná	P06	Culicidae	Culicinae	*Aedeomyia*	*Adeomyia squamipennis*	1
2020	Março	09/03/2020	11	41	Jaci Paraná	P06	Culicidae	Culicinae	*Aedes*	*Aedes sp.*	33
2020	Março	09/03/2020	11	41	Jaci Paraná	P06	Culicidae	Anophelinae	*Anopheles*	*Anopheles darlingi*	1
2020	Março	09/03/2020	11	41	Jaci Paraná	P06	Culicidae	Culicinae	*Culex*	*Culex sp.*	5
2020	Março	09/03/2020	11	41	Jaci Paraná	P06	Culicidae	Culicinae	*Limatus*	*Limatus durhamii*	2
2020	Março	09/03/2020	11	41	Jaci Paraná	P06	Culicidae	Culicinae	*Mansonia*	*Mansonia sp.*	10
2020	Março	09/03/2020	11	41	Santa Rita	P05	Culicidae	Culicinae	*Aedeomyia*	*Adeomyia squamipennis*	4
2020	Março	09/03/2020	11	41	Santa Rita	P05	Culicidae	Culicinae	*Coquillettidia*	*Coquillettidia sp.*	2
2020	Março	09/03/2020	11	41	Santa Rita	P05	Culicidae	Culicinae	*Mansonia*	*Mansonia sp.*	49
2020	Março	09/03/2020	11	41	Morrinhos	P04	Culicidae	Culicinae	*Aedes*	*Aedes sp.*	1
2020	Março	09/03/2020	11	41	Morrinhos	P04	Culicidae	Culicinae	*Culex*	*Culex sp.*	2
2020	Março	09/03/2020	11	41	Morrinhos	P04	Culicidae	Culicinae	*Limatus*	*Limatus durhamii*	2
2020	Março	09/03/2020	11	41	Morrinhos	P04	Culicidae	Culicinae	*Mansonia*	*Mansonia sp.*	51
2020	Março	09/03/2020	11	41	Teotônio	P03	Culicidae	Culicinae	*Coquillettidia*	*Coquillettidia sp.*	2
2020	Março	09/03/2020	11	41	Teotônio	P03	Culicidae	Culicinae	*Culex*	*Culex sp.*	2
2020	Março	09/03/2020	11	41	Teotônio	P03	Culicidae	Culicinae	*Mansonia*	*Mansonia sp.*	8
2020	Março	09/03/2020	11	41	São Domingos	P02	Culicidae	Culicinae	*Culex*	*Culex sp.*	4
2020	Março	09/03/2020	11	41	São Domingos	P02	Culicidae	Culicinae	*Limatus*	*Limatus durhamii*	1
2020	Março	09/03/2020	11	41	São Domingos	P02	Culicidae	Culicinae	*Psorophora*	*Psorophora sp.*	1
2020	Março	09/03/2020	11	41	Joana D'arc Line 15	P10	Culicidae	Culicinae	*Culex*	*Culex sp.*	1

2020	Março	09/03/2020	11	41	Joana D'arc Line 15	P10	Culicidae	Culicinae	*Mansonia*	*Mansonia sp.*	9
2020	Março	09/03/2020	11	41	Joana D'arc Line 09	P09	Culicidae	Culicinae	*Culex*	*Culex sp.*	1
2020	Março	09/03/2020	11	41	Joana D'arc Line 09	P09	Culicidae	Culicinae	*Limatus*	*Limatus durhamii*	2
2020	Março	09/03/2020	11	41	Joana D'arc Line 09	P09	Culicidae	Culicinae	*Mansonia*	*Mansonia sp.*	4
2020	Março	16/03/2020	12	42	Rio Contra	P07	Culicidae	Culicinae	*Mansonia*	*Mansonia sp.*	2
2020	Março	16/03/2020	12	42	Samauma	P08	Culicidae	Culicinae	*Aedeomyia*	*Adeomyia squamipennis*	4
2020	Março	16/03/2020	12	42	Samauma	P08	Culicidae	Culicinae	*Coquillettidia*	*Coquillettidia sp.*	1
2020	Março	16/03/2020	12	42	Samauma	P08	Culicidae	Culicinae	*Culex*	*Culex sp.*	14
2020	Março	16/03/2020	12	42	Samauma	P08	Culicidae	Culicinae	*Limatus*	*Limatus durhamii*	8
2020	Março	16/03/2020	12	42	Samauma	P08	Culicidae	Culicinae	*Mansonia*	*Mansonia sp.*	492
2020	Março	16/03/2020	12	42	Jaci Paraná	P06	Culicidae	Culicinae	*Aedeomyia*	*Adeomyia squamipennis*	3
2020	Março	16/03/2020	12	42	Jaci Paraná	P06	Culicidae	Culicinae	*Aedes*	*Aedes sp.*	1
2020	Março	16/03/2020	12	42	Jaci Paraná	P06	Culicidae	Culicinae	*Coquillettidia*	*Coquillettidia venezuelensis*	1
2020	Março	16/03/2020	12	42	Jaci Paraná	P06	Culicidae	Culicinae	*Culex*	*Culex sp.*	9
2020	Março	16/03/2020	12	42	Jaci Paraná	P06	Culicidae	Culicinae	*Mansonia*	*Mansonia sp.*	15
2020	Março	16/03/2020	12	42	Jaci Paraná	P06	Culicidae	Culicinae	*Psorophora*	*Psorophora confinnis*	2
2020	Março	16/03/2020	12	42	Santa Rita	P05	Culicidae	Culicinae	*Mansonia*	*Mansonia sp.*	40
2020	Março	16/03/2020	12	42	Morrinhos	P04	Culicidae	Culicinae	*Culex*	*Culex sp.*	2
2020	Março	16/03/2020	12	42	Morrinhos	P04	Culicidae	Culicinae	*Mansonia*	*Mansonia sp.*	52
2020	Março	16/03/2020	12	42	Teotônio	P03	Culicidae	Culicinae	*Aedeomyia*	*Adeomyia squamipennis*	2
2020	Março	16/03/2020	12	42	Teotônio	P03	Culicidae	Culicinae	*Coquillettidia*	*Coquillettidia sp.*	39
2020	Março	16/03/2020	12	42	Teotônio	P03	Culicidae	Culicinae	*Culex*	*Culex sp.*	5
2020	Março	16/03/2020	12	42	Teotônio	P03	Culicidae	Culicinae	*Mansonia*	*Mansonia sp.*	5
2020	Março	16/03/2020	12	42	São Domingos	P02	Culicidae	Culicinae	*Mansonia*	*Mansonia sp.*	3
2020	Março	16/03/2020	12	42	Cujubim Grande	P01	Culicidae	Culicinae	*Culex*	*Culex sp.*	1
2020	Março	16/03/2020	12	42	Cujubim Grande	P01	Culicidae	Culicinae	*Mansonia*	*Mansonia sp.*	2
2020	Março	23/03/2020	13	43	Rio Contra	P07	Culicidae	Culicinae	*Mansonia*	*Mansonia sp.*	22
2020	Março	23/03/2020	13	43	Samauma	P08	Culicidae	Culicinae	*Coquillettidia*	*Coquillettidia sp.*	11
2020	Março	23/03/2020	13	43	Samauma	P08	Culicidae	Culicinae	*Culex*	*Culex sp.*	18
2020	Março	23/03/2020	13	43	Samauma	P08	Culicidae	Culicinae	*Mansonia*	*Mansonia sp.*	305
2020	Março	23/03/2020	13	43	Jaci Paraná	P06	Culicidae	Culicinae	*Aedeomyia*	*Adeomyia squamipennis*	2
2020	Março	23/03/2020	13	43	Jaci Paraná	P06	Culicidae	Culicinae	*Aedes*	*Aedes sp.*	9
2020	Março	23/03/2020	13	43	Jaci Paraná	P06	Culicidae	Culicinae	*Culex*	*Culex sp.*	12
2020	Março	23/03/2020	13	43	Jaci Paraná	P06	Culicidae	Culicinae	*Mansonia*	*Mansonia sp.*	7
2020	Março	23/03/2020	13	43	Santa Rita	P05	Culicidae	Culicinae	*Culex*	*Culex sp.*	12
2020	Março	23/03/2020	13	43	Santa Rita	P05	Culicidae	Culicinae	*Mansonia*	*Mansonia sp.*	33
2020	Março	23/03/2020	13	43	Morrinhos	P04	Culicidae	Culicinae	*Culex*	*Culex sp.*	21
2020	Março	23/03/2020	13	43	Morrinhos	P04	Culicidae	Culicinae	*Mansonia*	*Mansonia sp.*	53
2020	Março	23/03/2020	13	43	Teotônio	P03	Culicidae	Culicinae	*Coquillettidia*	*Coquillettidia sp.*	12
2020	Março	23/03/2020	13	43	Teotônio	P03	Culicidae	Culicinae	*Culex*	*Culex sp.*	6
2020	Março	23/03/2020	13	43	Teotônio	P03	Culicidae	Culicinae	*Mansonia*	*Mansonia sp.*	15
2020	Março	23/03/2020	13	43	São Domingos	P02	Culicidae	Culicinae	*Aedeomyia*	*Adeomyia squamipennis*	3
2020	Março	23/03/2020	13	43	São Domingos	P02	Culicidae	Culicinae	*Culex*	*Culex sp.*	15
2020	Março	23/03/2020	13	43	São Domingos	P02	Culicidae	Culicinae	*Mansonia*	*Mansonia sp.*	37
2020	Março	23/03/2020	13	43	Cujubim Grande	P01	Culicidae	Culicinae	*Culex*	*Culex sp.*	3
2020	Março	23/03/2020	13	43	Cujubim Grande	P01	Culicidae	Culicinae	*Mansonia*	*Mansonia sp.*	8
2020	Março	30/03/2020	14	44	Rio Contra	P07	Culicidae	Culicinae	*Culex*	*Culex sp.*	12
2020	Março	30/03/2020	14	44	Rio Contra	P07	Culicidae	Culicinae	*Limatus*	*Limatus durhamii*	2
2020	Março	30/03/2020	14	44	Rio Contra	P07	Culicidae	Culicinae	*Mansonia*	*Mansonia sp.*	23
2020	Março	30/03/2020	14	44	Samauma	P08	Culicidae	Culicinae	*Coquillettidia*	*Coquillettidia sp.*	23
2020	Março	30/03/2020	14	44	Samauma	P08	Culicidae	Culicinae	*Culex*	*Culex sp.*	52
2020	Março	30/03/2020	14	44	Samauma	P08	Culicidae	Culicinae	*Mansonia*	*Mansonia sp.*	472
2020	Março	30/03/2020	14	44	Jaci Paraná	P06	Culicidae	Culicinae	*Aedes*	*Aedes aegypti*	11
2020	Março	30/03/2020	14	44	Jaci Paraná	P06	Culicidae	Culicinae	*Culex*	*Culex sp.*	13
2020	Março	30/03/2020	14	44	Jaci Paraná	P06	Culicidae	Culicinae	*Mansonia*	*Mansonia sp.*	8
2020	Março	30/03/2020	14	44	Santa Rita	P05	Culicidae	Culicinae	*Aedeomyia*	*Adeomyia squamipennis*	1
2020	Março	30/03/2020	14	44	Santa Rita	P05	Culicidae	Culicinae	*Culex*	*Culex sp.*	2
2020	Março	30/03/2020	14	44	Santa Rita	P05	Culicidae	Culicinae	*Mansonia*	*Mansonia sp.*	4
2020	Março	30/03/2020	14	44	Morrinhos	P04	Culicidae	Culicinae	*Culex*	*Culex sp.*	12
2020	Março	30/03/2020	14	44	Morrinhos	P04	Culicidae	Culicinae	*Mansonia*	*Mansonia sp.*	82
2020	Março	30/03/2020	14	44	Teotônio	P03	Culicidae	Culicinae	*Coquillettidia*	*Coquillettidia sp.*	10
2020	Março	30/03/2020	14	44	Teotônio	P03	Culicidae	Culicinae	*Culex*	*Culex sp.*	7
2020	Março	30/03/2020	14	44	Teotônio	P03	Culicidae	Culicinae	*Mansonia*	*Mansonia sp.*	18
2020	Março	30/03/2020	14	44	São Domingos	P02	Culicidae	Culicinae	*Aedeomyia*	*Adeomyia squamipennis*	3
2020	Março	30/03/2020	14	44	São Domingos	P02	Culicidae	Culicinae	*Culex*	*Culex sp.*	17
2020	Março	30/03/2020	14	44	São Domingos	P02	Culicidae	Culicinae	*Mansonia*	*Mansonia sp.*	42
2020	Março	30/03/2020	14	44	Cujubim Grande	P01	Culicidae	Culicinae	*Culex*	*Culex sp.*	12
2020	Março	30/03/2020	14	44	Cujubim Grande	P01	Culicidae	Culicinae	*Mansonia*	*Mansonia sp.*	5
2020	Abril	06/04/2020	15	45	Rio Contra	P07	Culicidae	Culicinae	*Aedeomyia*	*Adeomyia squamipennis*	4
2020	Abril	06/04/2020	15	45	Rio Contra	P07	Culicidae	Culicinae	*Mansonia*	*Mansonia sp.*	23
2020	Abril	06/04/2020	15	45	Samauma	P08	Culicidae	Culicinae	*Aedeomyia*	*Adeomyia squamipennis*	13
2020	Abril	06/04/2020	15	45	Samauma	P08	Culicidae	Culicinae	*Aedes*	*Aedes sp.*	4
2020	Abril	06/04/2020	15	45	Samauma	P08	Culicidae	Culicinae	*Coquillettidia*	*Coquillettidia sp.*	10
2020	Abril	06/04/2020	15	45	Samauma	P08	Culicidae	Culicinae	*Culex*	*Culex sp.*	23
2020	Abril	06/04/2020	15	45	Samauma	P08	Culicidae	Culicinae	*Mansonia*	*Mansonia sp.*	813
2020	Abril	06/04/2020	15	45	Jaci Paraná	P06	Culicidae	Culicinae	*Aedes*	*Aedes aegypti*	12
2020	Abril	06/04/2020	15	45	Jaci Paraná	P06	Culicidae	Culicinae	*Culex*	*Culex sp.*	8
2020	Abril	06/04/2020	15	45	Jaci Paraná	P06	Culicidae	Culicinae	*Mansonia*	*Mansonia sp.*	3
2020	Abril	06/04/2020	15	45	Santa Rita	P05	Culicidae	Culicinae	*Culex*	*Culex sp.*	15
2020	Abril	06/04/2020	15	45	Santa Rita	P05	Culicidae	Culicinae	*Mansonia*	*Mansonia sp.*	53
2020	Abril	06/04/2020	15	45	Morrinhos	P04	Culicidae	Culicinae	*Culex*	*Culex sp.*	23
2020	Abril	06/04/2020	15	45	Morrinhos	P04	Culicidae	Culicinae	*Mansonia*	*Mansonia sp.*	103
2020	Abril	06/04/2020	15	45	Teotônio	P03	Culicidae	Culicinae	*Coquillettidia*	*Coquillettidia sp.*	8
2020	Abril	06/04/2020	15	45	Teotônio	P03	Culicidae	Culicinae	*Culex*	*Culex sp.*	5
2020	Abril	06/04/2020	15	45	Teotônio	P03	Culicidae	Culicinae	*Mansonia*	*Mansonia sp.*	13
2020	Abril	06/04/2020	15	45	São Domingos	P02	Culicidae	Culicinae	*Culex*	*Culex sp.*	37
2020	Abril	06/04/2020	15	45	São Domingos	P02	Culicidae	Culicinae	*Mansonia*	*Mansonia sp.*	5
2020	Abril	06/04/2020	15	45	Cujubim Grande	P01	Culicidae	Culicinae	*Culex*	*Culex sp.*	10
2020	Abril	13/04/2020	16	46	Rio Contra	P07	Culicidae	Culicinae	*Mansonia*	*Mansonia sp.*	55
2020	Abril	13/04/2020	16	46	Samauma	P08	Culicidae	Culicinae	*Aedeomyia*	*Adeomyia squamipennis*	10
2020	Abril	13/04/2020	16	46	Samauma	P08	Culicidae	Culicinae	*Aedes*	*Aedes sp.*	5
2020	Abril	13/04/2020	16	46	Samauma	P08	Culicidae	Culicinae	*Culex*	*Culex sp.*	33
2020	Abril	13/04/2020	16	46	Samauma	P08	Culicidae	Culicinae	*Mansonia*	*Mansonia sp.*	917
2020	Abril	13/04/2020	16	46	Jaci Paraná	P06	Culicidae	Culicinae	*Aedes*	*Aedes aegypti*	22
2020	Abril	13/04/2020	16	46	Jaci Paraná	P06	Culicidae	Culicinae	*Culex*	*Culex sp.*	25
2020	Abril	13/04/2020	16	46	Jaci Paraná	P06	Culicidae	Culicinae	*Mansonia*	*Mansonia sp.*	12
2020	Abril	13/04/2020	16	46	Santa Rita	P05	Culicidae	Culicinae	*Culex*	*Culex sp.*	15
2020	Abril	13/04/2020	16	46	Santa Rita	P05	Culicidae	Culicinae	*Mansonia*	*Mansonia sp.*	28
2020	Abril	13/04/2020	16	46	Morrinhos	P04	Culicidae	Culicinae	*Culex*	*Culex sp.*	32
2020	Abril	13/04/2020	16	46	Morrinhos	P04	Culicidae	Culicinae	*Mansonia*	*Mansonia sp.*	111
2020	Abril	13/04/2020	16	46	Teotônio	P03	Culicidae	Culicinae	*Coquillettidia*	*Coquillettidia sp.*	5
2020	Abril	13/04/2020	16	46	Teotônio	P03	Culicidae	Culicinae	*Culex*	*Culex sp.*	8
2020	Abril	13/04/2020	16	46	Teotônio	P03	Culicidae	Culicinae	*Mansonia*	*Mansonia sp.*	12
2020	Abril	13/04/2020	16	46	São Domingos	P02	Culicidae	Culicinae	*Culex*	*Culex sp.*	52
2020	Abril	13/04/2020	16	46	São Domingos	P02	Culicidae	Culicinae	*Mansonia*	*Mansonia sp.*	5
2020	Abril	13/04/2020	16	46	Cujubim Grande	P01	Culicidae	Culicinae	*Culex*	*Culex sp.*	21
2020	Abril	20/04/2020	17	47	Rio Contra	P07	Culicidae	Culicinae	*Mansonia*	*Mansonia sp.*	42
2020	Abril	20/04/2020	17	47	Samauma	P08	Culicidae	Culicinae	*Aedeomyia*	*Adeomyia squamipennis*	7
2020	Abril	20/04/2020	17	47	Samauma	P08	Culicidae	Culicinae	*Aedes*	*Aedes sp.*	10
2020	Abril	20/04/2020	17	47	Samauma	P08	Culicidae	Anophelinae	*Anopheles*	*Anopheles sp.*	5
2020	Abril	20/04/2020	17	47	Samauma	P08	Culicidae	Culicinae	*Coquillettidia*	*Coquillettidia sp.*	8
2020	Abril	20/04/2020	17	47	Samauma	P08	Culicidae	Culicinae	*Culex*	*Culex sp.*	72
2020	Abril	20/04/2020	17	47	Samauma	P08	Culicidae	Culicinae	*Limatus*	*Limatus durhamii*	3
2020	Abril	20/04/2020	17	47	Samauma	P08	Culicidae	Culicinae	*Mansonia*	*Mansonia sp.*	1403
2020	Abril	20/04/2020	17	47	Jaci Paraná	P06	Culicidae	Culicinae	*Aedes*	*Aedes aegypti*	27
2020	Abril	20/04/2020	17	47	Jaci Paraná	P06	Culicidae	Culicinae	*Culex*	*Culex sp.*	12
2020	Abril	20/04/2020	17	47	Jaci Paraná	P06	Culicidae	Culicinae	*Mansonia*	*Mansonia sp.*	15
2020	Abril	20/04/2020	17	47	Santa Rita	P05	Culicidae	Culicinae	*Culex*	*Culex sp.*	22

2020	Abril	20/04/2020	17	47	Santa Rita	P05	Culicidae	Culicinae	*Mansonia*	*Mansonia sp.*	81
2020	Abril	20/04/2020	17	47	Morrinhos	P04	Culicidae	Culicinae	*Culex*	*Culex sp.*	87
2020	Abril	20/04/2020	17	47	Morrinhos	P04	Culicidae	Culicinae	*Mansonia*	*Mansonia sp.*	125
2020	Abril	20/04/2020	17	47	Teotônio	P03	Culicidae	Culicinae	*Coquillettidia*	*Coquillettidia sp.*	5
2020	Abril	20/04/2020	17	47	Teotônio	P03	Culicidae	Culicinae	*Culex*	*Culex sp.*	12
2020	Abril	20/04/2020	17	47	Teotônio	P03	Culicidae	Culicinae	*Mansonia*	*Mansonia sp.*	3
2020	Abril	20/04/2020	17	47	São Domingos	P02	Culicidae	Culicinae	*Culex*	*Culex sp.*	30
2020	Abril	20/04/2020	17	47	São Domingos	P02	Culicidae	Culicinae	*Limatus*	*Limatus durhamii*	1
2020	Abril	20/04/2020	17	47	São Domingos	P02	Culicidae	Culicinae	*Mansonia*	*Mansonia sp.*	8
2020	Abril	20/04/2020	17	47	Cujubim Grande	P01	Culicidae	Culicinae	*Culex*	*Culex sp.*	15
2020	Abril	27/04/2020	18	48	Rio Contra	P07	Culicidae	Culicinae	*Culex*	*Culex sp.*	12
2020	Abril	27/04/2020	18	48	Rio Contra	P07	Culicidae	Culicinae	*Mansonia*	*Mansonia sp.*	72
2020	Abril	27/04/2020	18	48	Samauma	P08	Culicidae	Culicinae	*Aedeomyia*	*Adeomyia squamipennis*	8
2020	Abril	27/04/2020	18	48	Samauma	P08	Culicidae	Anophelinae	*Anopheles*	*Anopheles sp.*	1
2020	Abril	27/04/2020	18	48	Samauma	P08	Culicidae	Culicinae	*Culex*	*Culex sp.*	53
2020	Abril	27/04/2020	18	48	Samauma	P08	Culicidae	Culicinae	*Limatus*	*Limatus durhamii*	3
2020	Abril	27/04/2020	18	48	Samauma	P08	Culicidae	Culicinae	*Mansonia*	*Mansonia sp.*	2008
2020	Abril	27/04/2020	18	48	Jaci Paraná	P06	Culicidae	Culicinae	*Aedes*	*Aedes aegypti*	5
2020	Abril	27/04/2020	18	48	Jaci Paraná	P06	Culicidae	Culicinae	*Culex*	*Culex sp.*	11
2020	Abril	27/04/2020	18	48	Jaci Paraná	P06	Culicidae	Culicinae	*Mansonia*	*Mansonia sp.*	17
2020	Abril	27/04/2020	18	48	Santa Rita	P05	Culicidae	Culicinae	*Aedes*	*Aedes sp.*	2
2020	Abril	27/04/2020	18	48	Santa Rita	P05	Culicidae	Culicinae	*Culex*	*Culex sp.*	18
2020	Abril	27/04/2020	18	48	Santa Rita	P05	Culicidae	Culicinae	*Mansonia*	*Mansonia sp.*	25
2020	Abril	27/04/2020	18	48	Morrinhos	P04	Culicidae	Culicinae	*Culex*	*Culex sp.*	28
2020	Abril	27/04/2020	18	48	Morrinhos	P04	Culicidae	Culicinae	*Mansonia*	*Mansonia sp.*	75
2020	Abril	27/04/2020	18	48	Teotônio	P03	Culicidae	Culicinae	*Coquillettidia*	*Coquillettidia sp.*	5
2020	Abril	27/04/2020	18	48	Teotônio	P03	Culicidae	Culicinae	*Culex*	*Culex sp.*	13
2020	Abril	27/04/2020	18	48	Teotônio	P03	Culicidae	Culicinae	*Mansonia*	*Mansonia sp.*	7
2020	Abril	27/04/2020	18	48	São Domingos	P02	Culicidae	Culicinae	*Culex*	*Culex sp.*	45
2020	Abril	27/04/2020	18	48	São Domingos	P02	Culicidae	Culicinae	*Mansonia*	*Mansonia sp.*	12
2020	Abril	27/04/2020	18	48	Cujubim Grande	P01	Culicidae	Culicinae	*Aedes*	*Aedes sp.*	2
2020	Abril	27/04/2020	18	48	Cujubim Grande	P01	Culicidae	Culicinae	*Culex*	*Culex sp.*	32
2020	Abril	27/04/2020	18	48	Cujubim Grande	P01	Culicidae	Culicinae	*Mansonia*	*Mansonia sp.*	7
2020	Maio	04/05/2020	19	49	Rio Contra	P07	Culicidae	Culicinae	*Aedeomyia*	*Adeomyia squamipennis*	12
2020	Maio	04/05/2020	19	49	Rio Contra	P07	Culicidae	Culicinae	*Coquillettidia*	*Coquillettidia sp.*	18
2020	Maio	04/05/2020	19	49	Rio Contra	P07	Culicidae	Culicinae	*Culex*	*Culex sp.*	83
2020	Maio	04/05/2020	19	49	Rio Contra	P07	Culicidae	Culicinae	*Mansonia*	*Mansonia sp.*	501
2020	Maio	04/05/2020	19	49	Samauma	P08	Culicidae	Culicinae	*Aedeomyia*	*Adeomyia squamipennis*	5
2020	Maio	04/05/2020	19	49	Samauma	P08	Culicidae	Culicinae	*Coquillettidia*	*Coquillettidia sp.*	10
2020	Maio	04/05/2020	19	49	Samauma	P08	Culicidae	Culicinae	*Culex*	*Culex sp.*	85
2020	Maio	04/05/2020	19	49	Samauma	P08	Culicidae	Culicinae	*Mansonia*	*Mansonia sp.*	302
2020	Maio	04/05/2020	19	49	Jaci Paraná	P06	Culicidae	Culicinae	*Aedes*	*Aedes aegypti*	8
2020	Maio	04/05/2020	19	49	Jaci Paraná	P06	Culicidae	Culicinae	*Mansonia*	*Mansonia sp.*	13
2020	Maio	04/05/2020	19	49	Santa Rita	P05	Culicidae	Culicinae	*Culex*	*Culex sp.*	5
2020	Maio	04/05/2020	19	49	Santa Rita	P05	Culicidae	Culicinae	*Mansonia*	*Mansonia sp.*	72
2020	Maio	04/05/2020	19	49	Morrinhos	P04	Culicidae	Culicinae	*Coquillettidia*	*Coquillettidia sp.*	2
2020	Maio	04/05/2020	19	49	Morrinhos	P04	Culicidae	Culicinae	*Mansonia*	*Mansonia sp.*	102
2020	Maio	04/05/2020	19	49	Teotônio	P03	Culicidae	Culicinae	*Culex*	*Culex sp.*	7
2020	Maio	04/05/2020	19	49	Teotônio	P03	Culicidae	Culicinae	*Mansonia*	*Mansonia sp.*	37
2020	Maio	04/05/2020	19	49	São Domingos	P02	Culicidae	Culicinae	*Mansonia*	*Mansonia sp.*	23
2020	Maio	04/05/2020	19	49	Cujubim Grande	P01	Culicidae	Culicinae	*Culex*	*Culex sp.*	35
2020	Maio	04/05/2020	19	49	Cujubim Grande	P01	Culicidae	Culicinae	*Mansonia*	*Mansonia sp.*	12
2020	Maio	11/05/2020	20	50	Rio Contra	P07	Culicidae	Culicinae	*Aedeomyia*	*Adeomyia squamipennis*	1
2020	Maio	11/05/2020	20	50	Rio Contra	P07	Culicidae	Culicinae	*Coquillettidia*	*Coquillettidia venezuelensis*	12
2020	Maio	11/05/2020	20	50	Rio Contra	P07	Culicidae	Culicinae	*Culex*	*Culex sp.*	23
2020	Maio	11/05/2020	20	50	Rio Contra	P07	Culicidae	Culicinae	*Mansonia*	*Mansonia sp.*	345
2020	Maio	11/05/2020	20	50	Samauma	P08	Culicidae	Culicinae	*Aedeomyia*	*Adeomyia squamipennis*	3
2020	Maio	11/05/2020	20	50	Samauma	P08	Culicidae	Culicinae	*Coquillettidia*	*Coquillettidia sp.*	11
2020	Maio	11/05/2020	20	50	Samauma	P08	Culicidae	Culicinae	*Culex*	*Culex sp.*	15
2020	Maio	11/05/2020	20	50	Samauma	P08	Culicidae	Culicinae	*Mansonia*	*Mansonia sp.*	121
2020	Maio	11/05/2020	20	50	Jaci Paraná	P06	Culicidae	Culicinae	*Aedes*	*Aedes aegypti*	5
2020	Maio	11/05/2020	20	50	Jaci Paraná	P06	Culicidae	Culicinae	*Mansonia*	*Mansonia sp.*	28
2020	Maio	11/05/2020	20	50	Santa Rita	P05	Culicidae	Culicinae	*Culex*	*Culex sp.*	3
2020	Maio	11/05/2020	20	50	Santa Rita	P05	Culicidae	Culicinae	*Mansonia*	*Mansonia sp.*	23
2020	Maio	11/05/2020	20	50	Morrinhos	P04	Culicidae	Culicinae	*Culex*	*Culex sp.*	13
2020	Maio	11/05/2020	20	50	Morrinhos	P04	Culicidae	Culicinae	*Mansonia*	*Mansonia sp.*	76
2020	Maio	11/05/2020	20	50	Teotônio	P03	Culicidae	Culicinae	*Coquillettidia*	*Coquillettidia sp.*	2
2020	Maio	11/05/2020	20	50	Teotônio	P03	Culicidae	Culicinae	*Mansonia*	*Mansonia sp.*	13
2020	Maio	11/05/2020	20	50	São Domingos	P02	Culicidae	Culicinae	*Culex*	*Culex sp.*	9
2020	Maio	11/05/2020	20	50	São Domingos	P02	Culicidae	Culicinae	*Mansonia*	*Mansonia sp.*	21
2020	Maio	11/05/2020	20	50	Cujubim Grande	P01	Culicidae	Culicinae	*Culex*	*Culex sp.*	15
2020	Maio	11/05/2020	20	50	Cujubim Grande	P01	Culicidae	Culicinae	*Mansonia*	*Mansonia sp.*	8
2020	Maio	18/05/2020	21	51	Rio Contra	P07	Culicidae	Culicinae	*Culex*	*Culex sp.*	45
2020	Maio	18/05/2020	21	51	Rio Contra	P07	Culicidae	Culicinae	*Limatus*	*Limatus durhamii*	3
2020	Maio	18/05/2020	21	51	Rio Contra	P07	Culicidae	Culicinae	*Mansonia*	*Mansonia sp.*	733
2020	Maio	18/05/2020	21	51	Samauma	P08	Culicidae	Culicinae	*Culex*	*Culex sp.*	12
2020	Maio	18/05/2020	21	51	Samauma	P08	Culicidae	Culicinae	*Mansonia*	*Mansonia sp.*	35
2020	Maio	18/05/2020	21	51	Jaci Paraná	P06	Culicidae	Culicinae	*Aedes*	*Aedes aegypti*	8
2020	Maio	18/05/2020	21	51	Jaci Paraná	P06	Culicidae	Culicinae	*Mansonia*	*Mansonia sp.*	25
2020	Maio	18/05/2020	21	51	Santa Rita	P05	Culicidae	Culicinae	*Culex*	*Culex sp.*	2
2020	Maio	18/05/2020	21	51	Santa Rita	P05	Culicidae	Culicinae	*Mansonia*	*Mansonia sp.*	5
2020	Maio	18/05/2020	21	51	Morrinhos	P04	Culicidae	Culicinae	*Culex*	*Culex sp.*	12
2020	Maio	18/05/2020	21	51	Morrinhos	P04	Culicidae	Culicinae	*Mansonia*	*Mansonia sp.*	86
2020	Maio	18/05/2020	21	51	Teotônio	P03	Culicidae	Culicinae	*Coquillettidia*	*Coquillettidia sp.*	5
2020	Maio	18/05/2020	21	51	Teotônio	P03	Culicidae	Culicinae	*Mansonia*	*Mansonia sp.*	10
2020	Maio	18/05/2020	21	51	São Domingos	P02	Culicidae	Culicinae	*Culex*	*Culex sp.*	22
2020	Maio	18/05/2020	21	51	São Domingos	P02	Culicidae	Culicinae	*Mansonia*	*Mansonia sp.*	9
2020	Maio	18/05/2020	21	51	Cujubim Grande	P01	Culicidae	Culicinae	*Culex*	*Culex sp.*	2
2020	Maio	18/05/2020	21	51	Cujubim Grande	P01	Culicidae	Culicinae	*Mansonia*	*Mansonia sp.*	8
2020	Maio	25/05/2020	22	52	Rio Contra	P07	Culicidae	Culicinae	*Coquillettidia*	*Coquillettidia sp.*	12
2020	Maio	25/05/2020	22	52	Rio Contra	P07	Culicidae	Culicinae	*Culex*	*Culex sp.*	33
2020	Maio	25/05/2020	22	52	Rio Contra	P07	Culicidae	Culicinae	*Mansonia*	*Mansonia sp.*	723
2020	Maio	25/05/2020	22	52	Samauma	P08	Culicidae	Culicinae	*Aedeomyia*	*Adeomyia squamipennis*	3
2020	Maio	25/05/2020	22	52	Samauma	P08	Culicidae	Culicinae	*Culex*	*Culex sp.*	8
2020	Maio	25/05/2020	22	52	Samauma	P08	Culicidae	Culicinae	*Mansonia*	*Mansonia sp.*	102
2020	Maio	25/05/2020	22	52	Jaci Paraná	P06	Culicidae	Culicinae	*Aedes*	*Aedes aegypti*	7
2020	Maio	25/05/2020	22	52	Jaci Paraná	P06	Culicidae	Culicinae	*Culex*	*Culex sp.*	5
2020	Maio	25/05/2020	22	52	Jaci Paraná	P06	Culicidae	Culicinae	*Mansonia*	*Mansonia sp.*	17
2020	Maio	25/05/2020	22	52	Santa Rita	P05	Culicidae	Culicinae	*Culex*	*Culex sp.*	6
2020	Maio	25/05/2020	22	52	Santa Rita	P05	Culicidae	Culicinae	*Mansonia*	*Mansonia sp.*	3
2020	Maio	25/05/2020	22	52	Morrinhos	P04	Culicidae	Culicinae	*Coquillettidia*	*Coquillettidia sp.*	1
2020	Maio	25/05/2020	22	52	Morrinhos	P04	Culicidae	Culicinae	*Mansonia*	*Mansonia sp.*	58
2020	Maio	25/05/2020	22	52	Teotônio	P03	Culicidae	Culicinae	*Culex*	*Culex sp.*	12
2020	Maio	25/05/2020	22	52	Teotônio	P03	Culicidae	Culicinae	*Mansonia*	*Mansonia sp.*	5
2020	Maio	25/05/2020	22	52	São Domingos	P02	Culicidae	Culicinae	*Coquillettidia*	*Coquillettidia sp.*	3
2020	Maio	25/05/2020	22	52	São Domingos	P02	Culicidae	Culicinae	*Culex*	*Culex sp.*	12
2020	Maio	25/05/2020	22	52	Cujubim Grande	P01	Culicidae	Culicinae	*Culex*	*Culex sp.*	5
2020	Junho	01/06/2020	23	53	Rio Contra	P07	Culicidae	Culicinae	*Mansonia*	*Mansonia sp.*	10
2020	Junho	01/06/2020	23	53	Rio Contra	P07	Culicidae	Culicinae	*Culex*	*Culex sp.*	6
2020	Junho	01/06/2020	23	53	Samauma	P08	Culicidae	Culicinae	*Mansonia*	*Mansonia sp.*	102
2020	Junho	01/06/2020	23	53	Samauma	P08	Culicidae	Culicinae	*Culex*	*Culex sp.*	15
2020	Junho	01/06/2020	23	53	Samauma	P08	Culicidae	Culicinae	*Aedeomyia*	*Adeomyia squamipennis*	3
2020	Junho	01/06/2020	23	53	Jaci Paraná	P06	Culicidae	Culicinae	*Mansonia*	*Mansonia sp.*	17
2020	Junho	01/06/2020	23	53	Jaci Paraná	P06	Culicidae	Culicinae	*Culex*	*Culex sp.*	4
2020	Junho	01/06/2020	23	53	Santa Rita	P05	Culicidae	Culicinae	*Mansonia*	*Mansonia sp.*	12
2020	Junho	01/06/2020	23	53	Santa Rita	P05	Culicidae	Culicinae	*Culex*	*Culex sp.*	15
2020	Junho	01/06/2020	23	53	Morrinhos	P04	Culicidae	Culicinae	*Mansonia*	*Mansonia sp.*	73
2020	Junho	01/06/2020	23	53	Morrinhos	P04	Culicidae	Culicinae	*Culex*	*Culex sp.*	13

2020	Junho	01/06/2020	23	53	Teotônio	P03	Culicidae	Culicinae	*Mansonia*	*Mansonia sp.*	3
2020	Junho	01/06/2020	23	53	Teotônio	P03	Culicidae	Culicinae	*Coquillettidia*	*Coquillettidia sp.*	3
2020	Junho	01/06/2020	23	53	São Domingos	P02	Culicidae	Culicinae	*Mansonia*	*Mansonia sp.*	12
2020	Junho	01/06/2020	23	53	São Domingos	P02	Culicidae	Culicinae	*Culex*	*Culex sp.*	5
2020	Junho	01/06/2020	23	53	Cujubim Grande	P01	Culicidae	Culicinae	*Mansonia*	*Mansonia sp.*	5
2020	Junho	01/06/2020	23	53	Cujubim Grande	P01	Culicidae	Culicinae	*Culex*	*Culex sp.*	2
2020	Junho	15/06/2020	25	55	Rio Contra	P07	Culicidae	Anophelinae	*Anopheles*	*Anopheles sp.*	1
2020	Junho	15/06/2020	25	55	Rio Contra	P07	Culicidae	Culicinae	*Culex*	*Culex sp.*	3
2020	Junho	15/06/2020	25	55	Rio Contra	P07	Culicidae	Culicinae	*Mansonia*	*Mansonia sp.*	6
2020	Junho	15/06/2020	25	55	Samauma	P08	Culicidae	Culicinae	*Culex*	*Culex sp.*	12
2020	Junho	15/06/2020	25	55	Samauma	P08	Culicidae	Culicinae	*Limatus*	*Limatus durhamii*	3
2020	Junho	15/06/2020	25	55	Samauma	P08	Culicidae	Culicinae	*Mansonia*	*Mansonia sp.*	28
2020	Junho	15/06/2020	25	55	Jaci Paraná	P06	Culicidae	Culicinae	*Aedes*	*Aedes aegypti*	8
2020	Junho	15/06/2020	25	55	Jaci Paraná	P06	Culicidae	Culicinae	*Culex*	*Culex sp.*	3
2020	Junho	15/06/2020	25	55	Jaci Paraná	P06	Culicidae	Culicinae	*Mansonia*	*Mansonia sp.*	5
2020	Junho	15/06/2020	25	55	Santa Rita	P05	Culicidae	Culicinae	*Culex*	*Culex sp.*	8
2020	Junho	15/06/2020	25	55	Santa Rita	P05	Culicidae	Culicinae	*Mansonia*	*Mansonia sp.*	5
2020	Junho	15/06/2020	25	55	Morrinhos	P04	Culicidae	Culicinae	*Culex*	*Culex sp.*	8
2020	Junho	15/06/2020	25	55	Morrinhos	P04	Culicidae	Culicinae	*Mansonia*	*Mansonia sp.*	33
2020	Junho	15/06/2020	25	55	Teotônio	P03	Culicidae	Culicinae	*Coquillettidia*	*Coquillettidia sp.*	3
2020	Junho	15/06/2020	25	55	Teotônio	P03	Culicidae	Culicinae	*Mansonia*	*Mansonia sp.*	1
2020	Junho	15/06/2020	25	55	São Domingos	P02	Culicidae	Culicinae	*Aedeomyia*	*Adeomyia squamipennis*	3
2020	Junho	15/06/2020	25	55	São Domingos	P02	Culicidae	Culicinae	*Culex*	*Culex sp.*	6
2020	Junho	15/06/2020	25	55	São Domingos	P02	Culicidae	Culicinae	*Mansonia*	*Mansonia sp.*	18
2020	Junho	15/06/2020	25	55	Cujubim Grande	P01	Culicidae	Culicinae	*Culex*	*Culex sp.*	10
2020	Junho	15/06/2020	25	55	Cujubim Grande	P01	Culicidae	Culicinae	*Mansonia*	*Mansonia sp.*	3
2020	Junho	22/06/2020	26	56	Morrinhos	P04	Culicidae	Culicinae	*Mansonia*	*Mansonia sp.*	39
2020	Junho	22/06/2020	26	56	Morrinhos	P04	Culicidae	Culicinae	*Culex*	*Culex sp.*	13
2020	Junho	22/06/2020	26	56	Morrinhos	P04	Culicidae	Culicinae	*Aedeomyia*	*Adeomyia squamipennis*	1
2020	Junho	22/06/2020	26	56	Santa Rita	P05	Culicidae	Culicinae	*Mansonia*	*Mansonia humeralis*	9
2020	Junho	22/06/2020	26	56	Santa Rita	P05	Culicidae	Culicinae	*Culex*	*Culex sp.*	6
2020	Junho	22/06/2020	26	56	Teotônio	P03	Culicidae	Culicinae	*Mansonia*	*Mansonia sp.*	5
2020	Junho	22/06/2020	26	56	Teotônio	P03	Culicidae	Culicinae	*Culex*	*Culex sp.*	8
2020	Junho	22/06/2020	26	56	Teotônio	P03	Culicidae	Culicinae	*Coquillettidia*	*Coquillettidia sp.*	3
2020	Junho	22/06/2020	26	56	Teotônio	P03	Culicidae	Anophelinae	*Anopheles*	*Anopheles sp.*	1
2020	Junho	22/06/2020	26	56	Teotônio	P03	Culicidae	Culicinae	*Wyeomyia*	*Wyeomyia sp.*	1
2020	Junho	22/06/2020	26	56	Cujubim Grande	P01	Culicidae	Culicinae	*Mansonia*	*Mansonia sp.*	5
2020	Junho	22/06/2020	26	56	Cujubim Grande	P01	Culicidae	Culicinae	*Culex*	*Culex sp.*	5
2020	Junho	22/06/2020	26	56	São Domingos	P02	Culicidae	Culicinae	*Mansonia*	*Mansonia sp.*	16
2020	Junho	22/06/2020	26	56	São Domingos	P02	Culicidae	Culicinae	*Culex*	*Culex sp.*	5
2020	Junho	22/06/2020	26	56	São Domingos	P02	Culicidae	Culicinae	*Aedeomyia*	*Adeomyia squamipennis*	1
2020	Junho	22/06/2020	26	56	Rio Contra	P07	Culicidae	Culicinae	*Mansonia*	*Mansonia sp.*	3
2020	Junho	22/06/2020	26	56	Rio Contra	P07	Culicidae	Culicinae	*Culex*	*Culex sp.*	4
2020	Junho	22/06/2020	26	56	Rio Contra	P07	Culicidae	Anophelinae	*Anopheles*	*Anopheles darlingi*	2
2020	Junho	22/06/2020	26	56	Rio Contra	P07	Culicidae	Anophelinae	*Anopheles*	*Anopheles sp.*	3
2020	Junho	22/06/2020	26	56	Samauma	P08	Culicidae	Culicinae	*Mansonia*	*Mansonia sp.*	23
2020	Junho	22/06/2020	26	56	Samauma	P08	Culicidae	Culicinae	*Mansonia*	*Mansonia sp.*	6
2020	Junho	22/06/2020	26	56	Samauma	P08	Culicidae	Culicinae	*Culex*	*Culex sp.*	17
2020	Junho	22/06/2020	26	56	Samauma	P08	Culicidae	Culicinae	*Uranotaenia*	*Uranotaenia geometrica*	3
2020	Junho	22/06/2020	26	56	Samauma	P08	Culicidae	Culicinae	*Limatus*	*Limatus durhamii*	1
2020	Junho	29/06/2020	27	57	Rio Contra	P07	Culicidae	Culicinae	*Aedeomyia*	*Adeomyia squamipennis*	1
2020	Junho	29/06/2020	27	57	Rio Contra	P07	Culicidae	Culicinae	*Culex*	*Culex sp.*	3
2020	Junho	29/06/2020	27	57	Rio Contra	P07	Culicidae	Culicinae	*Mansonia*	*Mansonia sp.*	23
2020	Junho	29/06/2020	27	57	Samauma	P08	Culicidae	Culicinae	*Culex*	*Culex sp.*	12
2020	Junho	29/06/2020	27	57	Samauma	P08	Culicidae	Culicinae	*Mansonia*	*Mansonia sp.*	104
2020	Junho	29/06/2020	27	57	Jaci Paraná	P06	Culicidae	Culicinae	*Aedes*	*Aedes aegypti*	7
2020	Junho	29/06/2020	27	57	Jaci Paraná	P06	Culicidae	Culicinae	*Culex*	*Culex sp.*	11
2020	Junho	29/06/2020	27	57	Jaci Paraná	P06	Culicidae	Culicinae	*Mansonia*	*Mansonia sp.*	5
2020	Junho	29/06/2020	27	57	Santa Rita	P05	Culicidae	Culicinae	*Culex*	*Culex sp.*	2
2020	Junho	29/06/2020	27	57	Santa Rita	P05	Culicidae	Culicinae	*Mansonia*	*Mansonia sp.*	3
2020	Junho	29/06/2020	27	57	Morrinhos	P04	Culicidae	Culicinae	*Culex*	*Culex sp.*	12
2020	Junho	29/06/2020	27	57	Morrinhos	P04	Culicidae	Culicinae	*Mansonia*	*Mansonia sp.*	87
2020	Junho	29/06/2020	27	57	Teotônio	P03	Culicidae	Culicinae	*Coquillettidia*	*Coquillettidia sp.*	12
2020	Junho	29/06/2020	27	57	Teotônio	P03	Culicidae	Culicinae	*Culex*	*Culex sp.*	4
2020	Junho	29/06/2020	27	57	Teotônio	P03	Culicidae	Culicinae	*Mansonia*	*Mansonia sp.*	5
2020	Junho	29/06/2020	27	57	São Domingos	P02	Culicidae	Culicinae	*Aedeomyia*	*Adeomyia squamipennis*	6
2020	Junho	29/06/2020	27	57	São Domingos	P02	Culicidae	Culicinae	*Coquillettidia*	*Coquillettidia sp.*	15
2020	Junho	29/06/2020	27	57	São Domingos	P02	Culicidae	Culicinae	*Culex*	*Culex sp.*	12
2020	Junho	29/06/2020	27	57	São Domingos	P02	Culicidae	Culicinae	*Mansonia*	*Mansonia sp.*	75
2020	Junho	29/06/2020	27	57	Cujubim Grande	P01	Culicidae	Culicinae	*Coquillettidia*	*Coquillettidia sp.*	3
2020	Junho	29/06/2020	27	57	Cujubim Grande	P01	Culicidae	Culicinae	*Culex*	*Culex sp.*	5
2020	Junho	29/06/2020	27	57	Cujubim Grande	P01	Culicidae	Culicinae	*Mansonia*	*Mansonia sp.*	13
2020	Julho	06/07/2020	28	58	Rio Contra	P07	Culicidae	Culicinae	*Aedeomyia*	*Adeomyia squamipennis*	3
2020	Julho	06/07/2020	28	58	Rio Contra	P07	Culicidae	Culicinae	*Culex*	*Culex sp.*	12
2020	Julho	06/07/2020	28	58	Rio Contra	P07	Culicidae	Culicinae	*Mansonia*	*Mansonia sp.*	53
2020	Julho	06/07/2020	28	58	Samauma	P08	Culicidae	Culicinae	*Culex*	*Culex sp.*	18
2020	Julho	06/07/2020	28	58	Samauma	P08	Culicidae	Culicinae	*Mansonia*	*Mansonia sp.*	72
2020	Julho	06/07/2020	28	58	Jaci Paraná	P06	Culicidae	Culicinae	*Aedes*	*Aedes aegypti*	2
2020	Julho	06/07/2020	28	58	Jaci Paraná	P06	Culicidae	Culicinae	*Mansonia*	*Mansonia sp.*	3
2020	Julho	06/07/2020	28	58	Santa Rita	P05	Culicidae	Culicinae	*Culex*	*Culex sp.*	5
2020	Julho	06/07/2020	28	58	Santa Rita	P05	Culicidae	Culicinae	*Mansonia*	*Mansonia sp.*	10
2020	Julho	06/07/2020	28	58	Morrinhos	P04	Culicidae	Culicinae	*Culex*	*Culex sp.*	6
2020	Julho	06/07/2020	28	58	Morrinhos	P04	Culicidae	Culicinae	*Mansonia*	*Mansonia sp.*	15
2020	Julho	06/07/2020	28	58	Teotônio	P03	Culicidae	Culicinae	*Coquillettidia*	*Coquillettidia sp.*	5
2020	Julho	06/07/2020	28	58	Teotônio	P03	Culicidae	Culicinae	*Culex*	*Culex sp.*	7
2020	Julho	06/07/2020	28	58	Teotônio	P03	Culicidae	Culicinae	*Mansonia*	*Mansonia sp.*	12
2020	Julho	06/07/2020	28	58	São Domingos	P02	Culicidae	Culicinae	*Culex*	*Culex sp.*	12
2020	Julho	06/07/2020	28	58	São Domingos	P02	Culicidae	Culicinae	*Mansonia*	*Mansonia sp.*	27
2020	Julho	06/07/2020	28	58	Cujubim Grande	P01	Culicidae	Culicinae	*Culex*	*Culex sp.*	8
2020	Julho	06/07/2020	28	58	Cujubim Grande	P01	Culicidae	Culicinae	*Mansonia*	*Mansonia sp.*	17
2020	Agosto	03/08/2020	32	62	Samauma	P08	Culicidae	Culicinae	*Culex*	*Culex sp.*	12
2020	Agosto	03/08/2020	32	62	Samauma	P08	Culicidae	Culicinae	*Mansonia*	*Mansonia sp.*	10
2020	Agosto	03/08/2020	32	62	Jaci Paraná	P06	Culicidae	Culicinae	*Aedes*	*Aedes aegypti*	7
2020	Agosto	03/08/2020	32	62	Jaci Paraná	P06	Culicidae	Culicinae	*Culex*	*Culex sp.*	5
2020	Agosto	03/08/2020	32	62	Jaci Paraná	P06	Culicidae	Culicinae	*Mansonia*	*Mansonia sp.*	5
2020	Agosto	03/08/2020	32	62	Santa Rita	P05	Culicidae	Culicinae	*Mansonia*	*Mansonia sp.*	5
2020	Agosto	03/08/2020	32	62	Morrinhos	P04	Culicidae	Culicinae	*Culex*	*Culex sp.*	5
2020	Agosto	03/08/2020	32	62	Morrinhos	P04	Culicidae	Culicinae	*Mansonia*	*Mansonia sp.*	13
2020	Agosto	03/08/2020	32	62	Teotônio	P03	Culicidae	Culicinae	*Coquillettidia*	*Coquillettidia sp.*	8
2020	Agosto	03/08/2020	32	62	Teotônio	P03	Culicidae	Culicinae	*Mansonia*	*Mansonia sp.*	12
2020	Agosto	03/08/2020	32	62	São Domingos	P02	Culicidae	Culicinae	*Coquillettidia*	*Coquillettidia sp.*	2
2020	Agosto	03/08/2020	32	62	São Domingos	P02	Culicidae	Culicinae	*Culex*	*Culex sp.*	3
2020	Agosto	03/08/2020	32	62	São Domingos	P02	Culicidae	Culicinae	*Mansonia*	*Mansonia sp.*	27
2020	Agosto	10/08/2020	33	63	Rio Contra	P07	Culicidae	Culicinae	*Culex*	*Culex sp.*	3
2020	Agosto	10/08/2020	33	63	Rio Contra	P07	Culicidae	Culicinae	*Mansonia*	*Mansonia sp.*	5
2020	Agosto	10/08/2020	33	63	Samauma	P08	Culicidae	Culicinae	*Culex*	*Culex sp.*	1
2020	Agosto	10/08/2020	33	63	Samauma	P08	Culicidae	Culicinae	*Mansonia*	*Mansonia sp.*	11
2020	Agosto	10/08/2020	33	63	Jaci Paraná	P06	Culicidae	Culicinae	*Aedes*	*Aedes aegypti*	5
2020	Agosto	10/08/2020	33	63	Jaci Paraná	P06	Culicidae	Culicinae	*Coquillettidia*	*Coquillettidia sp.*	2
2020	Agosto	10/08/2020	33	63	Jaci Paraná	P06	Culicidae	Culicinae	*Mansonia*	*Mansonia sp.*	5
2020	Agosto	10/08/2020	33	63	Santa Rita	P05	Culicidae	Culicinae	*Mansonia*	*Mansonia sp.*	6
2020	Agosto	10/08/2020	33	63	Morrinhos	P04	Culicidae	Culicinae	*Mansonia*	*Mansonia sp.*	3
2020	Agosto	10/08/2020	33	63	Teotônio	P03	Culicidae	Culicinae	*Coquillettidia*	*Coquillettidia sp.*	8
2020	Agosto	10/08/2020	33	63	Teotônio	P03	Culicidae	Culicinae	*Mansonia*	*Mansonia sp.*	8
2020	Agosto	10/08/2020	33	63	São Domingos	P02	Culicidae	Culicinae	*Mansonia*	*Mansonia sp.*	48
2020	Agosto	17/08/2020	34	64	Rio Contra	P07	Culicidae	Culicinae	*Mansonia*	*Mansonia sp.*	1
2020	Agosto	17/08/2020	34	64	Samauma	P08	Culicidae	Culicinae	*Mansonia*	*Mansonia sp.*	12
2020	Agosto	17/08/2020	34	64	Jaci Paraná	P06	Culicidae	Culicinae	*Aedes*	*Aedes aegypti*	9

2020	Agosto	17/08/2020	34	64	Jaci Paraná	P06	Culicidae	Culicinae	*Mansonia*	*Mansonia sp.*	3
2020	Agosto	17/08/2020	34	64	Santa Rita	P05	Culicidae	Culicinae	*Mansonia*	*Mansonia sp.*	10
2020	Agosto	17/08/2020	34	64	Morrinhos	P04	Culicidae	Culicinae	*Mansonia*	*Mansonia sp.*	7
2020	Agosto	17/08/2020	34	64	Teotônio	P03	Culicidae	Culicinae	*Coquillettidia*	*Coquillettidia sp.*	5
2020	Agosto	17/08/2020	34	64	Teotônio	P03	Culicidae	Culicinae	*Mansonia*	*Mansonia sp.*	1
2020	Agosto	17/08/2020	34	64	São Domingos	P02	Culicidae	Culicinae	*Mansonia*	*Mansonia sp.*	53
2020	Agosto	17/08/2020	34	64	Cujubim Grande	P01	Culicidae	Culicinae	*Mansonia*	*Mansonia sp.*	5
2020	Agosto	24/08/2020	35	65	Rio Contra	P07	Culicidae	Culicinae	*Culex*	*Culex sp.*	12
2020	Agosto	24/08/2020	35	65	Rio Contra	P07	Culicidae	Culicinae	*Mansonia*	*Mansonia sp.*	23
2020	Agosto	24/08/2020	35	65	Samauma	P08	Culicidae	Culicinae	*Culex*	*Culex sp.*	72
2020	Agosto	24/08/2020	35	65	Samauma	P08	Culicidae	Culicinae	*Mansonia*	*Mansonia sp.*	25
2020	Agosto	24/08/2020	35	65	Jaci Paraná	P06	Culicidae	Culicinae	*Aedes*	*Aedes aegypti*	5
2020	Agosto	24/08/2020	35	65	Jaci Paraná	P06	Culicidae	Culicinae	*Culex*	*Culex sp.*	7
2020	Agosto	24/08/2020	35	65	Jaci Paraná	P06	Culicidae	Culicinae	*Mansonia*	*Mansonia sp.*	8
2020	Agosto	24/08/2020	35	65	Santa Rita	P05	Culicidae	Culicinae	*Mansonia*	*Mansonia sp.*	8
2020	Agosto	24/08/2020	35	65	Morrinhos	P04	Culicidae	Culicinae	*Mansonia*	*Mansonia sp.*	28
2020	Agosto	24/08/2020	35	65	Teotônio	P03	Culicidae	Culicinae	*Coquillettidia*	*Coquillettidia sp.*	12
2020	Agosto	24/08/2020	35	65	Teotônio	P03	Culicidae	Culicinae	*Mansonia*	*Mansonia sp.*	5
2020	Agosto	24/08/2020	35	65	São Domingos	P02	Culicidae	Culicinae	*Culex*	*Culex sp.*	12
2020	Agosto	24/08/2020	35	65	São Domingos	P02	Culicidae	Culicinae	*Mansonia*	*Mansonia sp.*	48
2020	Agosto	24/08/2020	35	65	Cujubim Grande	P01	Culicidae	Culicinae	*Culex*	*Culex sp.*	2
2020	Agosto	24/08/2020	35	65	Cujubim Grande	P01	Culicidae	Culicinae	*Mansonia*	*Mansonia sp.*	5
2020	Agosto	01/09/2020	36	66	Rio Contra	P07	Culicidae	Culicinae	*Culex*	*Culex sp.*	18
2020	Agosto	01/09/2020	36	66	Rio Contra	P07	Culicidae	Culicinae	*Mansonia*	*Mansonia sp.*	23
2020	Agosto	01/09/2020	36	66	Samauma	P08	Culicidae	Culicinae	*Culex*	*Culex sp.*	65
2020	Agosto	01/09/2020	36	66	Samauma	P08	Culicidae	Culicinae	*Mansonia*	*Mansonia sp.*	53
2020	Agosto	01/09/2020	36	66	Jaci Paraná	P06	Culicidae	Culicinae	*Aedes*	*Aedes aegypti*	12
2020	Agosto	01/09/2020	36	66	Jaci Paraná	P06	Culicidae	Culicinae	*Culex*	*Culex sp.*	2
2020	Agosto	01/09/2020	36	66	Jaci Paraná	P06	Culicidae	Culicinae	*Mansonia*	*Mansonia sp.*	8
2020	Agosto	01/09/2020	36	66	Santa Rita	P05	Culicidae	Culicinae	*Culex*	*Culex sp.*	23
2020	Agosto	01/09/2020	36	66	Santa Rita	P05	Culicidae	Culicinae	*Mansonia*	*Mansonia sp.*	18
2020	Agosto	01/09/2020	36	66	Morrinhos	P04	Culicidae	Culicinae	*Culex*	*Culex sp.*	17
2020	Agosto	01/09/2020	36	66	Morrinhos	P04	Culicidae	Culicinae	*Mansonia*	*Mansonia sp.*	33
2020	Agosto	01/09/2020	36	66	Teotônio	P03	Culicidae	Culicinae	*Coquillettidia*	*Coquillettidia sp.*	5
2020	Agosto	01/09/2020	36	66	Teotônio	P03	Culicidae	Culicinae	*Culex*	*Culex sp.*	12
2020	Agosto	01/09/2020	36	66	Teotônio	P03	Culicidae	Culicinae	*Mansonia*	*Mansonia sp.*	3
2020	Agosto	01/09/2020	36	66	São Domingos	P02	Culicidae	Culicinae	*Coquillettidia*	*Coquillettidia sp.*	5
2020	Agosto	01/09/2020	36	66	São Domingos	P02	Culicidae	Culicinae	*Culex*	*Culex sp.*	15
2020	Agosto	01/09/2020	36	66	São Domingos	P02	Culicidae	Culicinae	*Mansonia*	*Mansonia sp.*	55
2020	Agosto	01/09/2020	36	66	Cujubim Grande	P01	Culicidae	Culicinae	*Mansonia*	*Mansonia sp.*	11
2020	Setembro	07/09/2020	37	67	Rio Contra	P07	Culicidae	Culicinae	*Culex*	*Culex sp.*	12
2020	Setembro	07/09/2020	37	67	Rio Contra	P07	Culicidae	Culicinae	*Mansonia*	*Mansonia sp.*	13
2020	Setembro	07/09/2020	37	67	Samauma	P08	Culicidae	Culicinae	*Culex*	*Culex sp.*	9
2020	Setembro	07/09/2020	37	67	Samauma	P08	Culicidae	Culicinae	*Mansonia*	*Mansonia sp.*	72
2020	Setembro	07/09/2020	37	67	Jaci Paraná	P06	Culicidae	Culicinae	*Aedes*	*Aedes aegypti*	18
2020	Setembro	07/09/2020	37	67	Santa Rita	P05	Culicidae	Culicinae	*Mansonia*	*Mansonia sp.*	23
2020	Setembro	07/09/2020	37	67	Morrinhos	P04	Culicidae	Culicinae	*Coquillettidia*	*Coquillettidia sp.*	2
2020	Setembro	07/09/2020	37	67	Morrinhos	P04	Culicidae	Culicinae	*Mansonia*	*Mansonia sp.*	42
2020	Setembro	07/09/2020	37	67	Teotônio	P03	Culicidae	Culicinae	*Coquillettidia*	*Coquillettidia sp.*	13
2020	Setembro	07/09/2020	37	67	Teotônio	P03	Culicidae	Culicinae	*Mansonia*	*Mansonia sp.*	8
2020	Setembro	07/09/2020	37	67	São Domingos	P02	Culicidae	Culicinae	*Aedeomyia*	*Adeomyia squamipennis*	5
2020	Setembro	07/09/2020	37	67	São Domingos	P02	Culicidae	Culicinae	*Culex*	*Culex sp.*	17
2020	Setembro	07/09/2020	37	67	São Domingos	P02	Culicidae	Culicinae	*Mansonia*	*Mansonia sp.*	33
2020	Setembro	07/09/2020	37	67	Cujubim Grande	P01	Culicidae	Culicinae	*Culex*	*Culex sp.*	3
2020	Setembro	14/09/2020	38	68	Rio Contra	P07	Culicidae	Culicinae	*Mansonia*	*Mansonia sp.*	10
2020	Setembro	14/09/2020	38	68	Samauma	P08	Culicidae	Culicinae	*Aedeomyia*	*Adeomyia squamipennis*	2
2020	Setembro	14/09/2020	38	68	Samauma	P08	Culicidae	Culicinae	*Coquillettidia*	*Coquillettidia sp.*	5
2020	Setembro	14/09/2020	38	68	Samauma	P08	Culicidae	Culicinae	*Culex*	*Culex sp.*	3
2020	Setembro	14/09/2020	38	68	Samauma	P08	Culicidae	Culicinae	*Mansonia*	*Mansonia sp.*	28
2020	Setembro	14/09/2020	38	68	Jaci Paraná	P06	Culicidae	Culicinae	*Aedes*	*Aedes aegypti*	3
2020	Setembro	14/09/2020	38	68	Jaci Paraná	P06	Culicidae	Culicinae	*Culex*	*Culex sp.*	8
2020	Setembro	14/09/2020	38	68	Jaci Paraná	P06	Culicidae	Culicinae	*Mansonia*	*Mansonia sp.*	12
2020	Setembro	14/09/2020	38	68	Santa Rita	P05	Culicidae	Culicinae	*Mansonia*	*Mansonia sp.*	12
2020	Setembro	14/09/2020	38	68	Morrinhos	P04	Culicidae	Culicinae	*Mansonia*	*Mansonia sp.*	41
2020	Setembro	14/09/2020	38	68	Teotônio	P03	Culicidae	Culicinae	*Coquillettidia*	*Coquillettidia sp.*	6
2020	Setembro	14/09/2020	38	68	Teotônio	P03	Culicidae	Culicinae	*Mansonia*	*Mansonia sp.*	18
2020	Setembro	14/09/2020	38	68	São Domingos	P02	Culicidae	Culicinae	*Coquillettidia*	*Coquillettidia sp.*	5
2020	Setembro	14/09/2020	38	68	São Domingos	P02	Culicidae	Culicinae	*Mansonia*	*Mansonia sp.*	23
2020	Setembro	14/09/2020	38	68	Cujubim Grande	P01	Culicidae	Culicinae	*Culex*	*Culex sp.*	8
2020	Setembro	21/09/2020	39	69	Rio Contra	P07	Culicidae	Culicinae	*Culex*	*Culex sp.*	33
2020	Setembro	21/09/2020	39	69	Rio Contra	P07	Culicidae	Culicinae	*Limatus*	*Limatus durhamii*	5
2020	Setembro	21/09/2020	39	69	Rio Contra	P07	Culicidae	Culicinae	*Mansonia*	*Mansonia sp.*	44
2020	Setembro	21/09/2020	39	69	Samauma	P08	Culicidae	Culicinae	*Aedeomyia*	*Adeomyia squamipennis*	3
2020	Setembro	21/09/2020	39	69	Samauma	P08	Culicidae	Culicinae	*Culex*	*Culex sp.*	22
2020	Setembro	21/09/2020	39	69	Samauma	P08	Culicidae	Culicinae	*Limatus*	*Limatus durhamii*	1
2020	Setembro	21/09/2020	39	69	Samauma	P08	Culicidae	Culicinae	*Mansonia*	*Mansonia sp.*	88
2020	Setembro	21/09/2020	39	69	Jaci Paraná	P06	Culicidae	Culicinae	*Aedes*	*Aedes aegypti*	25
2020	Setembro	21/09/2020	39	69	Jaci Paraná	P06	Culicidae	Culicinae	*Culex*	*Culex sp.*	28
2020	Setembro	21/09/2020	39	69	Jaci Paraná	P06	Culicidae	Culicinae	*Mansonia*	*Mansonia sp.*	37
2020	Setembro	21/09/2020	39	69	Santa Rita	P05	Culicidae	Culicinae	*Culex*	*Culex sp.*	2
2020	Setembro	21/09/2020	39	69	Santa Rita	P05	Culicidae	Culicinae	*Mansonia*	*Mansonia sp.*	35
2020	Setembro	21/09/2020	39	69	Morrinhos	P04	Culicidae	Culicinae	*Culex*	*Culex sp.*	12
2020	Setembro	21/09/2020	39	69	Morrinhos	P04	Culicidae	Culicinae	*Mansonia*	*Mansonia sp.*	75
2020	Setembro	21/09/2020	39	69	Teotônio	P03	Culicidae	Culicinae	*Coquillettidia*	*Coquillettidia sp.*	8
2020	Setembro	21/09/2020	39	69	Teotônio	P03	Culicidae	Culicinae	*Mansonia*	*Mansonia sp.*	15
2020	Setembro	21/09/2020	39	69	São Domingos	P02	Culicidae	Culicinae	*Coquillettidia*	*Coquillettidia sp.*	5
2020	Setembro	21/09/2020	39	69	São Domingos	P02	Culicidae	Culicinae	*Culex*	*Culex sp.*	13
2020	Setembro	21/09/2020	39	69	São Domingos	P02	Culicidae	Culicinae	*Mansonia*	*Mansonia sp.*	87
2020	Setembro	21/09/2020	39	69	Cujubim Grande	P01	Culicidae	Culicinae	*Culex*	*Culex sp.*	12
2020	Setembro	21/09/2020	39	69	Cujubim Grande	P01	Culicidae	Culicinae	*Mansonia*	*Mansonia sp.*	17
2020	Setembro	28/09/2020	40	70	Rio Contra	P07	Culicidae	Culicinae	*Mansonia*	*Mansonia sp.*	7
2020	Setembro	28/09/2020	40	70	Samauma	P08	Culicidae	Culicinae	*Culex*	*Culex sp.*	15
2020	Setembro	28/09/2020	40	70	Samauma	P08	Culicidae	Culicinae	*Limatus*	*Limatus durhamii*	1
2020	Setembro	28/09/2020	40	70	Samauma	P08	Culicidae	Culicinae	*Mansonia*	*Mansonia sp.*	23
2020	Setembro	28/09/2020	40	70	Jaci Paraná	P06	Culicidae	Culicinae	*Aedes*	*Aedes aegypti*	2
2020	Setembro	28/09/2020	40	70	Jaci Paraná	P06	Culicidae	Culicinae	*Mansonia*	*Mansonia sp.*	11
2020	Setembro	28/09/2020	40	70	Santa Rita	P05	Culicidae	Culicinae	*Mansonia*	*Mansonia sp.*	21
2020	Setembro	28/09/2020	40	70	Morrinhos	P04	Culicidae	Culicinae	*Mansonia*	*Mansonia sp.*	44
2020	Setembro	28/09/2020	40	70	Teotônio	P03	Culicidae	Culicinae	*Coquillettidia*	*Coquillettidia sp.*	7
2020	Setembro	28/09/2020	40	70	Teotônio	P03	Culicidae	Culicinae	*Mansonia*	*Mansonia sp.*	7
2020	Setembro	28/09/2020	40	70	São Domingos	P02	Culicidae	Culicinae	*Culex*	*Culex sp.*	23
2020	Setembro	28/09/2020	40	70	São Domingos	P02	Culicidae	Culicinae	*Mansonia*	*Mansonia sp.*	103
2020	Setembro	28/09/2020	40	70	Cujubim Grande	P01	Culicidae	Culicinae	*Mansonia*	*Mansonia sp.*	16
2020	Outubro	05/10/2020	41	71	Rio Contra	P07	Culicidae	Culicinae	*Mansonia*	*Mansonia sp.*	10
2020	Outubro	05/10/2020	41	71	Rio Contra	P07	Culicidae	Culicinae	*Culex*	*Culex sp.*	5
2020	Outubro	05/10/2020	41	71	Rio Contra	P07	Culicidae	Culicinae	*Limatus*	*Limatus durhamii*	2
2020	Outubro	05/10/2020	41	71	Samauma	P08	Culicidae	Culicinae	*Mansonia*	*Mansonia sp.*	18
2020	Outubro	05/10/2020	41	71	Samauma	P08	Culicidae	Culicinae	*Culex*	*Culex sp.*	5
2020	Outubro	05/10/2020	41	71	Jaci Paraná	P06	Culicidae	Culicinae	*Mansonia*	*Mansonia sp.*	2
2020	Outubro	05/10/2020	41	71	Jaci Paraná	P06	Culicidae	Culicinae	*Culex*	*Culex sp.*	3
2020	Outubro	05/10/2020	41	71	Jaci Paraná	P06	Culicidae	Culicinae	*Aedes*	*Aedes aegypti*	3
2020	Outubro	05/10/2020	41	71	Santa Rita	P05	Culicidae	Culicinae	*Mansonia*	*Mansonia sp.*	10
2020	Outubro	05/10/2020	41	71	Santa Rita	P05	Culicidae	Culicinae	*Culex*	*Culex sp.*	2
2020	Outubro	05/10/2020	41	71	Morrinhos	P04	Culicidae	Culicinae	*Mansonia*	*Mansonia sp.*	24
2020	Outubro	05/10/2020	41	71	Teotônio	P03	Culicidae	Culicinae	*Mansonia*	*Mansonia sp.*	15
2020	Outubro	05/10/2020	41	71	Teotônio	P03	Culicidae	Culicinae	*Culex*	*Culex sp.*	3
2020	Outubro	05/10/2020	41	71	Teotônio	P03	Culicidae	Culicinae	*Coquillettidia*	*Coquillettidia sp.*	3
2020	Outubro	05/10/2020	41	71	São Domingos	P02	Culicidae	Culicinae	*Mansonia*	*Mansonia sp.*	28

2020	Outubro	05/10/2020	41	71	São Domingos	P02	Culicidae	Culicinae	Culex	Culex sp.	7
2020	Outubro	05/10/2020	41	71	Cujubim Grande	P01	Culicidae	Culicinae	Culex	Culex sp.	9
2020	Outubro	12/10/2020	42	72	Rio Contra	P07	Culicidae	Culicinae	Mansonia	Mansonia sp.	6
2020	Outubro	12/10/2020	42	72	Rio Contra	P07	Culicidae	Culicinae	Limatus	Limatus durhamii	1
2020	Outubro	12/10/2020	42	72	Samauma	P08	Culicidae	Culicinae	Mansonia	Mansonia sp.	21
2020	Outubro	12/10/2020	42	72	Samauma	P08	Culicidae	Culicinae	Culex	Culex sp.	12
2020	Outubro	12/10/2020	42	72	Samauma	P08	Culicidae	Culicinae	Aedes	Aedes aegypti	4
2020	Outubro	12/10/2020	42	72	Jaci Paraná	P06	Culicidae	Culicinae	Aedes	Aedes aegypti	7
2020	Outubro	12/10/2020	42	72	Jaci Paraná	P06	Culicidae	Culicinae	Culex	Culex sp.	8
2020	Outubro	12/10/2020	42	72	Jaci Paraná	P06	Culicidae	Culicinae	Mansonia	Mansonia sp.	5
2020	Outubro	12/10/2020	42	72	Santa Rita	P05	Culicidae	Culicinae	Mansonia	Mansonia sp.	11
2020	Outubro	12/10/2020	42	72	Morrinhos	P04	Culicidae	Culicinae	Mansonia	Mansonia sp.	35
2020	Outubro	12/10/2020	42	72	Teotônio	P03	Culicidae	Culicinae	Mansonia	Mansonia sp.	5
2020	Outubro	12/10/2020	42	72	Teotônio	P03	Culicidae	Culicinae	Coquillettidia	Coquillettidia sp.	2
2020	Outubro	12/10/2020	42	72	São Domingos	P02	Culicidae	Culicinae	Mansonia	Mansonia sp.	3
2020	Outubro	12/10/2020	42	72	São Domingos	P02	Culicidae	Culicinae	Culex	Culex sp.	10
2020	Outubro	12/10/2020	42	72	Cujubim Grande	P01	Culicidae	Culicinae	Mansonia	Mansonia sp.	5
2020	Outubro	12/10/2020	42	72	Cujubim Grande	P01	Culicidae	Culicinae	Culex	Culex sp.	2
2020	Outubro	19/10/2020	43	73	Rio Contra	P07	Culicidae	Culicinae	Mansonia	Mansonia sp.	3
2020	Outubro	19/10/2020	43	73	Rio Contra	P07	Culicidae	Culicinae	Culex	Culex sp.	12
2020	Outubro	19/10/2020	43	73	Samauma	P08	Culicidae	Culicinae	Mansonia	Mansonia sp.	25
2020	Outubro	19/10/2020	43	73	Samauma	P08	Culicidae	Culicinae	Culex	Culex sp.	8
2020	Outubro	19/10/2020	43	73	Jaci Paraná	P06	Culicidae	Culicinae	Aedes	Aedes aegypti	3
2020	Outubro	19/10/2020	43	73	Jaci Paraná	P06	Culicidae	Culicinae	Mansonia	Mansonia sp.	8
2020	Outubro	19/10/2020	43	73	Santa Rita	P05	Culicidae	Culicinae	Mansonia	Mansonia sp.	11
2020	Outubro	19/10/2020	43	73	Santa Rita	P05	Culicidae	Culicinae	Culex	Culex sp.	5
2020	Outubro	19/10/2020	43	73	Morrinhos	P04	Culicidae	Culicinae	Mansonia	Mansonia sp.	35
2020	Outubro	19/10/2020	43	73	Teotônio	P03	Culicidae	Culicinae	Coquillettidia	Coquillettidia sp.	3
2020	Outubro	19/10/2020	43	73	Teotônio	P03	Culicidae	Culicinae	Mansonia	Mansonia sp.	1
2020	Outubro	19/10/2020	43	73	São Domingos	P02	Culicidae	Culicinae	Mansonia	Mansonia sp.	23
2020	Outubro	19/10/2020	43	73	São Domingos	P02	Culicidae	Culicinae	Culex	Culex sp.	2
2020	Outubro	19/10/2020	43	73	São Domingos	P02	Culicidae	Culicinae	Coquillettidia	Coquillettidia sp.	1
2020	Outubro	19/10/2020	43	73	Cujubim Grande	P01	Culicidae	Culicinae	Culex	Culex sp.	13
2020	Outubro	26/10/2020	44	74	Rio Contra	P07	Culicidae	Culicinae	Mansonia	Mansonia sp.	13
2020	Outubro	26/10/2020	44	74	Rio Contra	P07	Culicidae	Culicinae	Culex	Culex sp.	8
2020	Outubro	26/10/2020	44	74	Samauma	P08	Culicidae	Culicinae	Mansonia	Mansonia sp.	19
2020	Outubro	26/10/2020	44	74	Samauma	P08	Culicidae	Culicinae	Culex	Culex sp.	12
2020	Outubro	26/10/2020	44	74	Jaci Paraná	P06	Culicidae	Culicinae	Aedes	Aedes aegypti	8
2020	Outubro	26/10/2020	44	74	Jaci Paraná	P06	Culicidae	Culicinae	Mansonia	Mansonia sp.	7
2020	Outubro	26/10/2020	44	74	Jaci Paraná	P06	Culicidae	Culicinae	Culex	Culex sp.	12
2020	Outubro	26/10/2020	44	74	Santa Rita	P05	Culicidae	Culicinae	Mansonia	Mansonia sp.	11
2020	Outubro	26/10/2020	44	74	Morrinhos	P04	Culicidae	Culicinae	Mansonia	Mansonia sp.	27
2020	Outubro	26/10/2020	44	74	Teotônio	P03	Culicidae	Culicinae	Mansonia	Mansonia sp.	8
2020	Outubro	26/10/2020	44	74	Teotônio	P03	Culicidae	Culicinae	Coquillettidia	Coquillettidia sp.	3
2020	Outubro	26/10/2020	44	74	São Domingos	P02	Culicidae	Culicinae	Mansonia	Mansonia sp.	35
2020	Outubro	26/10/2020	44	74	São Domingos	P02	Culicidae	Culicinae	Culex	Culex sp.	8
2020	Outubro	26/10/2020	44	74	Cujubim Grande	P01	Culicidae	Culicinae	Mansonia	Mansonia sp.	3
2020	Outubro	26/10/2020	44	74	Cujubim Grande	P01	Culicidae	Culicinae	Culex	Culex sp.	22
2020	Novembro	02/11/2020	45	75	Rio Contra	P07	Culicidae	Culicinae	Culex	Culex sp.	12
2020	Novembro	02/11/2020	45	75	Rio Contra	P07	Culicidae	Culicinae	Mansonia	Mansonia sp.	6
2020	Novembro	02/11/2020	45	75	Samauma	P08	Culicidae	Culicinae	Mansonia	Mansonia sp.	10
2020	Novembro	02/11/2020	45	75	Samauma	P08	Culicidae	Culicinae	Culex	Culex sp.	12
2020	Novembro	02/11/2020	45	75	Jaci Paraná	P06	Culicidae	Culicinae	Aedes	Aedes aegypti	7
2020	Novembro	02/11/2020	45	75	Jaci Paraná	P06	Culicidae	Culicinae	Culex	Culex sp.	5
2020	Novembro	02/11/2020	45	75	Jaci Paraná	P06	Culicidae	Culicinae	Mansonia	Mansonia sp.	8
2020	Novembro	02/11/2020	45	75	Santa Rita	P05	Culicidae	Culicinae	Mansonia	Mansonia sp.	5
2020	Novembro	02/11/2020	45	75	Santa Rita	P05	Culicidae	Culicinae	Culex	Culex sp.	3
2020	Novembro	02/11/2020	45	75	Morrinhos	P04	Culicidae	Culicinae	Mansonia	Mansonia sp.	13
2020	Novembro	02/11/2020	45	75	Morrinhos	P04	Culicidae	Culicinae	Culex	Culex sp.	5
2020	Novembro	02/11/2020	45	75	Teotônio	P03	Culicidae	Culicinae	Coquillettidia	Coquillettidia sp.	8
2020	Novembro	02/11/2020	45	75	Teotônio	P03	Culicidae	Culicinae	Mansonia	Mansonia sp.	12
2020	Novembro	02/11/2020	45	75	São Domingos	P02	Culicidae	Culicinae	Mansonia	Mansonia sp.	27
2020	Novembro	02/11/2020	45	75	São Domingos	P02	Culicidae	Culicinae	Culex	Culex sp.	8
2020	Novembro	02/11/2020	45	75	São Domingos	P02	Culicidae	Culicinae	Coquillettidia	Coquillettidia sp.	4
2020	Novembro	02/11/2020	45	75	Cujubim Grande	P01	Culicidae	Culicinae	Culex	Culex sp.	5
2020	Novembro	09/11/2020	46	76	Rio Contra	P07	Culicidae	Culicinae	Mansonia	Mansonia sp.	7
2020	Novembro	09/11/2020	46	76	Samauma	P08	Culicidae	Culicinae	Mansonia	Mansonia sp.	23
2020	Novembro	09/11/2020	46	76	Samauma	P08	Culicidae	Culicinae	Culex	Culex sp.	5
2020	Novembro	09/11/2020	46	76	Jaci Paraná	P06	Culicidae	Culicinae	Aedes	Aedes aegypti	5
2020	Novembro	09/11/2020	46	76	Jaci Paraná	P06	Culicidae	Culicinae	Culex	Culex sp.	2
2020	Novembro	09/11/2020	46	76	Jaci Paraná	P06	Culicidae	Culicinae	Mansonia	Mansonia sp.	1
2020	Novembro	09/11/2020	46	76	Santa Rita	P05	Culicidae	Culicinae	Mansonia	Mansonia sp.	10
2020	Novembro	09/11/2020	46	76	Santa Rita	P05	Culicidae	Culicinae	Culex	Culex sp.	5
2020	Novembro	09/11/2020	46	76	Morrinhos	P04	Culicidae	Culicinae	Mansonia	Mansonia sp.	18
2020	Novembro	09/11/2020	46	76	Teotônio	P03	Culicidae	Culicinae	Coquillettidia	Coquillettidia sp.	3
2020	Novembro	09/11/2020	46	76	Teotônio	P03	Culicidae	Culicinae	Mansonia	Mansonia sp.	2
2020	Novembro	09/11/2020	46	76	São Domingos	P02	Culicidae	Culicinae	Mansonia	Mansonia sp.	15
2020	Novembro	09/11/2020	46	76	São Domingos	P02	Culicidae	Culicinae	Culex	Culex sp.	17
2020	Novembro	09/11/2020	46	76	Cujubim Grande	P01	Culicidae	Culicinae	Mansonia	Mansonia sp.	5
2020	Novembro	09/11/2020	46	76	Cujubim Grande	P01	Culicidae	Culicinae	Culex	Culex sp.	7
2020	Novembro	16/11/2020	47	77	Rio Contra	P07	Culicidae	Culicinae	Mansonia	Mansonia sp.	15
2020	Novembro	16/11/2020	47	77	Rio Contra	P07	Culicidae	Anophelinae	Anopheles	Anopheles sp.	3
2020	Novembro	16/11/2020	47	77	Samauma	P08	Culicidae	Culicinae	Culex	Culex sp.	87
2020	Novembro	16/11/2020	47	77	Samauma	P08	Culicidae	Culicinae	Mansonia	Mansonia sp.	23
2020	Novembro	16/11/2020	47	77	Samauma	P08	Culicidae	Culicinae	Aedeomyia	Adeomyia squamipennis	7
2020	Novembro	16/11/2020	47	77	Jaci Paraná	P06	Culicidae	Culicinae	Aedes	Aedes aegypti	5
2020	Novembro	16/11/2020	47	77	Jaci Paraná	P06	Culicidae	Culicinae	Culex	Culex sp.	12
2020	Novembro	16/11/2020	47	77	Jaci Paraná	P06	Culicidae	Culicinae	Mansonia	Mansonia sp.	3
2020	Novembro	16/11/2020	47	77	Santa Rita	P05	Culicidae	Culicinae	Mansonia	Mansonia sp.	22
2020	Novembro	16/11/2020	47	77	Santa Rita	P05	Culicidae	Culicinae	Culex	Culex sp.	2
2020	Novembro	16/11/2020	47	77	Santa Rita	P05	Culicidae	Culicinae	Aedes	Aedes sp.	1
2020	Novembro	16/11/2020	47	77	Morrinhos	P04	Culicidae	Culicinae	Mansonia	Mansonia sp.	13
2020	Novembro	16/11/2020	47	77	Morrinhos	P04	Culicidae	Culicinae	Culex	Culex sp.	5
2020	Novembro	16/11/2020	47	77	São Domingos	P02	Culicidae	Culicinae	Mansonia	Mansonia sp.	20
2020	Novembro	16/11/2020	47	77	São Domingos	P02	Culicidae	Culicinae	Coquillettidia	Coquillettidia sp.	4
2020	Novembro	16/11/2020	47	77	São Domingos	P02	Culicidae	Culicinae	Culex	Culex sp.	6
2020	Novembro	16/11/2020	47	77	Cujubim Grande	P01	Culicidae	Culicinae	Mansonia	Mansonia sp.	1
2020	Novembro	16/11/2020	47	77	Cujubim Grande	P01	Culicidae	Culicinae	Culex	Culex sp.	1
2020	Novembro	23/11/2020	48	78	Rio Contra	P07	Culicidae	Culicinae	Culex	Culex sp.	2
2020	Novembro	23/11/2020	48	78	Rio Contra	P07	Culicidae	Culicinae	Mansonia	Mansonia sp.	2
2020	Novembro	23/11/2020	48	78	Samauma	P08	Culicidae	Culicinae	Culex	Culex sp.	33
2020	Novembro	23/11/2020	48	78	Samauma	P08	Culicidae	Culicinae	Mansonia	Mansonia sp.	8
2020	Novembro	23/11/2020	48	78	Samauma	P08	Culicidae	Culicinae	Aedeomyia	Adeomyia squamipennis	2
2020	Novembro	23/11/2020	48	78	Jaci Paraná	P06	Culicidae	Culicinae	Mansonia	Mansonia sp.	2
2020	Novembro	23/11/2020	48	78	Jaci Paraná	P06	Culicidae	Culicinae	Culex	Culex sp.	23
2020	Novembro	23/11/2020	48	78	Jaci Paraná	P06	Culicidae	Culicinae	Aedes	Aedes aegypti	7
2020	Novembro	23/11/2020	48	78	Santa Rita	P05	Culicidae	Culicinae	Culex	Culex sp.	13
2020	Novembro	23/11/2020	48	78	Santa Rita	P05	Culicidae	Culicinae	Mansonia	Mansonia sp.	5
2020	Novembro	23/11/2020	48	78	Morrinhos	P04	Culicidae	Culicinae	Mansonia	Mansonia sp.	23
2020	Novembro	23/11/2020	48	78	Morrinhos	P04	Culicidae	Culicinae	Culex	Culex sp.	15
2020	Novembro	23/11/2020	48	78	Teotônio	P03	Culicidae	Culicinae	Culex	Culex sp.	4
2020	Novembro	23/11/2020	48	78	Teotônio	P03	Culicidae	Culicinae	Mansonia	Mansonia sp.	2
2020	Novembro	23/11/2020	48	78	São Domingos	P02	Culicidae	Culicinae	Mansonia	Mansonia sp.	10
2020	Novembro	23/11/2020	48	78	Cujubim Grande	P01	Culicidae	Culicinae	Culex	Culex sp.	5
2020	Novembro	23/11/2020	48	78	Cujubim Grande	P01	Culicidae	Culicinae	Mansonia	Mansonia sp.	1
2020	Novembro	30/11/2020	49	79	Rio Contra	P07	Culicidae	Culicinae	Culex	Culex sp.	1
2020	Novembro	30/11/2020	49	79	Samauma	P08	Culicidae	Culicinae	Culex	Culex sp.	10
2020	Novembro	30/11/2020	49	79	Samauma	P08	Culicidae	Culicinae	Mansonia	Mansonia sp.	23

2020	Novembro	30/11/2020	49	79	Jaci Paraná	P06	Culicidae	Culicinae	Aedes	Aedes aegypti	3
2020	Novembro	30/11/2020	49	79	Jaci Paraná	P06	Culicidae	Culicinae	Mansonia	Mansonia sp.	3
2020	Novembro	30/11/2020	49	79	Jaci Paraná	P06	Culicidae	Culicinae	Culex	Culex sp.	2
2020	Novembro	30/11/2020	49	79	Santa Rita	P05	Culicidae	Anophelinae	Anopheles	Anopheles sp.	1
2020	Novembro	30/11/2020	49	79	Santa Rita	P05	Culicidae	Culicinae	Mansonia	Mansonia sp.	5
2020	Novembro	30/11/2020	49	79	Santa Rita	P05	Culicidae	Culicinae	Culex	Culex sp.	8
2020	Novembro	30/11/2020	49	79	Morrinhos	P04	Culicidae	Culicinae	Culex	Culex sp.	7
2020	Novembro	30/11/2020	49	79	Morrinhos	P04	Culicidae	Culicinae	Mansonia	Mansonia sp.	12
2020	Novembro	30/11/2020	49	79	Teotônio	P03	Culicidae	Culicinae	Mansonia	Mansonia sp.	4
2020	Novembro	30/11/2020	49	79	São Domingos	P02	Culicidae	Culicinae	Mansonia	Mansonia sp.	11
2020	Novembro	30/11/2020	49	79	São Domingos	P02	Culicidae	Culicinae	Culex	Culex sp.	18
2020	Novembro	30/11/2020	49	79	Cujubim Grande	P01	Culicidae	Culicinae	Culex	Culex sp.	5
2020	Dezembro	07/12/2020	50	80	Rio Contra	P07	Culicidae	Culicinae	Mansonia	Mansonia sp.	2
2020	Dezembro	07/12/2020	50	80	Rio Contra	P07	Culicidae	Culicinae	Culex	Culex sp.	1
2020	Dezembro	07/12/2020	50	80	Samauma	P08	Culicidae	Culicinae	Mansonia	Mansonia sp.	17
2020	Dezembro	07/12/2020	50	80	Samauma	P08	Culicidae	Culicinae	Culex	Culex sp.	2
2020	Dezembro	07/12/2020	50	80	Jaci Paraná	P06	Culicidae	Culicinae	Culex	Culex sp.	13
2020	Dezembro	07/12/2020	50	80	Jaci Paraná	P06	Culicidae	Culicinae	Mansonia	Mansonia sp.	1
2020	Dezembro	07/12/2020	50	80	Jaci Paraná	P06	Culicidae	Culicinae	Aedes	Aedes aegypti	8
2020	Dezembro	07/12/2020	50	80	Santa Rita	P05	Culicidae	Culicinae	Mansonia	Mansonia sp.	8
2020	Dezembro	07/12/2020	50	80	Santa Rita	P05	Culicidae	Culicinae	Culex	Culex sp.	7
2020	Dezembro	07/12/2020	50	80	Morrinhos	P04	Culicidae	Culicinae	Mansonia	Mansonia sp.	9
2020	Dezembro	07/12/2020	50	80	Teotônio	P03	Culicidae	Culicinae	Limatus	Limatus durhamii	2
2020	Dezembro	07/12/2020	50	80	Teotônio	P03	Culicidae	Culicinae	Coquillettidia	Coquillettidia sp.	1
2020	Dezembro	07/12/2020	50	80	Teotônio	P03	Culicidae	Culicinae	Mansonia	Mansonia sp.	3
2020	Dezembro	07/12/2020	50	80	São Domingos	P02	Culicidae	Culicinae	Culex	Culex sp.	5
2020	Dezembro	07/12/2020	50	80	São Domingos	P02	Culicidae	Culicinae	Mansonia	Mansonia sp.	10
2020	Dezembro	07/12/2020	50	80	Cujubim Grande	P01	Culicidae	Culicinae	Mansonia	Mansonia sp.	7
2020	Dezembro	14/12/2020	51	81	Rio Contra	P07	Culicidae	Culicinae	Culex	Culex sp.	2
2020	Dezembro	14/12/2020	51	81	Samauma	P08	Culicidae	Culicinae	Mansonia	Mansonia sp.	15
2020	Dezembro	14/12/2020	51	81	Samauma	P08	Culicidae	Culicinae	Culex	Culex sp.	7
2020	Dezembro	14/12/2020	51	81	Samauma	P08	Culicidae	Culicinae	Aedeomyia	Adeomyia squamipennis	2
2020	Dezembro	14/12/2020	51	81	Jaci Paraná	P06	Culicidae	Culicinae	Aedes	Aedes aegypti	5
2020	Dezembro	14/12/2020	51	81	Jaci Paraná	P06	Culicidae	Culicinae	Culex	Culex sp.	3
2020	Dezembro	14/12/2020	51	81	Jaci Paraná	P06	Culicidae	Culicinae	Mansonia	Mansonia sp.	2
2020	Dezembro	14/12/2020	51	81	Santa Rita	P05	Culicidae	Culicinae	Mansonia	Mansonia sp.	7
2020	Dezembro	14/12/2020	51	81	Santa Rita	P05	Culicidae	Culicinae	Culex	Culex sp.	5
2020	Dezembro	14/12/2020	51	81	Morrinhos	P04	Culicidae	Culicinae	Mansonia	Mansonia sp.	11
2020	Dezembro	14/12/2020	51	81	Morrinhos	P04	Culicidae	Culicinae	Culex	Culex sp.	2
2020	Dezembro	14/12/2020	51	81	Teotônio	P03	Culicidae	Culicinae	Mansonia	Mansonia sp.	5
2020	Dezembro	14/12/2020	51	81	Teotônio	P03	Culicidae	Culicinae	Culex	Culex sp.	2
2020	Dezembro	14/12/2020	51	81	São Domingos	P02	Culicidae	Culicinae	Mansonia	Mansonia sp.	8
2020	Dezembro	14/12/2020	51	81	Cujubim Grande	P01	Culicidae	Culicinae	Mansonia	Mansonia sp.	3
2020	Dezembro	21/12/2020	52	82	Rio Contra	P07	Culicidae	Culicinae	Culex	Culex sp.	1
2020	Dezembro	21/12/2020	52	82	Samauma	P08	Culicidae	Culicinae	Culex	Culex sp.	5
2020	Dezembro	21/12/2020	52	82	Samauma	P08	Culicidae	Culicinae	Mansonia	Mansonia sp.	8
2020	Dezembro	21/12/2020	52	82	Jaci Paraná	P06	Culicidae	Culicinae	Culex	Culex sp.	5
2020	Dezembro	21/12/2020	52	82	Jaci Paraná	P06	Culicidae	Culicinae	Aedes	Aedes aegypti	2
2020	Dezembro	21/12/2020	52	82	Jaci Paraná	P06	Culicidae	Culicinae	Mansonia	Mansonia sp.	2
2020	Dezembro	21/12/2020	52	82	Santa Rita	P05	Culicidae	Culicinae	Mansonia	Mansonia sp.	5
2020	Dezembro	21/12/2020	52	82	Morrinhos	P04	Culicidae	Culicinae	Mansonia	Mansonia sp.	3
2020	Dezembro	21/12/2020	52	82	Morrinhos	P04	Culicidae	Culicinae	Culex	Culex sp.	1
2020	Dezembro	21/12/2020	52	82	Teotônio	P03	Culicidae	Culicinae	Mansonia	Mansonia sp.	5
2020	Dezembro	21/12/2020	52	82	Teotônio	P03	Culicidae	Culicinae	Coquillettidia	Coquillettidia sp.	1
2020	Dezembro	21/12/2020	52	82	São Domingos	P02	Culicidae	Culicinae	Mansonia	Mansonia sp.	5
2020	Dezembro	21/12/2020	52	82	São Domingos	P02	Culicidae	Culicinae	Culex	Culex sp.	3
2020	Dezembro	21/12/2020	52	82	Cujubim Grande	P01	Culicidae	Culicinae	Mansonia	Mansonia sp.	2
2020	Dezembro	21/12/2020	52	82	Cujubim Grande	P01	Culicidae	Culicinae	Culex	Culex sp.	1
2020	Dezembro	28/12/2020	53	83	Rio Contra	P07	Culicidae	Culicinae	Mansonia	Mansonia sp.	2
2020	Dezembro	28/12/2020	53	83	Rio Contra	P07	Culicidae	Culicinae	Culex	Culex sp.	2
2020	Dezembro	28/12/2020	53	83	Samauma	P08	Culicidae	Culicinae	Mansonia	Mansonia sp.	19
2020	Dezembro	28/12/2020	53	83	Samauma	P08	Culicidae	Culicinae	Aedeomyia	Adeomyia squamipennis	2
2020	Dezembro	28/12/2020	53	83	Jaci Paraná	P06	Culicidae	Culicinae	Mansonia	Mansonia sp.	2
2020	Dezembro	28/12/2020	53	83	Jaci Paraná	P06	Culicidae	Culicinae	Culex	Culex sp.	1
2020	Dezembro	28/12/2020	53	83	Jaci Paraná	P06	Culicidae	Culicinae	Aedes	Aedes aegypti	5
2020	Dezembro	28/12/2020	53	83	Santa Rita	P05	Culicidae	Culicinae	Mansonia	Mansonia sp.	10
2020	Dezembro	28/12/2020	53	83	Santa Rita	P05	Culicidae	Culicinae	Culex	Culex sp.	8
2020	Dezembro	28/12/2020	53	83	Morrinhos	P04	Culicidae	Culicinae	Mansonia	Mansonia sp.	3
2020	Dezembro	28/12/2020	53	83	Morrinhos	P04	Culicidae	Culicinae	Culex	Culex sp.	1
2020	Dezembro	28/12/2020	53	83	Teotônio	P03	Culicidae	Culicinae	Coquillettidia	Coquillettidia sp.	2
2020	Dezembro	28/12/2020	53	83	Teotônio	P03	Culicidae	Culicinae	Mansonia	Mansonia humeralis	1
2020	Dezembro	28/12/2020	53	83	Teotônio	P03	Culicidae	Culicinae	Culex	Culex sp.	1
2020	Dezembro	28/12/2020	53	83	São Domingos	P02	Culicidae	Culicinae	Mansonia	Mansonia sp.	5
2020	Dezembro	28/12/2020	53	83	Cujubim Grande	P01	Culicidae	Culicinae	Culex	Culex sp.	2
2020	Dezembro	28/12/2020	53	83	Cujubim Grande	P01	Culicidae	Culicinae	Mansonia	Mansonia sp.	1
2021	Janeiro	04/01/2021	1	84	Rio Contra	P07	Culicidae	Culicinae	Mansonia	Mansonia sp.	2
2021	Janeiro	04/01/2021	1	84	Samauma	P08	Culicidae	Culicinae	Culex	Culex sp.	22
2021	Janeiro	04/01/2021	1	84	Samauma	P08	Culicidae	Culicinae	Mansonia	Mansonia sp.	8
2021	Janeiro	04/01/2021	1	84	Samauma	P08	Culicidae	Culicinae	Limatus	Limatus durhamii	2
2021	Janeiro	04/01/2021	1	84	Jaci Paraná	P06	Culicidae	Culicinae	Aedes	Aedes aegypti	5
2021	Janeiro	04/01/2021	1	84	Jaci Paraná	P06	Culicidae	Culicinae	Mansonia	Mansonia sp.	3
2021	Janeiro	04/01/2021	1	84	Jaci Paraná	P06	Culicidae	Culicinae	Culex	Culex sp.	2
2021	Janeiro	04/01/2021	1	84	Santa Rita	P05	Culicidae	Culicinae	Mansonia	Mansonia sp.	11
2021	Janeiro	04/01/2021	1	84	Morrinhos	P04	Culicidae	Culicinae	Mansonia	Mansonia sp.	12
2021	Janeiro	04/01/2021	1	84	Morrinhos	P04	Culicidae	Culicinae	Culex	Culex sp.	15
2021	Janeiro	04/01/2021	1	84	Teotônio	P03	Culicidae	Culicinae	Limatus	Limatus durhamii	2
2021	Janeiro	04/01/2021	1	84	Teotônio	P03	Culicidae	Culicinae	Coquillettidia	Coquillettidia sp.	5
2021	Janeiro	04/01/2021	1	84	Teotônio	P03	Culicidae	Culicinae	Mansonia	Mansonia sp.	1
2021	Janeiro	04/01/2021	1	84	São Domingos	P02	Culicidae	Culicinae	Mansonia	Mansonia sp.	8
2021	Janeiro	04/01/2021	1	84	São Domingos	P02	Culicidae	Culicinae	Culex	Culex sp.	3
2021	Janeiro	04/01/2021	1	84	Cujubim Grande	P01	Culicidae	Culicinae	Culex	Culex sp.	10
2021	Janeiro	04/01/2021	1	84	Cujubim Grande	P01	Culicidae	Culicinae	Mansonia	Mansonia sp.	1
2021	Janeiro	11/01/2021	2	85	Samauma	P08	Culicidae	Culicinae	Mansonia	Mansonia sp.	8
2021	Janeiro	11/01/2021	2	85	Samauma	P08	Culicidae	Culicinae	Culex	Culex sp.	3
2021	Janeiro	11/01/2021	2	85	Samauma	P08	Culicidae	Culicinae	Aedeomyia	Adeomyia squamipennis	1
2021	Janeiro	11/01/2021	2	85	Jaci Paraná	P06	Culicidae	Culicinae	Aedes	Aedes aegypti	10
2021	Janeiro	11/01/2021	2	85	Jaci Paraná	P06	Culicidae	Culicinae	Mansonia	Mansonia sp.	3
2021	Janeiro	11/01/2021	2	85	Jaci Paraná	P06	Culicidae	Culicinae	Culex	Culex sp.	5
2021	Janeiro	11/01/2021	2	85	Santa Rita	P05	Culicidae	Culicinae	Mansonia	Mansonia sp.	3
2021	Janeiro	11/01/2021	2	85	Morrinhos	P04	Culicidae	Culicinae	Mansonia	Mansonia sp.	13
2021	Janeiro	11/01/2021	2	85	Morrinhos	P04	Culicidae	Culicinae	Culex	Culex sp.	2
2021	Janeiro	11/01/2021	2	85	Morrinhos	P04	Culicidae	Culicinae	Limatus	Limatus durhamii	1
2021	Janeiro	11/01/2021	2	85	Teotônio	P03	Culicidae	Culicinae	Coquillettidia	Coquillettidia sp.	3
2021	Janeiro	11/01/2021	2	85	Teotônio	P03	Culicidae	Culicinae	Culex	Culex sp.	2
2021	Janeiro	11/01/2021	2	85	São Domingos	P02	Culicidae	Culicinae	Aedes	Aedes sp.	2
2021	Janeiro	11/01/2021	2	85	São Domingos	P02	Culicidae	Culicinae	Mansonia	Mansonia sp.	3
2021	Janeiro	11/01/2021	2	85	São Domingos	P02	Culicidae	Culicinae	Culex	Culex sp.	5
2021	Janeiro	11/01/2021	2	85	São Domingos	P02	Culicidae	Culicinae	Coquillettidia	Coquillettidia sp.	1
2021	Janeiro	11/01/2021	2	85	Cujubim Grande	P01	Culicidae	Culicinae	Culex	Culex sp.	5
2021	Janeiro	11/01/2021	2	85	Cujubim Grande	P01	Culicidae	Culicinae	Mansonia	Mansonia sp.	2
2021	Janeiro	18/01/2021	3	86	Samauma	P08	Culicidae	Culicinae	Mansonia	Mansonia sp.	7
2021	Janeiro	18/01/2021	3	86	Samauma	P08	Culicidae	Culicinae	Culex	Culex sp.	21
2021	Janeiro	18/01/2021	3	86	Jaci Paraná	P06	Culicidae	Culicinae	Mansonia	Mansonia sp.	5
2021	Janeiro	18/01/2021	3	86	Jaci Paraná	P06	Culicidae	Culicinae	Aedes	Aedes aegypti	3
2021	Janeiro	18/01/2021	3	86	Jaci Paraná	P06	Culicidae	Culicinae	Culex	Culex sp.	10
2021	Janeiro	18/01/2021	3	86	Santa Rita	P05	Culicidae	Culicinae	Mansonia	Mansonia sp.	5
2021	Janeiro	18/01/2021	3	86	Santa Rita	P05	Culicidae	Culicinae	Culex	Culex sp.	5
2021	Janeiro	18/01/2021	3	86	Santa Rita	P05	Culicidae	Culicinae	Limatus	Limatus durhamii	2

2021	Janeiro	18/01/2021	3	86	Morrinhos	P04	Culicidae	Culicinae	*Culex*	*Culex sp.*	3
2021	Janeiro	18/01/2021	3	86	Morrinhos	P04	Culicidae	Culicinae	*Mansonia*	*Mansonia sp.*	1
2021	Janeiro	18/01/2021	3	86	Teotônio	P03	Culicidae	Culicinae	*Mansonia*	*Mansonia sp.*	2
2021	Janeiro	18/01/2021	3	86	Teotônio	P03	Culicidae	Culicinae	*Culex*	*Culex sp.*	1
2021	Janeiro	18/01/2021	3	86	São Domingos	P02	Culicidae	Culicinae	*Mansonia*	*Mansonia sp.*	7
2021	Janeiro	18/01/2021	3	86	São Domingos	P02	Culicidae	Culicinae	*Culex*	*Culex sp.*	2
2021	Janeiro	18/01/2021	3	86	Cujubim Grande	P01	Culicidae	Culicinae	*Culex*	*Culex sp.*	18
2021	Janeiro	18/01/2021	3	86	Cujubim Grande	P01	Culicidae	Culicinae	*Mansonia*	*Mansonia sp.*	4
2021	Janeiro	25/01/2021	4	87	Teotônio	P03	Culicidae	Culicinae	*Coquillettidia*	*Coquillettidia sp.*	3
2021	Janeiro	25/01/2021	4	87	Teotônio	P03	Culicidae	Culicinae	*Wyeomyia*	*Wyeomyia sp.*	1
2021	Janeiro	25/01/2021	4	87	Cujubim Grande	P01	Culicidae	Culicinae	*Culex*	*Culex sp.*	14
2021	Janeiro	25/01/2021	4	87	Cujubim Grande	P01	Culicidae	Culicinae	*Uranotaenia*	*Uranotaenia sp.*	1
2021	Janeiro	25/01/2021	4	87	São Domingos	P02	Culicidae	Culicinae	*Mansonia*	*Mansonia sp.*	5
2021	Janeiro	25/01/2021	4	87	São Domingos	P02	Culicidae	Culicinae	*Coquillettidia*	*Coquillettidia sp.*	1
2021	Janeiro	25/01/2021	4	87	Samauma	P08	Culicidae	Culicinae	*Mansonia*	*Mansonia sp.*	43
2021	Janeiro	25/01/2021	4	87	Samauma	P08	Culicidae	Culicinae	*Culex*	*Culex sp.*	18
2021	Janeiro	25/01/2021	4	87	Samauma	P08	Culicidae	Culicinae	*Coquillettidia*	*Coquillettidia sp.*	4
2021	Janeiro	25/01/2021	4	87	Samauma	P08	Culicidae	Culicinae	*Wyeomyia*	*Wyeomyia sp.*	2
2021	Janeiro	25/01/2021	4	87	Samauma	P08	Culicidae	Culicinae	*Aedes*	*Aedes scapularis*	1
2021	Janeiro	25/01/2021	4	87	Samauma	P08	Culicidae	Culicinae	*Psorophora*	*Psorophora sp.*	3
2021	Janeiro	25/01/2021	4	87	Morrinhos	P04	Culicidae	Culicinae	*Mansonia*	*Mansonia sp.*	22
2021	Janeiro	25/01/2021	4	87	Morrinhos	P04	Culicidae	Culicinae	*Culex*	*Culex sp.*	12
2021	Janeiro	25/01/2021	4	87	Morrinhos	P04	Culicidae	Culicinae	*Uranotaenia*	*Uranotaenia sp.*	1
2021	Janeiro	25/01/2021	4	87	Morrinhos	P04	Culicidae	Culicinae	*Coquillettidia*	*Coquillettidia sp.*	2
2021	Janeiro	25/01/2021	4	87	Jaci Paraná	P06	Culicidae	Culicinae	*Culex*	*Culex sp.*	70
2021	Janeiro	25/01/2021	4	87	Jaci Paraná	P06	Culicidae	Culicinae	*Aedes*	*Aedes aegypti*	18
2021	Janeiro	25/01/2021	4	87	Jaci Paraná	P06	Culicidae	Culicinae	*Aedes*	*Aedes sp.*	9
2021	Janeiro	25/01/2021	4	87	Jaci Paraná	P06	Culicidae	Culicinae	*Psorophora*	*Psorophora sp.*	2
2021	Janeiro	25/01/2021	4	87	Jaci Paraná	P06	Culicidae	Culicinae	*Mansonia*	*Mansonia sp.*	3
2021	Janeiro	25/01/2021	4	87	Jaci Paraná	P06	Culicidae	Culicinae	*Limatus*	*Limatus durhamii*	2
2021	Janeiro	25/01/2021	4	87	Jaci Paraná	P06	Culicidae	Culicinae	*Uranotaenia*	*Uranotaenia sp.*	1
2021	Fevereiro	01/02/2021	5	88	Jaci Paraná	P06	Culicidae	Culicinae	*Mansonia*	*Mansonia sp.*	6
2021	Fevereiro	01/02/2021	5	88	Jaci Paraná	P06	Culicidae	Culicinae	*Aedeomyia*	*Adeomyia squamipennis*	4
2021	Fevereiro	01/02/2021	5	88	Jaci Paraná	P06	Culicidae	Culicinae	*Limatus*	*Limatus durhamii*	1
2021	Fevereiro	01/02/2021	5	88	Jaci Paraná	P06	Culicidae	Culicinae	*Culex*	*Culex sp.*	70
2021	Fevereiro	01/02/2021	5	88	Jaci Paraná	P06	Culicidae	Culicinae	*Aedes*	*Aedes aegypti*	10
2021	Fevereiro	01/02/2021	5	88	Jaci Paraná	P06	Culicidae	Culicinae	*Aedes*	*Aedes scapularis*	3
2021	Fevereiro	01/02/2021	5	88	Jaci Paraná	P06	Culicidae	Culicinae	*Psorophora*	*Psorophora sp.*	3
2021	Fevereiro	01/02/2021	5	88	Jaci Paraná	P06	Culicidae	Culicinae	*Coquillettidia*	*Coquillettidia venezuelensis*	1
2021	Fevereiro	01/02/2021	5	88	Samauma	P08	Culicidae	Culicinae	*Culex*	*Culex sp.*	63
2021	Fevereiro	01/02/2021	5	88	Samauma	P08	Culicidae	Culicinae	*Coquillettidia*	*Coquillettidia shannoni*	4
2021	Fevereiro	01/02/2021	5	88	Samauma	P08	Culicidae	Culicinae	*Aedeomyia*	*Adeomyia squamipennis*	3
2021	Fevereiro	01/02/2021	5	88	Samauma	P08	Culicidae	Culicinae	*Aedes*	*Aedes scapularis*	2
2021	Fevereiro	01/02/2021	5	88	Samauma	P08	Culicidae	Culicinae	*Wyeomyia*	*Wyeomyia sp.*	4
2021	Fevereiro	01/02/2021	5	88	Samauma	P08	Culicidae	Culicinae	*Mansonia*	*Mansonia sp.*	90
2021	Fevereiro	01/02/2021	5	88	São Domingos	P02	Culicidae	Culicinae	*Mansonia*	*Mansonia sp.*	8
2021	Fevereiro	01/02/2021	5	88	São Domingos	P02	Culicidae	Culicinae	*Coquillettidia*	*Coquillettidia sp.*	4
2021	Fevereiro	01/02/2021	5	88	São Domingos	P02	Culicidae	Culicinae	*Culex*	*Culex sp.*	1
2021	Fevereiro	01/02/2021	5	88	Cujubim Grande	P01	Culicidae	Culicinae	*Culex*	*Culex sp.*	3
2021	Fevereiro	01/02/2021	5	88	Santa Rita	P05	Culicidae	Culicinae	*Mansonia*	*Mansonia sp.*	1
2021	Fevereiro	01/02/2021	5	88	Morrinhos	P04	Culicidae	Culicinae	*Culex*	*Culex sp.*	22
2021	Fevereiro	01/02/2021	5	88	Morrinhos	P04	Culicidae	Culicinae	*Mansonia*	*Mansonia sp.*	9
2021	Fevereiro	01/02/2021	5	88	Morrinhos	P04	Culicidae	Culicinae	*Uranotaenia*	*Uranotaenia sp.*	1
2021	Fevereiro	01/02/2021	5	88	Morrinhos	P04	Culicidae	Culicinae	*Aedes*	*Aedes scapularis*	1
2021	Fevereiro	01/02/2021	5	88	Rio Contra	P07	Culicidae	Culicinae	*Mansonia*	*Mansonia sp.*	27
2021	Fevereiro	01/02/2021	5	88	Rio Contra	P07	Culicidae	Culicinae	*Culex*	*Culex sp.*	7
2021	Fevereiro	01/02/2021	5	88	Rio Contra	P07	Culicidae	Culicinae	*Aedes*	*Aedes scapularis*	2
2021	Fevereiro	01/02/2021	5	88	Rio Contra	P07	Culicidae	Culicinae	*Psorophora*	*Psorophora sp.*	3
2021	Fevereiro	01/02/2021	5	88	Rio Contra	P07	Culicidae	Culicinae	*Limatus*	*Limatus durhamii*	2
2021	Fevereiro	01/02/2021	5	88	Rio Contra	P07	Culicidae	Anophelinae	*Anopheles*	*Anopheles sp.*	5
2021	Fevereiro	08/02/2021	6	89	Teotônio	P03	Culicidae	Culicinae	*Coquillettidia*	*Coquillettidia sp.*	1
2021	Fevereiro	08/02/2021	6	89	Morrinhos	P04	Culicidae	Culicinae	*Mansonia*	*Mansonia sp.*	22
2021	Fevereiro	08/02/2021	6	89	Morrinhos	P04	Culicidae	Culicinae	*Culex*	*Culex sp.*	14
2021	Fevereiro	08/02/2021	6	89	Morrinhos	P04	Culicidae	Culicinae	*Uranotaenia*	*Uranotaenia sp.*	2
2021	Fevereiro	08/02/2021	6	89	Morrinhos	P04	Culicidae	Culicinae	*Limatus*	*Limatus durhamii*	2
2021	Fevereiro	08/02/2021	6	89	Cujubim Grande	P01	Culicidae	Culicinae	*Culex*	*Culex sp.*	7
2021	Fevereiro	08/02/2021	6	89	Cujubim Grande	P01	Culicidae	Culicinae	*Mansonia*	*Mansonia sp.*	1
2021	Fevereiro	08/02/2021	6	89	Cujubim Grande	P01	Culicidae	Culicinae	*Aedes*	*Aedes aegypti*	1
2021	Fevereiro	08/02/2021	6	89	São Domingos	P02	Culicidae	Culicinae	*Mansonia*	*Mansonia sp.*	5
2021	Fevereiro	08/02/2021	6	89	São Domingos	P02	Culicidae	Culicinae	*Coquillettidia*	*Coquillettidia sp.*	1
2021	Fevereiro	08/02/2021	6	89	Jaci Paraná	P06	Culicidae	Culicinae	*Mansonia*	*Mansonia sp.*	6
2021	Fevereiro	08/02/2021	6	89	Jaci Paraná	P06	Culicidae	Culicinae	*Psorophora*	*Psorophora ferox*	3
2021	Fevereiro	08/02/2021	6	89	Jaci Paraná	P06	Culicidae	Culicinae	*Limatus*	*Limatus durhamii*	2
2021	Fevereiro	08/02/2021	6	89	Jaci Paraná	P06	Culicidae	Culicinae	*Culex*	*Culex sp.*	60
2021	Fevereiro	08/02/2021	6	89	Jaci Paraná	P06	Culicidae	Culicinae	*Aedes*	*Aedes aegypti*	29
2021	Fevereiro	08/02/2021	6	89	Rio Contra	P07	Culicidae	Anophelinae	*Anopheles*	*Anopheles sp.*	4
2021	Fevereiro	08/02/2021	6	89	Rio Contra	P07	Culicidae	Culicinae	*Culex*	*Culex sp.*	14
2021	Fevereiro	08/02/2021	6	89	Rio Contra	P07	Culicidae	Culicinae	*Mansonia*	*Mansonia sp.*	8
2021	Fevereiro	08/02/2021	6	89	Rio Contra	P07	Culicidae	Culicinae	*Uranotaenia*	*Uranotaenia sp.*	2
2021	Fevereiro	08/02/2021	6	89	Santa Rita	P05	Culicidae	Anophelinae	*Anopheles*	*Anopheles sp.*	2
2021	Fevereiro	08/02/2021	6	89	Santa Rita	P05	Culicidae	Culicinae	*Culex*	*Culex sp.*	6
2021	Fevereiro	08/02/2021	6	89	Santa Rita	P05	Culicidae	Culicinae	*Uranotaenia*	*Uranotaenia sp.*	3
2021	Fevereiro	08/02/2021	6	89	Santa Rita	P05	Culicidae	Culicinae	*Aedeomyia*	*Adeomyia squamipennis*	2
2021	Fevereiro	08/02/2021	6	89	Samauma	P08	Culicidae	Culicinae	*Mansonia*	*Mansonia sp.*	58
2021	Fevereiro	08/02/2021	6	89	Samauma	P08	Culicidae	Culicinae	*Culex*	*Culex sp.*	8
2021	Fevereiro	08/02/2021	6	89	Samauma	P08	Culicidae	Culicinae	*Aedeomyia*	*Adeomyia squamipennis*	1
2021	Fevereiro	15/02/2021	7	90	Morrinhos	P04	Culicidae	Culicinae	*Mansonia*	*Mansonia sp.*	9
2021	Fevereiro	15/02/2021	7	90	Morrinhos	P04	Culicidae	Culicinae	*Culex*	*Culex sp.*	14
2021	Fevereiro	15/02/2021	7	90	Morrinhos	P04	Culicidae	Culicinae	*Uranotaenia*	*Uranotaenia sp.*	1
2021	Fevereiro	15/02/2021	7	90	Cujubim Grande	P01	Culicidae	Culicinae	*Culex*	*Culex sp.*	80
2021	Fevereiro	15/02/2021	7	90	Cujubim Grande	P01	Culicidae	Culicinae	*Mansonia*	*Mansonia sp.*	6
2021	Fevereiro	15/02/2021	7	90	Cujubim Grande	P01	Culicidae	Culicinae	*Uranotaenia*	*Uranotaenia sp.*	4
2021	Fevereiro	15/02/2021	7	90	Cujubim Grande	P01	Culicidae	Culicinae	*Limatus*	*Limatus durhamii*	1
2021	Fevereiro	15/02/2021	7	90	Cujubim Grande	P01	Culicidae	Culicinae	*Wyeomyia*	*Wyeomyia sp.*	1
2021	Fevereiro	15/02/2021	7	90	Cujubim Grande	P01	Culicidae	Anophelinae	*Anopheles*	*Anopheles sp.*	1
2021	Fevereiro	15/02/2021	7	90	Cujubim Grande	P01	Culicidae	Culicinae	*Aedes*	*Aedes aegypti*	1
2021	Fevereiro	15/02/2021	7	90	Rio Contra	P07	Culicidae	Anophelinae	*Anopheles*	*Anopheles sp.*	2
2021	Fevereiro	15/02/2021	7	90	Rio Contra	P07	Culicidae	Culicinae	*Culex*	*Culex sp.*	1
2021	Fevereiro	15/02/2021	7	90	Samauma	P08	Culicidae	Culicinae	*Mansonia*	*Mansonia sp.*	22
2021	Fevereiro	15/02/2021	7	90	Samauma	P08	Culicidae	Culicinae	*Culex*	*Culex sp.*	23
2021	Fevereiro	15/02/2021	7	90	Samauma	P08	Culicidae	Culicinae	*Wyeomyia*	*Wyeomyia sp.*	4
2021	Fevereiro	15/02/2021	7	90	Samauma	P08	Culicidae	Culicinae	*Aedeomyia*	*Adeomyia squamipennis*	1
2021	Fevereiro	15/02/2021	7	90	Samauma	P08	Culicidae	Culicinae	*Psorophora*	*Psorophora sp.*	1
2021	Fevereiro	15/02/2021	7	90	Santa Rita	P05	Culicidae	Culicinae	*Mansonia*	*Mansonia sp.*	5
2021	Fevereiro	15/02/2021	7	90	Santa Rita	P05	Culicidae	Anophelinae	*Anopheles*	*Anopheles sp.*	4
2021	Fevereiro	15/02/2021	7	90	Santa Rita	P05	Culicidae	Culicinae	*Culex*	*Culex sp.*	1
2021	Fevereiro	15/02/2021	7	90	Jaci Paraná	P06	Culicidae	Culicinae	*Mansonia*	*Mansonia sp.*	8
2021	Fevereiro	15/02/2021	7	90	Jaci Paraná	P06	Culicidae	Culicinae	*Aedes*	*Aedes aegypti*	13
2021	Fevereiro	15/02/2021	7	90	Jaci Paraná	P06	Culicidae	Culicinae	*Culex*	*Culex sp.*	24
2021	Fevereiro	15/02/2021	7	90	Jaci Paraná	P06	Culicidae	Culicinae	*Psorophora*	*Psorophora sp.*	1
2021	Fevereiro	22/02/2021	8	91	Cujubim Grande	P01	Culicidae	Culicinae	*Mansonia*	*Mansonia sp.*	4
2021	Fevereiro	22/02/2021	8	91	Cujubim Grande	P01	Culicidae	Culicinae	*Culex*	*Culex sp.*	30
2021	Fevereiro	22/02/2021	8	91	Cujubim Grande	P01	Culicidae	Culicinae	*Wyeomyia*	*Wyeomyia sp.*	2
2021	Fevereiro	22/02/2021	8	91	Cujubim Grande	P01	Culicidae	Culicinae	*Limatus*	*Limatus durhamii*	2
2021	Fevereiro	22/02/2021	8	91	Cujubim Grande	P01	Culicidae	Culicinae	*Uranotaenia*	*Uranotaenia sp.*	2
2021	Fevereiro	22/02/2021	8	91	Cujubim Grande	P01	Culicidae	Anophelinae	*Anopheles*	*Anopheles sp.*	1
2021	Fevereiro	22/02/2021	8	91	Cujubim Grande	P01	Culicidae	Culicinae	*Aedes*	*Aedes aegypti*	1
2021	Fevereiro	22/02/2021	8	91	Teotônio	P03	Culicidae	Culicinae	*Coquillettidia*	*Coquillettidia sp.*	1

2021	Fevereiro	22/02/2021	8	91	Morrinhos	P04	Culicidae	Culicinae	*Culex*	*Culex sp.*	42
2021	Fevereiro	22/02/2021	8	91	Morrinhos	P04	Culicidae	Culicinae	*Mansonia*	*Mansonia sp.*	8
2021	Fevereiro	22/02/2021	8	91	Morrinhos	P04	Culicidae	Culicinae	*Psorophora*	*Psorophora sp.*	1
2021	Fevereiro	22/02/2021	8	91	Santa Rita	P05	Culicidae	Culicinae	*Mansonia*	*Mansonia sp.*	3
2021	Fevereiro	22/02/2021	8	91	Santa Rita	P05	Culicidae	Culicinae	*Culex*	*Culex sp.*	5
2021	Fevereiro	22/02/2021	8	91	Santa Rita	P05	Culicidae	Culicinae	*Uranotaenia*	*Uranotaenia sp.*	3
2021	Fevereiro	22/02/2021	8	91	Santa Rita	P05	Culicidae	Culicinae	*Coquillettidia*	*Coquillettidia sp.*	3
2021	Fevereiro	22/02/2021	8	91	Santa Rita	P05	Culicidae	Culicinae	*Aedeomyia*	*Adeomyia squamipennis*	1
2021	Fevereiro	22/02/2021	8	91	Samauma	P08	Culicidae	Culicinae	*Mansonia*	*Mansonia sp.*	8
2021	Fevereiro	22/02/2021	8	91	Samauma	P08	Culicidae	Culicinae	*Culex*	*Culex sp.*	8
2021	Fevereiro	22/02/2021	8	91	Samauma	P08	Culicidae	Culicinae	*Aedes*	*Aedes scapularis*	1
2021	Fevereiro	22/02/2021	8	91	Samauma	P08	Culicidae	Culicinae	*Aedes*	*Aedes aegypti*	1
2021	Fevereiro	22/02/2021	8	91	São Domingos	P02	Culicidae	Culicinae	*Mansonia*	*Mansonia sp.*	4
2021	Fevereiro	22/02/2021	8	91	São Domingos	P02	Culicidae	Culicinae	*Aedeomyia*	*Adeomyia squamipennis*	1
2021	Fevereiro	22/02/2021	8	91	São Domingos	P02	Culicidae	Culicinae	*Coquillettidia*	*Coquillettidia sp.*	1
2021	Fevereiro	22/02/2021	8	91	Jaci Paraná	P06	Culicidae	Culicinae	*Culex*	*Culex sp.*	46
2021	Fevereiro	22/02/2021	8	91	Jaci Paraná	P06	Culicidae	Culicinae	*Mansonia*	*Mansonia sp.*	10
2021	Fevereiro	22/02/2021	8	91	Jaci Paraná	P06	Culicidae	Culicinae	*Aedeomyia*	*Adeomyia squamipennis*	4
2021	Fevereiro	22/02/2021	8	91	Jaci Paraná	P06	Culicidae	Anophelinae	*Anopheles*	*Anopheles darlingi*	1
2021	Fevereiro	22/02/2021	8	91	Jaci Paraná	P06	Culicidae	Culicinae	*Limatus*	*Limatus durhamii*	1
2021	Fevereiro	22/02/2021	8	91	Jaci Paraná	P06	Culicidae	Culicinae	*Aedes*	*Aedes aegypti*	9
2021	Fevereiro	22/02/2021	8	91	Jaci Paraná	P06	Culicidae	Culicinae	*Aedes*	*Aedes scapularis*	1
2021	Fevereiro	22/02/2021	8	91	Rio Contra	P07	Culicidae	Culicinae	*Mansonia*	*Mansonia sp.*	6
2021	Fevereiro	22/02/2021	8	91	Rio Contra	P07	Culicidae	Culicinae	*Culex*	*Culex sp.*	9
2021	Fevereiro	22/02/2021	8	91	Rio Contra	P07	Culicidae	Culicinae	*Uranotaenia*	*Uranotaenia lowii*	3
2021	Fevereiro	22/02/2021	8	91	Rio Contra	P07	Culicidae	Culicinae	*Limatus*	*Limatus durhamii*	1
2021	Março	01/03/2021	9	92	Teotônio	P03	Culicidae	Culicinae	*Mansonia*	*Mansonia sp.*	4
2021	Março	01/03/2021	9	92	Teotônio	P03	Culicidae	Culicinae	*Culex*	*Culex sp.*	7
2021	Março	01/03/2021	9	92	Teotônio	P03	Culicidae	Culicinae	*Coquillettidia*	*Coquillettidia sp.*	9
2021	Março	01/03/2021	9	92	Teotônio	P03	Culicidae	Culicinae	*Uranotaenia*	*Uranotaenia sp.*	5
2021	Março	01/03/2021	9	92	Teotônio	P03	Culicidae	Culicinae	*Wyeomyia*	*Wyeomyia sp.*	1
2021	Março	01/03/2021	9	92	Teotônio	P03	Culicidae	Culicinae	*Wyeomyia*	*Wyeomyia sp.*	1
2021	Março	01/03/2021	9	92	Teotônio	P03	Culicidae	Anophelinae	*Anopheles*	*Anopheles sp.*	3
2021	Março	01/03/2021	9	92	Teotônio	P03	Culicidae	Culicinae	*Limatus*	*Limatus durhamii*	1
2021	Março	01/03/2021	9	92	Santa Rita	P05	Culicidae	Culicinae	*Mansonia*	*Mansonia sp.*	13
2021	Março	01/03/2021	9	92	Santa Rita	P05	Culicidae	Culicinae	*Culex*	*Culex sp.*	4
2021	Março	01/03/2021	9	92	Santa Rita	P05	Culicidae	Anophelinae	*Anopheles*	*Anopheles sp.*	3
2021	Março	01/03/2021	9	92	Jaci Paraná	P06	Culicidae	Culicinae	*Culex*	*Culex sp.*	115
2021	Março	01/03/2021	9	92	Jaci Paraná	P06	Culicidae	Culicinae	*Mansonia*	*Mansonia sp.*	9
2021	Março	01/03/2021	9	92	Jaci Paraná	P06	Culicidae	Culicinae	*Aedes*	*Aedes aegypti*	15
2021	Março	01/03/2021	9	92	Jaci Paraná	P06	Culicidae	Culicinae	*Aedes*	*Aedes scapularis*	1
2021	Março	01/03/2021	9	92	Jaci Paraná	P06	Culicidae	Culicinae	*Aedeomyia*	*Adeomyia squamipennis*	5
2021	Março	01/03/2021	9	92	Jaci Paraná	P06	Culicidae	Anophelinae	*Anopheles*	*Anopheles sp.*	1
2021	Março	01/03/2021	9	92	Morrinhos	P04	Culicidae	Culicinae	*Mansonia*	*Mansonia sp.*	6
2021	Março	01/03/2021	9	92	Morrinhos	P04	Culicidae	Culicinae	*Culex*	*Culex sp.*	4
2021	Março	01/03/2021	9	92	Samauma	P08	Culicidae	Culicinae	*Mansonia*	*Mansonia sp.*	7
2021	Março	01/03/2021	9	92	Samauma	P08	Culicidae	Culicinae	*Culex*	*Culex sp.*	16
2021	Março	01/03/2021	9	92	Cujubim Grande	P01	Culicidae	Culicinae	*Culex*	*Culex sp.*	18
2021	Março	01/03/2021	9	92	Cujubim Grande	P01	Culicidae	Culicinae	*Mansonia*	*Mansonia sp.*	2
2021	Março	01/03/2021	9	92	Cujubim Grande	P01	Culicidae	Culicinae	*Psorophora*	*Psorophora sp.*	1
2021	Março	01/03/2021	9	92	Cujubim Grande	P01	Culicidae	Anophelinae	*Anopheles*	*Anopheles sp.*	1
2021	Março	01/03/2021	9	92	Rio Contra	P07	Culicidae	Culicinae	*Mansonia*	*Mansonia sp.*	2
2021	Março	01/03/2021	9	92	Rio Contra	P07	Culicidae	Culicinae	*Culex*	*Culex sp.*	1
2021	Março	01/03/2021	9	92	Rio Contra	P07	Culicidae	Anophelinae	*Anopheles*	*Anopheles sp.*	1
2021	Março	08/03/2021	10	93	Rio Contra	P07	Culicidae	Culicinae	*Mansonia*	*Mansonia sp.*	8
2021	Março	08/03/2021	10	93	Samauma	P08	Culicidae	Culicinae	*Aedeomyia*	*Adeomyia squamipennis*	4
2021	Março	08/03/2021	10	93	Samauma	P08	Culicidae	Culicinae	*Culex*	*Culex sp.*	6
2021	Março	08/03/2021	10	93	Samauma	P08	Culicidae	Culicinae	*Mansonia*	*Mansonia sp.*	21
2021	Março	08/03/2021	10	93	Samauma	P08	Culicidae	Culicinae	*Coquillettidia*	*Coquillettidia sp.*	5
2021	Março	08/03/2021	10	93	Jaci Paraná	P06	Culicidae	Anophelinae	*Anopheles*	*Anopheles darlingi*	5
2021	Março	08/03/2021	10	93	Jaci Paraná	P06	Culicidae	Culicinae	*Culex*	*Culex sp.*	32
2021	Março	08/03/2021	10	93	Jaci Paraná	P06	Culicidae	Culicinae	*Aedes*	*Aedes sp.*	5
2021	Março	08/03/2021	10	93	Jaci Paraná	P06	Culicidae	Culicinae	*Mansonia*	*Mansonia sp.*	8
2021	Março	08/03/2021	10	93	Santa Rita	P05	Culicidae	Culicinae	*Culex*	*Culex sp.*	5
2021	Março	08/03/2021	10	93	Santa Rita	P05	Culicidae	Culicinae	*Mansonia*	*Mansonia sp.*	3
2021	Março	08/03/2021	10	93	Morrinhos	P04	Culicidae	Culicinae	*Mansonia*	*Mansonia sp.*	5
2021	Março	08/03/2021	10	93	Morrinhos	P04	Culicidae	Culicinae	*Culex*	*Culex sp.*	2
2021	Março	08/03/2021	10	93	Teotônio	P03	Culicidae	Culicinae	*Mansonia*	*Mansonia sp.*	3
2021	Março	08/03/2021	10	93	Teotônio	P03	Culicidae	Culicinae	*Coquillettidia*	*Coquillettidia sp.*	2
2021	Março	08/03/2021	10	93	São Domingos	P02	Culicidae	Culicinae	*Mansonia*	*Mansonia sp.*	1
2021	Março	08/03/2021	10	93	São Domingos	P02	Culicidae	Culicinae	*Coquillettidia*	*Coquillettidia sp.*	5
2021	Março	08/03/2021	10	93	Cujubim Grande	P01	Culicidae	Culicinae	*Uranotaenia*	*Uranotaenia sp.*	2
2021	Março	08/03/2021	10	93	Cujubim Grande	P01	Culicidae	Culicinae	*Aedes*	*Aedes sp.*	2
2021	Março	08/03/2021	10	93	Cujubim Grande	P01	Culicidae	Culicinae	*Culex*	*Culex sp.*	5
2021	Março	08/03/2021	10	93	Cujubim Grande	P01	Culicidae	Culicinae	*Mansonia*	*Mansonia sp.*	3
2021	Março	15/03/2021	11	94	Rio Contra	P07	Culicidae	Anophelinae	*Anopheles*	*Anopheles darlingi*	2
2021	Março	15/03/2021	11	94	Rio Contra	P07	Culicidae	Culicinae	*Mansonia*	*Mansonia sp.*	11
2021	Março	15/03/2021	11	94	Samauma	P08	Culicidae	Culicinae	*Mansonia*	*Mansonia sp.*	9
2021	Março	15/03/2021	11	94	Samauma	P08	Culicidae	Culicinae	*Culex*	*Culex sp.*	1
2021	Março	15/03/2021	11	94	Samauma	P08	Culicidae	Culicinae	*Aedeomyia*	*Adeomyia squamipennis*	2
2021	Março	15/03/2021	11	94	Jaci Paraná	P06	Culicidae	Anophelinae	*Anopheles*	*Anopheles darlingi*	2
2021	Março	15/03/2021	11	94	Jaci Paraná	P06	Culicidae	Culicinae	*Psorophora*	*Psorophora ferox*	3
2021	Março	15/03/2021	11	94	Jaci Paraná	P06	Culicidae	Culicinae	*Culex*	*Culex sp.*	52
2021	Março	15/03/2021	11	94	Jaci Paraná	P06	Culicidae	Culicinae	*Aedes*	*Aedes sp.*	5
2021	Março	15/03/2021	11	94	Jaci Paraná	P06	Culicidae	Culicinae	*Mansonia*	*Mansonia sp.*	4
2021	Março	15/03/2021	11	94	Jaci Paraná	P06	Culicidae	Culicinae	*Aedeomyia*	*Adeomyia squamipennis*	1
2021	Março	15/03/2021	11	94	Santa Rita	P05	Culicidae	Culicinae	*Mansonia*	*Mansonia sp.*	5
2021	Março	15/03/2021	11	94	Santa Rita	P05	Culicidae	Culicinae	*Culex*	*Culex sp.*	3
2021	Março	15/03/2021	11	94	Santa Rita	P05	Culicidae	Culicinae	*Aedes*	*Aedes sp.*	2
2021	Março	15/03/2021	11	94	Morrinhos	P04	Culicidae	Culicinae	*Psorophora*	*Psorophora sp.*	7
2021	Março	15/03/2021	11	94	Morrinhos	P04	Culicidae	Culicinae	*Culex*	*Culex sp.*	5
2021	Março	15/03/2021	11	94	Morrinhos	P04	Culicidae	Culicinae	*Mansonia*	*Mansonia sp.*	3
2021	Março	15/03/2021	11	94	Morrinhos	P04	Culicidae	Culicinae	*Uranotaenia*	*Uranotaenia sp.*	1
2021	Março	15/03/2021	11	94	Teotônio	P03	Culicidae	Anophelinae	*Anopheles*	*Anopheles sp.*	2
2021	Março	15/03/2021	11	94	Teotônio	P03	Culicidae	Culicinae	*Mansonia*	*Mansonia sp.*	7
2021	Março	15/03/2021	11	94	Teotônio	P03	Culicidae	Culicinae	*Uranotaenia*	*Uranotaenia sp.*	1
2021	Março	15/03/2021	11	94	Teotônio	P03	Culicidae	Culicinae	*Culex*	*Culex sp.*	2
2021	Março	15/03/2021	11	94	Cujubim Grande	P01	Culicidae	Culicinae	*Culex*	*Culex sp.*	4
2021	Março	22/03/2021	12	95	Rio Contra	P07	Culicidae	Culicinae	*Mansonia*	*Mansonia sp.*	18
2021	Março	22/03/2021	12	95	Samauma	P08	Culicidae	Culicinae	*Mansonia*	*Mansonia sp.*	2
2021	Março	22/03/2021	12	95	Samauma	P08	Culicidae	Culicinae	*Culex*	*Culex sp.*	1
2021	Março	22/03/2021	12	95	Jaci Paraná	P06	Culicidae	Culicinae	*Culex*	*Culex sp.*	11
2021	Março	22/03/2021	12	95	Jaci Paraná	P06	Culicidae	Culicinae	*Mansonia*	*Mansonia sp.*	8
2021	Março	22/03/2021	12	95	Jaci Paraná	P06	Culicidae	Culicinae	*Aedes*	*Aedes sp.*	5
2021	Março	22/03/2021	12	95	Santa Rita	P05	Culicidae	Culicinae	*Mansonia*	*Mansonia sp.*	4
2021	Março	22/03/2021	12	95	Morrinhos	P04	Culicidae	Culicinae	*Mansonia*	*Mansonia sp.*	3
2021	Março	22/03/2021	12	95	Teotônio	P03	Culicidae	Culicinae	*Coquillettidia*	*Coquillettidia sp.*	2
2021	Março	22/03/2021	12	95	Teotônio	P03	Culicidae	Culicinae	*Psorophora*	*Psorophora sp.*	2
2021	Março	22/03/2021	12	95	Teotônio	P03	Culicidae	Culicinae	*Mansonia*	*Mansonia sp.*	5
2021	Março	22/03/2021	12	95	São Domingos	P02	Culicidae	Culicinae	*Coquillettidia*	*Coquillettidia sp.*	2
2021	Março	22/03/2021	12	95	São Domingos	P02	Culicidae	Culicinae	*Mansonia*	*Mansonia sp.*	5
2021	Março	22/03/2021	12	95	São Domingos	P02	Culicidae	Culicinae	*Uranotaenia*	*Uranotaenia sp.*	3
2021	Março	22/03/2021	12	95	São Domingos	P02	Culicidae	Culicinae	*Aedeomyia*	*Adeomyia squamipennis*	1
2021	Março	22/03/2021	12	95	Cujubim Grande	P01	Culicidae	Culicinae	*Aedes*	*Aedes aegypti*	3
2021	Março	22/03/2021	12	95	Cujubim Grande	P01	Culicidae	Culicinae	*Culex*	*Culex sp.*	5
2021	Março	22/03/2021	12	95	Cujubim Grande	P01	Culicidae	Culicinae	*Mansonia*	*Mansonia sp.*	4
2021	Março	29/03/2021	13	96	Rio Contra	P07	Culicidae	Anophelinae	*Anopheles*	*Anopheles darlingi*	12
2021	Março	29/03/2021	13	96	Rio Contra	P07	Culicidae	Culicinae	*Mansonia*	*Mansonia sp.*	45

2021	Março	29/03/2021	13	96	Samauma	P08	Culicidae	Culicinae	*Mansonia*	*Mansonia sp.*	111
2021	Março	29/03/2021	13	96	Samauma	P08	Culicidae	Culicinae	*Psorophora*	*Psorophora sp.*	2
2021	Março	29/03/2021	13	96	Samauma	P08	Culicidae	Culicinae	*Culex*	*Culex sp.*	28
2021	Março	29/03/2021	13	96	Samauma	P08	Culicidae	Culicinae	*Coquillettidia*	*Coquillettidia sp.*	5
2021	Março	29/03/2021	13	96	Samauma	P08	Culicidae	Culicinae	*Aedeomyia*	*Adeomyia squamipennis*	3
2021	Março	29/03/2021	13	96	Samauma	P08	Culicidae	Anophelinae	*Anopheles*	*Anopheles sp.*	2
2021	Março	29/03/2021	13	96	Samauma	P08	Culicidae	Culicinae	*Limatus*	*Limatus durhamii*	1
2021	Março	29/03/2021	13	96	Jaci Paraná	P06	Culicidae	Culicinae	*Mansonia*	*Mansonia sp.*	5
2021	Março	29/03/2021	13	96	Jaci Paraná	P06	Culicidae	Culicinae	*Aedes*	*Aedes sp.*	8
2021	Março	29/03/2021	13	96	Santa Rita	P05	Culicidae	Culicinae	*Mansonia*	*Mansonia sp.*	9
2021	Março	29/03/2021	13	96	Morrinhos	P04	Culicidae	Culicinae	*Mansonia*	*Mansonia sp.*	28
2021	Março	29/03/2021	13	96	Morrinhos	P04	Culicidae	Culicinae	*Culex*	*Culex sp.*	12
2021	Março	29/03/2021	13	96	Teotônio	P03	Culicidae	Anophelinae	*Anopheles*	*Anopheles sp.*	2
2021	Março	29/03/2021	13	96	Teotônio	P03	Culicidae	Culicinae	*Mansonia*	*Mansonia sp.*	15
2021	Março	29/03/2021	13	96	Teotônio	P03	Culicidae	Culicinae	*Aedeomyia*	*Adeomyia squamipennis*	3
2021	Março	29/03/2021	13	96	Teotônio	P03	Culicidae	Culicinae	*Coquillettidia*	*Coquillettidia sp.*	6
2021	Março	29/03/2021	13	96	Teotônio	P03	Culicidae	Culicinae	*Uranotaenia*	*Uranotaenia sp.*	2
2021	Março	29/03/2021	13	96	São Domingos	P02	Culicidae	Anophelinae	*Anopheles*	*Anopheles sp.*	5
2021	Março	29/03/2021	13	96	São Domingos	P02	Culicidae	Culicinae	*Coquillettidia*	*Coquillettidia sp.*	8
2021	Março	29/03/2021	13	96	São Domingos	P02	Culicidae	Culicinae	*Mansonia*	*Mansonia sp.*	35
2021	Março	29/03/2021	13	96	São Domingos	P02	Culicidae	Culicinae	*Aedeomyia*	*Adeomyia squamipennis*	2
2021	Março	29/03/2021	13	96	Cujubim Grande	P01	Culicidae	Culicinae	*Culex*	*Culex sp.*	3
2021	Abril	05/04/2021	14	97	Rio Contra	P07	Culicidae	Culicinae	*Mansonia*	*Mansonia sp.*	12
2021	Abril	05/04/2021	14	97	Rio Contra	P07	Culicidae	Anophelinae	*Anopheles*	*Anopheles darlingi*	13
2021	Abril	05/04/2021	14	97	Samauma	P08	Culicidae	Culicinae	*Mansonia*	*Mansonia sp.*	248
2021	Abril	05/04/2021	14	97	Samauma	P08	Culicidae	Culicinae	*Coquillettidia*	*Coquillettidia sp.*	6
2021	Abril	05/04/2021	14	97	Jaci Paraná	P06	Culicidae	Culicinae	*Aedes*	*Aedes sp.*	3
2021	Abril	05/04/2021	14	97	Jaci Paraná	P06	Culicidae	Culicinae	*Mansonia*	*Mansonia sp.*	22
2021	Abril	05/04/2021	14	97	Jaci Paraná	P06	Culicidae	Culicinae	*Culex*	*Culex sp.*	25
2021	Abril	05/04/2021	14	97	Jaci Paraná	P06	Culicidae	Anophelinae	*Anopheles*	*Anopheles sp.*	3
2021	Abril	05/04/2021	14	97	Santa Rita	P05	Culicidae	Culicinae	*Mansonia*	*Mansonia sp.*	3
2021	Abril	05/04/2021	14	97	Morrinhos	P04	Culicidae	Culicinae	*Mansonia*	*Mansonia sp.*	3
2021	Abril	05/04/2021	14	97	Morrinhos	P04	Culicidae	Culicinae	*Culex*	*Culex sp.*	12
2021	Abril	05/04/2021	14	97	Morrinhos	P04	Culicidae	Culicinae	*Limatus*	*Limatus durhamii*	2
2021	Abril	05/04/2021	14	97	Teotônio	P03	Culicidae	Culicinae	*Mansonia*	*Mansonia sp.*	6
2021	Abril	05/04/2021	14	97	Teotônio	P03	Culicidae	Culicinae	*Coquillettidia*	*Coquillettidia sp.*	7
2021	Abril	05/04/2021	14	97	São Domingos	P02	Culicidae	Culicinae	*Mansonia*	*Mansonia sp.*	7
2021	Abril	05/04/2021	14	97	São Domingos	P02	Culicidae	Culicinae	*Coquillettidia*	*Coquillettidia sp.*	2
2021	Abril	05/04/2021	14	97	São Domingos	P02	Culicidae	Culicinae	*Culex*	*Culex sp.*	3
2021	Abril	05/04/2021	14	97	Cujubim Grande	P01	Culicidae	Culicinae	*Culex*	*Culex sp.*	5
2021	Abril	12/04/2021	15	98	Rio Contra	P07	Culicidae	Anophelinae	*Anopheles*	*Anopheles darlingi*	18
2021	Abril	12/04/2021	15	98	Rio Contra	P07	Culicidae	Culicinae	*Mansonia*	*Mansonia sp.*	23
2021	Abril	12/04/2021	15	98	Rio Contra	P07	Culicidae	Culicinae	*Culex*	*Culex sp.*	5
2021	Abril	12/04/2021	15	98	Samauma	P08	Culicidae	Culicinae	*Mansonia*	*Mansonia sp.*	153
2021	Abril	12/04/2021	15	98	Samauma	P08	Culicidae	Culicinae	*Culex*	*Culex sp.*	12
2021	Abril	12/04/2021	15	98	Samauma	P08	Culicidae	Culicinae	*Coquillettidia*	*Coquillettidia sp.*	3
2021	Abril	12/04/2021	15	98	Jaci Paraná	P06	Culicidae	Culicinae	*Culex*	*Culex sp.*	23
2021	Abril	12/04/2021	15	98	Jaci Paraná	P06	Culicidae	Culicinae	*Mansonia*	*Mansonia sp.*	12
2021	Abril	12/04/2021	15	98	Santa Rita	P05	Culicidae	Culicinae	*Mansonia*	*Mansonia sp.*	4
2021	Abril	12/04/2021	15	98	Morrinhos	P04	Culicidae	Culicinae	*Mansonia*	*Mansonia sp.*	5
2021	Abril	12/04/2021	15	98	Morrinhos	P04	Culicidae	Culicinae	*Coquillettidia*	*Coquillettidia sp.*	2
2021	Abril	12/04/2021	15	98	Morrinhos	P04	Culicidae	Culicinae	*Culex*	*Culex sp.*	1
2021	Abril	12/04/2021	15	98	Teotônio	P03	Culicidae	Culicinae	*Mansonia*	*Mansonia sp.*	10
2021	Abril	12/04/2021	15	98	Teotônio	P03	Culicidae	Culicinae	*Coquillettidia*	*Coquillettidia sp.*	7
2021	Abril	12/04/2021	15	98	São Domingos	P02	Culicidae	Anophelinae	*Anopheles*	*Anopheles sp.*	3
2021	Abril	12/04/2021	15	98	São Domingos	P02	Culicidae	Culicinae	*Culex*	*Culex sp.*	5
2021	Abril	12/04/2021	15	98	São Domingos	P02	Culicidae	Culicinae	*Mansonia*	*Mansonia sp.*	12
2021	Abril	12/04/2021	15	98	São Domingos	P02	Culicidae	Culicinae	*Coquillettidia*	*Coquillettidia sp.*	3
2021	Abril	12/04/2021	15	98	Cujubim Grande	P01	Culicidae	Anophelinae	*Anopheles*	*Anopheles sp.*	2
2021	Abril	12/04/2021	15	98	Cujubim Grande	P01	Culicidae	Culicinae	*Mansonia*	*Mansonia sp.*	5
2021	Abril	12/04/2021	15	98	Cujubim Grande	P01	Culicidae	Culicinae	*Culex*	*Culex sp.*	6
2021	Abril	12/04/2021	15	98	Cujubim Grande	P01	Culicidae	Culicinae	*Aedes*	*Aedes sp.*	3
2021	Abril	19/04/2021	16	99	Rio Contra	P07	Culicidae	Culicinae	*Mansonia*	*Mansonia sp.*	45
2021	Abril	19/04/2021	16	99	Rio Contra	P07	Culicidae	Anophelinae	*Anopheles*	*Anopheles sp.*	3
2021	Abril	19/04/2021	16	99	Rio Contra	P07	Culicidae	Anophelinae	*Anopheles*	*Anopheles darlingi*	2
2021	Abril	19/04/2021	16	99	Rio Contra	P07	Culicidae	Culicinae	*Culex*	*Culex sp.*	7
2021	Abril	19/04/2021	16	99	Samauma	P08	Culicidae	Culicinae	*Mansonia*	*Mansonia sp.*	63
2021	Abril	19/04/2021	16	99	Jaci Paraná	P06	Culicidae	Anophelinae	*Anopheles*	*Anopheles sp.*	2
2021	Abril	19/04/2021	16	99	Jaci Paraná	P06	Culicidae	Culicinae	*Mansonia*	*Mansonia sp.*	14
2021	Abril	19/04/2021	16	99	Jaci Paraná	P06	Culicidae	Culicinae	*Culex*	*Culex sp.*	3
2021	Abril	19/04/2021	16	99	Santa Rita	P05	Culicidae	Anophelinae	*Anopheles*	*Anopheles sp.*	1
2021	Abril	19/04/2021	16	99	Santa Rita	P05	Culicidae	Culicinae	*Mansonia*	*Mansonia sp.*	3
2021	Abril	19/04/2021	16	99	Santa Rita	P05	Culicidae	Culicinae	*Culex*	*Culex sp.*	1
2021	Abril	19/04/2021	16	99	Morrinhos	P04	Culicidae	Culicinae	*Culex*	*Culex sp.*	3
2021	Abril	19/04/2021	16	99	Morrinhos	P04	Culicidae	Culicinae	*Mansonia*	*Mansonia sp.*	22
2021	Abril	19/04/2021	16	99	Morrinhos	P04	Culicidae	Culicinae	*Aedes*	*Aedes sp.*	2
2021	Abril	19/04/2021	16	99	Teotônio	P03	Culicidae	Culicinae	*Mansonia*	*Mansonia sp.*	2
2021	Abril	19/04/2021	16	99	São Domingos	P02	Culicidae	Culicinae	*Aedes*	*Aedes sp.*	2
2021	Abril	19/04/2021	16	99	São Domingos	P02	Culicidae	Culicinae	*Mansonia*	*Mansonia sp.*	5
2021	Abril	19/04/2021	16	99	São Domingos	P02	Culicidae	Culicinae	*Culex*	*Culex sp.*	3
2021	Abril	19/04/2021	16	99	São Domingos	P02	Culicidae	Culicinae	*Coquillettidia*	*Coquillettidia sp.*	4
2021	Abril	19/04/2021	16	99	São Domingos	P02	Culicidae	Anophelinae	*Anopheles*	*Anopheles sp.*	2
2021	Abril	19/04/2021	16	99	Cujubim Grande	P01	Culicidae	Culicinae	*Culex*	*Culex sp.*	7
2021	Abril	19/04/2021	16	99	Cujubim Grande	P01	Culicidae	Culicinae	*Mansonia*	*Mansonia sp.*	2
2021	Abril	26/04/2021	17	100	Rio Contra	P07	Culicidae	Anophelinae	*Anopheles*	*Anopheles sp.*	2
2021	Abril	26/04/2021	17	100	Rio Contra	P07	Culicidae	Culicinae	*Mansonia*	*Mansonia sp.*	30
2021	Abril	26/04/2021	17	100	Rio Contra	P07	Culicidae	Culicinae	*Culex*	*Culex sp.*	1
2021	Abril	26/04/2021	17	100	Samauma	P08	Culicidae	Culicinae	*Aedeomyia*	*Adeomyia squamipennis*	3
2021	Abril	26/04/2021	17	100	Samauma	P08	Culicidae	Culicinae	*Culex*	*Culex sp.*	7
2021	Abril	26/04/2021	17	100	Samauma	P08	Culicidae	Culicinae	*Coquillettidia*	*Coquillettidia sp.*	5
2021	Abril	26/04/2021	17	100	Samauma	P08	Culicidae	Anophelinae	*Anopheles*	*Anopheles sp.*	2
2021	Abril	26/04/2021	17	100	Samauma	P08	Culicidae	Culicinae	*Mansonia*	*Mansonia sp.*	93
2021	Abril	26/04/2021	17	100	Jaci Paraná	P06	Culicidae	Anophelinae	*Anopheles*	*Anopheles sp.*	2
2021	Abril	26/04/2021	17	100	Jaci Paraná	P06	Culicidae	Culicinae	*Mansonia*	*Mansonia sp.*	10
2021	Abril	26/04/2021	17	100	Jaci Paraná	P06	Culicidae	Culicinae	*Aedes*	*Aedes sp.*	5
2021	Abril	26/04/2021	17	100	Jaci Paraná	P06	Culicidae	Culicinae	*Culex*	*Culex sp.*	3
2021	Abril	26/04/2021	17	100	Santa Rita	P05	Culicidae	Culicinae	*Mansonia*	*Mansonia sp.*	4
2021	Abril	26/04/2021	17	100	Morrinhos	P04	Culicidae	Culicinae	*Aedes*	*Aedes sp.*	1
2021	Abril	26/04/2021	17	100	Morrinhos	P04	Culicidae	Culicinae	*Mansonia*	*Mansonia sp.*	11
2021	Abril	26/04/2021	17	100	Morrinhos	P04	Culicidae	Culicinae	*Culex*	*Culex sp.*	6
2021	Abril	26/04/2021	17	100	Teotônio	P03	Culicidae	Culicinae	*Mansonia*	*Mansonia sp.*	2
2021	Abril	26/04/2021	17	100	Teotônio	P03	Culicidae	Culicinae	*Coquillettidia*	*Coquillettidia sp.*	2
2021	Abril	26/04/2021	17	100	São Domingos	P02	Culicidae	Anophelinae	*Anopheles*	*Anopheles sp.*	2
2021	Abril	26/04/2021	17	100	São Domingos	P02	Culicidae	Culicinae	*Culex*	*Culex sp.*	1
2021	Abril	26/04/2021	17	100	São Domingos	P02	Culicidae	Culicinae	*Coquillettidia*	*Coquillettidia sp.*	5
2021	Abril	26/04/2021	17	100	São Domingos	P02	Culicidae	Culicinae	*Limatus*	*Limatus durhamii*	3
2021	Abril	26/04/2021	17	100	São Domingos	P02	Culicidae	Culicinae	*Mansonia*	*Mansonia sp.*	6
2021	Abril	26/04/2021	17	100	Cujubim Grande	P01	Culicidae	Culicinae	*Culex*	*Culex sp.*	2
2021	Maio	03/05/2021	18	101	Rio Contra	P07	Culicidae	Anophelinae	*Anopheles*	*Anopheles darlingi*	6
2021	Maio	03/05/2021	18	101	Rio Contra	P07	Culicidae	Culicinae	*Culex*	*Culex sp.*	1
2021	Maio	03/05/2021	18	101	Rio Contra	P07	Culicidae	Culicinae	*Mansonia*	*Mansonia sp.*	12
2021	Maio	03/05/2021	18	101	Samauma	P08	Culicidae	Culicinae	*Coquillettidia*	*Coquillettidia sp.*	10
2021	Maio	03/05/2021	18	101	Samauma	P08	Culicidae	Culicinae	*Mansonia*	*Mansonia sp.*	83
2021	Maio	03/05/2021	18	101	Samauma	P08	Culicidae	Culicinae	*Aedeomyia*	*Adeomyia squamipennis*	7
2021	Maio	03/05/2021	18	101	Samauma	P08	Culicidae	Culicinae	*Culex*	*Culex sp.*	5
2021	Maio	03/05/2021	18	101	Jaci Paraná	P06	Culicidae	Culicinae	*Mansonia*	*Mansonia sp.*	8
2021	Maio	03/05/2021	18	101	Jaci Paraná	P06	Culicidae	Culicinae	*Aedes*	*Aedes sp.*	3
2021	Maio	03/05/2021	18	101	Jaci Paraná	P06	Culicidae	Culicinae	*Aedeomyia*	*Adeomyia squamipennis*	2

2021	Maio	03/05/2021	18	101	Santa Rita	P05	Culicidae	Culicinae	*Mansonia*	*Mansonia sp.*	9
2021	Maio	03/05/2021	18	101	Morrinhos	P04	Culicidae	Culicinae	*Mansonia*	*Mansonia sp.*	23
2021	Maio	03/05/2021	18	101	Morrinhos	P04	Culicidae	Culicinae	*Culex*	*Culex sp.*	6
2021	Maio	03/05/2021	18	101	Teotônio	P03	Culicidae	Culicinae	*Aedes*	*Aedes sp.*	2
2021	Maio	03/05/2021	18	101	Teotônio	P03	Culicidae	Culicinae	*Coquillettidia*	*Coquillettidia sp.*	15
2021	Maio	03/05/2021	18	101	Teotônio	P03	Culicidae	Culicinae	*Mansonia*	*Mansonia sp.*	2
2021	Maio	03/05/2021	18	101	Teotônio	P03	Culicidae	Culicinae	*Culex*	*Culex sp.*	1
2021	Maio	03/05/2021	18	101	São Domingos	P02	Culicidae	Culicinae	*Mansonia*	*Mansonia sp.*	5
2021	Maio	03/05/2021	18	101	Cujubim Grande	P01	Culicidae	Culicinae	*Culex*	*Culex sp.*	2
2021	Maio	10/05/2021	19	102	Rio Contra	P07	Culicidae	Anophelinae	*Anopheles*	*Anopheles darlingi*	2
2021	Maio	10/05/2021	19	102	Rio Contra	P07	Culicidae	Culicinae	*Mansonia*	*Mansonia sp.*	4
2021	Maio	10/05/2021	19	102	Rio Contra	P07	Culicidae	Culicinae	*Coquillettidia*	*Coquillettidia shannoni*	2
2021	Maio	10/05/2021	19	102	Samauma	P08	Culicidae	Culicinae	*Mansonia*	*Mansonia sp.*	72
2021	Maio	10/05/2021	19	102	Samauma	P08	Culicidae	Culicinae	*Coquillettidia*	*Coquillettidia sp.*	8
2021	Maio	10/05/2021	19	102	Samauma	P08	Culicidae	Culicinae	*Culex*	*Culex sp.*	7
2021	Maio	10/05/2021	19	102	Samauma	P08	Culicidae	Culicinae	*Aedeomyia*	*Adeomyia squamipennis*	4
2021	Maio	10/05/2021	19	102	Jaci Paraná	P06	Culicidae	Anophelinae	*Anopheles*	*Anopheles sp.*	3
2021	Maio	10/05/2021	19	102	Jaci Paraná	P06	Culicidae	Culicinae	*Aedeomyia*	*Adeomyia squamipennis*	4
2021	Maio	10/05/2021	19	102	Jaci Paraná	P06	Culicidae	Culicinae	*Mansonia*	*Mansonia sp.*	2
2021	Maio	10/05/2021	19	102	Jaci Paraná	P06	Culicidae	Culicinae	*Uranotaenia*	*Uranotaenia sp.*	1
2021	Maio	10/05/2021	19	102	Santa Rita	P05	Culicidae	Culicinae	*Culex*	*Culex sp.*	3
2021	Maio	10/05/2021	19	102	Santa Rita	P05	Culicidae	Culicinae	*Mansonia*	*Mansonia sp.*	1
2021	Maio	10/05/2021	19	102	Morrinhos	P04	Culicidae	Culicinae	*Mansonia*	*Mansonia sp.*	28
2021	Maio	10/05/2021	19	102	Morrinhos	P04	Culicidae	Culicinae	*Culex*	*Culex sp.*	15
2021	Maio	10/05/2021	19	102	Teotônio	P03	Culicidae	Culicinae	*Coquillettidia*	*Coquillettidia sp.*	6
2021	Maio	10/05/2021	19	102	Teotônio	P03	Culicidae	Culicinae	*Mansonia*	*Mansonia sp.*	1
2021	Maio	10/05/2021	19	102	Teotônio	P03	Culicidae	Culicinae	*Culex*	*Culex sp.*	2
2021	Maio	10/05/2021	19	102	São Domingos	P02	Culicidae	Culicinae	*Mansonia*	*Mansonia sp.*	4
2021	Maio	10/05/2021	19	102	São Domingos	P02	Culicidae	Culicinae	*Culex*	*Culex sp.*	1
2021	Maio	10/05/2021	19	102	Cujubim Grande	P01	Culicidae	Culicinae	*Culex*	*Culex sp.*	4
2021	Maio	10/05/2021	19	102	Cujubim Grande	P01	Culicidae	Culicinae	*Mansonia*	*Mansonia sp.*	2
2021	Maio	17/05/2021	20	103	Rio Contra	P07	Culicidae	Culicinae	*Mansonia*	*Mansonia sp.*	33
2021	Maio	17/05/2021	20	103	Rio Contra	P07	Culicidae	Anophelinae	*Anopheles*	*Anopheles darlingi*	10
2021	Maio	17/05/2021	20	103	Rio Contra	P07	Culicidae	Culicinae	*Culex*	*Culex sp.*	2
2021	Maio	17/05/2021	20	103	Samauma	P08	Culicidae	Culicinae	*Mansonia*	*Mansonia sp.*	57
2021	Maio	17/05/2021	20	103	Samauma	P08	Culicidae	Culicinae	*Culex*	*Culex sp.*	12
2021	Maio	17/05/2021	20	103	Samauma	P08	Culicidae	Culicinae	*Aedeomyia*	*Adeomyia squamipennis*	3
2021	Maio	17/05/2021	20	103	Samauma	P08	Culicidae	Culicinae	*Coquillettidia*	*Coquillettidia sp.*	4
2021	Maio	17/05/2021	20	103	Jaci Paraná	P06	Culicidae	Culicinae	*Mansonia*	*Mansonia sp.*	5
2021	Maio	17/05/2021	20	103	Jaci Paraná	P06	Culicidae	Culicinae	*Aedes*	*Aedes aegypti*	11
2021	Maio	17/05/2021	20	103	Jaci Paraná	P06	Culicidae	Culicinae	*Culex*	*Culex sp.*	21
2021	Maio	17/05/2021	20	103	Jaci Paraná	P06	Culicidae	Anophelinae	*Anopheles*	*Anopheles sp.*	3
2021	Maio	17/05/2021	20	103	Santa Rita	P05	Culicidae	Culicinae	*Mansonia*	*Mansonia sp.*	8
2021	Maio	17/05/2021	20	103	Santa Rita	P05	Culicidae	Culicinae	*Culex*	*Culex sp.*	3
2021	Maio	17/05/2021	20	103	Morrinhos	P04	Culicidae	Culicinae	*Mansonia*	*Mansonia sp.*	21
2021	Maio	17/05/2021	20	103	Morrinhos	P04	Culicidae	Culicinae	*Culex*	*Culex sp.*	3
2021	Maio	17/05/2021	20	103	Morrinhos	P04	Culicidae	Culicinae	*Limatus*	*Limatus durhamii*	1
2021	Maio	17/05/2021	20	103	Teotônio	P03	Culicidae	Culicinae	*Mansonia*	*Mansonia sp.*	5
2021	Maio	17/05/2021	20	103	Teotônio	P03	Culicidae	Culicinae	*Coquillettidia*	*Coquillettidia sp.*	5
2021	Maio	17/05/2021	20	103	Teotônio	P03	Culicidae	Culicinae	*Culex*	*Culex sp.*	1
2021	Maio	17/05/2021	20	103	São Domingos	P02	Culicidae	Culicinae	*Coquillettidia*	*Coquillettidia sp.*	2
2021	Maio	17/05/2021	20	103	São Domingos	P02	Culicidae	Culicinae	*Culex*	*Culex sp.*	3
2021	Maio	17/05/2021	20	103	São Domingos	P02	Culicidae	Culicinae	*Mansonia*	*Mansonia sp.*	7
2021	Maio	17/05/2021	20	103	Cujubim Grande	P01	Culicidae	Culicinae	*Culex*	*Culex sp.*	7
2021	Maio	17/05/2021	20	103	Cujubim Grande	P01	Culicidae	Culicinae	*Mansonia*	*Mansonia sp.*	1
2021	Maio	24/05/2021	21	104	Rio Contra	P07	Culicidae	Culicinae	*Mansonia*	*Mansonia sp.*	27
2021	Maio	24/05/2021	21	104	Rio Contra	P07	Culicidae	Anophelinae	*Anopheles*	*Anopheles darlingi*	8
2021	Maio	24/05/2021	21	104	Rio Contra	P07	Culicidae	Culicinae	*Culex*	*Culex sp.*	5
2021	Maio	24/05/2021	21	104	Samauma	P08	Culicidae	Culicinae	*Mansonia*	*Mansonia sp.*	72
2021	Maio	24/05/2021	21	104	Samauma	P08	Culicidae	Culicinae	*Culex*	*Culex sp.*	8
2021	Maio	24/05/2021	21	104	Samauma	P08	Culicidae	Culicinae	*Coquillettidia*	*Coquillettidia sp.*	4
2021	Maio	24/05/2021	21	104	Samauma	P08	Culicidae	Culicinae	*Limatus*	*Limatus durhamii*	1
2021	Maio	24/05/2021	21	104	Jaci Paraná	P06	Culicidae	Culicinae	*Mansonia*	*Mansonia sp.*	9
2021	Maio	24/05/2021	21	104	Jaci Paraná	P06	Culicidae	Culicinae	*Aedes*	*Aedes aegypti*	11
2021	Maio	24/05/2021	21	104	Jaci Paraná	P06	Culicidae	Culicinae	*Culex*	*Culex sp.*	3
2021	Maio	24/05/2021	21	104	Santa Rita	P05	Culicidae	Culicinae	*Mansonia*	*Mansonia sp.*	10
2021	Maio	24/05/2021	21	104	Santa Rita	P05	Culicidae	Culicinae	*Culex*	*Culex sp.*	2
2021	Maio	24/05/2021	21	104	Morrinhos	P04	Culicidae	Culicinae	*Mansonia*	*Mansonia sp.*	53
2021	Maio	24/05/2021	21	104	Morrinhos	P04	Culicidae	Culicinae	*Culex*	*Culex sp.*	8
2021	Maio	24/05/2021	21	104	Morrinhos	P04	Culicidae	Culicinae	*Aedes*	*Aedes sp.*	8
2021	Maio	24/05/2021	21	104	Teotônio	P03	Culicidae	Culicinae	*Coquillettidia*	*Coquillettidia sp.*	7
2021	Maio	24/05/2021	21	104	Teotônio	P03	Culicidae	Culicinae	*Mansonia*	*Mansonia humeralis*	5
2021	Maio	24/05/2021	21	104	Teotônio	P03	Culicidae	Culicinae	*Culex*	*Culex sp.*	2
2021	Maio	24/05/2021	21	104	São Domingos	P02	Culicidae	Culicinae	*Coquillettidia*	*Coquillettidia sp.*	5
2021	Maio	24/05/2021	21	104	São Domingos	P02	Culicidae	Culicinae	*Culex*	*Culex sp.*	1
2021	Maio	24/05/2021	21	104	São Domingos	P02	Culicidae	Culicinae	*Mansonia*	*Mansonia sp.*	18
2021	Maio	24/05/2021	21	104	Cujubim Grande	P01	Culicidae	Culicinae	*Culex*	*Culex sp.*	3
2021	Maio	24/05/2021	21	104	Cujubim Grande	P01	Culicidae	Culicinae	*Mansonia*	*Mansonia sp.*	1
2021	Maio	31/05/2021	22	105	Rio Contra	P07	Culicidae	Culicinae	*Mansonia*	*Mansonia sp.*	28
2021	Maio	31/05/2021	22	105	Rio Contra	P07	Culicidae	Culicinae	*Culex*	*Culex sp.*	10
2021	Maio	31/05/2021	22	105	Rio Contra	P07	Culicidae	Anophelinae	*Anopheles*	*Anopheles darlingi*	5
2021	Maio	31/05/2021	22	105	Samauma	P08	Culicidae	Culicinae	*Mansonia*	*Mansonia sp.*	45
2021	Maio	31/05/2021	22	105	Samauma	P08	Culicidae	Culicinae	*Culex*	*Culex sp.*	12
2021	Maio	31/05/2021	22	105	Samauma	P08	Culicidae	Culicinae	*Limatus*	*Limatus durhamii*	2
2021	Maio	31/05/2021	22	105	Samauma	P08	Culicidae	Culicinae	*Aedeomyia*	*Adeomyia squamipennis*	1
2021	Maio	31/05/2021	22	105	Jaci Paraná	P06	Culicidae	Culicinae	*Aedes*	*Aedes aegypti*	2
2021	Maio	31/05/2021	22	105	Jaci Paraná	P06	Culicidae	Culicinae	*Mansonia*	*Mansonia sp.*	7
2021	Maio	31/05/2021	22	105	Jaci Paraná	P06	Culicidae	Culicinae	*Culex*	*Culex sp.*	3
2021	Maio	31/05/2021	22	105	Santa Rita	P05	Culicidae	Culicinae	*Mansonia*	*Mansonia sp.*	8
2021	Maio	31/05/2021	22	105	Santa Rita	P05	Culicidae	Culicinae	*Culex*	*Culex sp.*	3
2021	Maio	31/05/2021	22	105	Morrinhos	P04	Culicidae	Culicinae	*Mansonia*	*Mansonia sp.*	28
2021	Maio	31/05/2021	22	105	Morrinhos	P04	Culicidae	Culicinae	*Culex*	*Culex sp.*	2
2021	Maio	31/05/2021	22	105	Teotônio	P03	Culicidae	Culicinae	*Mansonia*	*Mansonia sp.*	5
2021	Maio	31/05/2021	22	105	Teotônio	P03	Culicidae	Culicinae	*Coquillettidia*	*Coquillettidia sp.*	10
2021	Maio	31/05/2021	22	105	Teotônio	P03	Culicidae	Culicinae	*Culex*	*Culex sp.*	1
2021	Maio	31/05/2021	22	105	São Domingos	P02	Culicidae	Culicinae	*Mansonia*	*Mansonia sp.*	5
2021	Maio	31/05/2021	22	105	São Domingos	P02	Culicidae	Culicinae	*Culex*	*Culex sp.*	2
2021	Maio	31/05/2021	22	105	São Domingos	P02	Culicidae	Culicinae	*Coquillettidia*	*Coquillettidia sp.*	3
2021	Maio	31/05/2021	22	105	Cujubim Grande	P01	Culicidae	Culicinae	*Culex*	*Culex sp.*	8
2021	Maio	31/05/2021	22	105	Cujubim Grande	P01	Culicidae	Culicinae	*Mansonia*	*Mansonia sp.*	5
2021	Maio	31/05/2021	22	105	Cujubim Grande	P01	Culicidae	Culicinae	*Aedes*	*Aedes sp.*	1
2021	Junho	07/06/2021	23	106	Rio Contra	P07	Culicidae	Culicinae	*Mansonia*	*Mansonia sp.*	4
2021	Junho	07/06/2021	23	106	Rio Contra	P07	Culicidae	Anophelinae	*Anopheles*	*Anopheles darlingi*	1
2021	Junho	07/06/2021	23	106	Samauma	P08	Culicidae	Culicinae	*Mansonia*	*Mansonia sp.*	53
2021	Junho	07/06/2021	23	106	Samauma	P08	Culicidae	Culicinae	*Culex*	*Culex sp.*	12
2021	Junho	07/06/2021	23	106	Jaci Paraná	P06	Culicidae	Culicinae	*Mansonia*	*Mansonia sp.*	10
2021	Junho	07/06/2021	23	106	Jaci Paraná	P06	Culicidae	Culicinae	*Aedes*	*Aedes aegypti*	5
2021	Junho	07/06/2021	23	106	Jaci Paraná	P06	Culicidae	Culicinae	*Culex*	*Culex sp.*	3
2021	Junho	07/06/2021	23	106	Santa Rita	P05	Culicidae	Culicinae	*Culex*	*Culex sp.*	1
2021	Junho	07/06/2021	23	106	Santa Rita	P05	Culicidae	Culicinae	*Mansonia*	*Mansonia sp.*	11
2021	Junho	07/06/2021	23	106	Morrinhos	P04	Culicidae	Culicinae	*Mansonia*	*Mansonia sp.*	37
2021	Junho	07/06/2021	23	106	Teotônio	P03	Culicidae	Culicinae	*Mansonia*	*Mansonia sp.*	1
2021	Junho	07/06/2021	23	106	São Domingos	P02	Culicidae	Culicinae	*Mansonia*	*Mansonia sp.*	17
2021	Junho	07/06/2021	23	106	São Domingos	P02	Culicidae	Culicinae	*Culex*	*Culex sp.*	4
2021	Junho	07/06/2021	23	106	São Domingos	P02	Culicidae	Culicinae	*Coquillettidia*	*Coquillettidia sp.*	2
2021	Junho	07/06/2021	23	106	Cujubim Grande	P01	Culicidae	Culicinae	*Culex*	*Culex sp.*	3
2021	Junho	07/06/2021	23	106	Cujubim Grande	P01	Culicidae	Culicinae	*Mansonia*	*Mansonia sp.*	7
2021	Junho	07/06/2021	23	106	Cujubim Grande	P01	Culicidae	Culicinae	*Limatus*	*Limatus durhamii*	1

2021	Junho	14/06/2021	24	107	Rio Contra	P07	Culicidae	Anophelinae	*Anopheles*	*Anopheles darlingi*	10
2021	Junho	14/06/2021	24	107	Rio Contra	P07	Culicidae	Culicinae	*Mansonia*	*Mansonia sp.*	8
2021	Junho	14/06/2021	24	107	Rio Contra	P07	Culicidae	Culicinae	*Culex*	*Culex sp.*	2
2021	Junho	14/06/2021	24	107	Samauma	P08	Culicidae	Culicinae	*Mansonia*	*Mansonia sp.*	69
2021	Junho	14/06/2021	24	107	Samauma	P08	Culicidae	Culicinae	*Culex*	*Culex sp.*	10
2021	Junho	14/06/2021	24	107	Jaci Paraná	P06	Culicidae	Anophelinae	*Anopheles*	*Anopheles sp.*	2
2021	Junho	14/06/2021	24	107	Jaci Paraná	P06	Culicidae	Culicinae	*Mansonia*	*Mansonia sp.*	9
2021	Junho	14/06/2021	24	107	Jaci Paraná	P06	Culicidae	Culicinae	*Aedes*	*Aedes sp.*	3
2021	Junho	14/06/2021	24	107	Santa Rita	P05	Culicidae	Culicinae	*Mansonia*	*Mansonia sp.*	43
2021	Junho	14/06/2021	24	107	Morrinhos	P04	Culicidae	Culicinae	*Mansonia*	*Mansonia sp.*	52
2021	Junho	14/06/2021	24	107	Teotônio	P03	Culicidae	Culicinae	*Coquillettidia*	*Coquillettidia sp.*	3
2021	Junho	14/06/2021	24	107	Teotônio	P03	Culicidae	Culicinae	*Mansonia*	*Mansonia sp.*	5
2021	Junho	14/06/2021	24	107	São Domingos	P02	Culicidae	Culicinae	*Mansonia*	*Mansonia sp.*	8
2021	Junho	14/06/2021	24	107	Cujubim Grande	P01	Culicidae	Culicinae	*Culex*	*Culex sp.*	7
2021	Junho	14/06/2021	24	107	Cujubim Grande	P01	Culicidae	Culicinae	*Mansonia*	*Mansonia sp.*	5
2021	Junho	21/06/2021	25	108	Rio Contra	P07	Culicidae	Anophelinae	*Anopheles*	*Anopheles darlingi*	2
2021	Junho	21/06/2021	25	108	Rio Contra	P07	Culicidae	Culicinae	*Culex*	*Culex sp.*	5
2021	Junho	21/06/2021	25	108	Rio Contra	P07	Culicidae	Culicinae	*Mansonia*	*Mansonia sp.*	8
2021	Junho	21/06/2021	25	108	Samauma	P08	Culicidae	Culicinae	*Mansonia*	*Mansonia sp.*	68
2021	Junho	21/06/2021	25	108	Samauma	P08	Culicidae	Culicinae	*Culex*	*Culex sp.*	13
2021	Junho	21/06/2021	25	108	Samauma	P08	Culicidae	Culicinae	*Aedeomyia*	*Adeomyia squamipennis*	2
2021	Junho	21/06/2021	25	108	Jaci Paraná	P06	Culicidae	Culicinae	*Aedes*	*Aedes sp.*	3
2021	Junho	21/06/2021	25	108	Jaci Paraná	P06	Culicidae	Culicinae	*Culex*	*Culex sp.*	2
2021	Junho	21/06/2021	25	108	Jaci Paraná	P06	Culicidae	Culicinae	*Mansonia*	*Mansonia sp.*	5
2021	Junho	21/06/2021	25	108	Santa Rita	P05	Culicidae	Culicinae	*Mansonia*	*Mansonia sp.*	15
2021	Junho	21/06/2021	25	108	Morrinhos	P04	Culicidae	Culicinae	*Mansonia*	*Mansonia sp.*	23
2021	Junho	21/06/2021	25	108	Morrinhos	P04	Culicidae	Culicinae	*Culex*	*Culex sp.*	1
2021	Junho	21/06/2021	25	108	Teotônio	P03	Culicidae	Culicinae	*Mansonia*	*Mansonia sp.*	2
2021	Junho	21/06/2021	25	108	Teotônio	P03	Culicidae	Culicinae	*Coquillettidia*	*Coquillettidia sp.*	1
2021	Junho	21/06/2021	25	108	São Domingos	P02	Culicidae	Culicinae	*Mansonia*	*Mansonia sp.*	7
2021	Junho	21/06/2021	25	108	Cujubim Grande	P01	Culicidae	Culicinae	*Mansonia*	*Mansonia sp.*	5
2021	Junho	21/06/2021	25	108	Cujubim Grande	P01	Culicidae	Culicinae	*Culex*	*Culex sp.*	2
2021	Junho	28/06/2021	26	109	Rio Contra	P07	Culicidae	Anophelinae	*Anopheles*	*Anopheles darlingi*	3
2021	Junho	28/06/2021	26	109	Rio Contra	P07	Culicidae	Culicinae	*Mansonia*	*Mansonia sp.*	27
2021	Junho	28/06/2021	26	109	Rio Contra	P07	Culicidae	Culicinae	*Culex*	*Culex sp.*	8
2021	Junho	28/06/2021	26	109	Samauma	P08	Culicidae	Culicinae	*Mansonia*	*Mansonia sp.*	7
2021	Junho	28/06/2021	26	109	Samauma	P08	Culicidae	Culicinae	*Culex*	*Culex sp.*	5
2021	Junho	28/06/2021	26	109	Jaci Paraná	P06	Culicidae	Culicinae	*Mansonia*	*Mansonia sp.*	10
2021	Junho	28/06/2021	26	109	Jaci Paraná	P06	Culicidae	Culicinae	*Aedes*	*Aedes sp.*	2
2021	Junho	28/06/2021	26	109	Santa Rita	P05	Culicidae	Culicinae	*Mansonia*	*Mansonia sp.*	22
2021	Junho	28/06/2021	26	109	Santa Rita	P05	Culicidae	Culicinae	*Culex*	*Culex sp.*	5
2021	Junho	28/06/2021	26	109	Morrinhos	P04	Culicidae	Culicinae	*Mansonia*	*Mansonia sp.*	35
2021	Junho	28/06/2021	26	109	Morrinhos	P04	Culicidae	Culicinae	*Culex*	*Culex sp.*	12
2021	Junho	28/06/2021	26	109	Teotônio	P03	Culicidae	Culicinae	*Mansonia*	*Mansonia sp.*	2
2021	Junho	28/06/2021	26	109	Teotônio	P03	Culicidae	Culicinae	*Coquillettidia*	*Coquillettidia sp.*	1
2021	Junho	28/06/2021	26	109	São Domingos	P02	Culicidae	Culicinae	*Mansonia*	*Mansonia sp.*	5
2021	Junho	28/06/2021	26	109	Cujubim Grande	P01	Culicidae	Culicinae	*Culex*	*Culex sp.*	6
2021	Junho	28/06/2021	26	109	Cujubim Grande	P01	Culicidae	Culicinae	*Mansonia*	*Mansonia sp.*	4
2021	Julho	06/07/2021	27	110	Teotônio	P03	Culicidae	Culicinae	*Mansonia*	*Mansonia sp.*	2
2021	Julho	06/07/2021	27	110	São Domingos	P02	Culicidae	Culicinae	*Mansonia*	*Mansonia amazonensis*	12
2021	Julho	06/07/2021	27	110	São Domingos	P02	Culicidae	Culicinae	*Aedeomyia*	*Adeomyia squamipennis*	1
2021	Julho	06/07/2021	27	110	São Domingos	P02	Culicidae	Culicinae	*Uranotaenia*	*Uranotaenia sp.*	1
2021	Julho	06/07/2021	27	110	São Domingos	P02	Culicidae	Anophelinae	*Anopheles*	*Anopheles sp.*	1
2021	Julho	06/07/2021	27	110	São Domingos	P02	Culicidae	Culicinae	*Coquillettidia*	*Coquillettidia sp.*	1
2021	Julho	06/07/2021	27	110	Cujubim Grande	P01	Culicidae	Culicinae	*Culex*	*Culex sp.*	42
2021	Julho	06/07/2021	27	110	Cujubim Grande	P01	Culicidae	Culicinae	*Mansonia*	*Mansonia sp.*	4
2021	Julho	06/07/2021	27	110	Cujubim Grande	P01	Culicidae	Culicinae	*Aedeomyia*	*Adeomyia squamipennis*	1
2021	Julho	06/07/2021	27	110	Cujubim Grande	P01	Culicidae	Culicinae	*Aedes*	*Aedes aegypti*	1
2021	Julho	06/07/2021	27	110	Cujubim Grande	P01	Culicidae	Culicinae	*Uranotaenia*	*Uranotaenia sp.*	1
2021	Julho	12/07/2021	28	111	Rio Contra	P07	Culicidae	Culicinae	*Mansonia*	*Mansonia sp.*	12
2021	Julho	12/07/2021	28	111	Rio Contra	P07	Culicidae	Anophelinae	*Anopheles*	*Anopheles sp.*	5
2021	Julho	12/07/2021	28	111	Samauma	P08	Culicidae	Culicinae	*Mansonia*	*Mansonia sp.*	33
2021	Julho	12/07/2021	28	111	Samauma	P08	Culicidae	Culicinae	*Culex*	*Culex sp.*	17
2021	Julho	12/07/2021	28	111	Samauma	P08	Culicidae	Culicinae	*Limatus*	*Limatus durhamii*	1
2021	Julho	12/07/2021	28	111	Jaci Paraná	P06	Culicidae	Culicinae	*Aedes*	*Aedes aegypti*	5
2021	Julho	12/07/2021	28	111	Jaci Paraná	P06	Culicidae	Culicinae	*Culex*	*Culex sp.*	8
2021	Julho	12/07/2021	28	111	Jaci Paraná	P06	Culicidae	Culicinae	*Mansonia*	*Mansonia sp.*	12
2021	Julho	12/07/2021	28	111	Santa Rita	P05	Culicidae	Culicinae	*Mansonia*	*Mansonia sp.*	5
2021	Julho	12/07/2021	28	111	Santa Rita	P05	Culicidae	Culicinae	*Culex*	*Culex sp.*	3
2021	Julho	12/07/2021	28	111	Morrinhos	P04	Culicidae	Culicinae	*Mansonia*	*Mansonia sp.*	27
2021	Julho	12/07/2021	28	111	Morrinhos	P04	Culicidae	Culicinae	*Culex*	*Culex sp.*	3
2021	Julho	13/07/2021	28	111	Teotônio	P03	Culicidae	Culicinae	*Coquillettidia*	*Coquillettidia sp.*	2
2021	Julho	13/07/2021	28	111	Teotônio	P03	Culicidae	Culicinae	*Mansonia*	*Mansonia sp.*	1
2021	Julho	13/07/2021	28	111	Teotônio	P03	Culicidae	Culicinae	*Culex*	*Culex sp.*	1
2021	Julho	13/07/2021	28	111	São Domingos	P02	Culicidae	Culicinae	*Mansonia*	*Mansonia sp.*	5
2021	Julho	13/07/2021	28	111	São Domingos	P02	Culicidae	Culicinae	*Culex*	*Culex sp.*	5
2021	Julho	13/07/2021	28	111	Cujubim Grande	P01	Culicidae	Culicinae	*Culex*	*Culex sp.*	10
2021	Julho	13/07/2021	28	111	Cujubim Grande	P01	Culicidae	Culicinae	*Mansonia*	*Mansonia sp.*	8
2021	Julho	19/07/2021	29	112	Rio Contra	P07	Culicidae	Anophelinae	*Anopheles*	*Anopheles darlingi*	3
2021	Julho	19/07/2021	29	112	Rio Contra	P07	Culicidae	Culicinae	*Mansonia*	*Mansonia sp.*	11
2021	Julho	19/07/2021	29	112	Rio Contra	P07	Culicidae	Culicinae	*Culex*	*Culex sp.*	2
2021	Julho	19/07/2021	29	112	Rio Contra	P07	Culicidae	Culicinae	*Aedeomyia*	*Adeomyia squamipennis*	3
2021	Julho	19/07/2021	29	112	Samauma	P08	Culicidae	Culicinae	*Aedeomyia*	*Adeomyia squamipennis*	5
2021	Julho	19/07/2021	29	112	Samauma	P08	Culicidae	Culicinae	*Mansonia*	*Mansonia sp.*	47
2021	Julho	19/07/2021	29	112	Samauma	P08	Culicidae	Culicinae	*Culex*	*Culex sp.*	21
2021	Julho	19/07/2021	29	112	Jaci Paraná	P06	Culicidae	Culicinae	*Aedes*	*Aedes sp.*	13
2021	Julho	19/07/2021	29	112	Jaci Paraná	P06	Culicidae	Culicinae	*Mansonia*	*Mansonia sp.*	11
2021	Julho	19/07/2021	29	112	Jaci Paraná	P06	Culicidae	Culicinae	*Culex*	*Culex sp.*	5
2021	Julho	19/07/2021	29	112	Santa Rita	P05	Culicidae	Culicinae	*Culex*	*Culex sp.*	22
2021	Julho	19/07/2021	29	112	Santa Rita	P05	Culicidae	Culicinae	*Mansonia*	*Mansonia sp.*	28
2021	Julho	19/07/2021	29	112	Morrinhos	P04	Culicidae	Culicinae	*Culex*	*Culex sp.*	19
2021	Julho	19/07/2021	29	112	Morrinhos	P04	Culicidae	Culicinae	*Mansonia*	*Mansonia sp.*	53
2021	Julho	20/07/2021	29	112	Teotônio	P03	Culicidae	Culicinae	*Coquillettidia*	*Coquillettidia sp.*	6
2021	Julho	20/07/2021	29	112	Teotônio	P03	Culicidae	Culicinae	*Mansonia*	*Mansonia sp.*	3
2021	Julho	20/07/2021	29	112	São Domingos	P02	Culicidae	Culicinae	*Coquillettidia*	*Coquillettidia sp.*	1
2021	Julho	20/07/2021	29	112	São Domingos	P02	Culicidae	Culicinae	*Mansonia*	*Mansonia sp.*	16
2021	Julho	20/07/2021	29	112	São Domingos	P02	Culicidae	Culicinae	*Culex*	*Culex sp.*	7
2021	Julho	20/07/2021	29	112	Cujubim Grande	P01	Culicidae	Culicinae	*Mansonia*	*Mansonia sp.*	8
2021	Julho	20/07/2021	29	112	Cujubim Grande	P01	Culicidae	Culicinae	*Culex*	*Culex sp.*	3
2021	Julho	26/07/2021	30	113	Rio Contra	P07	Culicidae	Anophelinae	*Anopheles*	*Anopheles sp.*	3
2021	Julho	26/07/2021	30	113	Rio Contra	P07	Culicidae	Culicinae	*Culex*	*Culex sp.*	1
2021	Julho	26/07/2021	30	113	Rio Contra	P07	Culicidae	Culicinae	*Mansonia*	*Mansonia sp.*	2
2021	Julho	26/07/2021	30	113	Samauma	P08	Culicidae	Culicinae	*Mansonia*	*Mansonia sp.*	189
2021	Julho	26/07/2021	30	113	Samauma	P08	Culicidae	Culicinae	*Culex*	*Culex sp.*	25
2021	Julho	26/07/2021	30	113	Samauma	P08	Culicidae	Culicinae	*Aedeomyia*	*Adeomyia squamipennis*	7
2021	Julho	26/07/2021	30	113	Jaci Paraná	P06	Culicidae	Anophelinae	*Anopheles*	*Anopheles sp.*	3
2021	Julho	26/07/2021	30	113	Jaci Paraná	P06	Culicidae	Culicinae	*Culex*	*Culex sp.*	15
2021	Julho	26/07/2021	30	113	Jaci Paraná	P06	Culicidae	Culicinae	*Mansonia*	*Mansonia sp.*	7
2021	Julho	26/07/2021	30	113	Santa Rita	P05	Culicidae	Culicinae	*Mansonia*	*Mansonia sp.*	35
2021	Julho	26/07/2021	30	113	Santa Rita	P05	Culicidae	Culicinae	*Culex*	*Culex sp.*	8
2021	Julho	26/07/2021	30	113	Morrinhos	P04	Culicidae	Culicinae	*Mansonia*	*Mansonia sp.*	176
2021	Julho	26/07/2021	30	113	Morrinhos	P04	Culicidae	Culicinae	*Culex*	*Culex sp.*	4
2021	Julho	26/07/2021	30	113	Morrinhos	P04	Culicidae	Culicinae	*Limatus*	*Limatus durhamii*	1
2021	Julho	27/07/2021	30	113	Teotônio	P03	Culicidae	Culicinae	*Culex*	*Culex sp.*	7
2021	Julho	27/07/2021	30	113	Teotônio	P03	Culicidae	Culicinae	*Coquillettidia*	*Coquillettidia sp.*	5
2021	Julho	27/07/2021	30	113	Teotônio	P03	Culicidae	Culicinae	*Mansonia*	*Mansonia sp.*	27
2021	Julho	27/07/2021	30	113	São Domingos	P02	Culicidae	Anophelinae	*Anopheles*	*Anopheles sp.*	4
2021	Julho	27/07/2021	30	113	São Domingos	P02	Culicidae	Culicinae	*Culex*	*Culex sp.*	18

2021	Julho	27/07/2021	30	113	São Domingos	P02	Culicidae	Culicinae	*Coquillettidia*	*Coquillettidia sp.*	8
2021	Julho	27/07/2021	30	113	São Domingos	P02	Culicidae	Culicinae	*Limatus*	*Limatus durhamii*	2
2021	Julho	27/07/2021	30	113	São Domingos	P02	Culicidae	Culicinae	*Mansonia*	*Mansonia sp.*	630
2021	Julho	27/07/2021	30	113	Cujubim Grande	P01	Culicidae	Culicinae	*Culex*	*Culex sp.*	3
2021	Julho	27/07/2021	30	113	Cujubim Grande	P01	Culicidae	Culicinae	*Mansonia*	*Mansonia sp.*	7
2021	Agosto	02/08/2021	31	114	Santa Rita	P05	Culicidae	Culicinae	*Mansonia*	*Mansonia sp.*	6
2021	Agosto	02/08/2021	31	114	Santa Rita	P05	Culicidae	Culicinae	*Culex*	*Culex sp.*	25
2021	Agosto	02/08/2021	31	114	Morrinhos	P04	Culicidae	Culicinae	*Culex*	*Culex sp.*	2
2021	Agosto	02/08/2021	31	114	Morrinhos	P04	Culicidae	Culicinae	*Mansonia*	*Mansonia sp.*	72
2021	Agosto	02/08/2021	31	114	Teotônio	P03	Culicidae	Culicinae	*Aedeomyia*	*Adeomyia squamipennis*	1
2021	Agosto	02/08/2021	31	114	Teotônio	P03	Culicidae	Anophelinae	*Anopheles*	*Anopheles nuneztovari s.l.*	1
2021	Agosto	02/08/2021	31	114	Teotônio	P03	Culicidae	Culicinae	*Mansonia*	*Mansonia sp.*	28
2021	Agosto	02/08/2021	31	114	Teotônio	P03	Culicidae	Culicinae	*Culex*	*Culex sp.*	12
2021	Agosto	02/08/2021	31	114	Teotônio	P03	Culicidae	Culicinae	*Coquillettidia*	*Coquillettidia sp.*	53
2021	Agosto	02/08/2021	31	114	Teotônio	P03	Culicidae	Anophelinae	*Anopheles*	*Anopheles deaneorum*	2
2021	Agosto	02/08/2021	31	114	São Domingos	P02	Culicidae	Culicinae	*Mansonia*	*Mansonia sp.*	1877
2021	Agosto	02/08/2021	31	114	São Domingos	P02	Culicidae	Culicinae	*Culex*	*Culex sp.*	7
2021	Agosto	02/08/2021	31	114	São Domingos	P02	Culicidae	Culicinae	*Coquillettidia*	*Coquillettidia sp.*	33
2021	Agosto	02/08/2021	31	114	São Domingos	P02	Culicidae	Anophelinae	*Anopheles*	*Anopheles sp.*	12
2021	Agosto	02/08/2021	31	114	Cujubim Grande	P01	Culicidae	Culicinae	*Mansonia*	*Mansonia sp.*	2
2021	Agosto	02/08/2021	31	114	Cujubim Grande	P01	Culicidae	Culicinae	*Culex*	*Culex sp.*	4
2021	Agosto	03/08/2021	31	114	Rio Contra	P07	Culicidae	Anophelinae	*Anopheles*	*Anopheles sp.*	37
2021	Agosto	03/08/2021	31	114	Rio Contra	P07	Culicidae	Anophelinae	*Anopheles*	*Anopheles darlingi*	8
2021	Agosto	03/08/2021	31	114	Rio Contra	P07	Culicidae	Culicinae	*Mansonia*	*Mansonia sp.*	11
2021	Agosto	03/08/2021	31	114	Rio Contra	P07	Culicidae	Culicinae	*Limatus*	*Limatus durhamii*	1
2021	Agosto	03/08/2021	31	114	Rio Contra	P07	Culicidae	Culicinae	*Culex*	*Culex sp.*	3
2021	Agosto	03/08/2021	31	114	Samauma	P08	Culicidae	Culicinae	*Mansonia*	*Mansonia sp.*	126
2021	Agosto	03/08/2021	31	114	Samauma	P08	Culicidae	Culicinae	*Culex*	*Culex sp.*	5
2021	Agosto	03/08/2021	31	114	Samauma	P08	Culicidae	Culicinae	*Aedeomyia*	*Adeomyia squamipennis*	2
2021	Agosto	03/08/2021	31	114	Jaci Paraná	P06	Culicidae	Culicinae	*Aedes*	*Aedes sp.*	1
2021	Agosto	03/08/2021	31	114	Jaci Paraná	P06	Culicidae	Culicinae	*Mansonia*	*Mansonia sp.*	8
2021	Agosto	03/08/2021	31	114	Jaci Paraná	P06	Culicidae	Culicinae	*Culex*	*Culex sp.*	37
2021	Agosto	09/08/2021	32	115	Rio Contra	P07	Culicidae	Anophelinae	*Anopheles*	*Anopheles sp.*	1
2021	Agosto	09/08/2021	32	115	Rio Contra	P07	Culicidae	Culicinae	*Mansonia*	*Mansonia sp.*	3
2021	Agosto	09/08/2021	32	115	Rio Contra	P07	Culicidae	Culicinae	*Culex*	*Culex sp.*	1
2021	Agosto	09/08/2021	32	115	Samauma	P08	Culicidae	Culicinae	*Culex*	*Culex sp.*	2
2021	Agosto	09/08/2021	32	115	Samauma	P08	Culicidae	Culicinae	*Mansonia*	*Mansonia sp.*	26
2021	Agosto	09/08/2021	32	115	Samauma	P08	Culicidae	Culicinae	*Aedeomyia*	*Adeomyia squamipennis*	2
2021	Agosto	09/08/2021	32	115	Jaci Paraná	P06	Culicidae	Culicinae	*Culex*	*Culex sp.*	28
2021	Agosto	09/08/2021	32	115	Jaci Paraná	P06	Culicidae	Culicinae	*Mansonia*	*Mansonia sp.*	2
2021	Agosto	09/08/2021	32	115	Jaci Paraná	P06	Culicidae	Culicinae	*Aedes*	*Aedes aegypti*	3
2021	Agosto	09/08/2021	32	115	Santa Rita	P05	Culicidae	Culicinae	*Mansonia*	*Mansonia sp.*	31
2021	Agosto	09/08/2021	32	115	Santa Rita	P05	Culicidae	Culicinae	*Culex*	*Culex sp.*	2
2021	Agosto	09/08/2021	32	115	Morrinhos	P04	Culicidae	Culicinae	*Mansonia*	*Mansonia sp.*	53
2021	Agosto	09/08/2021	32	115	Morrinhos	P04	Culicidae	Culicinae	*Culex*	*Culex sp.*	12
2021	Agosto	09/08/2021	32	115	Teotônio	P03	Culicidae	Culicinae	*Coquillettidia*	*Coquillettidia sp.*	7
2021	Agosto	09/08/2021	32	115	Teotônio	P03	Culicidae	Culicinae	*Mansonia*	*Mansonia sp.*	8
2021	Agosto	09/08/2021	32	115	Teotônio	P03	Culicidae	Culicinae	*Culex*	*Culex sp.*	5
2021	Agosto	09/08/2021	32	115	São Domingos	P02	Culicidae	Culicinae	*Mansonia*	*Mansonia sp.*	1537
2021	Agosto	09/08/2021	32	115	São Domingos	P02	Culicidae	Culicinae	*Culex*	*Culex sp.*	8
2021	Agosto	09/08/2021	32	115	São Domingos	P02	Culicidae	Culicinae	*Coquillettidia*	*Coquillettidia sp.*	5
2021	Agosto	09/08/2021	32	115	Cujubim Grande	P01	Culicidae	Culicinae	*Culex*	*Culex sp.*	22
2021	Agosto	09/08/2021	32	115	Cujubim Grande	P01	Culicidae	Culicinae	*Mansonia*	*Mansonia sp.*	3
2021	Agosto	16/08/2021	33	116	Rio Contra	P07	Culicidae	Culicinae	*Mansonia*	*Mansonia sp.*	3
2021	Agosto	16/08/2021	33	116	Rio Contra	P07	Culicidae	Culicinae	*Culex*	*Culex sp.*	2
2021	Agosto	16/08/2021	33	116	Samauma	P08	Culicidae	Culicinae	*Mansonia*	*Mansonia sp.*	72
2021	Agosto	16/08/2021	33	116	Samauma	P08	Culicidae	Culicinae	*Culex*	*Culex sp.*	10
2021	Agosto	16/08/2021	33	116	Samauma	P08	Culicidae	Culicinae	*Aedeomyia*	*Adeomyia squamipennis*	5
2021	Agosto	16/08/2021	33	116	Jaci Paraná	P06	Culicidae	Culicinae	*Culex*	*Culex sp.*	32
2021	Agosto	16/08/2021	33	116	Jaci Paraná	P06	Culicidae	Culicinae	*Mansonia*	*Mansonia sp.*	3
2021	Agosto	16/08/2021	33	116	Santa Rita	P05	Culicidae	Culicinae	*Mansonia*	*Mansonia sp.*	1
2021	Agosto	16/08/2021	33	116	Santa Rita	P05	Culicidae	Culicinae	*Culex*	*Culex sp.*	2
2021	Agosto	16/08/2021	33	116	Morrinhos	P04	Culicidae	Culicinae	*Mansonia*	*Mansonia sp.*	28
2021	Agosto	16/08/2021	33	116	Morrinhos	P04	Culicidae	Culicinae	*Culex*	*Culex sp.*	3
2021	Agosto	16/08/2021	33	116	Teotônio	P03	Culicidae	Culicinae	*Mansonia*	*Mansonia sp.*	3
2021	Agosto	16/08/2021	33	116	Teotônio	P03	Culicidae	Culicinae	*Culex*	*Culex sp.*	1
2021	Agosto	16/08/2021	33	116	São Domingos	P02	Culicidae	Culicinae	*Mansonia*	*Mansonia sp.*	1483
2021	Agosto	16/08/2021	33	116	São Domingos	P02	Culicidae	Culicinae	*Culex*	*Culex sp.*	27
2021	Agosto	16/08/2021	33	116	São Domingos	P02	Culicidae	Culicinae	*Coquillettidia*	*Coquillettidia sp.*	13
2021	Agosto	16/08/2021	33	116	São Domingos	P02	Culicidae	Culicinae	*Culex*	*Culex sp.*	2
2021	Agosto	16/08/2021	33	116	Cujubim Grande	P01	Culicidae	Culicinae	*Culex*	*Culex sp.*	25
2021	Agosto	16/08/2021	33	116	Cujubim Grande	P01	Culicidae	Culicinae	*Mansonia*	*Mansonia sp.*	1
2021	Agosto	23/08/2021	34	117	Rio Contra	P07	Culicidae	Anophelinae	*Anopheles*	*Anopheles sp.*	2
2021	Agosto	23/08/2021	34	117	Rio Contra	P07	Culicidae	Culicinae	*Mansonia*	*Mansonia sp.*	4
2021	Agosto	23/08/2021	34	117	Samauma	P08	Culicidae	Culicinae	*Aedeomyia*	*Adeomyia squamipennis*	2
2021	Agosto	23/08/2021	34	117	Samauma	P08	Culicidae	Culicinae	*Culex*	*Culex sp.*	3
2021	Agosto	23/08/2021	34	117	Samauma	P08	Culicidae	Culicinae	*Mansonia*	*Mansonia sp.*	23
2021	Agosto	23/08/2021	34	117	Jaci Paraná	P06	Culicidae	Culicinae	*Culex*	*Culex sp.*	15
2021	Agosto	23/08/2021	34	117	Jaci Paraná	P06	Culicidae	Culicinae	*Mansonia*	*Mansonia sp.*	8
2021	Agosto	23/08/2021	34	117	Santa Rita	P05	Culicidae	Culicinae	*Culex*	*Culex sp.*	4
2021	Agosto	23/08/2021	34	117	Santa Rita	P05	Culicidae	Culicinae	*Mansonia*	*Mansonia sp.*	8
2021	Agosto	23/08/2021	34	117	Morrinhos	P04	Culicidae	Culicinae	*Mansonia*	*Mansonia sp.*	18
2021	Agosto	23/08/2021	34	117	Morrinhos	P04	Culicidae	Culicinae	*Culex*	*Culex sp.*	3
2021	Agosto	23/08/2021	34	117	Teotônio	P03	Culicidae	Culicinae	*Coquillettidia*	*Coquillettidia sp.*	1
2021	Agosto	23/08/2021	34	117	São Domingos	P02	Culicidae	Culicinae	*Mansonia*	*Mansonia sp.*	452
2021	Agosto	23/08/2021	34	117	São Domingos	P02	Culicidae	Culicinae	*Coquillettidia*	*Coquillettidia sp.*	2
2021	Agosto	23/08/2021	34	117	São Domingos	P02	Culicidae	Culicinae	*Culex*	*Culex sp.*	5
2021	Agosto	23/08/2021	34	117	Cujubim Grande	P01	Culicidae	Culicinae	*Culex*	*Culex sp.*	12
2021	Agosto	23/08/2021	34	117	Cujubim Grande	P01	Culicidae	Culicinae	*Mansonia*	*Mansonia sp.*	3
2021	Agosto	30/08/2021	35	118	Rio Contra	P07	Culicidae	Anophelinae	*Anopheles*	*Anopheles sp.*	2
2021	Agosto	30/08/2021	35	118	Rio Contra	P07	Culicidae	Culicinae	*Mansonia*	*Mansonia sp.*	5
2021	Agosto	30/08/2021	35	118	Samauma	P08	Culicidae	Culicinae	*Culex*	*Culex sp.*	6
2021	Agosto	30/08/2021	35	118	Samauma	P08	Culicidae	Culicinae	*Aedeomyia*	*Adeomyia squamipennis*	2
2021	Agosto	30/08/2021	35	118	Samauma	P08	Culicidae	Culicinae	*Mansonia*	*Mansonia sp.*	21
2021	Agosto	30/08/2021	35	118	Jaci Paraná	P06	Culicidae	Culicinae	*Culex*	*Culex sp.*	27
2021	Agosto	30/08/2021	35	118	Jaci Paraná	P06	Culicidae	Culicinae	*Mansonia*	*Mansonia sp.*	3
2021	Agosto	30/08/2021	35	118	Jaci Paraná	P06	Culicidae	Culicinae	*Aedes*	*Aedes aegypti*	2
2021	Agosto	30/08/2021	35	118	Santa Rita	P05	Culicidae	Culicinae	*Culex*	*Culex sp.*	20
2021	Agosto	30/08/2021	35	118	Santa Rita	P05	Culicidae	Culicinae	*Mansonia*	*Mansonia sp.*	1
2021	Agosto	30/08/2021	35	118	Morrinhos	P04	Culicidae	Culicinae	*Mansonia*	*Mansonia sp.*	48
2021	Agosto	30/08/2021	35	118	Morrinhos	P04	Culicidae	Culicinae	*Culex*	*Culex sp.*	4
2021	Agosto	30/08/2021	35	118	São Domingos	P02	Culicidae	Culicinae	*Coquillettidia*	*Coquillettidia sp.*	6
2021	Agosto	30/08/2021	35	118	São Domingos	P02	Culicidae	Culicinae	*Mansonia*	*Mansonia sp.*	283
2021	Agosto	30/08/2021	35	118	São Domingos	P02	Culicidae	Culicinae	*Culex*	*Culex sp.*	18
2021	Agosto	30/08/2021	35	118	São Domingos	P02	Culicidae	Anophelinae	*Anopheles*	*Anopheles sp.*	2
2021	Setembro	06/09/2021	36	119	Rio Contra	P07	Culicidae	Culicinae	*Mansonia*	*Mansonia sp.*	6
2021	Setembro	06/09/2021	36	119	Samauma	P08	Culicidae	Culicinae	*Mansonia*	*Mansonia sp.*	18
2021	Setembro	06/09/2021	36	119	Samauma	P08	Culicidae	Culicinae	*Culex*	*Culex sp.*	3
2021	Setembro	06/09/2021	36	119	Samauma	P08	Culicidae	Culicinae	*Aedeomyia*	*Adeomyia squamipennis*	2
2021	Setembro	06/09/2021	36	119	Jaci Paraná	P06	Culicidae	Culicinae	*Aedes*	*Aedes sp.*	3
2021	Setembro	06/09/2021	36	119	Jaci Paraná	P06	Culicidae	Culicinae	*Aedes*	*Aedes aegypti*	4
2021	Setembro	06/09/2021	36	119	Jaci Paraná	P06	Culicidae	Culicinae	*Mansonia*	*Mansonia sp.*	8
2021	Setembro	06/09/2021	36	119	Jaci Paraná	P06	Culicidae	Culicinae	*Culex*	*Culex sp.*	5
2021	Setembro	06/09/2021	36	119	Santa Rita	P05	Culicidae	Culicinae	*Mansonia*	*Mansonia sp.*	13
2021	Setembro	06/09/2021	36	119	Morrinhos	P04	Culicidae	Culicinae	*Mansonia*	*Mansonia sp.*	23
2021	Setembro	06/09/2021	36	119	Morrinhos	P04	Culicidae	Culicinae	*Culex*	*Culex sp.*	12
2021	Setembro	06/09/2021	36	119	Teotônio	P03	Culicidae	Culicinae	*Coquillettidia*	*Coquillettidia sp.*	2
2021	Setembro	06/09/2021	36	119	Teotônio	P03	Culicidae	Culicinae	*Mansonia*	*Mansonia sp.*	1

2021	Setembro	06/09/2021	36	119	São Domingos	P02	Culicidae	Culicinae	*Aedes*	*Aedes sp.*	5
2021	Setembro	06/09/2021	36	119	São Domingos	P02	Culicidae	Culicinae	*Coquillettidia*	*Coquillettidia sp.*	28
2021	Setembro	06/09/2021	36	119	São Domingos	P02	Culicidae	Culicinae	*Mansonia*	*Mansonia sp.*	1732
2021	Setembro	06/09/2021	36	119	São Domingos	P02	Culicidae	Culicinae	*Aedeomyia*	*Adeomyia squamipennis*	5
2021	Setembro	06/09/2021	36	119	Cujubim Grande	P01	Culicidae	Culicinae	*Mansonia*	*Mansonia sp.*	5
2021	Setembro	06/09/2021	36	119	Cujubim Grande	P01	Culicidae	Culicinae	*Culex*	*Culex sp.*	12
2021	Setembro	13/09/2021	37	120	Rio Contra	P07	Culicidae	Culicinae	*Mansonia*	*Mansonia sp.*	10
2021	Setembro	13/09/2021	37	120	Rio Contra	P07	Culicidae	Culicinae	*Culex*	*Culex sp.*	2
2021	Setembro	13/09/2021	37	120	Rio Contra	P07	Culicidae	Anophelinae	*Anopheles*	*Anopheles sp.*	1
2021	Setembro	13/09/2021	37	120	Samauma	P08	Culicidae	Culicinae	*Mansonia*	*Mansonia sp.*	53
2021	Setembro	13/09/2021	37	120	Samauma	P08	Culicidae	Culicinae	*Culex*	*Culex sp.*	17
2021	Setembro	13/09/2021	37	120	Samauma	P08	Culicidae	Culicinae	*Aedeomyia*	*Adeomyia squamipennis*	2
2021	Setembro	13/09/2021	37	120	Jaci Paraná	P06	Culicidae	Culicinae	*Mansonia*	*Mansonia sp.*	5
2021	Setembro	13/09/2021	37	120	Jaci Paraná	P06	Culicidae	Culicinae	*Culex*	*Culex sp.*	3
2021	Setembro	13/09/2021	37	120	Jaci Paraná	P06	Culicidae	Culicinae	*Aedes*	*Aedes sp.*	2
2021	Setembro	13/09/2021	37	120	Santa Rita	P05	Culicidae	Culicinae	*Mansonia*	*Mansonia sp.*	5
2021	Setembro	13/09/2021	37	120	Morrinhos	P04	Culicidae	Culicinae	*Mansonia*	*Mansonia sp.*	13
2021	Setembro	13/09/2021	37	120	Morrinhos	P04	Culicidae	Culicinae	*Culex*	*Culex sp.*	8
2021	Setembro	13/09/2021	37	120	Teotônio	P03	Culicidae	Culicinae	*Coquillettidia*	*Coquillettidia sp.*	7
2021	Setembro	13/09/2021	37	120	Teotônio	P03	Culicidae	Culicinae	*Culex*	*Culex sp.*	3
2021	Setembro	13/09/2021	37	120	São Domingos	P02	Culicidae	Culicinae	*Mansonia*	*Mansonia sp.*	902
2021	Setembro	13/09/2021	37	120	São Domingos	P02	Culicidae	Culicinae	*Coquillettidia*	*Coquillettidia sp.*	28
2021	Setembro	13/09/2021	37	120	São Domingos	P02	Culicidae	Culicinae	*Aedes*	*Aedes sp.*	7
2021	Setembro	13/09/2021	37	120	São Domingos	P02	Culicidae	Culicinae	*Psorophora*	*Psorophora sp.*	1
2021	Setembro	13/09/2021	37	120	Cujubim Grande	P01	Culicidae	Culicinae	*Culex*	*Culex sp.*	3
2021	Setembro	13/09/2021	37	120	Cujubim Grande	P01	Culicidae	Culicinae	*Mansonia*	*Mansonia sp.*	1
2021	Setembro	20/09/2021	38	121	Rio Contra	P07	Culicidae	Anophelinae	*Anopheles*	*Anopheles darlingi*	3
2021	Setembro	20/09/2021	38	121	Rio Contra	P07	Culicidae	Culicinae	*Culex*	*Culex sp.*	4
2021	Setembro	20/09/2021	38	121	Rio Contra	P07	Culicidae	Culicinae	*Mansonia*	*Mansonia sp.*	2
2021	Setembro	20/09/2021	38	121	Samauma	P08	Culicidae	Culicinae	*Mansonia*	*Mansonia sp.*	21
2021	Setembro	20/09/2021	38	121	Samauma	P08	Culicidae	Culicinae	*Culex*	*Culex sp.*	12
2021	Setembro	20/09/2021	38	121	Jaci Paraná	P06	Culicidae	Culicinae	*Aedes*	*Aedes sp.*	5
2021	Setembro	20/09/2021	38	121	Jaci Paraná	P06	Culicidae	Culicinae	*Mansonia*	*Mansonia sp.*	2
2021	Setembro	20/09/2021	38	121	Jaci Paraná	P06	Culicidae	Culicinae	*Culex*	*Culex sp.*	8
2021	Setembro	20/09/2021	38	121	Santa Rita	P05	Culicidae	Culicinae	*Mansonia*	*Mansonia sp.*	2
2021	Setembro	20/09/2021	38	121	Morrinhos	P04	Culicidae	Culicinae	*Mansonia*	*Mansonia sp.*	13
2021	Setembro	20/09/2021	38	121	São Domingos	P02	Culicidae	Culicinae	*Mansonia*	*Mansonia sp.*	3807
2021	Setembro	20/09/2021	38	121	São Domingos	P02	Culicidae	Culicinae	*Coquillettidia*	*Coquillettidia sp.*	28
2021	Setembro	20/09/2021	38	121	São Domingos	P02	Culicidae	Culicinae	*Psorophora*	*Psorophora sp.*	3
2021	Setembro	20/09/2021	38	121	São Domingos	P02	Culicidae	Culicinae	*Aedes*	*Aedes sp.*	7
2021	Setembro	20/09/2021	38	121	São Domingos	P02	Culicidae	Culicinae	*Culex*	*Culex sp.*	53
2021	Setembro	27/09/2021	39	122	Rio Contra	P07	Culicidae	Anophelinae	*Anopheles*	*Anopheles darlingi*	3
2021	Setembro	27/09/2021	39	122	Rio Contra	P07	Culicidae	Culicinae	*Mansonia*	*Mansonia sp.*	2
2021	Setembro	27/09/2021	39	122	Rio Contra	P07	Culicidae	Culicinae	*Culex*	*Culex sp.*	1
2021	Setembro	27/09/2021	39	122	Samauma	P08	Culicidae	Culicinae	*Mansonia*	*Mansonia sp.*	38
2021	Setembro	27/09/2021	39	122	Samauma	P08	Culicidae	Culicinae	*Culex*	*Culex sp.*	10
2021	Setembro	27/09/2021	39	122	Samauma	P08	Culicidae	Culicinae	*Coquillettidia*	*Coquillettidia sp.*	5
2021	Setembro	27/09/2021	39	122	Samauma	P08	Culicidae	Culicinae	*Aedeomyia*	*Adeomyia squamipennis*	1
2021	Setembro	27/09/2021	39	122	Jaci Paraná	P06	Culicidae	Culicinae	*Culex*	*Culex sp.*	15
2021	Setembro	27/09/2021	39	122	Jaci Paraná	P06	Culicidae	Culicinae	*Mansonia*	*Mansonia sp.*	3
2021	Setembro	27/09/2021	39	122	Jaci Paraná	P06	Culicidae	Culicinae	*Aedes*	*Aedes sp.*	2
2021	Setembro	27/09/2021	39	122	Santa Rita	P05	Culicidae	Culicinae	*Mansonia*	*Mansonia sp.*	5
2021	Setembro	27/09/2021	39	122	Santa Rita	P05	Culicidae	Culicinae	*Culex*	*Culex sp.*	2
2021	Setembro	27/09/2021	39	122	Morrinhos	P04	Culicidae	Culicinae	*Mansonia*	*Mansonia sp.*	23
2021	Setembro	27/09/2021	39	122	Morrinhos	P04	Culicidae	Culicinae	*Culex*	*Culex sp.*	4
2021	Setembro	27/09/2021	39	122	Teotônio	P03	Culicidae	Anophelinae	*Anopheles*	*Anopheles sp.*	2
2021	Setembro	27/09/2021	39	122	Teotônio	P03	Culicidae	Culicinae	*Coquillettidia*	*Coquillettidia sp.*	7
2021	Setembro	27/09/2021	39	122	Teotônio	P03	Culicidae	Culicinae	*Mansonia*	*Mansonia sp.*	8
2021	Setembro	27/09/2021	39	122	São Domingos	P02	Culicidae	Culicinae	*Mansonia*	*Mansonia sp.*	1237
2021	Setembro	27/09/2021	39	122	São Domingos	P02	Culicidae	Culicinae	*Culex*	*Culex sp.*	53
2021	Setembro	27/09/2021	39	122	São Domingos	P02	Culicidae	Culicinae	*Aedeomyia*	*Adeomyia squamipennis*	4
2021	Setembro	27/09/2021	39	122	São Domingos	P02	Culicidae	Culicinae	*Coquillettidia*	*Coquillettidia sp.*	12
2021	Setembro	27/09/2021	39	122	Cujubim Grande	P01	Culicidae	Culicinae	*Culex*	*Culex sp.*	2
2021	Setembro	27/09/2021	39	122	Cujubim Grande	P01	Culicidae	Culicinae	*Mansonia*	*Mansonia sp.*	1
2021	Outubro	04/10/2021	40	123	Rio Contra	P07	Culicidae	Anophelinae	*Anopheles*	*Anopheles sp.*	3
2021	Outubro	04/10/2021	40	123	Rio Contra	P07	Culicidae	Culicinae	*Mansonia*	*Mansonia sp.*	8
2021	Outubro	04/10/2021	40	123	Rio Contra	P07	Culicidae	Culicinae	*Culex*	*Culex sp.*	5
2021	Outubro	04/10/2021	40	123	Rio Contra	P07	Culicidae	Anophelinae	*Anopheles*	*Anopheles darlingi*	2
2021	Outubro	04/10/2021	40	123	Samauma	P08	Culicidae	Culicinae	*Culex*	*Culex sp.*	10
2021	Outubro	04/10/2021	40	123	Samauma	P08	Culicidae	Culicinae	*Mansonia*	*Mansonia sp.*	23
2021	Outubro	04/10/2021	40	123	Jaci Paraná	P06	Culicidae	Culicinae	*Mansonia*	*Mansonia sp.*	4
2021	Outubro	04/10/2021	40	123	Jaci Paraná	P06	Culicidae	Culicinae	*Aedes*	*Aedes sp.*	1
2021	Outubro	04/10/2021	40	123	Jaci Paraná	P06	Culicidae	Culicinae	*Culex*	*Culex sp.*	18
2021	Outubro	04/10/2021	40	123	Santa Rita	P05	Culicidae	Culicinae	*Mansonia*	*Mansonia sp.*	6
2021	Outubro	04/10/2021	40	123	Morrinhos	P04	Culicidae	Culicinae	*Mansonia*	*Mansonia sp.*	64
2021	Outubro	04/10/2021	40	123	Teotônio	P03	Culicidae	Culicinae	*Coquillettidia*	*Coquillettidia sp.*	4
2021	Outubro	04/10/2021	40	123	Teotônio	P03	Culicidae	Culicinae	*Mansonia*	*Mansonia sp.*	9
2021	Outubro	04/10/2021	40	123	Teotônio	P03	Culicidae	Culicinae	*Culex*	*Culex sp.*	5
2021	Outubro	04/10/2021	40	123	São Domingos	P02	Culicidae	Anophelinae	*Anopheles*	*Anopheles sp.*	5
2021	Outubro	04/10/2021	40	123	São Domingos	P02	Culicidae	Culicinae	*Coquillettidia*	*Coquillettidia sp.*	12
2021	Outubro	04/10/2021	40	123	São Domingos	P02	Culicidae	Culicinae	*Culex*	*Culex sp.*	1435
2021	Outubro	04/10/2021	40	123	São Domingos	P02	Culicidae	Culicinae	*Mansonia*	*Mansonia sp.*	71
2021	Outubro	04/10/2021	40	123	Cujubim Grande	P01	Culicidae	Culicinae	*Mansonia*	*Mansonia sp.*	3
2021	Outubro	04/10/2021	40	123	Cujubim Grande	P01	Culicidae	Culicinae	*Culex*	*Culex sp.*	2
2021	Outubro	04/10/2021	40	124	Rio Contra	P07	Culicidae	Anophelinae	*Anopheles*	*Anopheles sp.*	3
2021	Outubro	04/10/2021	40	124	Rio Contra	P07	Culicidae	Culicinae	*Mansonia*	*Mansonia sp.*	8
2021	Outubro	04/10/2021	40	124	Rio Contra	P07	Culicidae	Culicinae	*Culex*	*Culex sp.*	5
2021	Outubro	04/10/2021	40	124	Rio Contra	P07	Culicidae	Anophelinae	*Anopheles*	*Anopheles darlingi*	2
2021	Outubro	04/10/2021	40	124	Samauma	P08	Culicidae	Culicinae	*Culex*	*Culex sp.*	10
2021	Outubro	04/10/2021	40	124	Samauma	P08	Culicidae	Culicinae	*Mansonia*	*Mansonia sp.*	23
2021	Outubro	04/10/2021	40	124	Jaci Paraná	P06	Culicidae	Culicinae	*Mansonia*	*Mansonia sp.*	4
2021	Outubro	04/10/2021	40	124	Jaci Paraná	P06	Culicidae	Culicinae	*Aedes*	*Aedes sp.*	1
2021	Outubro	04/10/2021	40	124	Jaci Paraná	P06	Culicidae	Culicinae	*Culex*	*Culex sp.*	18
2021	Outubro	04/10/2021	40	124	Santa Rita	P05	Culicidae	Culicinae	*Mansonia*	*Mansonia sp.*	6
2021	Outubro	04/10/2021	40	124	Morrinhos	P04	Culicidae	Culicinae	*Mansonia*	*Mansonia sp.*	64
2021	Outubro	04/10/2021	40	124	Teotônio	P03	Culicidae	Culicinae	*Coquillettidia*	*Coquillettidia sp.*	4
2021	Outubro	04/10/2021	40	124	Teotônio	P03	Culicidae	Culicinae	*Mansonia*	*Mansonia sp.*	9
2021	Outubro	04/10/2021	40	124	Teotônio	P03	Culicidae	Culicinae	*Culex*	*Culex sp.*	5
2021	Outubro	04/10/2021	40	124	São Domingos	P02	Culicidae	Anophelinae	*Anopheles*	*Anopheles sp.*	5
2021	Outubro	04/10/2021	40	124	São Domingos	P02	Culicidae	Culicinae	*Coquillettidia*	*Coquillettidia sp.*	12
2021	Outubro	04/10/2021	40	124	São Domingos	P02	Culicidae	Culicinae	*Culex*	*Culex sp.*	1435
2021	Outubro	04/10/2021	40	124	São Domingos	P02	Culicidae	Culicinae	*Mansonia*	*Mansonia sp.*	71
2021	Outubro	04/10/2021	40	124	Cujubim Grande	P01	Culicidae	Culicinae	*Mansonia*	*Mansonia sp.*	3
2021	Outubro	04/10/2021	40	124	Cujubim Grande	P01	Culicidae	Culicinae	*Culex*	*Culex sp.*	2
2021	Outubro	11/10/2021	41	125	Rio Contra	P07	Culicidae	Anophelinae	*Anopheles*	*Anopheles sp.*	2
2021	Outubro	11/10/2021	41	125	Rio Contra	P07	Culicidae	Anophelinae	*Anopheles*	*Anopheles darlingi*	4
2021	Outubro	11/10/2021	41	125	Rio Contra	P07	Culicidae	Culicinae	*Mansonia*	*Mansonia sp.*	3
2021	Outubro	11/10/2021	41	125	Rio Contra	P07	Culicidae	Culicinae	*Culex*	*Culex sp.*	7
2021	Outubro	11/10/2021	41	125	Samauma	P08	Culicidae	Culicinae	*Mansonia*	*Mansonia sp.*	28
2021	Outubro	11/10/2021	41	125	Samauma	P08	Culicidae	Culicinae	*Culex*	*Culex sp.*	12
2021	Outubro	11/10/2021	41	125	Jaci Paraná	P06	Culicidae	Culicinae	*Mansonia*	*Mansonia sp.*	3
2021	Outubro	11/10/2021	41	125	Jaci Paraná	P06	Culicidae	Culicinae	*Culex*	*Culex sp.*	2
2021	Outubro	11/10/2021	41	125	Jaci Paraná	P06	Culicidae	Culicinae	*Aedes*	*Aedes sp.*	2
2021	Outubro	11/10/2021	41	125	Santa Rita	P05	Culicidae	Culicinae	*Mansonia*	*Mansonia humeralis*	2
2021	Outubro	11/10/2021	41	125	Morrinhos	P04	Culicidae	Culicinae	*Mansonia*	*Mansonia sp.*	53
2021	Outubro	11/10/2021	41	125	Morrinhos	P04	Culicidae	Culicinae	*Culex*	*Culex sp.*	15
2021	Outubro	11/10/2021	41	125	São Domingos	P02	Culicidae	Anophelinae	*Anopheles*	*Anopheles sp.*	8
2021	Outubro	11/10/2021	41	125	São Domingos	P02	Culicidae	Culicinae	*Coquillettidia*	*Coquillettidia sp.*	5

2021	Outubro	11/10/2021	41	125	São Domingos	P02	Culicidae	Culicinae	*Culex*	*Culex sp.*	1975
2021	Outubro	11/10/2021	41	125	São Domingos	P02	Culicidae	Culicinae	*Mansonia*	*Mansonia sp.*	82
2021	Outubro	11/10/2021	41	125	Cujubim Grande	P01	Culicidae	Culicinae	*Culex*	*Culex sp.*	2
2021	Outubro	11/10/2021	41	125	Rio Contra	P07	Culicidae	Anophelinae	*Anopheles*	*Anopheles sp.*	2
2021	Outubro	11/10/2021	41	125	Rio Contra	P07	Culicidae	Anophelinae	*Anopheles*	*Anopheles darlingi*	4
2021	Outubro	11/10/2021	41	125	Rio Contra	P07	Culicidae	Culicinae	*Mansonia*	*Mansonia sp.*	3
2021	Outubro	11/10/2021	41	125	Rio Contra	P07	Culicidae	Culicinae	*Culex*	*Culex sp.*	7
2021	Outubro	11/10/2021	41	125	Samauma	P08	Culicidae	Culicinae	*Mansonia*	*Mansonia sp.*	28
2021	Outubro	11/10/2021	41	125	Samauma	P08	Culicidae	Culicinae	*Culex*	*Culex sp.*	12
2021	Outubro	11/10/2021	41	125	Jaci Paraná	P06	Culicidae	Culicinae	*Mansonia*	*Mansonia sp.*	3
2021	Outubro	11/10/2021	41	125	Jaci Paraná	P06	Culicidae	Culicinae	*Culex*	*Culex sp.*	2
2021	Outubro	11/10/2021	41	125	Jaci Paraná	P06	Culicidae	Culicinae	*Aedes*	*Aedes sp.*	2
2021	Outubro	11/10/2021	41	125	Santa Rita	P05	Culicidae	Culicinae	*Mansonia*	*Mansonia sp.*	2
2021	Outubro	11/10/2021	41	125	Morrinhos	P04	Culicidae	Culicinae	*Mansonia*	*Mansonia sp.*	53
2021	Outubro	11/10/2021	41	125	Morrinhos	P04	Culicidae	Culicinae	*Culex*	*Culex sp.*	15
2021	Outubro	11/10/2021	41	125	São Domingos	P02	Culicidae	Anophelinae	*Anopheles*	*Anopheles sp.*	8
2021	Outubro	11/10/2021	41	125	São Domingos	P02	Culicidae	Culicinae	*Coquillettidia*	*Coquillettidia sp.*	5
2021	Outubro	11/10/2021	41	125	São Domingos	P02	Culicidae	Culicinae	*Culex*	*Culex sp.*	1975
2021	Outubro	11/10/2021	41	125	São Domingos	P02	Culicidae	Culicinae	*Mansonia*	*Mansonia sp.*	82
2021	Outubro	11/10/2021	41	125	Cujubim Grande	P01	Culicidae	Culicinae	*Culex*	*Culex sp.*	2
2021	Outubro	18/10/2021	42	126	Rio Contra	P07	Culicidae	Culicinae	*Mansonia*	*Mansonia sp.*	2
2021	Outubro	18/10/2021	42	126	Samauma	P08	Culicidae	Culicinae	*Mansonia*	*Mansonia sp.*	15
2021	Outubro	18/10/2021	42	126	Samauma	P08	Culicidae	Culicinae	*Culex*	*Culex sp.*	8
2021	Outubro	18/10/2021	42	126	Jaci Paraná	P06	Culicidae	Culicinae	*Mansonia*	*Mansonia sp.*	8
2021	Outubro	18/10/2021	42	126	Jaci Paraná	P06	Culicidae	Culicinae	*Culex*	*Culex sp.*	15
2021	Outubro	18/10/2021	42	126	Jaci Paraná	P06	Culicidae	Culicinae	*Aedes*	*Aedes sp.*	2
2021	Outubro	18/10/2021	42	126	Santa Rita	P05	Culicidae	Culicinae	*Culex*	*Culex sp.*	3
2021	Outubro	18/10/2021	42	126	Santa Rita	P05	Culicidae	Culicinae	*Mansonia*	*Mansonia sp.*	7
2021	Outubro	18/10/2021	42	126	Morrinhos	P04	Culicidae	Culicinae	*Mansonia*	*Mansonia sp.*	15
2021	Outubro	18/10/2021	42	126	Teotônio	P03	Culicidae	Culicinae	*Mansonia*	*Mansonia sp.*	3
2021	Outubro	18/10/2021	42	126	Teotônio	P03	Culicidae	Culicinae	*Coquillettidia*	*Coquillettidia sp.*	2
2021	Outubro	18/10/2021	42	126	Teotônio	P03	Culicidae	Culicinae	*Culex*	*Culex sp.*	1
2021	Outubro	18/10/2021	42	126	São Domingos	P02	Culicidae	Culicinae	*Culex*	*Culex sp.*	1037
2021	Outubro	18/10/2021	42	126	São Domingos	P02	Culicidae	Culicinae	*Coquillettidia*	*Coquillettidia sp.*	57
2021	Outubro	18/10/2021	42	126	São Domingos	P02	Culicidae	Culicinae	*Mansonia*	*Mansonia sp.*	22
2021	Outubro	18/10/2021	42	126	Rio Contra	P07	Culicidae	Culicinae	*Mansonia*	*Mansonia sp.*	2
2021	Outubro	18/10/2021	42	126	Samauma	P08	Culicidae	Culicinae	*Mansonia*	*Mansonia sp.*	15
2021	Outubro	18/10/2021	42	126	Samauma	P08	Culicidae	Culicinae	*Culex*	*Culex sp.*	8
2021	Outubro	18/10/2021	42	126	Jaci Paraná	P06	Culicidae	Culicinae	*Mansonia*	*Mansonia sp.*	8
2021	Outubro	18/10/2021	42	126	Jaci Paraná	P06	Culicidae	Culicinae	*Culex*	*Culex sp.*	15
2021	Outubro	18/10/2021	42	126	Jaci Paraná	P06	Culicidae	Culicinae	*Aedes*	*Aedes sp.*	2
2021	Outubro	18/10/2021	42	126	Santa Rita	P05	Culicidae	Culicinae	*Culex*	*Culex sp.*	3
2021	Outubro	18/10/2021	42	126	Santa Rita	P05	Culicidae	Culicinae	*Mansonia*	*Mansonia sp.*	7
2021	Outubro	18/10/2021	42	126	Morrinhos	P04	Culicidae	Culicinae	*Mansonia*	*Mansonia sp.*	15
2021	Outubro	18/10/2021	42	126	Teotônio	P03	Culicidae	Culicinae	*Mansonia*	*Mansonia sp.*	3
2021	Outubro	18/10/2021	42	126	Teotônio	P03	Culicidae	Culicinae	*Coquillettidia*	*Coquillettidia sp.*	2
2021	Outubro	18/10/2021	42	126	Teotônio	P03	Culicidae	Culicinae	*Culex*	*Culex sp.*	1
2021	Outubro	18/10/2021	42	126	São Domingos	P02	Culicidae	Culicinae	*Culex*	*Culex sp.*	1037
2021	Outubro	18/10/2021	42	126	São Domingos	P02	Culicidae	Culicinae	*Coquillettidia*	*Coquillettidia sp.*	57
2021	Outubro	18/10/2021	42	126	São Domingos	P02	Culicidae	Culicinae	*Mansonia*	*Mansonia sp.*	22
2021	Outubro	25/10/2021	43	127	Rio Contra	P07	Culicidae	Culicinae	*Mansonia*	*Mansonia sp.*	4
2021	Outubro	25/10/2021	43	127	Rio Contra	P07	Culicidae	Anophelinae	*Anopheles*	*Anopheles sp.*	1
2021	Outubro	25/10/2021	43	127	Rio Contra	P07	Culicidae	Culicinae	*Culex*	*Culex sp.*	3
2021	Outubro	25/10/2021	43	127	Samauma	P08	Culicidae	Culicinae	*Mansonia*	*Mansonia sp.*	2
2021	Outubro	25/10/2021	43	127	Samauma	P08	Culicidae	Culicinae	*Culex*	*Culex sp.*	23
2021	Outubro	25/10/2021	43	127	Jaci Paraná	P06	Culicidae	Culicinae	*Aedes*	*Aedes aegypti*	3
2021	Outubro	25/10/2021	43	127	Jaci Paraná	P06	Culicidae	Culicinae	*Mansonia*	*Mansonia sp.*	4
2021	Outubro	25/10/2021	43	127	Jaci Paraná	P06	Culicidae	Culicinae	*Culex*	*Culex sp.*	8
2021	Outubro	25/10/2021	43	127	Santa Rita	P05	Culicidae	Culicinae	*Mansonia*	*Mansonia sp.*	5
2021	Outubro	25/10/2021	43	127	Santa Rita	P05	Culicidae	Culicinae	*Culex*	*Culex sp.*	7
2021	Outubro	25/10/2021	43	127	Morrinhos	P04	Culicidae	Culicinae	*Mansonia*	*Mansonia sp.*	9
2021	Outubro	25/10/2021	43	127	São Domingos	P02	Culicidae	Culicinae	*Mansonia*	*Mansonia sp.*	20
2021	Outubro	25/10/2021	43	127	São Domingos	P02	Culicidae	Culicinae	*Coquillettidia*	*Coquillettidia sp.*	5
2021	Outubro	25/10/2021	43	127	São Domingos	P02	Culicidae	Culicinae	*Culex*	*Culex sp.*	2
2021	Outubro	25/10/2021	43	127	Rio Contra	P07	Culicidae	Culicinae	*Mansonia*	*Mansonia sp.*	4
2021	Outubro	25/10/2021	43	127	Rio Contra	P07	Culicidae	Anophelinae	*Anopheles*	*Anopheles sp.*	1
2021	Outubro	25/10/2021	43	127	Rio Contra	P07	Culicidae	Culicinae	*Culex*	*Culex sp.*	3
2021	Outubro	25/10/2021	43	127	Samauma	P08	Culicidae	Culicinae	*Mansonia*	*Mansonia sp.*	2
2021	Outubro	25/10/2021	43	127	Samauma	P08	Culicidae	Culicinae	*Culex*	*Culex sp.*	23
2021	Outubro	25/10/2021	43	127	Jaci Paraná	P06	Culicidae	Culicinae	*Aedes*	*Aedes aegypti*	3
2021	Outubro	25/10/2021	43	127	Jaci Paraná	P06	Culicidae	Culicinae	*Mansonia*	*Mansonia sp.*	4
2021	Outubro	25/10/2021	43	127	Jaci Paraná	P06	Culicidae	Culicinae	*Culex*	*Culex sp.*	8
2021	Outubro	25/10/2021	43	127	Santa Rita	P05	Culicidae	Culicinae	*Mansonia*	*Mansonia sp.*	5
2021	Outubro	25/10/2021	43	127	Santa Rita	P05	Culicidae	Culicinae	*Culex*	*Culex sp.*	7
2021	Outubro	25/10/2021	43	127	Morrinhos	P04	Culicidae	Culicinae	*Mansonia*	*Mansonia sp.*	9
2021	Outubro	25/10/2021	43	127	São Domingos	P02	Culicidae	Culicinae	*Mansonia*	*Mansonia sp.*	20
2021	Outubro	25/10/2021	43	127	São Domingos	P02	Culicidae	Culicinae	*Coquillettidia*	*Coquillettidia sp.*	5
2021	Outubro	25/10/2021	43	127	São Domingos	P02	Culicidae	Culicinae	*Culex*	*Culex sp.*	2
2021	Novembro	01/11/2021	44	128	Rio Contra	P07	Culicidae	Culicinae	*Mansonia*	*Mansonia sp.*	2
2021	Novembro	01/11/2021	44	128	Samauma	P08	Culicidae	Culicinae	*Mansonia*	*Mansonia sp.*	7
2021	Novembro	01/11/2021	44	128	Samauma	P08	Culicidae	Culicinae	*Culex*	*Culex sp.*	9
2021	Novembro	01/11/2021	44	128	Jaci Paraná	P06	Culicidae	Culicinae	*Aedes*	*Aedes aegypti*	4
2021	Novembro	01/11/2021	44	128	Jaci Paraná	P06	Culicidae	Culicinae	*Culex*	*Culex sp.*	37
2021	Novembro	01/11/2021	44	128	Santa Rita	P05	Culicidae	Culicinae	*Culex*	*Culex sp.*	1
2021	Novembro	01/11/2021	44	128	Santa Rita	P05	Culicidae	Culicinae	*Mansonia*	*Mansonia sp.*	3
2021	Novembro	01/11/2021	44	128	Morrinhos	P04	Culicidae	Culicinae	*Mansonia*	*Mansonia sp.*	7
2021	Novembro	01/11/2021	44	128	Teotônio	P03	Culicidae	Culicinae	*Culex*	*Culex sp.*	4
2021	Novembro	01/11/2021	44	128	São Domingos	P02	Culicidae	Culicinae	*Coquillettidia*	*Coquillettidia sp.*	6
2021	Novembro	01/11/2021	44	128	São Domingos	P02	Culicidae	Culicinae	*Culex*	*Culex sp.*	87
2021	Novembro	01/11/2021	44	128	São Domingos	P02	Culicidae	Culicinae	*Mansonia*	*Mansonia sp.*	12
2021	Novembro	01/11/2021	44	128	Rio Contra	P07	Culicidae	Culicinae	*Mansonia*	*Mansonia sp.*	2
2021	Novembro	01/11/2021	44	128	Samauma	P08	Culicidae	Culicinae	*Mansonia*	*Mansonia sp.*	7
2021	Novembro	01/11/2021	44	128	Samauma	P08	Culicidae	Culicinae	*Culex*	*Culex sp.*	9
2021	Novembro	01/11/2021	44	128	Jaci Paraná	P06	Culicidae	Culicinae	*Aedes*	*Aedes aegypti*	4
2021	Novembro	01/11/2021	44	128	Jaci Paraná	P06	Culicidae	Culicinae	*Culex*	*Culex sp.*	37
2021	Novembro	01/11/2021	44	128	Santa Rita	P05	Culicidae	Culicinae	*Culex*	*Culex sp.*	1
2021	Novembro	01/11/2021	44	128	Santa Rita	P05	Culicidae	Culicinae	*Mansonia*	*Mansonia sp.*	3
2021	Novembro	01/11/2021	44	128	Morrinhos	P04	Culicidae	Culicinae	*Mansonia*	*Mansonia sp.*	7
2021	Novembro	01/11/2021	44	128	Teotônio	P03	Culicidae	Culicinae	*Culex*	*Culex sp.*	4
2021	Novembro	01/11/2021	44	128	São Domingos	P02	Culicidae	Culicinae	*Coquillettidia*	*Coquillettidia sp.*	6
2021	Novembro	01/11/2021	44	128	São Domingos	P02	Culicidae	Culicinae	*Culex*	*Culex sp.*	87
2021	Novembro	01/11/2021	44	128	São Domingos	P02	Culicidae	Culicinae	*Mansonia*	*Mansonia sp.*	12
2021	Novembro	08/11/2021	45	129	Rio Contra	P07	Culicidae	Culicinae	*Mansonia*	*Mansonia sp.*	3
2021	Novembro	08/11/2021	45	129	Rio Contra	P07	Culicidae	Culicinae	*Culex*	*Culex sp.*	12
2021	Novembro	08/11/2021	45	129	Samauma	P08	Culicidae	Culicinae	*Culex*	*Culex sp.*	18
2021	Novembro	08/11/2021	45	129	Samauma	P08	Culicidae	Culicinae	*Mansonia*	*Mansonia sp.*	8
2021	Novembro	08/11/2021	45	129	Jaci Paraná	P06	Culicidae	Culicinae	*Aedes*	*Aedes aegypti*	8
2021	Novembro	08/11/2021	45	129	Jaci Paraná	P06	Culicidae	Culicinae	*Mansonia*	*Mansonia sp.*	5
2021	Novembro	08/11/2021	45	129	Jaci Paraná	P06	Culicidae	Culicinae	*Culex*	*Culex sp.*	17
2021	Novembro	08/11/2021	45	129	Santa Rita	P05	Culicidae	Culicinae	*Culex*	*Culex sp.*	3
2021	Novembro	08/11/2021	45	129	Morrinhos	P04	Culicidae	Culicinae	*Culex*	*Culex sp.*	37
2021	Novembro	08/11/2021	45	129	Morrinhos	P04	Culicidae	Culicinae	*Mansonia*	*Mansonia sp.*	52
2021	Novembro	08/11/2021	45	129	Morrinhos	P04	Culicidae	Culicinae	*Aedes*	*Aedes sp.*	2
2021	Novembro	08/11/2021	45	129	Teotônio	P03	Culicidae	Culicinae	*Mansonia*	*Mansonia sp.*	8
2021	Novembro	08/11/2021	45	129	Teotônio	P03	Culicidae	Culicinae	*Coquillettidia*	*Coquillettidia sp.*	15
2021	Novembro	08/11/2021	45	129	Teotônio	P03	Culicidae	Culicinae	*Culex*	*Culex sp.*	20
2021	Novembro	08/11/2021	45	129	São Domingos	P02	Culicidae	Culicinae	*Culex*	*Culex sp.*	33
2021	Novembro	08/11/2021	45	129	São Domingos	P02	Culicidae	Culicinae	*Mansonia*	*Mansonia sp.*	12

2021	Novembro	08/11/2021	45	129	Cujubim Grande	P01	Culicidae	Culicinae	*Culex*	*Culex sp.*	2
2021	Novembro	08/11/2021	45	129	Cujubim Grande	P01	Culicidae	Culicinae	*Mansonia*	*Mansonia sp.*	1
2021	Novembro	08/11/2021	45	129	Rio Contra	P07	Culicidae	Culicinae	*Mansonia*	*Mansonia sp.*	3
2021	Novembro	08/11/2021	45	129	Rio Contra	P07	Culicidae	Culicinae	*Culex*	*Culex sp.*	12
2021	Novembro	08/11/2021	45	129	Samauma	P08	Culicidae	Culicinae	*Culex*	*Culex sp.*	18
2021	Novembro	08/11/2021	45	129	Samauma	P08	Culicidae	Culicinae	*Mansonia*	*Mansonia sp.*	8
2021	Novembro	08/11/2021	45	129	Jaci Paraná	P06	Culicidae	Culicinae	*Aedes*	*Aedes aegypti*	8
2021	Novembro	08/11/2021	45	129	Jaci Paraná	P06	Culicidae	Culicinae	*Mansonia*	*Mansonia sp.*	5
2021	Novembro	08/11/2021	45	129	Jaci Paraná	P06	Culicidae	Culicinae	*Culex*	*Culex sp.*	17
2021	Novembro	08/11/2021	45	129	Santa Rita	P05	Culicidae	Culicinae	*Culex*	*Culex sp.*	3
2021	Novembro	08/11/2021	45	129	Morrinhos	P04	Culicidae	Culicinae	*Culex*	*Culex sp.*	37
2021	Novembro	08/11/2021	45	129	Morrinhos	P04	Culicidae	Culicinae	*Mansonia*	*Mansonia sp.*	52
2021	Novembro	08/11/2021	45	129	Morrinhos	P04	Culicidae	Culicinae	*Aedes*	*Aedes sp.*	2
2021	Novembro	08/11/2021	45	129	Teotônio	P03	Culicidae	Culicinae	*Mansonia*	*Mansonia sp.*	8
2021	Novembro	08/11/2021	45	129	Teotônio	P03	Culicidae	Culicinae	*Coquillettidia*	*Coquillettidia sp.*	15
2021	Novembro	08/11/2021	45	129	Teotônio	P03	Culicidae	Culicinae	*Culex*	*Culex sp.*	20
2021	Novembro	08/11/2021	45	129	São Domingos	P02	Culicidae	Culicinae	*Culex*	*Culex sp.*	33
2021	Novembro	08/11/2021	45	129	São Domingos	P02	Culicidae	Culicinae	*Mansonia*	*Mansonia sp.*	12
2021	Novembro	08/11/2021	45	129	Cujubim Grande	P01	Culicidae	Culicinae	*Culex*	*Culex sp.*	2
2021	Novembro	08/11/2021	45	129	Cujubim Grande	P01	Culicidae	Culicinae	*Mansonia*	*Mansonia sp.*	1
2021	Novembro	15/11/2021	46	130	Samauma	P08	Culicidae	Culicinae	*Mansonia*	*Mansonia sp.*	10
2021	Novembro	15/11/2021	46	130	Samauma	P08	Culicidae	Culicinae	*Culex*	*Culex sp.*	5
2021	Novembro	15/11/2021	46	130	Samauma	P08	Culicidae	Culicinae	*Coquillettidia*	*Coquillettidia sp.*	2
2021	Novembro	15/11/2021	46	130	Samauma	P08	Culicidae	Culicinae	*Aedes*	*Aedes sp.*	1
2021	Novembro	15/11/2021	46	130	Jaci Paraná	P06	Culicidae	Culicinae	*Culex*	*Culex sp.*	11
2021	Novembro	15/11/2021	46	130	Jaci Paraná	P06	Culicidae	Culicinae	*Aedes*	*Aedes aegypti*	3
2021	Novembro	15/11/2021	46	130	Jaci Paraná	P06	Culicidae	Culicinae	*Mansonia*	*Mansonia sp.*	5
2021	Novembro	15/11/2021	46	130	Santa Rita	P05	Culicidae	Culicinae	*Culex*	*Culex sp.*	2
2021	Novembro	15/11/2021	46	130	Santa Rita	P05	Culicidae	Culicinae	*Mansonia*	*Mansonia sp.*	1
2021	Novembro	15/11/2021	46	130	Morrinhos	P04	Culicidae	Culicinae	*Mansonia*	*Mansonia sp.*	48
2021	Novembro	15/11/2021	46	130	Morrinhos	P04	Culicidae	Culicinae	*Culex*	*Culex sp.*	12
2021	Novembro	15/11/2021	46	130	Morrinhos	P04	Culicidae	Culicinae	*Aedes*	*Aedes sp.*	5
2021	Novembro	15/11/2021	46	130	Morrinhos	P04	Culicidae	Culicinae	*Limatus*	*Limatus durhamii*	2
2021	Novembro	15/11/2021	46	130	Teotônio	P03	Culicidae	Culicinae	*Culex*	*Culex sp.*	22
2021	Novembro	15/11/2021	46	130	Teotônio	P03	Culicidae	Culicinae	*Coquillettidia*	*Coquillettidia sp.*	5
2021	Novembro	15/11/2021	46	130	Teotônio	P03	Culicidae	Culicinae	*Mansonia*	*Mansonia sp.*	3
2021	Novembro	15/11/2021	46	130	São Domingos	P02	Culicidae	Culicinae	*Mansonia*	*Mansonia sp.*	12
2021	Novembro	15/11/2021	46	130	São Domingos	P02	Culicidae	Culicinae	*Culex*	*Culex sp.*	15
2021	Novembro	15/11/2021	46	130	Cujubim Grande	P01	Culicidae	Culicinae	*Culex*	*Culex sp.*	5
2021	Novembro	15/11/2021	46	130	Cujubim Grande	P01	Culicidae	Culicinae	*Mansonia*	*Mansonia sp.*	2
2021	Novembro	15/11/2021	46	130	Cujubim Grande	P01	Culicidae	Culicinae	*Aedes*	*Aedes sp.*	2
2021	Novembro	15/11/2021	46	130	Samauma	P08	Culicidae	Culicinae	*Mansonia*	*Mansonia sp.*	10
2021	Novembro	15/11/2021	46	130	Samauma	P08	Culicidae	Culicinae	*Culex*	*Culex sp.*	5
2021	Novembro	15/11/2021	46	130	Samauma	P08	Culicidae	Culicinae	*Coquillettidia*	*Coquillettidia sp.*	2
2021	Novembro	15/11/2021	46	130	Samauma	P08	Culicidae	Culicinae	*Aedes*	*Aedes sp.*	1
2021	Novembro	15/11/2021	46	130	Jaci Paraná	P06	Culicidae	Culicinae	*Culex*	*Culex sp.*	11
2021	Novembro	15/11/2021	46	130	Jaci Paraná	P06	Culicidae	Culicinae	*Aedes*	*Aedes aegypti*	3
2021	Novembro	15/11/2021	46	130	Jaci Paraná	P06	Culicidae	Culicinae	*Mansonia*	*Mansonia sp.*	5
2021	Novembro	15/11/2021	46	130	Santa Rita	P05	Culicidae	Culicinae	*Culex*	*Culex sp.*	2
2021	Novembro	15/11/2021	46	130	Santa Rita	P05	Culicidae	Culicinae	*Mansonia*	*Mansonia sp.*	1
2021	Novembro	15/11/2021	46	130	Morrinhos	P04	Culicidae	Culicinae	*Mansonia*	*Mansonia sp.*	48
2021	Novembro	15/11/2021	46	136	Morrinhos	P04	Culicidae	Culicinae	*Culex*	*Culex sp.*	12
2021	Novembro	15/11/2021	46	136	Morrinhos	P04	Culicidae	Culicinae	*Aedes*	*Aedes sp.*	5
2021	Novembro	15/11/2021	46	136	Morrinhos	P04	Culicidae	Culicinae	*Limatus*	*Limatus durhamii*	2
2021	Novembro	15/11/2021	46	136	Teotônio	P03	Culicidae	Culicinae	*Culex*	*Culex sp.*	22
2021	Novembro	15/11/2021	46	136	Teotônio	P03	Culicidae	Culicinae	*Coquillettidia*	*Coquillettidia sp.*	5
2021	Novembro	15/11/2021	46	136	Teotônio	P03	Culicidae	Culicinae	*Mansonia*	*Mansonia sp.*	3
2021	Novembro	15/11/2021	46	136	São Domingos	P02	Culicidae	Culicinae	*Mansonia*	*Mansonia sp.*	12
2021	Novembro	15/11/2021	46	136	São Domingos	P02	Culicidae	Culicinae	*Culex*	*Culex sp.*	15
2021	Novembro	15/11/2021	46	136	Cujubim Grande	P01	Culicidae	Culicinae	*Culex*	*Culex sp.*	5
2021	Novembro	15/11/2021	46	136	Cujubim Grande	P01	Culicidae	Culicinae	*Mansonia*	*Mansonia sp.*	2
2021	Novembro	15/11/2021	46	136	Cujubim Grande	P01	Culicidae	Culicinae	*Aedes*	*Aedes sp.*	2
2021	Novembro	22/11/2021	47	136	Rio Contra	P07	Culicidae	Culicinae	*Mansonia*	*Mansonia sp.*	2
2021	Novembro	22/11/2021	47	136	Samauma	P08	Culicidae	Culicinae	*Mansonia*	*Mansonia sp.*	5
2021	Novembro	22/11/2021	47	136	Samauma	P08	Culicidae	Culicinae	*Culex*	*Culex sp.*	1
2021	Novembro	22/11/2021	47	136	Jaci Paraná	P06	Culicidae	Culicinae	*Aedes*	*Aedes aegypti*	2
2021	Novembro	22/11/2021	47	136	Jaci Paraná	P06	Culicidae	Culicinae	*Mansonia*	*Mansonia sp.*	2
2021	Novembro	22/11/2021	47	136	Jaci Paraná	P06	Culicidae	Culicinae	*Culex*	*Culex sp.*	21
2021	Novembro	22/11/2021	47	136	Santa Rita	P05	Culicidae	Culicinae	*Culex*	*Culex sp.*	2
2021	Novembro	22/11/2021	47	136	Santa Rita	P05	Culicidae	Culicinae	*Mansonia*	*Mansonia sp.*	3
2021	Novembro	22/11/2021	47	136	Morrinhos	P04	Culicidae	Culicinae	*Culex*	*Culex sp.*	75
2021	Novembro	22/11/2021	47	136	Morrinhos	P04	Culicidae	Culicinae	*Mansonia*	*Mansonia sp.*	12
2021	Novembro	22/11/2021	47	136	Teotônio	P03	Culicidae	Culicinae	*Culex*	*Culex sp.*	32
2021	Novembro	22/11/2021	47	136	Teotônio	P03	Culicidae	Culicinae	*Coquillettidia*	*Coquillettidia sp.*	10
2021	Novembro	22/11/2021	47	136	Teotônio	P03	Culicidae	Culicinae	*Mansonia*	*Mansonia sp.*	8
2021	Novembro	22/11/2021	47	136	São Domingos	P02	Culicidae	Culicinae	*Culex*	*Culex sp.*	17
2021	Novembro	22/11/2021	47	137	São Domingos	P02	Culicidae	Culicinae	*Mansonia*	*Mansonia sp.*	2
2021	Novembro	22/11/2021	47	137	Rio Contra	P07	Culicidae	Culicinae	*Mansonia*	*Mansonia sp.*	2
2021	Novembro	22/11/2021	47	137	Samauma	P08	Culicidae	Culicinae	*Mansonia*	*Mansonia sp.*	5
2021	Novembro	22/11/2021	47	137	Samauma	P08	Culicidae	Culicinae	*Culex*	*Culex sp.*	1
2021	Novembro	22/11/2021	47	137	Jaci Paraná	P06	Culicidae	Culicinae	*Aedes*	*Aedes aegypti*	2
2021	Novembro	22/11/2021	47	137	Jaci Paraná	P06	Culicidae	Culicinae	*Mansonia*	*Mansonia sp.*	2
2021	Novembro	22/11/2021	47	137	Jaci Paraná	P06	Culicidae	Culicinae	*Culex*	*Culex sp.*	21
2021	Novembro	22/11/2021	47	137	Santa Rita	P05	Culicidae	Culicinae	*Culex*	*Culex sp.*	2
2021	Novembro	22/11/2021	47	137	Santa Rita	P05	Culicidae	Culicinae	*Mansonia*	*Mansonia sp.*	3
2021	Novembro	22/11/2021	47	137	Morrinhos	P04	Culicidae	Culicinae	*Culex*	*Culex sp.*	75
2021	Novembro	22/11/2021	47	137	Morrinhos	P04	Culicidae	Culicinae	*Mansonia*	*Mansonia sp.*	12
2021	Novembro	22/11/2021	47	137	Teotônio	P03	Culicidae	Culicinae	*Culex*	*Culex sp.*	32
2021	Novembro	22/11/2021	47	137	Teotônio	P03	Culicidae	Culicinae	*Coquillettidia*	*Coquillettidia sp.*	10
2021	Novembro	22/11/2021	47	137	Teotônio	P03	Culicidae	Culicinae	*Mansonia*	*Mansonia sp.*	8
2021	Novembro	22/11/2021	47	137	São Domingos	P02	Culicidae	Culicinae	*Culex*	*Culex sp.*	17
2021	Novembro	22/11/2021	47	137	São Domingos	P02	Culicidae	Culicinae	*Mansonia*	*Mansonia amazonensis*	2
2021	Novembro	29/11/2021	48	137	Rio Contra	P07	Culicidae	Culicinae	*Mansonia*	*Mansonia sp.*	7
2021	Novembro	29/11/2021	48	137	Samauma	P08	Culicidae	Culicinae	*Mansonia*	*Mansonia sp.*	2
2021	Novembro	29/11/2021	48	137	Jaci Paraná	P06	Culicidae	Culicinae	*Mansonia*	*Mansonia sp.*	4
2021	Novembro	29/11/2021	48	137	Jaci Paraná	P06	Culicidae	Culicinae	*Aedes*	*Aedes aegypti*	5
2021	Novembro	29/11/2021	48	137	Jaci Paraná	P06	Culicidae	Culicinae	*Culex*	*Culex sp.*	17
2021	Novembro	29/11/2021	48	137	Santa Rita	P05	Culicidae	Culicinae	*Mansonia*	*Mansonia sp.*	2
2021	Novembro	29/11/2021	48	137	Santa Rita	P05	Culicidae	Culicinae	*Culex*	*Culex sp.*	8
2021	Novembro	29/11/2021	48	137	Santa Rita	P05	Culicidae	Culicinae	*Coquillettidia*	*Coquillettidia sp.*	3
2021	Novembro	29/11/2021	48	137	Morrinhos	P04	Culicidae	Culicinae	*Aedes*	*Aedes albopictus*	3
2021	Novembro	29/11/2021	48	137	Morrinhos	P04	Culicidae	Culicinae	*Mansonia*	*Mansonia sp.*	21
2021	Novembro	29/11/2021	48	132	Morrinhos	P04	Culicidae	Culicinae	*Aedes*	*Aedes scapularis*	1
2021	Novembro	29/11/2021	48	132	Morrinhos	P04	Culicidae	Culicinae	*Culex*	*Culex sp.*	98
2021	Novembro	29/11/2021	48	132	São Domingos	P02	Culicidae	Culicinae	*Coquillettidia*	*Coquillettidia sp.*	27
2021	Novembro	29/11/2021	48	132	São Domingos	P02	Culicidae	Culicinae	*Mansonia*	*Mansonia sp.*	18
2021	Novembro	29/11/2021	48	132	São Domingos	P02	Culicidae	Culicinae	*Culex*	*Culex sp.*	43
2021	Novembro	29/11/2021	48	132	São Domingos	P02	Culicidae	Culicinae	*Uranotaenia*	*Uranotaenia sp.*	2
2021	Novembro	29/11/2021	48	132	Cujubim Grande	P01	Culicidae	Culicinae	*Culex*	*Culex sp.*	6
2021	Novembro	29/11/2021	48	132	Cujubim Grande	P01	Culicidae	Culicinae	*Mansonia*	*Mansonia sp.*	3
2021	Novembro	29/11/2021	48	132	Rio Contra	P07	Culicidae	Culicinae	*Mansonia*	*Mansonia sp.*	7
2021	Novembro	29/11/2021	48	132	Samauma	P08	Culicidae	Culicinae	*Mansonia*	*Mansonia sp.*	2
2021	Novembro	29/11/2021	48	132	Jaci Paraná	P06	Culicidae	Culicinae	*Mansonia*	*Mansonia sp.*	4
2021	Novembro	29/11/2021	48	132	Jaci Paraná	P06	Culicidae	Culicinae	*Aedes*	*Aedes aegypti*	5
2021	Novembro	29/11/2021	48	132	Jaci Paraná	P06	Culicidae	Culicinae	*Culex*	*Culex sp.*	17
2021	Novembro	29/11/2021	48	132	Santa Rita	P05	Culicidae	Culicinae	*Mansonia*	*Mansonia sp.*	2
2021	Novembro	29/11/2021	48	132	Santa Rita	P05	Culicidae	Culicinae	*Culex*	*Culex sp.*	8
2021	Novembro	29/11/2021	48	132	Santa Rita	P05	Culicidae	Culicinae	*Coquillettidia*	*Coquillettidia sp.*	3

2021	Novembro	29/11/2021	48	132	Morrinhos	P04	Culicidae	Culicinae	*Aedes*	*Aedes albopictus*	3
2021	Novembro	29/11/2021	48	132	Morrinhos	P04	Culicidae	Culicinae	*Mansonia*	*Mansonia sp.*	21
2021	Novembro	29/11/2021	48	132	Morrinhos	P04	Culicidae	Culicinae	*Aedes*	*Aedes scapularis*	1
2021	Novembro	29/11/2021	48	132	Morrinhos	P04	Culicidae	Culicinae	*Culex*	*Culex sp.*	98
2021	Novembro	29/11/2021	48	132	São Domingos	P02	Culicidae	Culicinae	*Coquillettidia*	*Coquillettidia sp.*	27
2021	Novembro	29/11/2021	48	132	São Domingos	P02	Culicidae	Culicinae	*Mansonia*	*Mansonia sp.*	18
2021	Novembro	29/11/2021	48	132	São Domingos	P02	Culicidae	Culicinae	*Culex*	*Culex sp.*	43
2021	Novembro	29/11/2021	48	132	São Domingos	P02	Culicidae	Culicinae	*Uranotaenia*	*Uranotaenia sp.*	2
2021	Novembro	29/11/2021	48	132	Cujubim Grande	P01	Culicidae	Culicinae	*Culex*	*Culex sp.*	6
2021	Novembro	29/11/2021	48	132	Cujubim Grande	P01	Culicidae	Culicinae	*Mansonia*	*Mansonia sp.*	3
2021	Dezembro	06/12/2021	49	133	Rio Contra	P07	Culicidae	Culicinae	*Mansonia*	*Mansonia sp.*	12
2021	Dezembro	06/12/2021	49	133	Rio Contra	P07	Culicidae	Anophelinae	*Anopheles*	*Anopheles sp.*	5
2021	Dezembro	06/12/2021	49	133	Rio Contra	P07	Culicidae	Anophelinae	*Anopheles*	*Anopheles darlingi*	1
2021	Dezembro	06/12/2021	49	133	Samauma	P08	Culicidae	Culicinae	*Mansonia*	*Mansonia sp.*	3
2021	Dezembro	06/12/2021	49	133	Samauma	P08	Culicidae	Culicinae	*Culex*	*Culex sp.*	1
2021	Dezembro	06/12/2021	49	133	Jaci Paraná	P06	Culicidae	Culicinae	*Culex*	*Culex sp.*	5
2021	Dezembro	06/12/2021	49	133	Jaci Paraná	P06	Culicidae	Culicinae	*Mansonia*	*Mansonia sp.*	2
2021	Dezembro	06/12/2021	49	133	Jaci Paraná	P06	Culicidae	Culicinae	*Aedes*	*Aedes aegypti*	1
2021	Dezembro	06/12/2021	49	133	Santa Rita	P05	Culicidae	Culicinae	*Mansonia*	*Mansonia sp.*	22
2021	Dezembro	06/12/2021	49	133	Santa Rita	P05	Culicidae	Culicinae	*Culex*	*Culex sp.*	15
2021	Dezembro	06/12/2021	49	133	Morrinhos	P04	Culicidae	Culicinae	*Mansonia*	*Mansonia sp.*	67
2021	Dezembro	06/12/2021	49	133	Morrinhos	P04	Culicidae	Culicinae	*Culex*	*Culex sp.*	12
2021	Dezembro	06/12/2021	49	133	Teotônio	P03	Culicidae	Culicinae	*Coquillettidia*	*Coquillettidia sp.*	5
2021	Dezembro	06/12/2021	49	133	Teotônio	P03	Culicidae	Culicinae	*Culex*	*Culex sp.*	10
2021	Dezembro	06/12/2021	49	133	Teotônio	P03	Culicidae	Culicinae	*Mansonia*	*Mansonia sp.*	1
2021	Dezembro	06/12/2021	49	133	São Domingos	P02	Culicidae	Culicinae	*Mansonia*	*Mansonia sp.*	33
2021	Dezembro	06/12/2021	49	133	São Domingos	P02	Culicidae	Culicinae	*Culex*	*Culex sp.*	8
2021	Dezembro	06/12/2021	49	133	São Domingos	P02	Culicidae	Culicinae	*Aedeomyia*	*Adeomyia squamipennis*	2
2021	Dezembro	06/12/2021	49	133	Cujubim Grande	P01	Culicidae	Culicinae	*Culex*	*Culex sp.*	2
2021	Dezembro	06/12/2021	49	133	Rio Contra	P07	Culicidae	Culicinae	*Mansonia*	*Mansonia sp.*	12
2021	Dezembro	06/12/2021	49	133	Rio Contra	P07	Culicidae	Anophelinae	*Anopheles*	*Anopheles sp.*	5
2021	Dezembro	06/12/2021	49	133	Rio Contra	P07	Culicidae	Anophelinae	*Anopheles*	*Anopheles darlingi*	1
2021	Dezembro	06/12/2021	49	133	Samauma	P08	Culicidae	Culicinae	*Mansonia*	*Mansonia sp.*	3
2021	Dezembro	06/12/2021	49	133	Samauma	P08	Culicidae	Culicinae	*Culex*	*Culex sp.*	1
2021	Dezembro	06/12/2021	49	133	Jaci Paraná	P06	Culicidae	Culicinae	*Culex*	*Culex sp.*	5
2021	Dezembro	06/12/2021	49	133	Jaci Paraná	P06	Culicidae	Culicinae	*Mansonia*	*Mansonia sp.*	2
2021	Dezembro	06/12/2021	49	133	Jaci Paraná	P06	Culicidae	Culicinae	*Aedes*	*Aedes aegypti*	1
2021	Dezembro	06/12/2021	49	133	Santa Rita	P05	Culicidae	Culicinae	*Mansonia*	*Mansonia sp.*	22
2021	Dezembro	06/12/2021	49	133	Santa Rita	P05	Culicidae	Culicinae	*Culex*	*Culex sp.*	15
2021	Dezembro	06/12/2021	49	133	Morrinhos	P04	Culicidae	Culicinae	*Mansonia*	*Mansonia sp.*	67
2021	Dezembro	06/12/2021	49	133	Morrinhos	P04	Culicidae	Culicinae	*Culex*	*Culex sp.*	12
2021	Dezembro	06/12/2021	49	133	Teotônio	P03	Culicidae	Culicinae	*Coquillettidia*	*Coquillettidia sp.*	5
2021	Dezembro	06/12/2021	49	133	Teotônio	P03	Culicidae	Culicinae	*Culex*	*Culex sp.*	10
2021	Dezembro	06/12/2021	49	133	Teotônio	P03	Culicidae	Culicinae	*Mansonia*	*Mansonia sp.*	1
2021	Dezembro	06/12/2021	49	133	São Domingos	P02	Culicidae	Culicinae	*Mansonia*	*Mansonia sp.*	33
2021	Dezembro	06/12/2021	49	133	São Domingos	P02	Culicidae	Culicinae	*Culex*	*Culex sp.*	8
2021	Dezembro	06/12/2021	49	133	São Domingos	P02	Culicidae	Culicinae	*Aedeomyia*	*Adeomyia squamipennis*	2
2021	Dezembro	06/12/2021	49	133	Cujubim Grande	P01	Culicidae	Culicinae	*Culex*	*Culex sp.*	2
2021	Dezembro	13/12/2021	50	134	Rio Contra	P07	Culicidae	Culicinae	*Mansonia*	*Mansonia sp.*	28
2021	Dezembro	13/12/2021	50	134	Rio Contra	P07	Culicidae	Culicinae	*Limatus*	*Limatus durhamii*	1
2021	Dezembro	13/12/2021	50	134	Rio Contra	P07	Culicidae	Culicinae	*Culex*	*Culex sp.*	5
2021	Dezembro	13/12/2021	50	134	Samauma	P08	Culicidae	Culicinae	*Mansonia*	*Mansonia sp.*	3
2021	Dezembro	13/12/2021	50	134	Jaci Paraná	P06	Culicidae	Culicinae	*Aedes*	*Aedes aegypti*	5
2021	Dezembro	13/12/2021	50	134	Jaci Paraná	P06	Culicidae	Culicinae	*Culex*	*Culex sp.*	12
2021	Dezembro	13/12/2021	50	134	Jaci Paraná	P06	Culicidae	Culicinae	*Mansonia*	*Mansonia sp.*	7
2021	Dezembro	13/12/2021	50	134	Santa Rita	P05	Culicidae	Culicinae	*Mansonia*	*Mansonia sp.*	1
2021	Dezembro	13/12/2021	50	134	Santa Rita	P05	Culicidae	Culicinae	*Culex*	*Culex sp.*	1
2021	Dezembro	13/12/2021	50	134	Morrinhos	P04	Culicidae	Culicinae	*Mansonia*	*Mansonia sp.*	37
2021	Dezembro	13/12/2021	50	134	Morrinhos	P04	Culicidae	Culicinae	*Culex*	*Culex sp.*	5
2021	Dezembro	13/12/2021	50	134	Teotônio	P03	Culicidae	Culicinae	*Culex*	*Culex sp.*	12
2021	Dezembro	13/12/2021	50	134	Teotônio	P03	Culicidae	Culicinae	*Mansonia*	*Mansonia sp.*	2
2021	Dezembro	13/12/2021	50	134	São Domingos	P02	Culicidae	Culicinae	*Mansonia*	*Mansonia sp.*	34
2021	Dezembro	13/12/2021	50	134	São Domingos	P02	Culicidae	Culicinae	*Culex*	*Culex sp.*	5
2021	Dezembro	13/12/2021	50	134	Cujubim Grande	P01	Culicidae	Culicinae	*Culex*	*Culex sp.*	2
2021	Dezembro	13/12/2021	50	134	Rio Contra	P07	Culicidae	Culicinae	*Mansonia*	*Mansonia sp.*	28
2021	Dezembro	13/12/2021	50	134	Rio Contra	P07	Culicidae	Culicinae	*Limatus*	*Limatus durhamii*	1
2021	Dezembro	13/12/2021	50	134	Rio Contra	P07	Culicidae	Culicinae	*Culex*	*Culex sp.*	5
2021	Dezembro	13/12/2021	50	134	Samauma	P08	Culicidae	Culicinae	*Mansonia*	*Mansonia sp.*	3
2021	Dezembro	13/12/2021	50	134	Jaci Paraná	P06	Culicidae	Culicinae	*Aedes*	*Aedes aegypti*	5
2021	Dezembro	13/12/2021	50	134	Jaci Paraná	P06	Culicidae	Culicinae	*Culex*	*Culex sp.*	12
2021	Dezembro	13/12/2021	50	134	Jaci Paraná	P06	Culicidae	Culicinae	*Mansonia*	*Mansonia sp.*	7
2021	Dezembro	13/12/2021	50	134	Santa Rita	P05	Culicidae	Culicinae	*Mansonia*	*Mansonia sp.*	1
2021	Dezembro	13/12/2021	50	134	Santa Rita	P05	Culicidae	Culicinae	*Culex*	*Culex sp.*	1
2021	Dezembro	13/12/2021	50	134	Morrinhos	P04	Culicidae	Culicinae	*Mansonia*	*Mansonia sp.*	37
2021	Dezembro	13/12/2021	50	134	Morrinhos	P04	Culicidae	Culicinae	*Culex*	*Culex sp.*	5
2021	Dezembro	13/12/2021	50	134	Teotônio	P03	Culicidae	Culicinae	*Culex*	*Culex sp.*	12
2021	Dezembro	13/12/2021	50	134	Teotônio	P03	Culicidae	Culicinae	*Mansonia*	*Mansonia sp.*	2
2021	Dezembro	13/12/2021	50	134	São Domingos	P02	Culicidae	Culicinae	*Mansonia*	*Mansonia sp.*	34
2021	Dezembro	13/12/2021	50	134	São Domingos	P02	Culicidae	Culicinae	*Culex*	*Culex sp.*	5
2021	Dezembro	13/12/2021	50	134	Cujubim Grande	P01	Culicidae	Culicinae	*Culex*	*Culex sp.*	2
2021	Dezembro	20/12/2021	51	135	Rio Contra	P07	Culicidae	Culicinae	*Mansonia*	*Mansonia sp.*	25
2021	Dezembro	20/12/2021	51	135	Rio Contra	P07	Culicidae	Culicinae	*Culex*	*Culex sp.*	12
2021	Dezembro	20/12/2021	51	135	Rio Contra	P07	Culicidae	Anophelinae	*Anopheles*	*Anopheles darlingi*	3
2021	Dezembro	20/12/2021	51	135	Samauma	P08	Culicidae	Culicinae	*Mansonia*	*Mansonia sp.*	8
2021	Dezembro	20/12/2021	51	135	Samauma	P08	Culicidae	Culicinae	*Culex*	*Culex sp.*	1
2021	Dezembro	20/12/2021	51	135	Jaci Paraná	P06	Culicidae	Culicinae	*Mansonia*	*Mansonia sp.*	3
2021	Dezembro	20/12/2021	51	135	Jaci Paraná	P06	Culicidae	Culicinae	*Culex*	*Culex sp.*	2
2021	Dezembro	20/12/2021	51	135	Jaci Paraná	P06	Culicidae	Culicinae	*Aedes*	*Aedes aegypti*	1
2021	Dezembro	20/12/2021	51	135	Santa Rita	P05	Culicidae	Culicinae	*Mansonia*	*Mansonia sp.*	5
2021	Dezembro	20/12/2021	51	135	Santa Rita	P05	Culicidae	Culicinae	*Culex*	*Culex sp.*	2
2021	Dezembro	20/12/2021	51	135	Morrinhos	P04	Culicidae	Culicinae	*Mansonia*	*Mansonia sp.*	12
2021	Dezembro	20/12/2021	51	135	Morrinhos	P04	Culicidae	Culicinae	*Culex*	*Culex sp.*	5
2021	Dezembro	20/12/2021	51	135	Morrinhos	P04	Culicidae	Culicinae	*Limatus*	*Limatus durhamii*	1
2021	Dezembro	20/12/2021	51	135	Teotônio	P03	Culicidae	Culicinae	*Mansonia*	*Mansonia sp.*	5
2021	Dezembro	20/12/2021	51	135	Teotônio	P03	Culicidae	Culicinae	*Coquillettidia*	*Coquillettidia sp.*	3
2021	Dezembro	20/12/2021	51	135	Teotônio	P03	Culicidae	Culicinae	*Culex*	*Culex sp.*	7
2021	Dezembro	20/12/2021	51	135	São Domingos	P02	Culicidae	Culicinae	*Mansonia*	*Mansonia sp.*	21
2021	Dezembro	20/12/2021	51	135	São Domingos	P02	Culicidae	Culicinae	*Culex*	*Culex sp.*	15
2021	Dezembro	20/12/2021	51	135	Cujubim Grande	P01	Culicidae	Culicinae	*Culex*	*Culex sp.*	1
2021	Dezembro	20/12/2021	51	135	Rio Contra	P07	Culicidae	Culicinae	*Mansonia*	*Mansonia sp.*	25
2021	Dezembro	20/12/2021	51	135	Rio Contra	P07	Culicidae	Culicinae	*Culex*	*Culex sp.*	12
2021	Dezembro	20/12/2021	51	135	Rio Contra	P07	Culicidae	Anophelinae	*Anopheles*	*Anopheles darlingi*	3
2021	Dezembro	20/12/2021	51	135	Samauma	P08	Culicidae	Culicinae	*Mansonia*	*Mansonia sp.*	8
2021	Dezembro	20/12/2021	51	135	Samauma	P08	Culicidae	Culicinae	*Culex*	*Culex sp.*	1
2021	Dezembro	20/12/2021	51	135	Jaci Paraná	P06	Culicidae	Culicinae	*Mansonia*	*Mansonia titillans*	3
2021	Dezembro	20/12/2021	51	135	Jaci Paraná	P06	Culicidae	Culicinae	*Culex*	*Culex sp.*	2
2021	Dezembro	20/12/2021	51	135	Jaci Paraná	P06	Culicidae	Culicinae	*Aedes*	*Aedes aegypti*	1
2021	Dezembro	20/12/2021	51	135	Santa Rita	P05	Culicidae	Culicinae	*Mansonia*	*Mansonia sp.*	5
2021	Dezembro	20/12/2021	51	135	Santa Rita	P05	Culicidae	Culicinae	*Culex*	*Culex sp.*	2
2021	Dezembro	20/12/2021	51	135	Morrinhos	P04	Culicidae	Culicinae	*Mansonia*	*Mansonia sp.*	12
2021	Dezembro	20/12/2021	51	135	Morrinhos	P04	Culicidae	Culicinae	*Culex*	*Culex sp.*	5
2021	Dezembro	20/12/2021	51	135	Morrinhos	P04	Culicidae	Culicinae	*Limatus*	*Limatus durhamii*	1
2021	Dezembro	20/12/2021	51	135	Teotônio	P03	Culicidae	Culicinae	*Mansonia*	*Mansonia sp.*	5
2021	Dezembro	20/12/2021	51	135	Teotônio	P03	Culicidae	Culicinae	*Coquillettidia*	*Coquillettidia sp.*	3
2021	Dezembro	20/12/2021	51	135	Teotônio	P03	Culicidae	Culicinae	*Culex*	*Culex sp.*	7
2021	Dezembro	20/12/2021	51	135	São Domingos	P02	Culicidae	Culicinae	*Mansonia*	*Mansonia sp.*	21
2021	Dezembro	20/12/2021	51	135	São Domingos	P02	Culicidae	Culicinae	*Culex*	*Culex sp.*	15
2021	Dezembro	20/12/2021	51	135	Cujubim Grande	P01	Culicidae	Culicinae	*Culex*	*Culex sp.*	1

2021	Dezembro	27/12/2021	52	136	Rio Contra	P07	Culicidae	Culicinae	*Mansonia*	*Mansonia sp.*	27
2021	Dezembro	27/12/2021	52	136	Rio Contra	P07	Culicidae	Culicinae	*Culex*	*Culex sp.*	10
2021	Dezembro	27/12/2021	52	136	Rio Contra	P07	Culicidae	Anophelinae	*Anopheles*	*Anopheles sp.*	3
2021	Dezembro	27/12/2021	52	136	Samauma	P08	Culicidae	Culicinae	*Mansonia*	*Mansonia sp.*	2
2021	Dezembro	27/12/2021	52	136	Jaci Paraná	P06	Culicidae	Culicinae	*Culex*	*Culex sp.*	33
2021	Dezembro	27/12/2021	52	136	Jaci Paraná	P06	Culicidae	Culicinae	*Mansonia*	*Mansonia sp.*	15
2021	Dezembro	27/12/2021	52	136	Jaci Paraná	P06	Culicidae	Culicinae	*Aedes*	*Aedes aegypti*	7
2021	Dezembro	27/12/2021	52	136	Santa Rita	P05	Culicidae	Culicinae	*Mansonia*	*Mansonia sp.*	7
2021	Dezembro	27/12/2021	52	136	Santa Rita	P05	Culicidae	Culicinae	*Culex*	*Culex sp.*	2
2021	Dezembro	27/12/2021	52	136	Santa Rita	P05	Culicidae	Culicinae	*Aedes*	*Aedes sp.*	1
2021	Dezembro	27/12/2021	52	136	Morrinhos	P04	Culicidae	Culicinae	*Mansonia*	*Mansonia sp.*	18
2021	Dezembro	27/12/2021	52	136	Morrinhos	P04	Culicidae	Culicinae	*Culex*	*Culex sp.*	7
2021	Dezembro	27/12/2021	52	136	Teotônio	P03	Culicidae	Culicinae	*Culex*	*Culex sp.*	5
2021	Dezembro	27/12/2021	52	136	Teotônio	P03	Culicidae	Culicinae	*Coquillettidia*	*Coquillettidia sp.*	3
2021	Dezembro	27/12/2021	52	136	Teotônio	P03	Culicidae	Culicinae	*Mansonia*	*Mansonia sp.*	2
2021	Dezembro	27/12/2021	52	136	São Domingos	P02	Culicidae	Culicinae	*Mansonia*	*Mansonia sp.*	43
2021	Dezembro	27/12/2021	52	136	São Domingos	P02	Culicidae	Culicinae	*Culex*	*Culex sp.*	21
2021	Dezembro	27/12/2021	52	136	São Domingos	P02	Culicidae	Culicinae	*Coquillettidia*	*Coquillettidia sp.*	2
2021	Dezembro	27/12/2021	52	136	São Domingos	P02	Culicidae	Culicinae	*Aedeomyia*	*Adeomyia squamipennis*	3
2021	Dezembro	27/12/2021	52	136	Rio Contra	P07	Culicidae	Culicinae	*Mansonia*	*Mansonia sp.*	27
2021	Dezembro	27/12/2021	52	136	Rio Contra	P07	Culicidae	Culicinae	*Culex*	*Culex sp.*	10
2021	Dezembro	27/12/2021	52	136	Rio Contra	P07	Culicidae	Anophelinae	*Anopheles*	*Anopheles sp.*	3
2021	Dezembro	27/12/2021	52	136	Samauma	P08	Culicidae	Culicinae	*Mansonia*	*Mansonia sp.*	2
2021	Dezembro	27/12/2021	52	136	Jaci Paraná	P06	Culicidae	Culicinae	*Culex*	*Culex sp.*	33
2021	Dezembro	27/12/2021	52	136	Jaci Paraná	P06	Culicidae	Culicinae	*Mansonia*	*Mansonia sp.*	15
2021	Dezembro	27/12/2021	52	136	Jaci Paraná	P06	Culicidae	Culicinae	*Aedes*	*Aedes aegypti*	7
2021	Dezembro	27/12/2021	52	136	Santa Rita	P05	Culicidae	Culicinae	*Mansonia*	*Mansonia sp.*	7
2021	Dezembro	27/12/2021	52	136	Santa Rita	P05	Culicidae	Culicinae	*Culex*	*Culex sp.*	2
2021	Dezembro	27/12/2021	52	136	Santa Rita	P05	Culicidae	Culicinae	*Aedes*	*Aedes sp.*	1
2021	Dezembro	27/12/2021	52	136	Morrinhos	P04	Culicidae	Culicinae	*Mansonia*	*Mansonia sp.*	18
2021	Dezembro	27/12/2021	52	136	Morrinhos	P04	Culicidae	Culicinae	*Culex*	*Culex sp.*	7
2021	Dezembro	27/12/2021	52	136	Teotônio	P03	Culicidae	Culicinae	*Culex*	*Culex sp.*	5
2021	Dezembro	27/12/2021	52	136	Teotônio	P03	Culicidae	Culicinae	*Coquillettidia*	*Coquillettidia sp.*	3
2021	Dezembro	27/12/2021	52	136	Teotônio	P03	Culicidae	Culicinae	*Mansonia*	*Mansonia sp.*	2
2021	Dezembro	27/12/2021	52	136	São Domingos	P02	Culicidae	Culicinae	*Mansonia*	*Mansonia sp.*	43
2021	Dezembro	27/12/2021	52	136	São Domingos	P02	Culicidae	Culicinae	*Culex*	*Culex sp.*	21
2021	Dezembro	27/12/2021	52	136	São Domingos	P02	Culicidae	Culicinae	*Coquillettidia*	*Coquillettidia sp.*	2
2021	Dezembro	27/12/2021	52	136	São Domingos	P02	Culicidae	Culicinae	*Aedeomyia*	*Adeomyia squamipennis*	3
2022	Janeiro	03/01/2022	1	137	Samauma	P08	Culicidae	Culicinae	*Mansonia*	*Mansonia sp.*	22
2022	Janeiro	03/01/2022	1	137	Samauma	P08	Culicidae	Anophelinae	*Anopheles*	*Anopheles sp.*	1
2022	Janeiro	03/01/2022	1	137	Samauma	P08	Culicidae	Culicinae	*Coquillettidia*	*Coquillettidia sp.*	1
2022	Janeiro	03/01/2022	1	137	Morrinhos	P04	Culicidae	Culicinae	*Culex*	*Culex sp.*	48
2022	Janeiro	03/01/2022	1	137	Morrinhos	P04	Culicidae	Culicinae	*Mansonia*	*Mansonia humeralis*	5
2022	Janeiro	03/01/2022	1	137	Teotônio	P03	Culicidae	Culicinae	*Mansonia*	*Mansonia sp.*	1
2022	Janeiro	03/01/2022	1	137	São Domingos	P02	Culicidae	Culicinae	*Mansonia*	*Mansonia sp.*	12
2022	Janeiro	03/01/2022	1	137	São Domingos	P02	Culicidae	Culicinae	*Culex*	*Culex sp.*	16
2022	Janeiro	03/01/2022	1	137	São Domingos	P02	Culicidae	Culicinae	*Coquillettidia*	*Coquillettidia sp.*	6
2022	Janeiro	03/01/2022	1	137	São Domingos	P02	Culicidae	Culicinae	*Uranotaenia*	*Uranotaenia sp.*	6
2022	Janeiro	03/01/2022	1	137	São Domingos	P02	Culicidae	Culicinae	*Aedeomyia*	*Adeomyia squamipennis*	2
2022	Janeiro	03/01/2022	1	137	São Domingos	P02	Culicidae	Anophelinae	*Anopheles*	*Anopheles sp.*	5
2022	Janeiro	03/01/2022	1	137	Santa Rita	P05	Culicidae	Culicinae	*Mansonia*	*Mansonia sp.*	17
2022	Janeiro	03/01/2022	1	137	Santa Rita	P05	Culicidae	Culicinae	*Culex*	*Culex sp.*	32
2022	Janeiro	03/01/2022	1	137	Santa Rita	P05	Culicidae	Culicinae	*Coquillettidia*	*Coquillettidia sp.*	1
2022	Janeiro	03/01/2022	1	137	Santa Rita	P05	Culicidae	Culicinae	*Uranotaenia*	*Uranotaenia sp.*	1
2022	Janeiro	03/01/2022	1	137	Santa Rita	P05	Culicidae	Anophelinae	*Anopheles*	*Anopheles sp.*	5
2022	Janeiro	03/01/2022	1	137	Rio Contra	P07	Culicidae	Culicinae	*Mansonia*	*Mansonia sp.*	77
2022	Janeiro	03/01/2022	1	137	Rio Contra	P07	Culicidae	Culicinae	*Culex*	*Culex sp.*	1
2022	Janeiro	03/01/2022	1	137	Rio Contra	P07	Culicidae	Anophelinae	*Anopheles*	*Anopheles sp.*	1
2022	Janeiro	03/01/2022	1	137	Jaci Paraná	P06	Culicidae	Culicinae	*Mansonia*	*Mansonia sp.*	3
2022	Janeiro	03/01/2022	1	137	Jaci Paraná	P06	Culicidae	Culicinae	*Culex*	*Culex sp.*	10
2022	Janeiro	03/01/2022	1	137	Jaci Paraná	P06	Culicidae	Anophelinae	*Anopheles*	*Anopheles sp.*	1
2022	Janeiro	03/01/2022	1	137	Jaci Paraná	P06	Culicidae	Culicinae	*Aedes*	*Aedes sp.*	1
2022	Janeiro	03/01/2022	1	137	Jaci Paraná	P06	Culicidae	Culicinae	*Aedes*	*Aedes albopictus*	3
2022	Janeiro	10/01/2022	2	138	Rio Contra	P07	Culicidae	Culicinae	*Mansonia*	*Mansonia sp.*	52
2022	Janeiro	10/01/2022	2	138	Rio Contra	P07	Culicidae	Anophelinae	*Anopheles*	*Anopheles nuneztovari s.l.*	1
2022	Janeiro	10/01/2022	2	138	Rio Contra	P07	Culicidae	Culicinae	*Culex*	*Culex sp.*	4
2022	Janeiro	10/01/2022	2	138	Santa Rita	P05	Culicidae	Culicinae	*Mansonia*	*Mansonia sp.*	18
2022	Janeiro	10/01/2022	2	138	Santa Rita	P05	Culicidae	Culicinae	*Culex*	*Culex sp.*	2
2022	Janeiro	10/01/2022	2	138	Jaci Paraná	P06	Culicidae	Culicinae	*Mansonia*	*Mansonia sp.*	8
2022	Janeiro	10/01/2022	2	138	Jaci Paraná	P06	Culicidae	Culicinae	*Culex*	*Culex sp.*	22
2022	Janeiro	10/01/2022	2	138	Jaci Paraná	P06	Culicidae	Culicinae	*Uranotaenia*	*Uranotaenia sp.*	1
2022	Janeiro	10/01/2022	2	138	Jaci Paraná	P06	Culicidae	Culicinae	*Limatus*	*Limatus durhamii*	1
2022	Janeiro	10/01/2022	2	138	Jaci Paraná	P06	Culicidae	Anophelinae	*Anopheles*	*Anopheles sp.*	1
2022	Janeiro	10/01/2022	2	138	Jaci Paraná	P06	Culicidae	Culicinae	*Aedes*	*Aedes scapularis*	3
2022	Janeiro	10/01/2022	2	138	Morrinhos	P04	Culicidae	Culicinae	*Mansonia*	*Mansonia sp.*	6
2022	Janeiro	10/01/2022	2	138	Morrinhos	P04	Culicidae	Culicinae	*Culex*	*Culex sp.*	26
2022	Janeiro	10/01/2022	2	138	Samauma	P08	Culicidae	Culicinae	*Mansonia*	*Mansonia sp.*	15
2022	Janeiro	10/01/2022	2	138	Samauma	P08	Culicidae	Anophelinae	*Anopheles*	*Anopheles darlingi*	1
2022	Janeiro	10/01/2022	2	138	Samauma	P08	Culicidae	Anophelinae	*Anopheles*	*Anopheles triannulatus*	1
2022	Janeiro	10/01/2022	2	138	Samauma	P08	Culicidae	Culicinae	*Wyeomyia*	*Wyeomyia sp.*	1
2022	Janeiro	12/01/2022	2	138	Teotônio	P03	Culicidae	Culicinae	*Mansonia*	*Mansonia sp.*	8
2022	Janeiro	12/01/2022	2	138	Teotônio	P03	Culicidae	Culicinae	*Culex*	*Culex sp.*	6
2022	Janeiro	12/01/2022	2	138	Teotônio	P03	Culicidae	Culicinae	*Coquillettidia*	*Coquillettidia sp.*	10
2022	Janeiro	12/01/2022	2	138	Teotônio	P03	Culicidae	Anophelinae	*Anopheles*	*Anopheles sp.*	3
2022	Janeiro	12/01/2022	2	138	São Domingos	P02	Culicidae	Culicinae	*Mansonia*	*Mansonia sp.*	37
2022	Janeiro	12/01/2022	2	138	São Domingos	P02	Culicidae	Culicinae	*Coquillettidia*	*Coquillettidia venezuelensis*	15
2022	Janeiro	12/01/2022	2	138	São Domingos	P02	Culicidae	Culicinae	*Culex*	*Culex sp.*	20
2022	Janeiro	12/01/2022	2	138	São Domingos	P02	Culicidae	Culicinae	*Uranotaenia*	*Uranotaenia sp.*	6
2022	Janeiro	12/01/2022	2	138	São Domingos	P02	Culicidae	Anophelinae	*Anopheles*	*Anopheles sp.*	1
2022	Janeiro	17/01/2022	3	139	Teotônio	P03	Culicidae	Culicinae	*Mansonia*	*Mansonia sp.*	5
2022	Janeiro	17/01/2022	3	139	Teotônio	P03	Culicidae	Anophelinae	*Anopheles*	*Anopheles sp.*	5
2022	Janeiro	17/01/2022	3	139	Teotônio	P03	Culicidae	Culicinae	*Culex*	*Culex sp.*	1
2022	Janeiro	17/01/2022	3	139	Teotônio	P03	Culicidae	Culicinae	*Coquillettidia*	*Coquillettidia sp.*	8
2022	Janeiro	17/01/2022	3	139	Rio Contra	P07	Culicidae	Culicinae	*Mansonia*	*Mansonia indubitans*	2
2022	Janeiro	17/01/2022	3	139	Jaci Paraná	P06	Culicidae	Culicinae	*Culex*	*Culex sp.*	6
2022	Janeiro	17/01/2022	3	139	Morrinhos	P04	Culicidae	Culicinae	*Culex*	*Culex sp.*	3
2022	Janeiro	17/01/2022	3	139	Samauma	P08	Culicidae	Culicinae	*Mansonia*	*Mansonia sp.*	17
2022	Janeiro	17/01/2022	3	139	Samauma	P08	Culicidae	Culicinae	*Aedeomyia*	*Adeomyia squamipennis*	2
2022	Janeiro	17/01/2022	3	139	Samauma	P08	Culicidae	Culicinae	*Coquillettidia*	*Coquillettidia sp.*	2
2022	Janeiro	17/01/2022	3	139	Santa Rita	P05	Culicidae	Culicinae	*Culex*	*Culex sp.*	15
2022	Janeiro	17/01/2022	3	139	Santa Rita	P05	Culicidae	Culicinae	*Mansonia*	*Mansonia sp.*	4
2022	Janeiro	17/01/2022	3	139	São Domingos	P02	Culicidae	Culicinae	*Culex*	*Culex sp.*	8
2022	Janeiro	17/01/2022	3	139	Cujubim Grande	P01	Culicidae	Culicinae	*Culex*	*Culex sp.*	1
2022	Janeiro	24/01/2022	4	140	Samauma	P08	Culicidae	Culicinae	*Mansonia*	*Mansonia sp.*	5
2022	Janeiro	24/01/2022	4	140	Samauma	P08	Culicidae	Culicinae	*Culex*	*Culex sp.*	2
2022	Janeiro	24/01/2022	4	140	Samauma	P08	Culicidae	Culicinae	*Aedeomyia*	*Adeomyia squamipennis*	2
2022	Janeiro	24/01/2022	4	140	Jaci Paraná	P06	Culicidae	Culicinae	*Mansonia*	*Mansonia sp.*	3
2022	Janeiro	24/01/2022	4	140	Jaci Paraná	P06	Culicidae	Culicinae	*Aedes*	*Aedes sp.*	2
2022	Janeiro	24/01/2022	4	140	Santa Rita	P05	Culicidae	Culicinae	*Mansonia*	*Mansonia sp.*	2
2022	Janeiro	24/01/2022	4	140	Santa Rita	P05	Culicidae	Culicinae	*Culex*	*Culex sp.*	3
2022	Janeiro	24/01/2022	4	140	Morrinhos	P04	Culicidae	Culicinae	*Mansonia*	*Mansonia sp.*	2
2022	Janeiro	24/01/2022	4	140	São Domingos	P02	Culicidae	Culicinae	*Mansonia*	*Mansonia sp.*	11
2022	Janeiro	24/01/2022	4	140	São Domingos	P02	Culicidae	Culicinae	*Culex*	*Culex sp.*	3
2022	Janeiro	24/01/2022	4	140	Cujubim Grande	P01	Culicidae	Culicinae	*Culex*	*Culex sp.*	3
2022	Janeiro	31/01/2022	5	141	Rio Contra	P07	Culicidae	Anophelinae	*Anopheles*	*Anopheles darlingi*	1
2022	Janeiro	31/01/2022	5	141	Rio Contra	P07	Culicidae	Culicinae	*Mansonia*	*Mansonia pseudotitillans*	1
2022	Janeiro	31/01/2022	5	141	Rio Contra	P07	Culicidae	Culicinae	*Culex*	*Culex sp.*	2
2022	Janeiro	31/01/2022	5	141	Samauma	P08	Culicidae	Culicinae	*Mansonia*	*Mansonia sp.*	3

2022	Janeiro	31/01/2022	5	141	Samauma	P08	Culicidae	Culicinae	*Culex*	*Culex sp.*	1
2022	Janeiro	31/01/2022	5	141	Jaci Paraná	P06	Culicidae	Culicinae	*Aedes*	*Aedes sp.*	5
2022	Janeiro	31/01/2022	5	141	Jaci Paraná	P06	Culicidae	Culicinae	*Mansonia*	*Mansonia sp.*	3
2022	Janeiro	31/01/2022	5	141	Jaci Paraná	P06	Culicidae	Culicinae	*Culex*	*Culex sp.*	5
2022	Janeiro	31/01/2022	5	141	Santa Rita	P05	Culicidae	Culicinae	*Mansonia*	*Mansonia sp.*	1
2022	Janeiro	31/01/2022	5	141	Morrinhos	P04	Culicidae	Culicinae	*Culex*	*Culex sp.*	10
2022	Janeiro	31/01/2022	5	141	Morrinhos	P04	Culicidae	Culicinae	*Mansonia*	*Mansonia sp.*	5
2022	Janeiro	31/01/2022	5	141	São Domingos	P02	Culicidae	Culicinae	*Coquillettidia*	*Coquillettidia sp.*	3
2022	Janeiro	31/01/2022	5	141	São Domingos	P02	Culicidae	Culicinae	*Culex*	*Culex sp.*	2
2022	Janeiro	31/01/2022	5	141	São Domingos	P02	Culicidae	Culicinae	*Mansonia*	*Mansonia sp.*	7
2022	Fevereiro	07/02/2022	6	142	Rio Contra	P07	Culicidae	Culicinae	*Mansonia*	*Mansonia sp.*	5
2022	Fevereiro	07/02/2022	6	142	Samauma	P08	Culicidae	Culicinae	*Mansonia*	*Mansonia sp.*	5
2022	Fevereiro	07/02/2022	6	142	Samauma	P08	Culicidae	Culicinae	*Culex*	*Culex sp.*	2
2022	Fevereiro	07/02/2022	6	142	Jaci Paraná	P06	Culicidae	Culicinae	*Culex*	*Culex sp.*	10
2022	Fevereiro	07/02/2022	6	142	Jaci Paraná	P06	Culicidae	Culicinae	*Aedes*	*Aedes aegypti*	2
2022	Fevereiro	07/02/2022	6	142	Jaci Paraná	P06	Culicidae	Culicinae	*Mansonia*	*Mansonia sp.*	5
2022	Fevereiro	07/02/2022	6	142	Santa Rita	P05	Culicidae	Culicinae	*Mansonia*	*Mansonia sp.*	5
2022	Fevereiro	07/02/2022	6	142	Morrinhos	P04	Culicidae	Culicinae	*Mansonia*	*Mansonia sp.*	23
2022	Fevereiro	07/02/2022	6	142	Morrinhos	P04	Culicidae	Culicinae	*Culex*	*Culex sp.*	8
2022	Fevereiro	07/02/2022	6	142	São Domingos	P02	Culicidae	Culicinae	*Mansonia*	*Mansonia sp.*	15
2022	Fevereiro	07/02/2022	6	142	São Domingos	P02	Culicidae	Culicinae	*Culex*	*Culex sp.*	39
2022	Fevereiro	07/02/2022	6	142	São Domingos	P02	Culicidae	Culicinae	*Coquillettidia*	*Coquillettidia sp.*	2
2022	Fevereiro	14/02/2022	7	143	Rio Contra	P07	Culicidae	Culicinae	*Mansonia*	*Mansonia sp.*	25
2022	Fevereiro	14/02/2022	7	143	Rio Contra	P07	Culicidae	Anophelinae	*Anopheles*	*Anopheles sp.*	5
2022	Fevereiro	14/02/2022	7	143	Rio Contra	P07	Culicidae	Anophelinae	*Anopheles*	*Anopheles darlingi*	2
2022	Fevereiro	14/02/2022	7	143	Samauma	P08	Culicidae	Culicinae	*Mansonia*	*Mansonia sp.*	18
2022	Fevereiro	14/02/2022	7	143	Samauma	P08	Culicidae	Culicinae	*Culex*	*Culex sp.*	8
2022	Fevereiro	14/02/2022	7	143	Samauma	P08	Culicidae	Culicinae	*Aedeomyia*	*Adeomyia squamipennis*	3
2022	Fevereiro	14/02/2022	7	143	Jaci Paraná	P06	Culicidae	Culicinae	*Aedes*	*Aedes aegypti*	7
2022	Fevereiro	14/02/2022	7	143	Jaci Paraná	P06	Culicidae	Culicinae	*Aedes*	*Aedes sp.*	9
2022	Fevereiro	14/02/2022	7	143	Jaci Paraná	P06	Culicidae	Culicinae	*Culex*	*Culex sp.*	11
2022	Fevereiro	14/02/2022	7	143	Jaci Paraná	P06	Culicidae	Culicinae	*Mansonia*	*Mansonia sp.*	5
2022	Fevereiro	14/02/2022	7	143	Santa Rita	P05	Culicidae	Culicinae	*Mansonia*	*Mansonia sp.*	12
2022	Fevereiro	14/02/2022	7	143	Santa Rita	P05	Culicidae	Culicinae	*Culex*	*Culex sp.*	7
2022	Fevereiro	14/02/2022	7	143	Santa Rita	P05	Culicidae	Culicinae	*Aedes*	*Aedes sp.*	2
2022	Fevereiro	14/02/2022	7	143	Morrinhos	P04	Culicidae	Culicinae	*Mansonia*	*Mansonia sp.*	43
2022	Fevereiro	14/02/2022	7	143	Morrinhos	P04	Culicidae	Culicinae	*Culex*	*Culex sp.*	11
2022	Fevereiro	14/02/2022	7	143	Teotônio	P03	Culicidae	Culicinae	*Mansonia*	*Mansonia sp.*	2
2022	Fevereiro	14/02/2022	7	143	Teotônio	P03	Culicidae	Culicinae	*Coquillettidia*	*Coquillettidia sp.*	5
2022	Fevereiro	14/02/2022	7	143	São Domingos	P02	Culicidae	Culicinae	*Culex*	*Culex sp.*	33
2022	Fevereiro	14/02/2022	7	143	São Domingos	P02	Culicidae	Culicinae	*Mansonia*	*Mansonia sp.*	12
2022	Fevereiro	14/02/2022	7	143	São Domingos	P02	Culicidae	Anophelinae	*Anopheles*	*Anopheles sp.*	1
2022	Fevereiro	14/02/2022	7	143	São Domingos	P02	Culicidae	Culicinae	*Coquillettidia*	*Coquillettidia sp.*	1
2022	Fevereiro	21/02/2022	8	144	Rio Contra	P07	Culicidae	Culicinae	*Mansonia*	*Mansonia sp.*	5
2022	Fevereiro	21/02/2022	8	144	Rio Contra	P07	Culicidae	Culicinae	*Culex*	*Culex sp.*	1
2022	Fevereiro	21/02/2022	8	144	Rio Contra	P07	Culicidae	Anophelinae	*Anopheles*	*Anopheles sp.*	1
2022	Fevereiro	21/02/2022	8	144	Samauma	P08	Culicidae	Culicinae	*Mansonia*	*Mansonia sp.*	16
2022	Fevereiro	21/02/2022	8	144	Samauma	P08	Culicidae	Culicinae	*Culex*	*Culex sp.*	3
2022	Fevereiro	21/02/2022	8	144	Jaci Paraná	P06	Culicidae	Culicinae	*Aedes*	*Aedes sp.*	2
2022	Fevereiro	21/02/2022	8	144	Jaci Paraná	P06	Culicidae	Culicinae	*Aedes*	*Aedes aegypti*	3
2022	Fevereiro	21/02/2022	8	144	Jaci Paraná	P06	Culicidae	Culicinae	*Culex*	*Culex sp.*	21
2022	Fevereiro	21/02/2022	8	144	Jaci Paraná	P06	Culicidae	Culicinae	*Mansonia*	*Mansonia sp.*	4
2022	Fevereiro	21/02/2022	8	144	Santa Rita	P05	Culicidae	Culicinae	*Mansonia*	*Mansonia sp.*	5
2022	Fevereiro	21/02/2022	8	144	Morrinhos	P04	Culicidae	Culicinae	*Mansonia*	*Mansonia sp.*	8
2022	Fevereiro	21/02/2022	8	144	Morrinhos	P04	Culicidae	Culicinae	*Culex*	*Culex sp.*	5
2022	Fevereiro	21/02/2022	8	144	Teotônio	P03	Culicidae	Culicinae	*Coquillettidia*	*Coquillettidia sp.*	2
2022	Fevereiro	21/02/2022	8	144	Teotônio	P03	Culicidae	Culicinae	*Culex*	*Culex sp.*	3
2022	Fevereiro	21/02/2022	8	144	Teotônio	P03	Culicidae	Culicinae	*Mansonia*	*Mansonia sp.*	1
2022	Fevereiro	21/02/2022	8	144	São Domingos	P02	Culicidae	Culicinae	*Mansonia*	*Mansonia sp.*	35
2022	Fevereiro	21/02/2022	8	144	São Domingos	P02	Culicidae	Culicinae	*Culex*	*Culex sp.*	42
2022	Fevereiro	21/02/2022	8	144	São Domingos	P02	Culicidae	Anophelinae	*Anopheles*	*Anopheles sp.*	2
2022	Fevereiro	21/02/2022	8	144	São Domingos	P02	Culicidae	Culicinae	*Coquillettidia*	*Coquillettidia sp.*	2
2022	Fevereiro	21/02/2022	8	144	Cujubim Grande	P01	Culicidae	Culicinae	*Culex*	*Culex sp.*	10
2022	Março	28/02/2022	9	145	Rio Contra	P07	Culicidae	Culicinae	*Mansonia*	*Mansonia sp.*	11
2022	Março	28/02/2022	9	145	Rio Contra	P07	Culicidae	Culicinae	*Culex*	*Culex sp.*	1
2022	Março	28/02/2022	9	145	Samauma	P08	Culicidae	Culicinae	*Mansonia*	*Mansonia sp.*	7
2022	Março	28/02/2022	9	145	Jaci Paraná	P06	Culicidae	Culicinae	*Culex*	*Culex sp.*	5
2022	Março	28/02/2022	9	145	Jaci Paraná	P06	Culicidae	Culicinae	*Aedes*	*Aedes sp.*	3
2022	Março	28/02/2022	9	145	Jaci Paraná	P06	Culicidae	Culicinae	*Mansonia*	*Mansonia sp.*	10
2022	Março	28/02/2022	9	145	Santa Rita	P05	Culicidae	Culicinae	*Mansonia*	*Mansonia sp.*	3
2022	Março	28/02/2022	9	145	Santa Rita	P05	Culicidae	Culicinae	*Aedes*	*Aedes sp.*	1
2022	Março	28/02/2022	9	145	Santa Rita	P05	Culicidae	Culicinae	*Culex*	*Culex sp.*	2
2022	Março	28/02/2022	9	145	Morrinhos	P04	Culicidae	Culicinae	*Mansonia*	*Mansonia sp.*	23
2022	Março	28/02/2022	9	145	Morrinhos	P04	Culicidae	Culicinae	*Culex*	*Culex sp.*	1
2022	Março	28/02/2022	9	145	Teotônio	P03	Culicidae	Culicinae	*Mansonia*	*Mansonia sp.*	2
2022	Março	28/02/2022	9	145	São Domingos	P02	Culicidae	Culicinae	*Culex*	*Culex sp.*	15
2022	Março	28/02/2022	9	145	São Domingos	P02	Culicidae	Culicinae	*Coquillettidia*	*Coquillettidia sp.*	1
2022	Março	28/02/2022	9	145	São Domingos	P02	Culicidae	Culicinae	*Mansonia*	*Mansonia sp.*	6
2022	Março	07/03/2022	10	146	Rio Contra	P07	Culicidae	Anophelinae	*Anopheles*	*Anopheles sp.*	2
2022	Março	07/03/2022	10	146	Rio Contra	P07	Culicidae	Culicinae	*Mansonia*	*Mansonia sp.*	6
2022	Março	07/03/2022	10	146	Samauma	P08	Culicidae	Culicinae	*Mansonia*	*Mansonia sp.*	25
2022	Março	07/03/2022	10	146	Samauma	P08	Culicidae	Culicinae	*Culex*	*Culex sp.*	4
2022	Março	07/03/2022	10	146	Samauma	P08	Culicidae	Culicinae	*Aedeomyia*	*Adeomyia squamipennis*	2
2022	Março	07/03/2022	10	146	Jaci Paraná	P06	Culicidae	Culicinae	*Culex*	*Culex sp.*	8
2022	Março	07/03/2022	10	146	Jaci Paraná	P06	Culicidae	Culicinae	*Mansonia*	*Mansonia sp.*	5
2022	Março	07/03/2022	10	146	Jaci Paraná	P06	Culicidae	Culicinae	*Aedes*	*Aedes sp.*	2
2022	Março	07/03/2022	10	146	Santa Rita	P05	Culicidae	Culicinae	*Culex*	*Culex sp.*	20
2022	Março	07/03/2022	10	146	Santa Rita	P05	Culicidae	Culicinae	*Mansonia*	*Mansonia sp.*	9
2022	Março	07/03/2022	10	146	Morrinhos	P04	Culicidae	Culicinae	*Mansonia*	*Mansonia sp.*	12
2022	Março	07/03/2022	10	146	Morrinhos	P04	Culicidae	Culicinae	*Culex*	*Culex sp.*	14
2022	Março	07/03/2022	10	146	Teotônio	P03	Culicidae	Culicinae	*Coquillettidia*	*Coquillettidia sp.*	3
2022	Março	07/03/2022	10	146	Teotônio	P03	Culicidae	Culicinae	*Mansonia*	*Mansonia sp.*	2
2022	Março	07/03/2022	10	146	Teotônio	P03	Culicidae	Culicinae	*Culex*	*Culex sp.*	5
2022	Março	07/03/2022	10	146	São Domingos	P02	Culicidae	Culicinae	*Culex*	*Culex sp.*	22
2022	Março	07/03/2022	10	146	São Domingos	P02	Culicidae	Culicinae	*Mansonia*	*Mansonia sp.*	15
2022	Março	07/03/2022	10	146	Cujubim Grande	P01	Culicidae	Culicinae	*Culex*	*Culex sp.*	1
2022	Março	07/03/2022	10	146	Cujubim Grande	P01	Culicidae	Culicinae	*Mansonia*	*Mansonia sp.*	3
2022	Março	14/03/2022	11	147	Rio Contra	P07	Culicidae	Anophelinae	*Anopheles*	*Anopheles sp.*	2
2022	Março	14/03/2022	11	147	Rio Contra	P07	Culicidae	Culicinae	*Mansonia*	*Mansonia sp.*	3
2022	Março	14/03/2022	11	147	Rio Contra	P07	Culicidae	Culicinae	*Culex*	*Culex sp.*	1
2022	Março	14/03/2022	11	147	Samauma	P08	Culicidae	Culicinae	*Culex*	*Culex sp.*	3
2022	Março	14/03/2022	11	147	Samauma	P08	Culicidae	Culicinae	*Mansonia*	*Mansonia sp.*	1
2022	Março	14/03/2022	11	147	Samauma	P08	Culicidae	Culicinae	*Aedeomyia*	*Adeomyia squamipennis*	1
2022	Março	14/03/2022	11	147	Jaci Paraná	P06	Culicidae	Culicinae	*Aedes*	*Aedes aegypti*	2
2022	Março	14/03/2022	11	147	Jaci Paraná	P06	Culicidae	Culicinae	*Culex*	*Culex sp.*	6
2022	Março	14/03/2022	11	147	Jaci Paraná	P06	Culicidae	Culicinae	*Mansonia*	*Mansonia sp.*	1
2022	Março	14/03/2022	11	147	Santa Rita	P05	Culicidae	Culicinae	*Mansonia*	*Mansonia sp.*	3
2022	Março	14/03/2022	11	147	Santa Rita	P05	Culicidae	Culicinae	*Culex*	*Culex sp.*	8
2022	Março	14/03/2022	11	147	Morrinhos	P04	Culicidae	Culicinae	*Mansonia*	*Mansonia sp.*	22
2022	Março	14/03/2022	11	147	Morrinhos	P04	Culicidae	Culicinae	*Culex*	*Culex sp.*	7
2022	Março	14/03/2022	11	147	Teotônio	P03	Culicidae	Culicinae	*Coquillettidia*	*Coquillettidia sp.*	3
2022	Março	14/03/2022	11	147	Teotônio	P03	Culicidae	Culicinae	*Mansonia*	*Mansonia sp.*	3
2022	Março	14/03/2022	11	147	Teotônio	P03	Culicidae	Culicinae	*Culex*	*Culex sp.*	2
2022	Março	14/03/2022	11	147	São Domingos	P02	Culicidae	Culicinae	*Culex*	*Culex sp.*	25
2022	Março	14/03/2022	11	147	São Domingos	P02	Culicidae	Culicinae	*Mansonia*	*Mansonia sp.*	12
2022	Março	14/03/2022	11	147	Cujubim Grande	P01	Culicidae	Culicinae	*Culex*	*Culex sp.*	5
2022	Março	21/03/2022	12	148	Rio Contra	P07	Culicidae	Anophelinae	*Anopheles*	*Anopheles darlingi*	5
2022	Março	21/03/2022	12	148	Rio Contra	P07	Culicidae	Culicinae	*Mansonia*	*Mansonia sp.*	22

2022	Março	21/03/2022	12	148	Rio Contra	P07	Culicidae	Culicinae	*Culex*	*Culex sp.*	5
2022	Março	21/03/2022	12	148	Samauma	P08	Culicidae	Culicinae	*Mansonia*	*Mansonia sp.*	5
2022	Março	21/03/2022	12	148	Jaci Paraná	P06	Culicidae	Culicinae	*Culex*	*Culex sp.*	10
2022	Março	21/03/2022	12	148	Jaci Paraná	P06	Culicidae	Culicinae	*Mansonia*	*Mansonia sp.*	8
2022	Março	21/03/2022	12	148	Jaci Paraná	P06	Culicidae	Culicinae	*Aedes*	*Aedes sp.*	3
2022	Março	21/03/2022	12	148	Jaci Paraná	P06	Culicidae	Culicinae	*Aedes*	*Aedes aegypti*	3
2022	Março	21/03/2022	12	148	Santa Rita	P05	Culicidae	Culicinae	*Culex*	*Culex sp.*	33
2022	Março	21/03/2022	12	148	Santa Rita	P05	Culicidae	Culicinae	*Mansonia*	*Mansonia sp.*	18
2022	Março	21/03/2022	12	148	Morrinhos	P04	Culicidae	Culicinae	*Mansonia*	*Mansonia sp.*	13
2022	Março	21/03/2022	12	148	Morrinhos	P04	Culicidae	Culicinae	*Culex*	*Culex sp.*	15
2022	Março	21/03/2022	12	148	Teotônio	P03	Culicidae	Culicinae	*Coquillettidia*	*Coquillettidia sp.*	2
2022	Março	21/03/2022	12	148	Teotônio	P03	Culicidae	Culicinae	*Culex*	*Culex sp.*	3
2022	Março	21/03/2022	12	148	Teotônio	P03	Culicidae	Culicinae	*Mansonia*	*Mansonia sp.*	5
2022	Março	21/03/2022	12	148	São Domingos	P02	Culicidae	Culicinae	*Mansonia*	*Mansonia humeralis*	32
2022	Março	21/03/2022	12	148	São Domingos	P02	Culicidae	Culicinae	*Culex*	*Culex sp.*	25
2022	Março	21/03/2022	12	148	São Domingos	P02	Culicidae	Culicinae	*Coquillettidia*	*Coquillettidia sp.*	4
2022	Março	21/03/2022	12	148	Cujubim Grande	P01	Culicidae	Culicinae	*Mansonia*	*Mansonia sp.*	3
2022	Março	21/03/2022	12	148	Cujubim Grande	P01	Culicidae	Culicinae	*Culex*	*Culex sp.*	8
2022	Março	28/03/2022	13	149	Rio Contra	P07	Culicidae	Anophelinae	*Anopheles*	*Anopheles darlingi*	3
2022	Março	28/03/2022	13	149	Rio Contra	P07	Culicidae	Culicinae	*Culex*	*Culex sp.*	5
2022	Março	28/03/2022	13	149	Rio Contra	P07	Culicidae	Culicinae	*Mansonia*	*Mansonia sp.*	1
2022	Março	28/03/2022	13	149	Samauma	P08	Culicidae	Culicinae	*Mansonia*	*Mansonia sp.*	5
2022	Março	28/03/2022	13	149	Jaci Paraná	P06	Culicidae	Culicinae	*Aedes*	*Aedes aegypti*	5
2022	Março	28/03/2022	13	149	Jaci Paraná	P06	Culicidae	Culicinae	*Culex*	*Culex sp.*	3
2022	Março	28/03/2022	13	149	Jaci Paraná	P06	Culicidae	Culicinae	*Mansonia*	*Mansonia sp.*	2
2022	Março	28/03/2022	13	149	Santa Rita	P05	Culicidae	Culicinae	*Mansonia*	*Mansonia sp.*	5
2022	Março	28/03/2022	13	149	Santa Rita	P05	Culicidae	Culicinae	*Culex*	*Culex sp.*	9
2022	Março	28/03/2022	13	149	Santa Rita	P05	Culicidae	Culicinae	*Aedeomyia*	*Adeomyia squamipennis*	2
2022	Março	28/03/2022	13	149	Morrinhos	P04	Culicidae	Culicinae	*Mansonia*	*Mansonia sp.*	17
2022	Março	28/03/2022	13	149	Morrinhos	P04	Culicidae	Culicinae	*Culex*	*Culex sp.*	6
2022	Março	28/03/2022	13	149	Teotônio	P03	Culicidae	Culicinae	*Culex*	*Culex sp.*	2
2022	Março	28/03/2022	13	149	Teotônio	P03	Culicidae	Culicinae	*Coquillettidia*	*Coquillettidia sp.*	3
2022	Março	28/03/2022	13	149	Teotônio	P03	Culicidae	Culicinae	*Mansonia*	*Mansonia sp.*	1
2022	Março	28/03/2022	13	149	São Domingos	P02	Culicidae	Culicinae	*Culex*	*Culex sp.*	15
2022	Março	28/03/2022	13	149	São Domingos	P02	Culicidae	Culicinae	*Mansonia*	*Mansonia sp.*	11
2022	Março	28/03/2022	13	149	Cujubim Grande	P01	Culicidae	Culicinae	*Mansonia*	*Mansonia sp.*	5
2022	Março	28/03/2022	13	149	Cujubim Grande	P01	Culicidae	Culicinae	*Culex*	*Culex sp.*	5
2022	Abril	04/04/2022	14	150	Rio Contra	P07	Culicidae	Anophelinae	*Anopheles*	*Anopheles darlingi*	2
2022	Abril	04/04/2022	14	150	Rio Contra	P07	Culicidae	Culicinae	*Mansonia*	*Mansonia indubitans*	1
2022	Abril	04/04/2022	14	150	Samauma	P08	Culicidae	Culicinae	*Mansonia*	*Mansonia sp.*	2
2022	Abril	04/04/2022	14	150	Samauma	P08	Culicidae	Culicinae	*Culex*	*Culex sp.*	3
2022	Abril	04/04/2022	14	150	Jaci Paraná	P06	Culicidae	Culicinae	*Aedes*	*Aedes aegypti*	1
2022	Abril	04/04/2022	14	150	Jaci Paraná	P06	Culicidae	Culicinae	*Mansonia*	*Mansonia sp.*	2
2022	Abril	04/04/2022	14	150	Jaci Paraná	P06	Culicidae	Culicinae	*Culex*	*Culex sp.*	5
2022	Abril	04/04/2022	14	150	Santa Rita	P05	Culicidae	Culicinae	*Mansonia*	*Mansonia sp.*	33
2022	Abril	04/04/2022	14	150	Santa Rita	P05	Culicidae	Culicinae	*Culex*	*Culex sp.*	12
2022	Abril	04/04/2022	14	150	Santa Rita	P05	Culicidae	Culicinae	*Aedeomyia*	*Adeomyia squamipennis*	5
2022	Abril	04/04/2022	14	150	Morrinhos	P04	Culicidae	Culicinae	*Mansonia*	*Mansonia sp.*	6
2022	Abril	04/04/2022	14	150	Teotônio	P03	Culicidae	Culicinae	*Coquillettidia*	*Coquillettidia sp.*	5
2022	Abril	04/04/2022	14	150	Teotônio	P03	Culicidae	Culicinae	*Mansonia*	*Mansonia sp.*	4
2022	Abril	04/04/2022	14	150	Teotônio	P03	Culicidae	Culicinae	*Aedeomyia*	*Adeomyia squamipennis*	1
2022	Abril	04/04/2022	14	150	São Domingos	P02	Culicidae	Culicinae	*Mansonia*	*Mansonia sp.*	53
2022	Abril	04/04/2022	14	150	São Domingos	P02	Culicidae	Culicinae	*Culex*	*Culex sp.*	21
2022	Abril	04/04/2022	14	150	São Domingos	P02	Culicidae	Culicinae	*Coquillettidia*	*Coquillettidia sp.*	5
2022	Abril	04/04/2022	14	150	Cujubim Grande	P01	Culicidae	Culicinae	*Mansonia*	*Mansonia sp.*	5
2022	Abril	04/04/2022	14	150	Cujubim Grande	P01	Culicidae	Culicinae	*Culex*	*Culex sp.*	12
2022	Abril	11/04/2022	15	151	Rio Contra	P07	Culicidae	Culicinae	*Mansonia*	*Mansonia sp.*	12
2022	Abril	11/04/2022	15	151	Rio Contra	P07	Culicidae	Culicinae	*Culex*	*Culex sp.*	3
2022	Abril	11/04/2022	15	151	Rio Contra	P07	Culicidae	Anophelinae	*Anopheles*	*Anopheles darlingi*	1
2022	Abril	11/04/2022	15	151	Samauma	P08	Culicidae	Culicinae	*Mansonia*	*Mansonia sp.*	112
2022	Abril	11/04/2022	15	151	Samauma	P08	Culicidae	Culicinae	*Culex*	*Culex sp.*	25
2022	Abril	11/04/2022	15	151	Samauma	P08	Culicidae	Culicinae	*Aedeomyia*	*Adeomyia squamipennis*	7
2022	Abril	11/04/2022	15	151	Jaci Paraná	P06	Culicidae	Culicinae	*Aedes*	*Aedes aegypti*	1
2022	Abril	11/04/2022	15	151	Jaci Paraná	P06	Culicidae	Culicinae	*Mansonia*	*Mansonia sp.*	1
2022	Abril	11/04/2022	15	151	Jaci Paraná	P06	Culicidae	Culicinae	*Culex*	*Culex sp.*	2
2022	Abril	11/04/2022	15	151	Santa Rita	P05	Culicidae	Culicinae	*Mansonia*	*Mansonia sp.*	3
2022	Abril	11/04/2022	15	151	Santa Rita	P05	Culicidae	Culicinae	*Culex*	*Culex sp.*	5
2022	Abril	11/04/2022	15	151	Morrinhos	P04	Culicidae	Culicinae	*Mansonia*	*Mansonia humeralis*	7
2022	Abril	11/04/2022	15	151	Morrinhos	P04	Culicidae	Culicinae	*Culex*	*Culex sp.*	4
2022	Abril	11/04/2022	15	151	Teotônio	P03	Culicidae	Culicinae	*Mansonia*	*Mansonia sp.*	3
2022	Abril	11/04/2022	15	151	Teotônio	P03	Culicidae	Culicinae	*Coquillettidia*	*Coquillettidia sp.*	2
2022	Abril	11/04/2022	15	151	Teotônio	P03	Culicidae	Culicinae	*Culex*	*Culex sp.*	5
2022	Abril	11/04/2022	15	151	São Domingos	P02	Culicidae	Anophelinae	*Anopheles*	*Anopheles sp.*	1
2022	Abril	11/04/2022	15	151	São Domingos	P02	Culicidae	Culicinae	*Mansonia*	*Mansonia sp.*	11
2022	Abril	11/04/2022	15	151	São Domingos	P02	Culicidae	Culicinae	*Culex*	*Culex sp.*	2
2022	Abril	11/04/2022	15	151	São Domingos	P02	Culicidae	Culicinae	*Coquillettidia*	*Coquillettidia sp.*	2
2022	Abril	11/04/2022	15	151	Cujubim Grande	P01	Culicidae	Culicinae	*Culex*	*Culex sp.*	3
2022	Abril	11/04/2022	15	151	Cujubim Grande	P01	Culicidae	Culicinae	*Mansonia*	*Mansonia sp.*	1
2022	Abril	18/04/2022	16	152	Rio Contra	P07	Culicidae	Culicinae	*Mansonia*	*Mansonia sp.*	2
2022	Abril	18/04/2022	16	152	Rio Contra	P07	Culicidae	Culicinae	*Culex*	*Culex sp.*	14
2022	Abril	18/04/2022	16	152	Rio Contra	P07	Culicidae	Anophelinae	*Anopheles*	*Anopheles sp.*	5
2022	Abril	18/04/2022	16	152	Samauma	P08	Culicidae	Culicinae	*Mansonia*	*Mansonia sp.*	1233
2022	Abril	18/04/2022	16	152	Samauma	P08	Culicidae	Culicinae	*Culex*	*Culex sp.*	221
2022	Abril	18/04/2022	16	152	Samauma	P08	Culicidae	Culicinae	*Aedeomyia*	*Adeomyia squamipennis*	2
2022	Abril	18/04/2022	16	152	Jaci Paraná	P06	Culicidae	Culicinae	*Culex*	*Culex sp.*	335
2022	Abril	18/04/2022	16	152	Jaci Paraná	P06	Culicidae	Culicinae	*Mansonia*	*Mansonia sp.*	102
2022	Abril	18/04/2022	16	152	Jaci Paraná	P06	Culicidae	Culicinae	*Aedes*	*Aedes aegypti*	23
2022	Abril	18/04/2022	16	152	Santa Rita	P05	Culicidae	Culicinae	*Mansonia*	*Mansonia sp.*	53
2022	Abril	18/04/2022	16	152	Santa Rita	P05	Culicidae	Culicinae	*Culex*	*Culex sp.*	12
2022	Abril	18/04/2022	16	152	Santa Rita	P05	Culicidae	Culicinae	*Aedes*	*Aedes sp.*	2
2022	Abril	18/04/2022	16	152	Morrinhos	P04	Culicidae	Culicinae	*Mansonia*	*Mansonia sp.*	75
2022	Abril	18/04/2022	16	152	Morrinhos	P04	Culicidae	Culicinae	*Culex*	*Culex sp.*	28
2022	Abril	18/04/2022	16	152	Morrinhos	P04	Culicidae	Culicinae	*Aedes*	*Aedes sp.*	1
2022	Abril	18/04/2022	16	152	Morrinhos	P04	Culicidae	Culicinae	*Aedeomyia*	*Adeomyia squamipennis*	1
2022	Abril	18/04/2022	16	152	Teotônio	P03	Culicidae	Culicinae	*Coquillettidia*	*Coquillettidia sp.*	6
2022	Abril	18/04/2022	16	152	Teotônio	P03	Culicidae	Culicinae	*Mansonia*	*Mansonia amazonensis*	12
2022	Abril	18/04/2022	16	152	Teotônio	P03	Culicidae	Culicinae	*Culex*	*Culex sp.*	5
2022	Abril	18/04/2022	16	152	São Domingos	P02	Culicidae	Culicinae	*Mansonia*	*Mansonia sp.*	35
2022	Abril	18/04/2022	16	152	São Domingos	P02	Culicidae	Culicinae	*Culex*	*Culex sp.*	28
2022	Abril	18/04/2022	16	152	São Domingos	P02	Culicidae	Culicinae	*Coquillettidia*	*Coquillettidia sp.*	2
2022	Abril	18/04/2022	16	152	São Domingos	P02	Culicidae	Culicinae	*Aedeomyia*	*Adeomyia squamipennis*	3
2022	Abril	18/04/2022	16	152	Cujubim Grande	P01	Culicidae	Culicinae	*Mansonia*	*Mansonia sp.*	10
2022	Abril	18/04/2022	16	152	Cujubim Grande	P01	Culicidae	Culicinae	*Culex*	*Culex sp.*	18
2022	Abril	25/04/2022	17	153	Rio Contra	P07	Culicidae	Anophelinae	*Anopheles*	*Anopheles sp.*	2
2022	Abril	25/04/2022	17	153	Rio Contra	P07	Culicidae	Culicinae	*Mansonia*	*Mansonia sp.*	3
2022	Abril	25/04/2022	17	153	Samauma	P08	Culicidae	Culicinae	*Mansonia*	*Mansonia sp.*	378
2022	Abril	25/04/2022	17	153	Samauma	P08	Culicidae	Culicinae	*Culex*	*Culex sp.*	63
2022	Abril	25/04/2022	17	153	Samauma	P08	Culicidae	Culicinae	*Aedeomyia*	*Adeomyia squamipennis*	5
2022	Abril	25/04/2022	17	153	Jaci Paraná	P06	Culicidae	Culicinae	*Mansonia*	*Mansonia sp.*	18
2022	Abril	25/04/2022	17	153	Jaci Paraná	P06	Culicidae	Culicinae	*Culex*	*Culex sp.*	5
2022	Abril	25/04/2022	17	153	Jaci Paraná	P06	Culicidae	Culicinae	*Aedes*	*Aedes aegypti*	5
2022	Abril	25/04/2022	17	153	Santa Rita	P05	Culicidae	Culicinae	*Mansonia*	*Mansonia humeralis*	8
2022	Abril	25/04/2022	17	153	Santa Rita	P05	Culicidae	Culicinae	*Culex*	*Culex sp.*	5
2022	Abril	25/04/2022	17	153	Morrinhos	P04	Culicidae	Culicinae	*Mansonia*	*Mansonia sp.*	25
2022	Abril	25/04/2022	17	153	Morrinhos	P04	Culicidae	Culicinae	*Culex*	*Culex sp.*	12
2022	Abril	25/04/2022	17	153	Teotônio	P03	Culicidae	Culicinae	*Mansonia*	*Mansonia sp.*	5
2022	Abril	25/04/2022	17	153	Teotônio	P03	Culicidae	Culicinae	*Culex*	*Culex sp.*	2
2022	Abril	25/04/2022	17	153	Teotônio	P03	Culicidae	Culicinae	*Coquillettidia*	*Coquillettidia sp.*	4

2022	Abril	25/04/2022	17	153	São Domingos	P02	Culicidae	Culicinae	*Mansonia*	*Mansonia sp.*	33
2022	Abril	25/04/2022	17	153	São Domingos	P02	Culicidae	Culicinae	*Culex*	*Culex sp.*	12
2022	Abril	25/04/2022	17	153	São Domingos	P02	Culicidae	Culicinae	*Coquillettidia*	*Coquillettidia sp.*	7
2022	Abril	25/04/2022	17	153	Cujubim Grande	P01	Culicidae	Culicinae	*Culex*	*Culex sp.*	12
2022	Maio	02/05/2022	18	154	Samauma	P08	Culicidae	Culicinae	*Mansonia*	*Mansonia sp.*	118
2022	Maio	02/05/2022	18	154	Samauma	P08	Culicidae	Culicinae	*Culex*	*Culex sp.*	26
2022	Maio	02/05/2022	18	154	Samauma	P08	Culicidae	Culicinae	*Aedeomyia*	*Adeomyia squamipennis*	2
2022	Maio	02/05/2022	18	154	Santa Rita	P05	Culicidae	Culicinae	*Culex*	*Culex sp.*	37
2022	Maio	02/05/2022	18	154	Santa Rita	P05	Culicidae	Culicinae	*Mansonia*	*Mansonia sp.*	21
2022	Maio	02/05/2022	18	154	Morrinhos	P04	Culicidae	Culicinae	*Culex*	*Culex sp.*	10
2022	Maio	02/05/2022	18	154	Morrinhos	P04	Culicidae	Culicinae	*Mansonia*	*Mansonia sp.*	8
2022	Maio	02/05/2022	18	154	Teotônio	P03	Culicidae	Culicinae	*Culex*	*Culex sp.*	3
2022	Maio	02/05/2022	18	154	Teotônio	P03	Culicidae	Culicinae	*Mansonia*	*Mansonia sp.*	1
2022	Maio	02/05/2022	18	154	Teotônio	P03	Culicidae	Culicinae	*Coquillettidia*	*Coquillettidia sp.*	1
2022	Maio	02/05/2022	18	154	São Domingos	P02	Culicidae	Culicinae	*Mansonia*	*Mansonia sp.*	21
2022	Maio	02/05/2022	18	154	São Domingos	P02	Culicidae	Culicinae	*Culex*	*Culex sp.*	11
2022	Maio	02/05/2022	18	154	São Domingos	P02	Culicidae	Culicinae	*Aedes*	*Aedes sp.*	3
2022	Maio	02/05/2022	18	154	São Domingos	P02	Culicidae	Culicinae	*Coquillettidia*	*Coquillettidia sp.*	1
2022	Maio	02/05/2022	18	154	São Domingos	P02	Culicidae	Culicinae	*Limatus*	*Limatus durhamii*	1
2022	Maio	02/05/2022	18	154	Cujubim Grande	P01	Culicidae	Culicinae	*Culex*	*Culex sp.*	10
2022	Maio	02/05/2022	18	154	Cujubim Grande	P01	Culicidae	Culicinae	*Mansonia*	*Mansonia sp.*	8
2022	Maio	10/05/2022	19	155	Samauma	P08	Culicidae	Culicinae	*Mansonia*	*Mansonia sp.*	254
2022	Maio	10/05/2022	19	155	Samauma	P08	Culicidae	Culicinae	*Culex*	*Culex sp.*	9
2022	Maio	10/05/2022	19	155	Samauma	P08	Culicidae	Culicinae	*Aedeomyia*	*Adeomyia squamipennis*	1
2022	Maio	10/05/2022	19	155	Santa Rita	P05	Culicidae	Culicinae	*Mansonia*	*Mansonia sp.*	28
2022	Maio	10/05/2022	19	155	Santa Rita	P05	Culicidae	Culicinae	*Culex*	*Culex sp.*	13
2022	Maio	10/05/2022	19	155	Santa Rita	P05	Culicidae	Culicinae	*Aedes*	*Aedes sp.*	5
2022	Maio	10/05/2022	19	155	Morrinhos	P04	Culicidae	Culicinae	*Mansonia*	*Mansonia sp.*	57
2022	Maio	10/05/2022	19	155	Morrinhos	P04	Culicidae	Culicinae	*Culex*	*Culex sp.*	8
2022	Maio	10/05/2022	19	155	Morrinhos	P04	Culicidae	Culicinae	*Limatus*	*Limatus durhamii*	3
2022	Maio	10/05/2022	19	155	Teotônio	P03	Culicidae	Culicinae	*Coquillettidia*	*Coquillettidia sp.*	5
2022	Maio	10/05/2022	19	155	Teotônio	P03	Culicidae	Culicinae	*Mansonia*	*Mansonia sp.*	2
2022	Maio	10/05/2022	19	155	São Domingos	P02	Culicidae	Culicinae	*Mansonia*	*Mansonia sp.*	26
2022	Maio	10/05/2022	19	155	São Domingos	P02	Culicidae	Culicinae	*Culex*	*Culex sp.*	2
2022	Maio	10/05/2022	19	155	São Domingos	P02	Culicidae	Culicinae	*Coquillettidia*	*Coquillettidia sp.*	2
2022	Maio	10/05/2022	19	155	São Domingos	P02	Culicidae	Culicinae	*Aedeomyia*	*Adeomyia squamipennis*	1
2022	Maio	10/05/2022	19	155	Cujubim Grande	P01	Culicidae	Culicinae	*Mansonia*	*Mansonia sp.*	7
2022	Maio	10/05/2022	19	155	Cujubim Grande	P01	Culicidae	Culicinae	*Culex*	*Culex sp.*	5
2022	Maio	30/05/2022	22	159	Samauma	P08	Culicidae	Culicinae	*Mansonia*	*Mansonia sp.*	85
2022	Maio	30/05/2022	22	159	Samauma	P08	Culicidae	Culicinae	*Culex*	*Culex sp.*	12
2022	Maio	30/05/2022	22	159	Samauma	P08	Culicidae	Culicinae	*Aedes*	*Aedes sp.*	3
2022	Maio	30/05/2022	22	159	Santa Rita	P05	Culicidae	Culicinae	*Aedeomyia*	*Adeomyia squamipennis*	3
2022	Maio	30/05/2022	22	159	Santa Rita	P05	Culicidae	Culicinae	*Culex*	*Culex sp.*	42
2022	Maio	30/05/2022	22	159	Morrinhos	P04	Culicidae	Culicinae	*Mansonia*	*Mansonia sp.*	37
2022	Maio	30/05/2022	22	159	Teotônio	P03	Culicidae	Culicinae	*Mansonia*	*Mansonia sp.*	1
2022	Maio	30/05/2022	22	159	Teotônio	P03	Culicidae	Anophelinae	*Anopheles*	*Anopheles sp.*	2
2022	Maio	30/05/2022	22	159	Teotônio	P03	Culicidae	Culicinae	*Culex*	*Culex sp.*	3
2022	Maio	30/05/2022	22	159	Teotônio	P03	Culicidae	Culicinae	*Coquillettidia*	*Coquillettidia sp.*	12
2022	Maio	30/05/2022	22	159	São Domingos	P02	Culicidae	Culicinae	*Mansonia*	*Mansonia sp.*	75
2022	Maio	30/05/2022	22	159	São Domingos	P02	Culicidae	Culicinae	*Mansonia*	*Mansonia sp.*	26
2022	Maio	30/05/2022	22	159	São Domingos	P02	Culicidae	Culicinae	*Coquillettidia*	*Coquillettidia sp.*	12
2022	Junho	06/06/2022	23	159	Samauma	P08	Culicidae	Culicinae	*Mansonia*	*Mansonia sp.*	107
2022	Junho	06/06/2022	23	159	Samauma	P08	Culicidae	Culicinae	*Culex*	*Culex sp.*	15
2022	Junho	06/06/2022	23	159	Samauma	P08	Culicidae	Culicinae	*Coquillettidia*	*Coquillettidia sp.*	4
2022	Junho	06/06/2022	23	159	Samauma	P08	Culicidae	Culicinae	*Aedeomyia*	*Adeomyia squamipennis*	2
2022	Junho	06/06/2022	23	159	Morrinhos	P04	Culicidae	Culicinae	*Mansonia*	*Mansonia sp.*	37
2022	Junho	06/06/2022	23	159	Teotônio	P03	Culicidae	Culicinae	*Mansonia*	*Mansonia sp.*	16
2022	Junho	06/06/2022	23	159	São Domingos	P02	Culicidae	Culicinae	*Mansonia*	*Mansonia sp.*	88
2022	Junho	06/06/2022	23	159	São Domingos	P02	Culicidae	Culicinae	*Culex*	*Culex sp.*	25
2022	Junho	06/06/2022	23	159	Cujubim Grande	P01	Culicidae	Culicinae	*Mansonia*	*Mansonia sp.*	5
2022	Junho	06/06/2022	23	159	Cujubim Grande	P01	Culicidae	Culicinae	*Culex*	*Culex sp.*	15
2022	Junho	13/06/2022	24	160	Samauma	P08	Culicidae	Culicinae	*Culex*	*Culex sp.*	33
2022	Junho	13/06/2022	24	160	Samauma	P08	Culicidae	Culicinae	*Mansonia*	*Mansonia sp.*	67
2022	Junho	13/06/2022	24	160	Morrinhos	P04	Culicidae	Culicinae	*Mansonia*	*Mansonia sp.*	37
2022	Junho	13/06/2022	24	160	Teotônio	P03	Culicidae	Culicinae	*Mansonia*	*Mansonia sp.*	10
2022	Junho	13/06/2022	24	160	Teotônio	P03	Culicidae	Culicinae	*Culex*	*Culex sp.*	10
2022	Junho	13/06/2022	24	160	São Domingos	P02	Culicidae	Anophelinae	*Anopheles*	*Anopheles sp.*	8
2022	Junho	13/06/2022	24	160	São Domingos	P02	Culicidae	Culicinae	*Mansonia*	*Mansonia sp.*	142
2022	Junho	13/06/2022	24	160	São Domingos	P02	Culicidae	Culicinae	*Culex*	*Culex sp.*	72
2022	Junho	13/06/2022	24	160	Cujubim Grande	P01	Culicidae	Culicinae	*Coquillettidia*	*Coquillettidia sp.*	2
2022	Junho	13/06/2022	24	160	Cujubim Grande	P01	Culicidae	Culicinae	*Mansonia*	*Mansonia sp.*	5
2022	Junho	13/06/2022	24	160	Cujubim Grande	P01	Culicidae	Culicinae	*Culex*	*Culex sp.*	21
2022	Junho	20/06/2022	25	161	Samauma	P08	Culicidae	Culicinae	*Mansonia*	*Mansonia sp.*	378
2022	Junho	20/06/2022	25	161	Samauma	P08	Culicidae	Culicinae	*Culex*	*Culex sp.*	59
2022	Junho	20/06/2022	25	161	Samauma	P08	Culicidae	Culicinae	*Aedeomyia*	*Adeomyia squamipennis*	6
2022	Junho	20/06/2022	25	161	Morrinhos	P04	Culicidae	Culicinae	*Mansonia*	*Mansonia sp.*	101
2022	Junho	20/06/2022	25	161	Morrinhos	P04	Culicidae	Culicinae	*Culex*	*Culex sp.*	24
2022	Junho	20/06/2022	25	161	Teotônio	P03	Culicidae	Culicinae	*Mansonia*	*Mansonia sp.*	9
2022	Junho	20/06/2022	25	161	São Domingos	P02	Culicidae	Culicinae	*Mansonia*	*Mansonia sp.*	308
2022	Junho	20/06/2022	25	161	São Domingos	P02	Culicidae	Culicinae	*Culex*	*Culex sp.*	53
2022	Junho	20/06/2022	25	161	São Domingos	P02	Culicidae	Culicinae	*Coquillettidia*	*Coquillettidia sp.*	38
2022	Junho	20/06/2022	25	161	Cujubim Grande	P01	Culicidae	Culicinae	*Mansonia*	*Mansonia sp.*	2
2022	Junho	20/06/2022	25	161	Cujubim Grande	P01	Culicidae	Culicinae	*Culex*	*Culex sp.*	57
2022	Junho	27/06/2022	26	162	Samauma	P08	Culicidae	Culicinae	*Mansonia*	*Mansonia sp.*	298
2022	Junho	27/06/2022	26	162	Samauma	P08	Culicidae	Culicinae	*Culex*	*Culex sp.*	31
2022	Junho	27/06/2022	26	162	Morrinhos	P04	Culicidae	Culicinae	*Mansonia*	*Mansonia sp.*	519
2022	Junho	27/06/2022	26	162	Teotônio	P03	Culicidae	Culicinae	*Coquillettidia*	*Coquillettidia sp.*	2
2022	Junho	27/06/2022	26	162	Teotônio	P03	Culicidae	Culicinae	*Mansonia*	*Mansonia sp.*	13
2022	Junho	27/06/2022	26	162	São Domingos	P02	Culicidae	Culicinae	*Mansonia*	*Mansonia sp.*	53
2022	Junho	27/06/2022	26	162	São Domingos	P02	Culicidae	Culicinae	*Culex*	*Culex sp.*	11
2022	Junho	27/06/2022	26	162	Cujubim Grande	P01	Culicidae	Culicinae	*Culex*	*Culex sp.*	62
2022	Julho	04/07/2022	27	163	Samauma	P08	Culicidae	Culicinae	*Mansonia*	*Mansonia sp.*	76
2022	Julho	04/07/2022	27	163	Morrinhos	P04	Culicidae	Culicinae	*Mansonia*	*Mansonia sp.*	28
2022	Julho	04/07/2022	27	163	Teotônio	P03	Culicidae	Culicinae	*Coquillettidia*	*Coquillettidia sp.*	5
2022	Julho	04/07/2022	27	163	Teotônio	P03	Culicidae	Culicinae	*Mansonia*	*Mansonia sp.*	8
2022	Julho	04/07/2022	27	163	São Domingos	P02	Culicidae	Culicinae	*Mansonia*	*Mansonia sp.*	3
2022	Julho	04/07/2022	27	163	São Domingos	P02	Culicidae	Culicinae	*Culex*	*Culex sp.*	8
2022	Julho	04/07/2022	27	163	Cujubim Grande	P01	Culicidae	Anophelinae	*Anopheles*	*Anopheles sp.*	1
2022	Julho	04/07/2022	27	163	Cujubim Grande	P01	Culicidae	Culicinae	*Mansonia*	*Mansonia sp.*	2
2022	Julho	04/07/2022	27	163	Cujubim Grande	P01	Culicidae	Culicinae	*Culex*	*Culex sp.*	76
2022	Julho	11/07/2022	28	164	Samauma	P08	Culicidae	Culicinae	*Mansonia*	*Mansonia sp.*	257
2022	Julho	11/07/2022	28	164	Morrinhos	P04	Culicidae	Culicinae	*Mansonia*	*Mansonia sp.*	33
2022	Julho	11/07/2022	28	164	Teotônio	P03	Culicidae	Culicinae	*Coquillettidia*	*Coquillettidia sp.*	25
2022	Julho	11/07/2022	28	164	Teotônio	P03	Culicidae	Culicinae	*Mansonia*	*Mansonia sp.*	59
2022	Julho	11/07/2022	28	164	São Domingos	P02	Culicidae	Culicinae	*Culex*	*Culex sp.*	30
2022	Julho	11/07/2022	28	164	São Domingos	P02	Culicidae	Culicinae	*Mansonia*	*Mansonia sp.*	20
2022	Julho	11/07/2022	28	164	São Domingos	P02	Culicidae	Anophelinae	*Anopheles*	*Anopheles sp.*	3
2022	Julho	11/07/2022	28	164	Cujubim Grande	P01	Culicidae	Culicinae	*Mansonia*	*Mansonia sp.*	11
2022	Julho	11/07/2022	28	164	Cujubim Grande	P01	Culicidae	Culicinae	*Culex*	*Culex sp.*	106
2022	Julho	25/07/2022	29	165	Samauma	P08	Culicidae	Culicinae	*Mansonia*	*Mansonia sp.*	17
2022	Julho	25/07/2022	29	165	Samauma	P08	Culicidae	Culicinae	*Culex*	*Culex sp.*	70
2022	Julho	25/07/2022	29	165	Morrinhos	P04	Culicidae	Culicinae	*Mansonia*	*Mansonia sp.*	26
2022	Julho	25/07/2022	29	165	Teotônio	P03	Culicidae	Culicinae	*Mansonia*	*Mansonia sp.*	11
2022	Julho	25/07/2022	29	165	São Domingos	P02	Culicidae	Culicinae	*Culex*	*Culex sp.*	6
2022	Julho	25/07/2022	29	165	São Domingos	P02	Culicidae	Culicinae	*Mansonia*	*Mansonia sp.*	57
2022	Julho	25/07/2022	29	165	Cujubim Grande	P01	Culicidae	Culicinae	*Mansonia*	*Mansonia sp.*	9
2022	Julho	25/07/2022	29	165	Cujubim Grande	P01	Culicidae	Culicinae	*Culex*	*Culex sp.*	147
2022	Agosto	01/08/2022	30	166	Morrinhos	P04	Culicidae	Culicinae	*Mansonia*	*Mansonia sp.*	14

2022	Agosto	01/08/2022	30	166	Morrinhos	P04	Culicidae	Culicinae	*Culex*	*Culex sp.*	8
2022	Agosto	01/08/2022	30	166	Samauma	P08	Culicidae	Culicinae	*Mansonia*	*Mansonia sp.*	38
2022	Agosto	01/08/2022	30	166	Samauma	P08	Culicidae	Culicinae	*Culex*	*Culex sp.*	23
2022	Agosto	01/08/2022	30	166	Teotônio	P03	Culicidae	Culicinae	*Mansonia*	*Mansonia sp.*	339
2022	Agosto	01/08/2022	30	166	Teotônio	P03	Culicidae	Culicinae	*Coquillettidia*	*Coquillettidia sp.*	62
2022	Agosto	01/08/2022	30	166	Teotônio	P03	Culicidae	Culicinae	*Culex*	*Culex sp.*	6
2022	Agosto	01/08/2022	30	166	Teotônio	P03	Culicidae	Culicinae	*Uranotaenia*	*Uranotaenia sp.*	4
2022	Agosto	01/08/2022	30	166	Teotônio	P03	Culicidae	Anophelinae	*Anopheles*	*Anopheles sp.*	3
2022	Agosto	01/08/2022	30	166	Teotônio	P03	Culicidae	Culicinae	*Aedes*	*Aedes albopictus*	1
2022	Agosto	01/08/2022	30	166	São Domingos	P02	Culicidae	Culicinae	*Mansonia*	*Mansonia sp.*	132
2022	Agosto	01/08/2022	30	166	São Domingos	P02	Culicidae	Culicinae	*Culex*	*Culex sp.*	113
2022	Agosto	01/08/2022	30	166	São Domingos	P02	Culicidae	Culicinae	*Coquillettidia*	*Coquillettidia sp.*	17
2022	Agosto	01/08/2022	30	166	São Domingos	P02	Culicidae	Anophelinae	*Anopheles*	*Anopheles sp.*	3
2022	Agosto	01/08/2022	30	166	São Domingos	P02	Culicidae	Culicinae	*Aedes*	*Aedes albopictus*	1
2022	Agosto	01/08/2022	30	166	Cujubim Grande	P01	Culicidae	Culicinae	*Mansonia*	*Mansonia sp.*	10
2022	Agosto	01/08/2022	30	166	Cujubim Grande	P01	Culicidae	Culicinae	*Culex*	*Culex sp.*	58
2022	Agosto	01/08/2022	30	166	Cujubim Grande	P01	Culicidae	Anophelinae	*Anopheles*	*Anopheles sp.*	1
2022	Agosto	08/08/2022	31	167	Morrinhos	P04	Culicidae	Culicinae	*Mansonia*	*Mansonia sp.*	290
2022	Agosto	08/08/2022	31	167	Morrinhos	P04	Culicidae	Culicinae	*Culex*	*Culex sp.*	8
2022	Agosto	08/08/2022	31	167	Morrinhos	P04	Culicidae	Culicinae	*Coquillettidia*	*Coquillettidia sp.*	1
2022	Agosto	08/08/2022	31	167	Samauma	P08	Culicidae	Culicinae	*Mansonia*	*Mansonia sp.*	279
2022	Agosto	08/08/2022	31	167	Samauma	P08	Culicidae	Culicinae	*Culex*	*Culex sp.*	106
2022	Agosto	08/08/2022	31	167	Samauma	P08	Culicidae	Culicinae	*Coquillettidia*	*Coquillettidia sp.*	1
2022	Agosto	08/08/2022	31	167	Samauma	P08	Culicidae	Culicinae	*Aedeomyia*	*Adeomyia squamipennis*	1
2022	Agosto	08/08/2022	31	167	Teotônio	P03	Culicidae	Culicinae	*Mansonia*	*Mansonia sp.*	164
2022	Agosto	08/08/2022	31	167	Teotônio	P03	Culicidae	Culicinae	*Coquillettidia*	*Coquillettidia sp.*	38
2022	Agosto	08/08/2022	31	167	Teotônio	P03	Culicidae	Culicinae	*Culex*	*Culex sp.*	1
2022	Agosto	08/08/2022	31	167	Teotônio	P03	Culicidae	Anophelinae	*Anopheles*	*Anopheles sp.*	8
2022	Agosto	08/08/2022	31	167	Teotônio	P03	Culicidae	Culicinae	*Aedeomyia*	*Adeomyia squamipennis*	1
2022	Agosto	08/08/2022	31	167	São Domingos	P02	Culicidae	Culicinae	*Mansonia*	*Mansonia sp.*	250
2022	Agosto	08/08/2022	31	167	São Domingos	P02	Culicidae	Culicinae	*Culex*	*Culex sp.*	25
2022	Agosto	08/08/2022	31	167	São Domingos	P02	Culicidae	Culicinae	*Coquillettidia*	*Coquillettidia sp.*	24
2022	Agosto	08/08/2022	31	167	São Domingos	P02	Culicidae	Anophelinae	*Anopheles*	*Anopheles sp.*	12
2022	Agosto	08/08/2022	31	167	Cujubim Grande	P01	Culicidae	Culicinae	*Mansonia*	*Mansonia sp.*	33
2022	Agosto	08/08/2022	31	167	Cujubim Grande	P01	Culicidae	Culicinae	*Culex*	*Culex sp.*	23
2022	Agosto	08/08/2022	31	167	Cujubim Grande	P01	Culicidae	Culicinae	*Aedes*	*Aedes albopictus*	1
2022	Agosto	15/08/2022	32	168	Morrinhos	P04	Culicidae	Culicinae	*Mansonia*	*Mansonia sp.*	34
2022	Agosto	15/08/2022	32	168	Morrinhos	P04	Culicidae	Culicinae	*Culex*	*Culex sp.*	3
2022	Agosto	15/08/2022	32	168	Morrinhos	P04	Culicidae	Anophelinae	*Anopheles*	*Anopheles sp.*	1
2022	Agosto	15/08/2022	32	168	Samauma	P08	Culicidae	Culicinae	*Mansonia*	*Mansonia sp.*	50
2022	Agosto	15/08/2022	32	168	Samauma	P08	Culicidae	Culicinae	*Culex*	*Culex sp.*	92
2022	Agosto	15/08/2022	32	168	Samauma	P08	Culicidae	Culicinae	*Aedeomyia*	*Adeomyia squamipennis*	1
2022	Agosto	15/08/2022	32	168	Teotônio	P03	Culicidae	Culicinae	*Mansonia*	*Mansonia sp.*	12
2022	Agosto	15/08/2022	32	168	Teotônio	P03	Culicidae	Culicinae	*Culex*	*Culex sp.*	3
2022	Agosto	15/08/2022	32	168	São Domingos	P02	Culicidae	Culicinae	*Mansonia*	*Mansonia sp.*	237
2022	Agosto	15/08/2022	32	168	São Domingos	P02	Culicidae	Culicinae	*Culex*	*Culex sp.*	28
2022	Agosto	15/08/2022	32	168	São Domingos	P02	Culicidae	Culicinae	*Coquillettidia*	*Coquillettidia sp.*	45
2022	Agosto	15/08/2022	32	168	São Domingos	P02	Culicidae	Anophelinae	*Anopheles*	*Anopheles sp.*	13
2022	Agosto	15/08/2022	32	168	Cujubim Grande	P01	Culicidae	Culicinae	*Mansonia*	*Mansonia sp.*	11
2022	Agosto	15/08/2022	32	168	Cujubim Grande	P01	Culicidae	Culicinae	*Culex*	*Culex sp.*	43
2022	Agosto	22/08/2022	33	169	Morrinhos	P04	Culicidae	Culicinae	*Mansonia*	*Mansonia sp.*	653
2022	Agosto	22/08/2022	33	169	Morrinhos	P04	Culicidae	Culicinae	*Culex*	*Culex sp.*	11
2022	Agosto	22/08/2022	33	169	Morrinhos	P04	Culicidae	Culicinae	*Coquillettidia*	*Coquillettidia sp.*	3
2022	Agosto	22/08/2022	33	169	Samauma	P08	Culicidae	Culicinae	*Mansonia*	*Mansonia sp.*	122
2022	Agosto	22/08/2022	33	169	Samauma	P08	Culicidae	Culicinae	*Culex*	*Culex sp.*	66
2022	Agosto	22/08/2022	33	169	Samauma	P08	Culicidae	Culicinae	*Coquillettidia*	*Coquillettidia sp.*	1
2022	Agosto	22/08/2022	33	169	Samauma	P08	Culicidae	Culicinae	*Uranotaenia*	*Uranotaenia lowii*	1
2022	Agosto	22/08/2022	33	169	Samauma	P08	Culicidae	Culicinae	*Psorophora*	*Psorophora sp.*	1
2022	Agosto	22/08/2022	33	169	Teotônio	P03	Culicidae	Culicinae	*Mansonia*	*Mansonia sp.*	23
2022	Agosto	22/08/2022	33	169	Teotônio	P03	Culicidae	Culicinae	*Culex*	*Culex sp.*	2
2022	Agosto	22/08/2022	33	169	São Domingos	P02	Culicidae	Culicinae	*Mansonia*	*Mansonia sp.*	120
2022	Agosto	22/08/2022	33	169	São Domingos	P02	Culicidae	Culicinae	*Culex*	*Culex sp.*	14
2022	Agosto	22/08/2022	33	169	São Domingos	P02	Culicidae	Culicinae	*Coquillettidia*	*Coquillettidia sp.*	7
2022	Agosto	22/08/2022	33	169	São Domingos	P02	Culicidae	Anophelinae	*Anopheles*	*Anopheles sp.*	4
2022	Agosto	22/08/2022	33	169	Cujubim Grande	P01	Culicidae	Culicinae	*Mansonia*	*Mansonia sp.*	3
2022	Agosto	22/08/2022	33	169	Cujubim Grande	P01	Culicidae	Culicinae	*Culex*	*Culex sp.*	16
2022	Agosto	29/08/2022	34	170	Morrinhos	P04	Culicidae	Culicinae	*Mansonia*	*Mansonia sp.*	1101
2022	Agosto	29/08/2022	34	170	Morrinhos	P04	Culicidae	Culicinae	*Culex*	*Culex sp.*	40
2022	Agosto	29/08/2022	34	170	Morrinhos	P04	Culicidae	Anophelinae	*Anopheles*	*Anopheles sp.*	3
2022	Agosto	29/08/2022	34	170	Morrinhos	P04	Culicidae	Culicinae	*Psorophora*	*Psorophora sp.*	1
2022	Agosto	29/08/2022	34	170	Samauma	P08	Culicidae	Culicinae	*Mansonia*	*Mansonia sp.*	156
2022	Agosto	29/08/2022	34	170	Samauma	P08	Culicidae	Culicinae	*Culex*	*Culex sp.*	56
2022	Agosto	29/08/2022	34	170	Samauma	P08	Culicidae	Culicinae	*Coquillettidia*	*Coquillettidia sp.*	2
2022	Agosto	29/08/2022	34	170	Samauma	P08	Culicidae	Culicinae	*Aedes*	*Aedes aegypti*	1
2022	Agosto	29/08/2022	34	170	Teotônio	P03	Culicidae	Culicinae	*Mansonia*	*Mansonia sp.*	252
2022	Agosto	29/08/2022	34	170	Teotônio	P03	Culicidae	Culicinae	*Culex*	*Culex sp.*	9
2022	Agosto	29/08/2022	34	170	Teotônio	P03	Culicidae	Culicinae	*Coquillettidia*	*Coquillettidia sp.*	10
2022	Agosto	29/08/2022	34	170	Teotônio	P03	Culicidae	Culicinae	*Aedeomyia*	*Adeomyia squamipennis*	3
2022	Agosto	29/08/2022	34	170	Teotônio	P03	Culicidae	Anophelinae	*Anopheles*	*Anopheles sp.*	6
2022	Agosto	29/08/2022	34	170	Teotônio	P03	Culicidae	Culicinae	*Uranotaenia*	*Uranotaenia sp.*	3
2022	Agosto	29/08/2022	34	170	Teotônio	P03	Culicidae	Culicinae	*Wyeomyia*	*Wyeomyia sp.*	1
2022	Agosto	29/08/2022	34	170	Teotônio	P03	Culicidae	Culicinae	*Aedes*	*Aedes albopictus*	1
2022	Agosto	29/08/2022	34	170	São Domingos	P02	Culicidae	Culicinae	*Mansonia*	*Mansonia sp.*	10
2022	Agosto	29/08/2022	34	170	São Domingos	P02	Culicidae	Culicinae	*Culex*	*Culex sp.*	1
2022	Agosto	29/08/2022	34	170	Cujubim Grande	P01	Culicidae	Culicinae	*Mansonia*	*Mansonia sp.*	30
2022	Agosto	29/08/2022	34	170	Cujubim Grande	P01	Culicidae	Culicinae	*Culex*	*Culex sp.*	4
2022	Agosto	29/08/2022	34	170	Cujubim Grande	P01	Culicidae	Culicinae	*Aedes*	*Aedes sp.*	1
2022	Setembro	05/09/2022	35	171	Morrinhos	P04	Culicidae	Culicinae	*Mansonia*	*Mansonia sp.*	1305
2022	Setembro	05/09/2022	35	171	Morrinhos	P04	Culicidae	Culicinae	*Culex*	*Culex sp.*	35
2022	Setembro	05/09/2022	35	171	Morrinhos	P04	Culicidae	Culicinae	*Coquillettidia*	*Coquillettidia sp.*	4
2022	Setembro	05/09/2022	35	171	Morrinhos	P04	Culicidae	Culicinae	*Aedes*	*Aedes albopictus*	1
2022	Setembro	05/09/2022	35	171	Morrinhos	P04	Culicidae	Anophelinae	*Anopheles*	*Anopheles sp.*	4
2022	Setembro	05/09/2022	35	171	Samauma	P08	Culicidae	Culicinae	*Mansonia*	*Mansonia sp.*	130
2022	Setembro	05/09/2022	35	171	Samauma	P08	Culicidae	Culicinae	*Coquillettidia*	*Coquillettidia sp.*	4
2022	Setembro	05/09/2022	35	171	Samauma	P08	Culicidae	Culicinae	*Culex*	*Culex sp.*	68
2022	Setembro	05/09/2022	35	171	Teotônio	P03	Culicidae	Culicinae	*Mansonia*	*Mansonia sp.*	248
2022	Setembro	05/09/2022	35	171	Teotônio	P03	Culicidae	Culicinae	*Coquillettidia*	*Coquillettidia sp.*	24
2022	Setembro	05/09/2022	35	171	Teotônio	P03	Culicidae	Culicinae	*Culex*	*Culex sp.*	4
2022	Setembro	05/09/2022	35	171	Teotônio	P03	Culicidae	Culicinae	*Aedes*	*Aedes albopictus*	3
2022	Setembro	05/09/2022	35	171	Teotônio	P03	Culicidae	Anophelinae	*Anopheles*	*Anopheles sp.*	1
2022	Setembro	05/09/2022	35	171	São Domingos	P02	Culicidae	Culicinae	*Mansonia*	*Mansonia sp.*	23
2022	Setembro	05/09/2022	35	171	São Domingos	P02	Culicidae	Culicinae	*Coquillettidia*	*Coquillettidia sp.*	6
2022	Setembro	05/09/2022	35	171	São Domingos	P02	Culicidae	Anophelinae	*Anopheles*	*Anopheles sp.*	1
2022	Setembro	05/09/2022	35	171	Cujubim Grande	P01	Culicidae	Culicinae	*Mansonia*	*Mansonia sp.*	7
2022	Setembro	05/09/2022	35	171	Cujubim Grande	P01	Culicidae	Culicinae	*Culex*	*Culex sp.*	21
2022	Setembro	12/09/2022	36	172	Samauma	P08	Culicidae	Culicinae	*Mansonia*	*Mansonia sp.*	58
2022	Setembro	12/09/2022	36	172	Samauma	P08	Culicidae	Culicinae	*Culex*	*Culex sp.*	16
2022	Setembro	12/09/2022	36	172	Teotônio	P03	Culicidae	Culicinae	*Mansonia*	*Mansonia sp.*	48
2022	Setembro	12/09/2022	36	172	Teotônio	P03	Culicidae	Culicinae	*Coquillettidia*	*Coquillettidia sp.*	39
2022	Setembro	12/09/2022	36	172	Teotônio	P03	Culicidae	Anophelinae	*Anopheles*	*Anopheles sp.*	3
2022	Setembro	12/09/2022	36	172	Teotônio	P03	Culicidae	Culicinae	*Culex*	*Culex sp.*	2
2022	Setembro	12/09/2022	36	172	Teotônio	P03	Culicidae	Culicinae	*Aedes*	*Aedes albopictus*	1
2022	Setembro	12/09/2022	36	172	São Domingos	P02	Culicidae	Culicinae	*Mansonia*	*Mansonia sp.*	84
2022	Setembro	12/09/2022	36	172	São Domingos	P02	Culicidae	Culicinae	*Coquillettidia*	*Coquillettidia sp.*	44
2022	Setembro	12/09/2022	36	172	São Domingos	P02	Culicidae	Culicinae	*Aedeomyia*	*Adeomyia squamipennis*	5
2022	Setembro	12/09/2022	36	172	São Domingos	P02	Culicidae	Anophelinae	*Anopheles*	*Anopheles sp.*	2
2022	Setembro	12/09/2022	36	172	São Domingos	P02	Culicidae	Culicinae	*Culex*	*Culex sp.*	1
2022	Setembro	12/09/2022	36	172	Cujubim Grande	P01	Culicidae	Culicinae	*Mansonia*	*Mansonia sp.*	2

2022	Setembro	12/09/2022	36	172	Cujubim Grande	P01	Culicidae	Culicinae	*Culex*	*Culex sp.*	17
2022	Setembro	19/09/2022	37	173	Samauma	P08	Culicidae	Culicinae	*Culex*	*Culex sp.*	111
2022	Setembro	19/09/2022	37	173	Samauma	P08	Culicidae	Culicinae	*Mansonia*	*Mansonia sp.*	63
2022	Setembro	19/09/2022	37	173	Samauma	P08	Culicidae	Culicinae	*Coquillettidia*	*Coquillettidia sp.*	8
2022	Setembro	19/09/2022	37	173	Morrinhos	P04	Culicidae	Culicinae	*Mansonia*	*Mansonia sp.*	251
2022	Setembro	19/09/2022	37	173	Morrinhos	P04	Culicidae	Culicinae	*Culex*	*Culex sp.*	7
2022	Setembro	19/09/2022	37	173	Morrinhos	P04	Culicidae	Culicinae	*Aedes*	*Aedes aegypti*	2
2022	Setembro	19/09/2022	37	173	Morrinhos	P04	Culicidae	Culicinae	*Coquillettidia*	*Coquillettidia sp.*	2
2022	Setembro	19/09/2022	37	173	Teotônio	P03	Culicidae	Culicinae	*Mansonia*	*Mansonia sp.*	147
2022	Setembro	19/09/2022	37	173	Teotônio	P03	Culicidae	Culicinae	*Coquillettidia*	*Coquillettidia sp.*	59
2022	Setembro	19/09/2022	37	173	Teotônio	P03	Culicidae	Culicinae	*Culex*	*Culex sp.*	3
2022	Setembro	19/09/2022	37	173	Teotônio	P03	Culicidae	Anophelinae	*Anopheles*	*Anopheles sp.*	4
2022	Setembro	19/09/2022	37	173	Teotônio	P03	Culicidae	Culicinae	*Aedes*	*Aedes aegypti*	2
2022	Setembro	19/09/2022	37	173	São Domingos	P02	Culicidae	Culicinae	*Mansonia*	*Mansonia sp.*	134
2022	Setembro	19/09/2022	37	173	São Domingos	P02	Culicidae	Culicinae	*Coquillettidia*	*Coquillettidia sp.*	59
2022	Setembro	19/09/2022	37	173	São Domingos	P02	Culicidae	Culicinae	*Aedeomyia*	*Adeomyia squamipennis*	3
2022	Setembro	19/09/2022	37	173	São Domingos	P02	Culicidae	Culicinae	*Culex*	*Culex sp.*	2
2022	Setembro	19/09/2022	37	173	Cujubim Grande	P01	Culicidae	Culicinae	*Mansonia*	*Mansonia sp.*	4
2022	Setembro	19/09/2022	37	173	Cujubim Grande	P01	Culicidae	Culicinae	*Culex*	*Culex sp.*	9
2022	Setembro	26/09/2022	38	174	Samauma	P08	Culicidae	Culicinae	*Culex*	*Culex sp.*	98
2022	Setembro	26/09/2022	38	174	Samauma	P08	Culicidae	Culicinae	*Mansonia*	*Mansonia sp.*	26
2022	Setembro	26/09/2022	38	174	Samauma	P08	Culicidae	Culicinae	*Coquillettidia*	*Coquillettidia sp.*	4
2022	Setembro	26/09/2022	38	174	Morrinhos	P04	Culicidae	Culicinae	*Mansonia*	*Mansonia sp.*	221
2022	Setembro	26/09/2022	38	174	Morrinhos	P04	Culicidae	Culicinae	*Coquillettidia*	*Coquillettidia sp.*	8
2022	Setembro	26/09/2022	38	174	Teotônio	P03	Culicidae	Culicinae	*Mansonia*	*Mansonia sp.*	54
2022	Setembro	26/09/2022	38	174	Teotônio	P03	Culicidae	Culicinae	*Coquillettidia*	*Coquillettidia sp.*	27
2022	Setembro	26/09/2022	38	174	Teotônio	P03	Culicidae	Anophelinae	*Anopheles*	*Anopheles sp.*	5
2022	Setembro	26/09/2022	38	174	Teotônio	P03	Culicidae	Culicinae	*Aedeomyia*	*Adeomyia squamipennis*	2
2022	Setembro	26/09/2022	38	174	Teotônio	P03	Culicidae	Culicinae	*Culex*	*Culex sp.*	2
2022	Setembro	26/09/2022	38	174	Teotônio	P03	Culicidae	Culicinae	*Aedes*	*Aedes aegypti*	1
2022	Setembro	26/09/2022	38	174	São Domingos	P02	Culicidae	Culicinae	*Mansonia*	*Mansonia sp.*	57
2022	Setembro	26/09/2022	38	174	São Domingos	P02	Culicidae	Culicinae	*Coquillettidia*	*Coquillettidia sp.*	24
2022	Setembro	26/09/2022	38	174	São Domingos	P02	Culicidae	Culicinae	*Culex*	*Culex sp.*	13
2022	Setembro	26/09/2022	38	174	São Domingos	P02	Culicidae	Culicinae	*Aedeomyia*	*Adeomyia squamipennis*	3
2022	Setembro	26/09/2022	38	174	São Domingos	P02	Culicidae	Anophelinae	*Anopheles*	*Anopheles sp.*	2
2022	Setembro	26/09/2022	38	174	Cujubim Grande	P01	Culicidae	Culicinae	*Culex*	*Culex sp.*	7
2022	Outubro	03/10/2022	39	175	Samauma	P08	Culicidae	Culicinae	*Culex*	*Culex sp.*	140
2022	Outubro	03/10/2022	39	175	Samauma	P08	Culicidae	Culicinae	*Mansonia*	*Mansonia sp.*	17
2022	Outubro	03/10/2022	39	175	Morrinhos	P04	Culicidae	Culicinae	*Mansonia*	*Mansonia sp.*	137
2022	Outubro	03/10/2022	39	175	Morrinhos	P04	Culicidae	Culicinae	*Culex*	*Culex sp.*	9
2022	Outubro	03/10/2022	39	175	Teotônio	P03	Culicidae	Culicinae	*Mansonia*	*Mansonia sp.*	143
2022	Outubro	03/10/2022	39	175	Teotônio	P03	Culicidae	Culicinae	*Coquillettidia*	*Coquillettidia sp.*	34
2022	Outubro	03/10/2022	39	175	Teotônio	P03	Culicidae	Anophelinae	*Anopheles*	*Anopheles sp.*	10
2022	Outubro	03/10/2022	39	175	Teotônio	P03	Culicidae	Culicinae	*Culex*	*Culex sp.*	6
2022	Outubro	03/10/2022	39	175	Teotônio	P03	Culicidae	Culicinae	*Aedeomyia*	*Adeomyia squamipennis*	3
2022	Outubro	03/10/2022	39	175	São Domingos	P02	Culicidae	Culicinae	*Mansonia*	*Mansonia sp.*	133
2022	Outubro	03/10/2022	39	175	São Domingos	P02	Culicidae	Culicinae	*Coquillettidia*	*Coquillettidia sp.*	104
2022	Outubro	03/10/2022	39	175	São Domingos	P02	Culicidae	Culicinae	*Culex*	*Culex sp.*	15
2022	Outubro	03/10/2022	39	175	São Domingos	P02	Culicidae	Culicinae	*Aedeomyia*	*Adeomyia squamipennis*	1
2022	Outubro	03/10/2022	39	175	Cujubim Grande	P01	Culicidae	Culicinae	*Mansonia*	*Mansonia sp.*	2
2022	Outubro	03/10/2022	39	175	Cujubim Grande	P01	Culicidae	Culicinae	*Culex*	*Culex sp.*	6
2022	Outubro	03/10/2022	39	175	Cujubim Grande	P01	Culicidae	Culicinae	*Aedes*	*Aedes aegypti*	3
2022	Outubro	17/10/2022	42	178	Samauma	P08	Culicidae	Culicinae	*Mansonia*	*Mansonia sp.*	23
2022	Outubro	17/10/2022	42	178	Teotônio	P03	Culicidae	Culicinae	*Culex*	*Culex sp.*	5
2022	Outubro	17/10/2022	42	178	Teotônio	P03	Culicidae	Culicinae	*Mansonia*	*Mansonia sp.*	12
2022	Outubro	17/10/2022	42	178	Teotônio	P03	Culicidae	Culicinae	*Aedes*	*Aedes sp.*	1
2022	Outubro	17/10/2022	42	178	Teotônio	P03	Culicidae	Anophelinae	*Anopheles*	*Anopheles sp.*	1
2022	Outubro	17/10/2022	42	178	Teotônio	P03	Culicidae	Culicinae	*Coquillettidia*	*Coquillettidia sp.*	142
2022	Outubro	17/10/2022	42	178	São Domingos	P02	Culicidae	Culicinae	*Mansonia*	*Mansonia sp.*	74
2022	Outubro	17/10/2022	42	178	São Domingos	P02	Culicidae	Culicinae	*Coquillettidia*	*Coquillettidia sp.*	9
2022	Outubro	17/10/2022	42	178	São Domingos	P02	Culicidae	Culicinae	*Culex*	*Culex sp.*	4
2022	Outubro	17/10/2022	42	178	São Domingos	P02	Culicidae	Culicinae	*Aedeomyia*	*Adeomyia squamipennis*	1
2022	Outubro	17/10/2022	42	178	Cujubim Grande	P01	Culicidae	Culicinae	*Culex*	*Culex sp.*	2
2022	Outubro	24/10/2022	43	179	Samauma	P08	Culicidae	Culicinae	*Mansonia*	*Mansonia sp.*	19
2022	Outubro	24/10/2022	43	179	Samauma	P08	Culicidae	Culicinae	*Coquillettidia*	*Coquillettidia sp.*	1
2022	Outubro	24/10/2022	43	179	Samauma	P08	Culicidae	Culicinae	*Culex*	*Culex sp.*	72
2022	Outubro	24/10/2022	43	179	Morrinhos	P04	Culicidae	Culicinae	*Mansonia*	*Mansonia sp.*	139
2022	Outubro	24/10/2022	43	179	Morrinhos	P04	Culicidae	Culicinae	*Coquillettidia*	*Coquillettidia sp.*	1
2022	Outubro	24/10/2022	43	179	Morrinhos	P04	Culicidae	Culicinae	*Aedes*	*Aedes sp.*	8
2022	Outubro	24/10/2022	43	179	Morrinhos	P04	Culicidae	Culicinae	*Culex*	*Culex sp.*	8
2022	Outubro	24/10/2022	43	179	Teotônio	P03	Culicidae	Culicinae	*Mansonia*	*Mansonia sp.*	28
2022	Outubro	24/10/2022	43	179	São Domingos	P02	Culicidae	Culicinae	*Mansonia*	*Mansonia sp.*	78
2022	Outubro	24/10/2022	43	179	São Domingos	P02	Culicidae	Culicinae	*Coquillettidia*	*Coquillettidia sp.*	5
2022	Outubro	24/10/2022	43	179	São Domingos	P02	Culicidae	Culicinae	*Aedes*	*Aedes sp.*	1
2022	Outubro	24/10/2022	43	179	São Domingos	P02	Culicidae	Culicinae	*Culex*	*Culex sp.*	15
2022	Outubro	24/10/2022	43	179	Cujubim Grande	P01	Culicidae	Culicinae	*Culex*	*Culex sp.*	9
2022	Outubro	24/10/2022	43	179	Cujubim Grande	P01	Culicidae	Culicinae	*Aedes*	*Aedes sp.*	2
2022	Outubro	31/10/2022	44	180	Samauma	P08	Culicidae	Culicinae	*Mansonia*	*Mansonia sp.*	8
2022	Outubro	31/10/2022	44	180	Morrinhos	P04	Culicidae	Culicinae	*Mansonia*	*Mansonia sp.*	178
2022	Outubro	31/10/2022	44	180	Morrinhos	P04	Culicidae	Culicinae	*Culex*	*Culex sp.*	3
2022	Outubro	31/10/2022	44	180	Morrinhos	P04	Culicidae	Culicinae	*Aedes*	*Aedes sp.*	2
2022	Outubro	31/10/2022	44	180	Teotônio	P03	Culicidae	Culicinae	*Mansonia*	*Mansonia sp.*	2
2022	Outubro	31/10/2022	44	180	Teotônio	P03	Culicidae	Culicinae	*Wyeomyia*	*Wyeomyia sp.*	1
2022	Outubro	31/10/2022	44	180	Teotônio	P03	Culicidae	Culicinae	*Coquillettidia*	*Coquillettidia sp.*	13
2022	Outubro	31/10/2022	44	180	São Domingos	P02	Culicidae	Culicinae	*Culex*	*Culex sp.*	4
2022	Outubro	31/10/2022	44	180	São Domingos	P02	Culicidae	Culicinae	*Mansonia*	*Mansonia sp.*	78
2022	Outubro	31/10/2022	44	180	Cujubim Grande	P01	Culicidae	Culicinae	*Culex*	*Culex sp.*	1
2022	Outubro	10/10/2022	40	176	Samauma	P08	Culicidae	Culicinae	*Mansonia*	*Mansonia sp.*	20
2022	Outubro	10/10/2022	40	176	Samauma	P08	Culicidae	Culicinae	*Culex*	*Culex sp.*	89
2022	Outubro	10/10/2022	40	176	Morrinhos	P04	Culicidae	Culicinae	*Mansonia*	*Mansonia sp.*	79
2022	Outubro	10/10/2022	40	176	Morrinhos	P04	Culicidae	Culicinae	*Culex*	*Culex sp.*	4
2022	Outubro	10/10/2022	40	176	Morrinhos	P04	Culicidae	Culicinae	*Coquillettidia*	*Coquillettidia sp.*	3
2022	Outubro	10/10/2022	40	176	Teotônio	P03	Culicidae	Culicinae	*Mansonia*	*Mansonia sp.*	108
2022	Outubro	10/10/2022	40	176	Teotônio	P03	Culicidae	Culicinae	*Coquillettidia*	*Coquillettidia sp.*	56
2022	Outubro	10/10/2022	40	176	Teotônio	P03	Culicidae	Anophelinae	*Anopheles*	*Anopheles sp.*	4
2022	Outubro	10/10/2022	40	176	Teotônio	P03	Culicidae	Culicinae	*Culex*	*Culex sp.*	2
2022	Outubro	10/10/2022	40	176	São Domingos	P02	Culicidae	Culicinae	*Mansonia*	*Mansonia sp.*	105
2022	Outubro	10/10/2022	40	176	São Domingos	P02	Culicidae	Culicinae	*Coquillettidia*	*Coquillettidia sp.*	13
2022	Outubro	10/10/2022	40	176	São Domingos	P02	Culicidae	Culicinae	*Culex*	*Culex sp.*	4
2022	Outubro	10/10/2022	40	176	Cujubim Grande	P01	Culicidae	Culicinae	*Mansonia*	*Mansonia sp.*	10
2022	Outubro	10/10/2022	40	176	Cujubim Grande	P01	Culicidae	Culicinae	*Coquillettidia*	*Coquillettidia sp.*	1
2022	Outubro	10/10/2022	40	176	Cujubim Grande	P01	Culicidae	Culicinae	*Culex*	*Culex sp.*	7
2022	Novembro	07/11/2022	45	181	Samauma	P08	Culicidae	Culicinae	*Culex*	*Culex sp.*	12
2022	Novembro	07/11/2022	45	181	Samauma	P08	Culicidae	Culicinae	*Mansonia*	*Mansonia sp.*	15
2022	Novembro	07/11/2022	45	181	Morrinhos	P04	Culicidae	Culicinae	*Mansonia*	*Mansonia sp.*	38
2022	Novembro	07/11/2022	45	181	Morrinhos	P04	Culicidae	Culicinae	*Culex*	*Culex sp.*	77
2022	Novembro	07/11/2022	45	181	Morrinhos	P04	Culicidae	Culicinae	*Aedes*	*Aedes sp.*	6
2022	Novembro	07/11/2022	45	181	Morrinhos	P04	Culicidae	Anophelinae	*Anopheles*	*Anopheles sp.*	1
2022	Novembro	07/11/2022	45	181	Teotônio	P03	Culicidae	Culicinae	*Coquillettidia*	*Coquillettidia sp.*	17
2022	Novembro	07/11/2022	45	181	Teotônio	P03	Culicidae	Culicinae	*Mansonia*	*Mansonia sp.*	2
2022	Novembro	07/11/2022	45	181	São Domingos	P02	Culicidae	Culicinae	*Mansonia*	*Mansonia humeralis*	31
2022	Novembro	07/11/2022	45	181	São Domingos	P02	Culicidae	Culicinae	*Culex*	*Culex sp.*	1
2022	Novembro	07/11/2022	45	181	São Domingos	P02	Culicidae	Culicinae	*Aedes*	*Aedes sp.*	1
2022	Novembro	07/11/2022	45	181	São Domingos	P02	Culicidae	Culicinae	*Limatus*	*Limatus durhamii*	1
2022	Novembro	07/11/2022	45	181	São Domingos	P02	Culicidae	Culicinae	*Coquillettidia*	*Coquillettidia sp.*	97
2022	Novembro	07/11/2022	45	181	Cujubim Grande	P01	Culicidae	Anophelinae	*Anopheles*	*Anopheles sp.*	2
2022	Novembro	07/11/2022	45	181	Cujubim Grande	P01	Culicidae	Culicinae	*Culex*	*Culex sp.*	2
2022	Novembro	07/11/2022	45	181	Cujubim Grande	P01	Culicidae	Culicinae	*Mansonia*	*Mansonia titillans*	1

2022	Novembro	14/11/2022	46	182	Samauma	P08	Culicidae	Culicinae	*Mansonia*	*Mansonia sp.*	7
2022	Novembro	14/11/2022	46	182	Samauma	P08	Culicidae	Culicinae	*Culex*	*Culex sp.*	42
2022	Novembro	14/11/2022	46	182	Morrinhos	P04	Culicidae	Culicinae	*Mansonia*	*Mansonia sp.*	117
2022	Novembro	14/11/2022	46	182	Morrinhos	P04	Culicidae	Culicinae	*Culex*	*Culex sp.*	35
2022	Novembro	14/11/2022	46	182	Morrinhos	P04	Culicidae	Culicinae	*Aedes*	*Aedes sp.*	22
2022	Novembro	14/11/2022	46	182	Morrinhos	P04	Culicidae	Culicinae	*Aedes*	*Aedes albopictus*	2
2022	Novembro	14/11/2022	46	182	Morrinhos	P04	Culicidae	Anophelinae	*Anopheles*	*Anopheles sp.*	1
2022	Novembro	14/11/2022	46	182	Teotônio	P03	Culicidae	Anophelinae	*Anopheles*	*Anopheles nuneztovari s.l.*	2
2022	Novembro	14/11/2022	46	182	Teotônio	P03	Culicidae	Culicinae	*Coquillettidia*	*Coquillettidia sp.*	9
2022	Novembro	14/11/2022	46	182	Teotônio	P03	Culicidae	Culicinae	*Aedes*	*Aedes sp.*	1
2022	Novembro	14/11/2022	46	182	Teotônio	P03	Culicidae	Culicinae	*Mansonia*	*Mansonia sp.*	2
2022	Novembro	14/11/2022	46	182	São Domingos	P02	Culicidae	Culicinae	*Culex*	*Culex sp.*	7
2022	Novembro	14/11/2022	46	182	São Domingos	P02	Culicidae	Culicinae	*Coquillettidia*	*Coquillettidia sp.*	37
2022	Novembro	14/11/2022	46	182	São Domingos	P02	Culicidae	Culicinae	*Mansonia*	*Mansonia sp.*	18
2022	Novembro	14/11/2022	46	182	Cujubim Grande	P01	Culicidae	Culicinae	*Mansonia*	*Mansonia sp.*	3
2022	Novembro	14/11/2022	46	182	Cujubim Grande	P01	Culicidae	Culicinae	*Coquillettidia*	*Coquillettidia sp.*	1
2022	Novembro	14/11/2022	46	182	Cujubim Grande	P01	Culicidae	Culicinae	*Aedes*	*Aedes sp.*	1
2022	Novembro	14/11/2022	46	182	Cujubim Grande	P01	Culicidae	Culicinae	*Culex*	*Culex sp.*	1
2022	Novembro	21/11/2022	47	183	Samauma	P08	Culicidae	Culicinae	*Mansonia*	*Mansonia sp.*	8
2022	Novembro	21/11/2022	47	183	Samauma	P08	Culicidae	Culicinae	*Culex*	*Culex sp.*	77
2022	Novembro	21/11/2022	47	183	Morrinhos	P04	Culicidae	Culicinae	*Mansonia*	*Mansonia sp.*	177
2022	Novembro	21/11/2022	47	183	Morrinhos	P04	Culicidae	Culicinae	*Culex*	*Culex sp.*	18
2022	Novembro	21/11/2022	47	183	Morrinhos	P04	Culicidae	Culicinae	*Aedes*	*Aedes sp.*	6
2022	Novembro	21/11/2022	47	183	Teotônio	P03	Culicidae	Culicinae	*Mansonia*	*Mansonia sp.*	4
2022	Novembro	21/11/2022	47	183	Teotônio	P03	Culicidae	Culicinae	*Coquillettidia*	*Coquillettidia sp.*	6
2022	Novembro	21/11/2022	47	183	Teotônio	P03	Culicidae	Culicinae	*Culex*	*Culex sp.*	1
2022	Novembro	21/11/2022	47	183	São Domingos	P02	Culicidae	Culicinae	*Culex*	*Culex sp.*	13
2022	Novembro	21/11/2022	47	183	São Domingos	P02	Culicidae	Culicinae	*Mansonia*	*Mansonia sp.*	15
2022	Novembro	21/11/2022	47	183	Cujubim Grande	P01	Culicidae	Culicinae	*Culex*	*Culex sp.*	1
2022	Novembro	28/11/2022	48	184	Samauma	P08	Culicidae	Culicinae	*Culex*	*Culex sp.*	15
2022	Novembro	28/11/2022	48	184	Samauma	P08	Culicidae	Culicinae	*Mansonia*	*Mansonia sp.*	23
2022	Novembro	28/11/2022	48	184	Samauma	P08	Culicidae	Anophelinae	*Anopheles*	*Anopheles sp.*	1
2022	Novembro	28/11/2022	48	184	Morrinhos	P04	Culicidae	Culicinae	*Mansonia*	*Mansonia sp.*	235
2022	Novembro	28/11/2022	48	184	Morrinhos	P04	Culicidae	Culicinae	*Culex*	*Culex sp.*	6
2022	Novembro	28/11/2022	48	184	Morrinhos	P04	Culicidae	Culicinae	*Aedes*	*Aedes sp.*	5
2022	Novembro	28/11/2022	48	184	Morrinhos	P04	Culicidae	Culicinae	*Limatus*	*Limatus durhamii*	1
2022	Novembro	28/11/2022	48	184	Teotônio	P03	Culicidae	Anophelinae	*Anopheles*	*Anopheles sp.*	1
2022	Novembro	28/11/2022	48	184	Teotônio	P03	Culicidae	Culicinae	*Culex*	*Culex sp.*	2
2022	Novembro	28/11/2022	48	184	Teotônio	P03	Culicidae	Culicinae	*Coquillettidia*	*Coquillettidia sp.*	4
2022	Novembro	28/11/2022	48	184	Teotônio	P03	Culicidae	Culicinae	*Mansonia*	*Mansonia sp.*	8
2022	Novembro	28/11/2022	48	184	São Domingos	P02	Culicidae	Culicinae	*Mansonia*	*Mansonia sp.*	28
2022	Novembro	28/11/2022	48	184	São Domingos	P02	Culicidae	Culicinae	*Coquillettidia*	*Coquillettidia sp.*	4
2022	Novembro	28/11/2022	48	184	Cujubim Grande	P01	Culicidae	Anophelinae	*Anopheles*	*Anopheles sp.*	1
2022	Novembro	28/11/2022	48	184	Cujubim Grande	P01	Culicidae	Culicinae	*Mansonia*	*Mansonia sp.*	1
2022	Novembro	28/11/2022	48	184	Cujubim Grande	P01	Culicidae	Culicinae	*Aedes*	*Aedes sp.*	2
2022	Novembro	28/11/2022	48	184	Cujubim Grande	P01	Culicidae	Culicinae	*Culex*	*Culex sp.*	1
2022	Dezembro	05/12/2022	49	185	Samauma	P08	Culicidae	Culicinae	*Culex*	*Culex sp.*	12
2022	Dezembro	05/12/2022	49	185	Samauma	P08	Culicidae	Culicinae	*Mansonia*	*Mansonia sp.*	28
2022	Dezembro	05/12/2022	49	185	Morrinhos	P04	Culicidae	Culicinae	*Mansonia*	*Mansonia sp.*	22
2022	Dezembro	05/12/2022	49	185	Teotônio	P03	Culicidae	Anophelinae	*Anopheles*	*Anopheles sp.*	2
2022	Dezembro	05/12/2022	49	185	Teotônio	P03	Culicidae	Culicinae	*Mansonia*	*Mansonia sp.*	3
2022	Dezembro	05/12/2022	49	185	Teotônio	P03	Culicidae	Culicinae	*Culex*	*Culex sp.*	1
2022	Dezembro	05/12/2022	49	185	Teotônio	P03	Culicidae	Culicinae	*Coquillettidia*	*Coquillettidia sp.*	6
2022	Dezembro	05/12/2022	49	185	São Domingos	P02	Culicidae	Culicinae	*Mansonia*	*Mansonia sp.*	6
2022	Dezembro	05/12/2022	49	185	Cujubim Grande	P01	Culicidae	Culicinae	*Mansonia*	*Mansonia sp.*	1
2022	Dezembro	05/12/2022	49	185	Cujubim Grande	P01	Culicidae	Culicinae	*Culex*	*Culex sp.*	2
2022	Dezembro	12/12/2022	50	186	Samauma	P08	Culicidae	Culicinae	*Mansonia*	*Mansonia sp.*	8
2022	Dezembro	12/12/2022	50	186	Samauma	P08	Culicidae	Culicinae	*Culex*	*Culex sp.*	7
2022	Dezembro	12/12/2022	50	186	Samauma	P08	Culicidae	Culicinae	*Aedes*	*Aedes sp.*	5
2022	Dezembro	12/12/2022	50	186	Morrinhos	P04	Culicidae	Culicinae	*Mansonia*	*Mansonia humeralis*	2
2022	Dezembro	12/12/2022	50	186	Teotônio	P03	Culicidae	Culicinae	*Culex*	*Culex sp.*	2
2022	Dezembro	12/12/2022	50	186	Teotônio	P03	Culicidae	Culicinae	*Coquillettidia*	*Coquillettidia sp.*	5
2022	Dezembro	12/12/2022	50	186	Teotônio	P03	Culicidae	Culicinae	*Mansonia*	*Mansonia sp.*	1
2022	Dezembro	12/12/2022	50	186	São Domingos	P02	Culicidae	Culicinae	*Mansonia*	*Mansonia sp.*	47
2022	Dezembro	12/12/2022	50	186	São Domingos	P02	Culicidae	Culicinae	*Coquillettidia*	*Coquillettidia sp.*	6
2022	Dezembro	12/12/2022	50	186	São Domingos	P02	Culicidae	Culicinae	*Culex*	*Culex sp.*	12
2022	Dezembro	19/12/2022	51	187	Samauma	P08	Culicidae	Culicinae	*Mansonia*	*Mansonia sp.*	10
2022	Dezembro	19/12/2022	51	187	Samauma	P08	Culicidae	Culicinae	*Culex*	*Culex sp.*	5
2022	Dezembro	19/12/2022	51	187	Morrinhos	P04	Culicidae	Culicinae	*Mansonia*	*Mansonia sp.*	75
2022	Dezembro	19/12/2022	51	187	Teotônio	P03	Culicidae	Culicinae	*Mansonia*	*Mansonia sp.*	7
2022	Dezembro	19/12/2022	51	187	Teotônio	P03	Culicidae	Culicinae	*Coquillettidia*	*Coquillettidia sp.*	4
2022	Dezembro	19/12/2022	51	187	Teotônio	P03	Culicidae	Culicinae	*Culex*	*Culex sp.*	2
2022	Dezembro	19/12/2022	51	187	São Domingos	P02	Culicidae	Culicinae	*Mansonia*	*Mansonia sp.*	26
2022	Dezembro	19/12/2022	51	187	São Domingos	P02	Culicidae	Culicinae	*Culex*	*Culex sp.*	2
2022	Dezembro	19/12/2022	51	187	São Domingos	P02	Culicidae	Culicinae	*Coquillettidia*	*Coquillettidia sp.*	2
2022	Dezembro	19/12/2022	51	187	Cujubim Grande	P01	Culicidae	Anophelinae	*Anopheles*	*Anopheles sp.*	1
2022	Dezembro	26/12/2022	52	188	Samauma	P08	Culicidae	Culicinae	*Mansonia*	*Mansonia sp.*	4
2022	Dezembro	26/12/2022	52	188	Samauma	P08	Culicidae	Culicinae	*Culex*	*Culex sp.*	2
2022	Dezembro	26/12/2022	52	188	Morrinhos	P04	Culicidae	Culicinae	*Mansonia*	*Mansonia sp.*	22
2022	Dezembro	26/12/2022	52	188	Morrinhos	P04	Culicidae	Culicinae	*Culex*	*Culex sp.*	5
2022	Dezembro	26/12/2022	52	188	Teotônio	P03	Culicidae	Anophelinae	*Anopheles*	*Anopheles sp.*	1
2022	Dezembro	26/12/2022	52	188	Teotônio	P03	Culicidae	Culicinae	*Mansonia*	*Mansonia humeralis*	6
2023	Janeiro	03/01/2023	1	189	Morrinhos	P04	Culicidae	Culicinae	*Mansonia*	*Mansonia sp.*	107
2023	Janeiro	03/01/2023	1	189	Morrinhos	P04	Culicidae	Culicinae	*Culex*	*Culex sp.*	6
2023	Janeiro	03/01/2023	1	189	Morrinhos	P04	Culicidae	Culicinae	*Aedes*	*Aedes sp.*	2
2023	Janeiro	03/01/2023	1	189	Teotônio	P03	Culicidae	Culicinae	*Mansonia*	*Mansonia sp.*	8
2023	Janeiro	03/01/2023	1	189	Teotônio	P03	Culicidae	Culicinae	*Culex*	*Culex sp.*	5
2023	Janeiro	03/01/2023	1	189	Teotônio	P03	Culicidae	Culicinae	*Coquillettidia*	*Coquillettidia sp.*	4
2023	Janeiro	03/01/2023	1	189	São Domingos	P02	Culicidae	Anophelinae	*Anopheles*	*Anopheles sp.*	1
2023	Janeiro	03/01/2023	1	189	São Domingos	P02	Culicidae	Culicinae	*Mansonia*	*Mansonia sp.*	11
2023	Janeiro	03/01/2023	1	189	São Domingos	P02	Culicidae	Culicinae	*Culex*	*Culex sp.*	3
2023	Janeiro	03/01/2023	1	189	São Domingos	P02	Culicidae	Culicinae	*Limatus*	*Limatus durhamii*	2
2023	Janeiro	03/01/2023	1	189	Cujubim Grande	P01	Culicidae	Culicinae	*Culex*	*Culex sp.*	1
2023	Janeiro	03/01/2023	1	189	Cujubim Grande	P01	Culicidae	Anophelinae	*Anopheles*	*Anopheles sp.*	3
2023	Janeiro	09/01/2023	2	190	Samauma	P08	Culicidae	Culicinae	*Mansonia*	*Mansonia titillans*	3
2023	Janeiro	09/01/2023	2	190	Morrinhos	P04	Culicidae	Culicinae	*Mansonia*	*Mansonia sp.*	44
2023	Janeiro	09/01/2023	2	190	Morrinhos	P04	Culicidae	Culicinae	*Culex*	*Culex sp.*	2
2023	Janeiro	09/01/2023	2	190	Teotônio	P03	Culicidae	Culicinae	*Mansonia*	*Mansonia sp.*	101
2023	Janeiro	09/01/2023	2	190	Teotônio	P03	Culicidae	Culicinae	*Coquillettidia*	*Coquillettidia sp.*	35
2023	Janeiro	09/01/2023	2	190	Teotônio	P03	Culicidae	Culicinae	*Culex*	*Culex sp.*	72
2023	Janeiro	09/01/2023	2	190	Teotônio	P03	Culicidae	Anophelinae	*Anopheles*	*Anopheles sp.*	15
2023	Janeiro	09/01/2023	2	190	São Domingos	P02	Culicidae	Culicinae	*Mansonia*	*Mansonia sp.*	12
2023	Janeiro	09/01/2023	2	190	São Domingos	P02	Culicidae	Culicinae	*Coquillettidia*	*Coquillettidia sp.*	18
2023	Janeiro	09/01/2023	2	190	São Domingos	P02	Culicidae	Culicinae	*Culex*	*Culex sp.*	10
2023	Janeiro	09/01/2023	2	190	Cujubim Grande	P01	Culicidae	Culicinae	*Culex*	*Culex sp.*	1
2023	Janeiro	16/01/2023	3	191	Samauma	P08	Culicidae	Culicinae	*Mansonia*	*Mansonia sp.*	6
2023	Janeiro	16/01/2023	3	191	Samauma	P08	Culicidae	Culicinae	*Culex*	*Culex sp.*	1
2023	Janeiro	16/01/2023	3	191	Morrinhos	P04	Culicidae	Culicinae	*Culex*	*Culex sp.*	5
2023	Janeiro	16/01/2023	3	191	Morrinhos	P04	Culicidae	Culicinae	*Mansonia*	*Mansonia sp.*	121
2023	Janeiro	16/01/2023	3	191	Teotônio	P03	Culicidae	Culicinae	*Mansonia*	*Mansonia sp.*	11
2023	Janeiro	16/01/2023	3	191	Teotônio	P03	Culicidae	Culicinae	*Culex*	*Culex sp.*	6
2023	Janeiro	16/01/2023	3	191	Teotônio	P03	Culicidae	Culicinae	*Coquillettidia*	*Coquillettidia sp.*	3
2023	Janeiro	16/01/2023	3	191	São Domingos	P02	Culicidae	Culicinae	*Culex*	*Culex sp.*	3
2023	Janeiro	16/01/2023	3	191	São Domingos	P02	Culicidae	Culicinae	*Mansonia*	*Mansonia sp.*	9
2023	Janeiro	16/01/2023	3	191	São Domingos	P02	Culicidae	Culicinae	*Aedes*	*Aedes sp.*	1
2023	Janeiro	16/01/2023	3	191	Cujubim Grande	P01	Culicidae	Anophelinae	*Anopheles*	*Anopheles nuneztovari s.l.*	1
2023	Janeiro	24/01/2023	4	192	Samauma	P08	Culicidae	Culicinae	*Mansonia*	*Mansonia sp.*	1
2023	Janeiro	24/01/2023	4	192	Morrinhos	P04	Culicidae	Culicinae	*Mansonia*	*Mansonia sp.*	142

2023	Janeiro	24/01/2023	4	192	Morrinhos	P04	Culicidae	Culicinae	*Culex*	*Culex sp.*	27
2023	Janeiro	24/01/2023	4	192	Teotônio	P03	Culicidae	Culicinae	*Mansonia*	*Mansonia sp.*	6
2023	Janeiro	24/01/2023	4	192	Teotônio	P03	Culicidae	Culicinae	*Coquillettidia*	*Coquillettidia sp.*	3
2023	Janeiro	24/01/2023	4	192	Teotônio	P03	Culicidae	Culicinae	*Culex*	*Culex sp.*	2
2023	Janeiro	24/01/2023	4	192	Cujubim Grande	P01	Culicidae	Culicinae	*Mansonia*	*Mansonia sp.*	1
