# Supplementary material for: Trends of Mansonia (Diptera, Culicidae, Mansoniini) in Porto Velho: Seasonal patterns and meteorological influences
Source: PLoS One. 2024 May 8;19(5):e0303405. doi: 10.1371/journal.pone.0303405 (PMC11078429; doi:10.1371/journal.pone.0303405)
Supplement: Attachment — Submitted filename: Response-to-reviewers.docx [file pone.0303405.s002.docx]

# **Rebuttal letter**

Subject: Response to Review for Article Submission PONE-D-23-44055

Dear Editor,

I hope this email finds you well. We sincerely appreciate the thorough review conducted by the esteemed reviewers of our article titled "**Seasonality of *Mansonia* Blanchard (Diptera, Culicidae, Mansoniini) in Porto Velho, State of Rondônia, Brazil**" with submission number **PONE-D-23-44055**.

To address the questions raised, we have conducted a thorough, point-by-point analysis to provide comprehensive clarification for each query. Please find the detailed responses attached to this email. We hope these clarifications meet the reviewers' expectations.

Furthermore, we would like to request a change in the title of our work to: "**Trends of *Mansonia* (Diptera, Culicidae, Mansoniini) in Porto Velho: Seasonal Patterns and Meteorological Influences**". This change, agreed by the other co-authors, aims to avoid confusion with another published article that addressed a similar issue, but using a different sampling technique (HLC) “**Seasonality and Biting Behavior of *Mansonia* (Diptera, Culicidae) in Rural Settlements Near Porto Velho, State of Rondônia, Brazil**”. We believe that this new designation better aligns with the purpose of the article presented.

Thank you for your continued support and guidance throughout this process.

Yours sincerely

Dr. José F. Saraiva

**Editor**

1. Please ensure that your manuscript meets PLOS ONE's style requirements, including those for file naming.

Answer: done.

2. Please note that PLOS ONE has specific guidelines on code sharing for submissions in which author-generated code underpins the findings in the manuscript. In these cases, all author-generated code must be made available without restrictions upon publication of the work. Please review our guidelines at https://journals.plos.org/plosone/s/materials-and-software-sharing#loc-sharing-code and ensure that your code is shared in a way that follows best practice and facilitates reproducibility and reuse.

Answer: done.

3. In your Methods section, please provide additional information regarding the permits you obtained for the work. Please ensure you have included the full name of the authority that approved the field site access and, if no permits were required, a brief statement explaining why.

Answer: done.

4. Thank you for stating the following financial disclosure:

"Research and Development project from Santo Antônio Energia (ANEEL project CT.PD.124.2018)."

Please state what role the funders took in the study. If the funders had no role, please state: ""The funders had no role in study design, data collection and analysis, decision to publish, or preparation of the manuscript.""

If this statement is not correct you must amend it as needed.

Please include this amended Role of Funder statement in your cover letter; we will change the online submission form on your behalf.

Answer: done.

5. We note that your Data Availability Statement is currently as follows: All relevant data are within the manuscript and its Supporting Information files.

Please confirm at this time whether or not your submission contains all raw data required to replicate the results of your study. Authors must share the “minimal data set” for their submission. PLOS defines the minimal data set to consist of the data required to replicate all study findings reported in the article, as well as related metadata and methods (https://journals.plos.org/plosone/s/data-availability#loc-minimal-data-set-definition).

For example, authors should submit the following data:

- The values behind the means, standard deviations and other measures reported;

- The values used to build graphs;

- The points extracted from images for analysis.

Authors do not need to submit their entire data set if only a portion of the data was used in the reported study.

If your submission does not contain these data, please either upload them as Supporting Information files or deposit them to a stable, public repository and provide us with the relevant URLs, DOIs, or accession numbers. For a list of recommended repositories, please see https://journals.plos.org/plosone/s/recommended-repositories.

If there are ethical or legal restrictions on sharing a de-identified data set, please explain them in detail (e.g., data contain potentially sensitive information, data are owned by a third-party organization, etc.) and who has imposed them (e.g., an ethics committee). Please also provide contact information for a data access committee, ethics committee, or other institutional body to which data requests may be sent. If data are owned by a third party, please indicate how others may request data access.

Answer: done.

6. When completing the data availability statement of the submission form, you indicated that you will make your data available on acceptance. We strongly recommend all authors decide on a data sharing plan before acceptance, as the process can be lengthy and hold up publication timelines. Please note that, though access restrictions are acceptable now, your entire data will need to be made freely accessible if your manuscript is accepted for publication. This policy applies to all data except where public deposition would breach compliance with the protocol approved by your research ethics board. If you are unable to adhere to our open data policy, please kindly revise your statement to explain your reasoning and we will seek the editor's input on an exemption. Please be assured that, once you have provided your new statement, the assessment of your exemption will not hold up the peer review process.

Answer: done.

7. We note that Figure 1 in your submission contain map/satellite images which may be copyrighted. All PLOS content is published under the Creative Commons Attribution License (CC BY 4.0), which means that the manuscript, images, and Supporting Information files will be freely available online, and any third party is permitted to access, download, copy, distribute, and use these materials in any way, even commercially, with proper attribution. For these reasons, we cannot publish previously copyrighted maps or satellite images created using proprietary data, such as Google software (Google Maps, Street View, and Earth). For more information, see our copyright guidelines: http://journals.plos.org/plosone/s/licenses-and-copyright.

We require you to either (1) present written permission from the copyright holder to publish these figures specifically under the CC BY 4.0 license, or (2) remove the figures from your submission:

a. You may seek permission from the original copyright holder of Figure 1 to publish the content specifically under the CC BY 4.0 license.

We recommend that you contact the original copyright holder with the Content Permission Form (http://journals.plos.org/plosone/s/file?id=7c09/content-permission-form.pdf) and the following text:

“I request permission for the open-access journal PLOS ONE to publish XXX under the Creative Commons Attribution License (CCAL) CC BY 4.0 (http://creativecommons.org/licenses/by/4.0/). Please be aware that this license allows unrestricted use and distribution, even commercially, by third parties. Please reply and provide explicit written permission to publish XXX under a CC BY license and complete the attached form.”

Please upload the completed Content Permission Form or other proof of granted permissions as an ""Other"" file with your submission.

In the figure caption of the copyrighted figure, please include the following text: “Reprinted from [ref] under a CC BY license, with permission from [name of publisher], original copyright [original copyright year].”

Answer: adjusted.

b. If you are unable to obtain permission from the original copyright holder to publish these figures under the CC BY 4.0 license or if the copyright holder’s requirements are incompatible with the CC BY 4.0 license, please either i) remove the figure or ii) supply a replacement figure that complies with the CC BY 4.0 license. Please check copyright information on all replacement figures and update the figure caption with source information. If applicable, please specify in the figure caption text when a figure is similar but not identical to the original image and is therefore for illustrative purposes only.

The following resources for replacing copyrighted map figures may be helpful:

Answer: checked.

**Reviewer #1:**

Abstract
Pg. 2. First sentence is too long and could be better written to improve comprehension. Suggestion: "Entomological research is vital for shaping strategies to control mosquito vectors. Its significance also reaches into environmental management, aiming to prevent inconveniences caused by non-vector mosquitoes like the Mansonia Blanchard mosquito."
Answer: suggestion accepted.

Pg. 2. Where it says: "In this study, we carried out a five-year comprehensive monitoring.." I would recommend writing the years of study in parenthesis. E.g.: "…we carried out a five-year (2019-2023)…".

Answer: done.

Pg. 2. Second sentence. There is too much repetition of the word location. Suggestion: "In this study, we conducted a comprehensive five-year (2019-2023) monitoring of these mosquitoes at ten sites in Porto Velho, Rondônia, using SkeeterVac SV3100 automatic traps positioned within the Madeira River hydroelectric complex."

Answer: done.

Pg. 2. Fourth sentence. "Notably, a significant decreasing trend in local abundance was
observed throughout the study." It would be interesting to inform which months or year was this significant decrease in the density of Mansonia spp.

Answer: information inserted in the sentence.

Introduction
Pg. 3. First sentence: "Entomological monitoring of mosquitoes represents an important part of public health data collection, as many of these insects are vectors of several human diseases." Mosquitoes are vector of pathogens not diseases. Suggestion: "Entomological monitoring of mosquitoes represents an important part of public health data collection, as many of these insects are vectors of several human pathogens."
Answer: change accepted.

Pg. 3. Delete the "– "symbol at the middle of the third sentence of the paragraph.
Answer: done.

Material and methods
Area of the study
Indicate in the Methods section the license number of the collection.
Answer: done.

What was the criterion used to decide on the distances between the collection areas?
The authors emphasize the importance of selecting a study area for comprehensive point sampling.
Answer:

Add sources for demography.
Answer: done.

In which entomological collection were the collected specimens deposited? Please provide this information along with their deposit numbers.
Answer: Entomological collection of the Instituto de Pesquisas Científicas e Tecnológicas do Estado do Amapá - IEPA. Deposit voucher numbers were inserted into the manuscript.

However, there are concerns about the methodology. Mosquitoes were extracted weekly from traps with a high specimen abundance, causing damage and compromised identifications. It's unclear why specimens weren't removed sooner. Identifying species richness is challenging, especially as many specimens were only identified to the genus level (Mansonia sp.) due to drying out in traps.

Answer: The decision to conduct a weekly inspection of the traps was motivated mainly by the significant distance between them, as exemplified by the distance of 155 km between Cujubim Grande and Jaci Paraná. Moreover, most traps were installed on unpaved rural roads, making it even more challenging to move between points to remove mosquitoes. Faced with these adversities and considering the limited resources available to cover the extensive distance, we inspected the traps weekly.

It should be noted that this activity was carried out by two technicians using one vehicle. Each inspection took the entire day, starting at 7:00 in the morning and ending at 6:00 in the evening. Although we recognize that this prolonged time interval may have resulted in damage to the specimens, we emphasize that this was the most feasible and viable period to cover the entire area of interest of the study.

Figure 1. The legend of the map shows the color of Brazil as white, however the country appears as green, please correct it.
Answer: adjusted.

Pg. 5. First line of the third paragraph. Where it says: "The automatic traps were installed at ten points, each positioned in the surroundings of the selected residences." How many residencies? Earlier on it mentions that the locations chosen for the study are characterized as rural, semi-rural, or peri-urban environments, how many traps were placed in each one of these classifications of level of urbanization?
Answer: A total of 10 traps were used. Each trap was installed in the peri-domestic area of the selected residence, i.e., 10 residences. To summarize the number of traps per environment, we provided a table showing the geographic location of the traps, land use, and straight-line distances from water bodies closest to the point.

Pg. 5. It is not mandatory, however, since the SkeeterVac SV3100 Automatic Traps are a new technology not used extensively, it would be more informative to add a picture of this trap and describe better its different parts, giving more details about its design and structure.
Answer: We understand the importance of clarifying the design of the SkeeterVac trap. Therefore, we have improved the section that describes the trap in detail and included specific references to the design used. We believe this information is comprehensive enough so anyone interested in the trap can easily find and purchase it.

Pg. 5. It appears the traps were installed in different time periods in each location. Ideally for a correct statistical analysis all locations, to be compared to one another, should have the same period. How is it possible to compare July 2019 from locations: Jaci Paraná, Teotônio, and São Domingos if you don't have data from the same month and year from the other locations (e.g. Cujubim Grande, Morrinhos, Rio Contra, Samauma, and Santa Rita)?
Answer: Comparisons between locations were conducted, taking into account the same time interval in which the traps were in operation. In the case of traps installed before and that remained in operation until the end of the study, when comparing them with traps installed later, we proceeded with cutting the initial data from the first trap. This same approach was applied to traps that were removed after the start of the study, with the final data excluded from the analyses.

Pg. 6 Second paragraph under "Processing and taxonomic identification of Mosquitoes" says: "Taxonomic determination took place down to the genus level due to the low quality of the specimens when they were removed from the trap compartment." Than latter on in the text under "Data Analysis" it says: "Initially, the database was statistically explored to obtain quantity, mean, and frequency metrics per species of mosquitoes…". It could not have been "per species" because they were only identified to genus level right? Please correct it.
Answer: adjusted.

Pg. 8. Second paragraph. Please revise the presence of em dash "—" in this paragraph, is it necessary or was it a typing error. Also, where it says: "…revealed the presence of two Mansonia species each, and Morrinho, Santa Rita Joana D'arc line 15, Samauma.." is "line 15" part of the name of the location? If not, please delete it.

Answer: adjusted.

Table 2 lacks clarity and doesn't quantify identified species, hindering understanding. Clarifications in the table are needed for a more straightforward presentation of the study's findings.
Answer: The table has been corrected to show all species identified in the study.

Pg. 9 The paragraph starting with "Considering the geographic location…" Seems to belong in the discussion part of the manuscript, since it justifies the results obtained. Consider if this is a change worth making.
Answer: adjusted.

Pg 9. Where it says: "…obtained in the Joana D'Arc settlement is justified by the geographic proximity between these two collection points (14 km). The same can be said between Morrinhos and Teotônio (20 km)." Were these distances at random? I don't recall seeing the trap distances in the Material and Methods section of the manuscript.
Answer: Distance values refer to straight-line measurements. We have included further clarifications about this measure in the text.

Pg. 9 Under "Time series and forecast" where it says: "The decomposition of the historical series…." A better word choice could be made in this sentence. Suggestion: "The breakdown and analysis of the historical data series gathered through the SkeeterVac SV3100 over a five-year period is presented in Figure 5."
Answer: "decomposition" is a term generally used for time series analyses. However, we will accept the change to "breakdown".

Pg 9. Second sentence of the last paragraph on page nine. Could be improved. Suggestion: "The peaks of Mansonia mosquitoes' abundance were more pronounced in the initial years of the study (2019-2020)."
Answer: done.

Pg 11. Correct "CO2" to "CO_2_".
Answer: done. CO_2_

Discussion
The discussion is extensive, however, lacks important results obtained in the study. It does not discuss the results regarding wind speed being the most relevant meteorological factor influencing the abundance of *Mansonia*.
Answer: We consider the discussion to be of good size. Furthermore, we discuss the wind results on line 359, with results found by Alencar et al. 2021, who carried out studies with dispersion in the same area of study.

Pg 11. Last sentence of the last paragraph on page ten that goes on to page eleven. Where it says: "Furthermore*, Aedes aegypti* (Linnaeus, 1762) and *Aedes albopictus* (Skuse, 1895) are invasive species in the Amazon region." Were these species collected in this study?
Answer: Yes.

Pg. 11. First paragraph mentions Anopheles darlingi, Haemagogus janthinomys, Aedes aegypti and Aedes albopictus, were all these species collected in this study? If not, it would be important to focus on Mansonia and not other species. At the end of the parahraph it mentions deforestation, how do the results from the different levels of urbanization mentioned at the beginning (rural, semi-rural, or peri-urban environments) play a role in mosquito abundance? It would be interesting to mention this here.

Answer: All species except *Haemagogus janthinomys* were included in our study's sampling. We specifically mentioned *Hg. janthinomys* to highlight the significance of native wild species in the pathogen transmission dynamics of Amazon region.

Regarding "deforestation, how do the results of the different levels of urbanization mentioned at the beginning (rural, semi-rural or peri-urban environments) play a role in mosquito abundance?" - the paragraph was adjusted to better understand this relationship.

The authors could insert the paragraph that starts with: "Our entomological monitoring revealed that the area’s most prone to abundance of Mansonia spp. are located close to cattle farms, areas of forest deforestation, small lakes…" Right after this one, it seems that this is the information that was lacking.

Answer: done.

**Reviewer #2:**

The manuscript needs to be reviewed by either an editing company or by an English native speaker.
Answer: One of our co-authors, Dr.Ahana Maitra, is a native/bilingual speaker. She has thoroughly reviewed the manuscript for language. Moreover, the manuscript has also been reviewed by the editing company @Stylus–California–USA (<https://www.linkedin.com/company/atstylus/>) for English language editing.

In addition, authors must either deposit their data set in Dryad or other platform that gives a doi number for the data set.
Answer: The other co-authors of the manuscript did not agree to make the entire database available to the public because it contains information that will be analyzed later in future publications of the project. However, we agree to provide the information used in this study only as supplementary material for consultation by reviewers and readers of the article.

Authors declared no conflict of interest. However, their study was financially supported by Research and Development project from Santo Antônio Energia (ANEEL project CT.PD.124.2018). Also, the field collections were carried out in locations in the vicinities of the reservoir lake of the same company that gave them the financial support Santo Antônio (SAE). They should clarify this important conflict.
Answer: Thank you for your careful observation. We acknowledge that the study received financial support from the Santo Antônio Energia Research and Development project (ANEEL project CT.PD.124.2018), and field collections were carried out in locations close to the reservoir lake of the same company that granted the financial support. We clarify that, although we received this financial support, the company did not influence the data analysis, writing of the manuscript, or the decision to publish this article. We conducted our research with autonomy and scientific integrity, ensuring impartiality in both the conduct and presentation of results. Our commitment is to provide transparent information and uphold the credibility of our work.
Statistical analyses are adequate and defined based on the major objectives of the study and data they have at hand. The number of mosquitoes they collected in five years using 10 traps and a few locations is impressive (153,125 mosquitoes were sampled in 10 SkeeterVac
SV3100 traps. The genus Mansonia was the most abundant, with 82,819 (54.09%) specimens, followed by Culex with 63,521 (41.48%) and Coquillettidia with 3,305 (2.16%).

It is not clear why authors did not separate the species in the study and in Table 2. If they identified a certain number of species in each genus; I assume they know which species they have at hand. I suggest they redo the analysis using the species level for Mansonia (the focus of the study) as far as possible, keeping the genus analyses they already did. The stud will be sounder and bring to light new information on species associated with lake reservoirs of hydroelectric power plants.

Answer: Table 2 has been revised to reflect all species identified during the study. It is essential to clarify that, due to limitations in the preservation of specimens collected in the trap, many of our specimens could only be safely identified to the genus level. Furthermore, I emphasize that most specific identifications in the genus Mansonia have been based mainly on the analysis of male genitalia.

Therefore, we decided not to conduct the proposed analyses for the species, as this would result in a significant bias toward capturing male specimens of the genus Mansonia in the analyses. Our approach was adopted to maintain the integrity of the results, acknowledging the limitations in gender-level identification. We aim to avoid skewed conclusions stemming from an analysis predominantly based on single sex (male), typically found at low density in entomological inventories.
