# Supplementary material for: Trends of Mansonia (Diptera, Culicidae, Mansoniini) in Porto Velho: Seasonal patterns and meteorological influences
Source: PLoS One. 2024 May 8;19(5):e0303405. doi: 10.1371/journal.pone.0303405 (PMC11078429; doi:10.1371/journal.pone.0303405)
Supplement: Attachment — Submitted filename: Response-to-reviewers.docx [file pone.0303405.s003.docx]

# **Rebuttal letter**

Subject: Response to Review for Article Submission PONE-D-23-44055

Dear Editor,

I hope this email finds you well. I would like to express sincere thanks for the thorough review conducted by the esteemed reviewers regarding our manuscript "Trends of Mansonia (Diptera, Culicidae, Mansoniini) in Porto Velho: seasonal patterns and meteorological influences", with submission number PONE-D-23 -44055R1.

Below, we present the response to a question raised by reviewer 1. Additionally, we have included further clarifications on the methodology for selecting points in the materials and methods section, lines 122 to 126. We hope that this clarification satisfactorily addresses the query of the esteemed reviewer.

We sincerely appreciate your continued support and guidance throughout this process.

Yours sincerely

Dr. José F. Saraiva

**Reviewer #1:**

**What was the criterion used to decide on the distances between the collection areas?
The authors emphasize the importance of selecting a study area for comprehensive point sampling.**

Answer:

The SkeeterVac traps were strategically installed based on three main considerations: 1. the presence of human communities, 2. the prevalence of *Mansonia* mosquito attacks, and 3. the presence of potential breeding sites, such as stagnant water bodies with aquatic macrophytes. We used satellite images to identify and georeferenced the lakes and lagoons close to the communities with the most of *Mansonia* mosquitoes attacks. The breeding sites were further validated by on-site surveys. This facilitated the determination of distances between traps and the nearest breeding sites.

Following installation based on these criteria, the spacing between traps was measured. A minimum spacing of 5km was maintained between traps, as exemplified by P08-P07-P06, while the greatest distances recorded were between points P05 and P06 (32km) and between points P01 and P02 (37) km. This spacing ensured no overlap in sampling, considering that the reported flight radius for *Mansonia* is on average 2km (de Mello et al., 2021).

Reference

de Mello, C.F., Alencar, J. Dispersion pattern of *Mansonia* in the surroundings of the Amazon Jirau Hydroelectric Power Plant. *Sci Rep* **11**, 24273 (2021). https://doi.org/10.1038/s41598-021-03682-1
